# Complete chloroplast genomes of eight *Delphinium* taxa (Ranunculaceae) endemic to Xinjiang, China: insights into genome structure, comparative analysis, and phylogenetic relationships

**DOI:** 10.1186/s12870-024-05279-y

**Published:** 2024-06-26

**Authors:** Chunfeng Song, Junwen Zhu, Huimin Li

**Affiliations:** Jiangsu Key Laboratory for the Research and Utilization of Plant Resources, Institute of Botany, Jiangsu Province and Chinese Academy of Sciences (Nanjing Botanical Garden Mem. Sun Yat- Sen), Nanjing, 210014 Jiangsu China

**Keywords:** China, Chloroplast genome, Comparative analysis, *Delphinium*, Phylogeny, Xinjiang

## Abstract

**Background:**

*Delphinium* L. represents a taxonomically intricate genus of significant phylogenetic and economic importance in Ranunculaceae. Despite the existence of few chloroplast genome datasets, a comprehensive understanding of genome structures and selective pressures within the genus remains unknown. Furthermore, several taxa in this genus are exclusively found in Xinjiang, China, a region renowned for its distribution and diversity of Chinese and Central Asian *Delphinium* species. Therefore, investigating the features of chloroplast genomes in this area will provide valuable insights into the evolutionary processes and phylogenetic relationships of the genus.

**Results:**

In this study, the eight newly completed chloroplast genomes are examined, ranging in length from 153,979 bp to 154,284 bp. Alongside these, analysing six previously reported taxa re-annotated in *Delphinium*, 111 unique genes are identified across all samples. Genome structure, distributions of simple sequence repeats and short dispersed repeats, as well as gene content are similar among these *Delphinium* taxa. Nine hypervariable intergenic spacers and protein coding regions, including *ndh*F-*trn*L^(TAG)^, *rpl*16-intron, *rpl*33, *rps*15, *rps*18, *trn*K^(TTT)^-*trn*Q^(TTG)^, *trn*P^(TGG)^-*psa*J, *trn*T^(GGT)^-*psb*D and *ycf*1, are identified among 13 perennial *Delphinium*. Selective pressure and codon usage bias of all the plastid genes are performed within 14 *Delphinium* taxa. Phylogenetic analysis based on 14 *Delphinium* plastomes, alongside two *Aconitum* (Ranunculaceae) species serving as outgroup taxa, reveals the monophyletic nature of *Delphinium*. Our findings further discern *Delphinium* into two distinct clades: perennial species (clade I) and annual species (clade II). In addition, compared with the nrDNA ITS topology, cytological data and morphological characters, *D. mollifolium* and *D. maackianum* showed potential involvement in hybridization or polyploidization processes. Excluding these two species, the perennial *Delphinium* (clade I) exhibits a stronger consistency with the morphology-based system that utilized seed morphology.

**Conclusion:**

This study represents the first comprehensive analysis of plastomic variations among *Delphinium* taxa, based on the examination of 14 complete plastomes. The chloroplast genome structure of *Delphinium* is similar to other angiosperms and possesses the typical quadripartite structure with the conserved genome arrangement and gene features. In addition, the variation of non-coding regions is larger than coding regions of the chloroplast genome. Through DNA sequence divergence across *Delphinium* plastomes and subsequent phylogenomic analyses *ndh*F-*trn*L^(TAG)^ and *ycf*1 are identified as promising molecular markers. These highly variable loci held significant potential for future phylogenetic and phylogeographic studies on *Delphinium*. Our phylogenomic analyses based on the whole plastomes, concatenation of 132 unique intergenic spacer regions, concatenation of 77 unique protein-coding genes and nrDNA ITS, all support the monophyly of *Delphinium* and perennial taxa clusters together into one clade within this genus. These findings provide crucial data for systematic, phylogenomic and evolutionary research in the genus for future studies.

**Supplementary Information:**

The online version contains supplementary material available at 10.1186/s12870-024-05279-y.

## Introduction

The Genus *Delphinium* L. (Ranunculaceae) was established by Linnaeus in 1753 [1] and initially comprised only six species. Later, in 1842, De Candolle [2] introduced a classification system that divided 53 contemporaneous species into four sections, namely *D.* sect. *Consolida* DC., *D.* sect. *Delphinellum* DC., *D.* sect. *Staphisagria* DC. and *D.* sect. *Delphinastrum* DC., based on the characters of flowers, carpel and growth cycle. This classification was widely accepted by many taxonomists later [3–8]. As time passed, the perennial group *D.* sect. *Delphinastrum* became the largest part of the genus, comprising around 364 species [8]. However, due to the significant morphological variability and the increasing number of species, constructing clear infrageneric divisions within *D.* sect. *Delphinastrum* remained challenging and contentious [6–9].

*Delphinium* was widely distributed in the Northern Hemisphere and tropical African mountains, with approximately 500 species, more than 150 of which were native to China [6, 10–12]. Among the Chinese *Delphinium* species, the majority were perennial herbs, with only two taxa being annual [6, 11, 12]. Moreover, the taxa distributed in Xinjiang stand out as a significant distribution centre for *Delphinium* species around China and central Asia, with around 15 taxa [13, 14]. Additionally, *Delphinium* plants in China, especially in Xinjiang, had a rich history of traditional medicinal use in folk medicine, where they were used to treat various conditions such as bruises, rheumatism, toothache, and enteritis [15, 16]. They also contained chemical constituents, including flavonoids and sterols, known for their physiological activities [17]. Furthermore, some *Delphinium* species, like *D. yunnanense* Franch. and *D. grandiflorum* L., were highly valued for their ornamental qualities, highlighting the economic significance of *Delphinium* in terms of development and utilization [18, 19].

The taxonomic and phylogenetic study of *Delphinium* presented challenges within the Ranunculaceae family [20–22]. The morphological variability and large number of species made it difficult to establish clear infrageneric divisions, especially within the perennial species that dominated the genus [8, 9, 20–22]. Although two crucial morphological characteristics like staminode color (black vs. blue) and seed morphology (seeds winged along angles vs. squamulose winged) had been used to group the perennials, their lack of correlation had led to conflicting classifications proposed by different authors [6, 8]. Traditionally, Chinese *Delphinium* species were classified into five sections based on various morphological features mainly related to the staminode color, combined with the shape of leaf, the seed morphology and the growth cycle [6]. Both infrageneric classification and taxonomic inconsistency in taxa delimitation remained a challenge in the genus [6, 8, 13, 23–27]. Furthermore, recent taxonomic revisions in different regions of China, particularly in southwestern and northwestern areas, suggested a possible decrease in the number of *Delphinium* species. For example, *D. iliense* Huth and *D. naviculare* var. *lasiocarpum* W. T. Wang were discussed here; *D. conaense* W. T. Wang was treated as a synonym of *D. bhutanicum* Munz by Yuan and Yang [24], while they were recognized as distinct species by Wang and Warnock [6], Kletter and Kriechbaum [28], respectively.

In the last two decades, molecular studies mainly focused on the phylogeny of the tribe Delphineae in Ranunculaceae [9, 29–31], with rare concentrates specifically on the infrageneric relationships within *Delphinium*, especially the Chinese group. Despite previous efforts to elucidate infrageneric relationships within the genus, several chloroplast markers shed some light on evolutionary patterns, supporting the monophyly of the *Delphinium*. However, numerous interspecies relationships based on these markers remained unresolved [9, 29–31]. Moreover, conflicting results were demonstrated in several *Delphinium* taxa between the chloroplast markers and nuclear DNA phylogenies, suggesting that unresolved intrageneric relationship might be attributed to the limited phylogenetic data available for interspecific hybridization or chloroplast capture [9, 32]. For instance, Jabbour & Renner [29] conducted phylogenetic analyses based on three chloroplast (cp.) DNA (*trn*K-*mat*K, *trn*S-*trn*G, *trn*L-*trn*F) and nrDNA (ITS) data, recognizing eight perennial *Delphinium* from China and North America. However, these species were divided into two geographical distribution clades with weak support. Subsequently, the authors [30] expanded their sample size to include 98 perennial *Delphinium* species from around the world, including 18 Chinese *Delphinium* taxa, and revised the phylogeny of the tribe Delphinieae (Ranunculaceae) based on nrDNA ITS region and cpDNA *trn*L-*trn*F data. Despite this broader dataset, the relationships among taxa remained weakly supported within the perennial *Delphinium* group. In another study, Zuo [9] investigated the evolution of seed morphology and staminode color in Chinese *Delphinium* by sampling 90 populations representing 72 perennial species. The study employed six fragment chloroplast sequences and a single-copy nuclear gene. Although the phylogenetic tree constructed from the chloroplast sequences supported the proposed classification system based on seed morphology, most interspecies relationships remained unresolved, suggesting that several species, such as *D. gyalanum* C. Marquand & Airy Shaw, *D. giraldii* Diels, *D. pulanense* W. T. Wang, experienced hybridization or chloroplast capture.

Chloroplast genomes have been frequently used in systematics due to their conserved quadripartite structure, predominantly clonal inheritance along the maternal line, and highly variable even at low taxonomic levels [33]. However, currently, only seven Chinese *Delphinium* cp. genomes were available (https://www.ncbi.nlm.nih.gov), which limited our knowledge of the organization and evolution of plastomes in *Delphinium* and the tribe Delphinieae [34–38].

Thus, to address these challenges in Chinese *Delphinium*, this study firstly took a comprehensive approach by analyzing the complete cp. genomes of eight Chinese *Delphinium* taxa endemic to Xinjiang. These eight cp. genomes were combined and compared with previously reported plastomes of six other Chinese *Delphinium* species [33–37]. The aims were: (1) to present the structure of cp. genome in the eight Xinjiang *Delphinium* taxa; (2) to compare the global structural patterns, investigate and screen mutational hotspots, examine variations of simple sequence repeats (SSRs) and short dispersed repeat sequences (SDRs); (3) to calculate nucleotide diversity in Chinese *Delphinium* cp. genomes for future species determination; (4) to reconstruct phylogenetic relationships among *Delphinium* species using cp. genome and nrDNA ITS region data respectively, and identify more effective molecular markers through this analysis; (5) to test for the presence of adaptive evolution in all annotated genes by analyzing selective pressure and codon usage bias. The results of this study are expected to provide valuable information for phylogenetic and phylogeographic studies within *Delphinium* and contribute to the exploration and utilization of *Delphinium* plants.

## Results

### Chloroplast genome structure and features

**Fig. 1:**
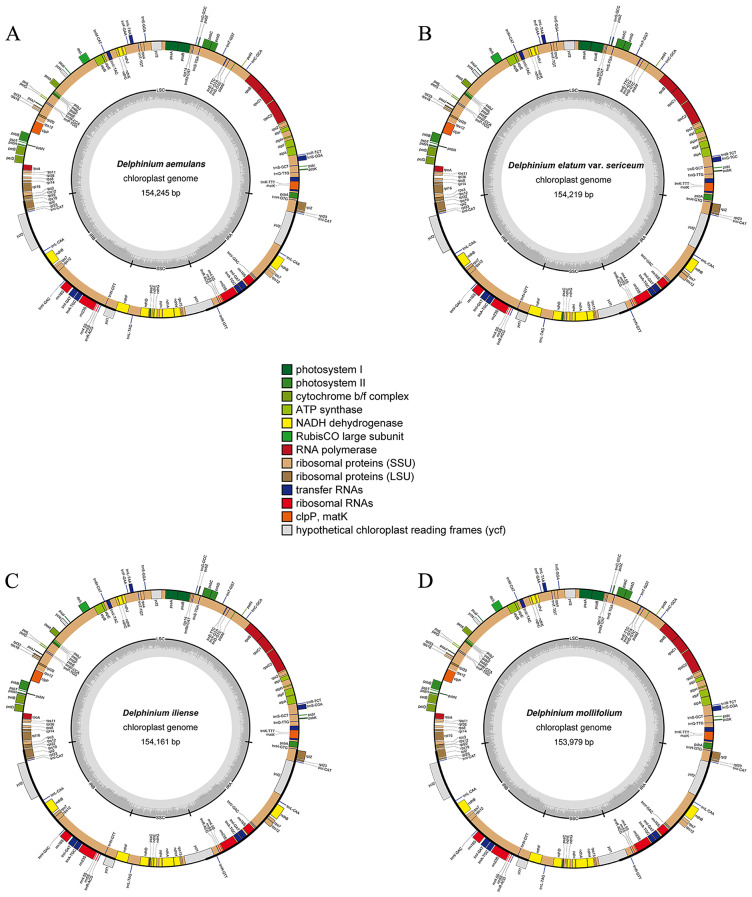
Plastomes of four *Delphinium* taxa, including *D. aemulans*, *D. elatum* var. *sericeum*, *D. iliense* and *D. mollifolium*. The outer circle shows the genes at each locus, and inverted repeat regions are indicated with thicker lines. Genes on the outside of the outer circle are transcribed in a counterclockwise direction, while genes on the inside of the outer circle are transcribed in a clockwise direction. The inner circle indicates the range of the large single-copy (LSC), small single-copy (SSC), and the inverted repeats (IRs), and also shows a GC content graph of the genome. In the GC content graph, the dark gray lines indicate GC content, while light gray lines indicate the AT content at each locus

**Fig. 2:**
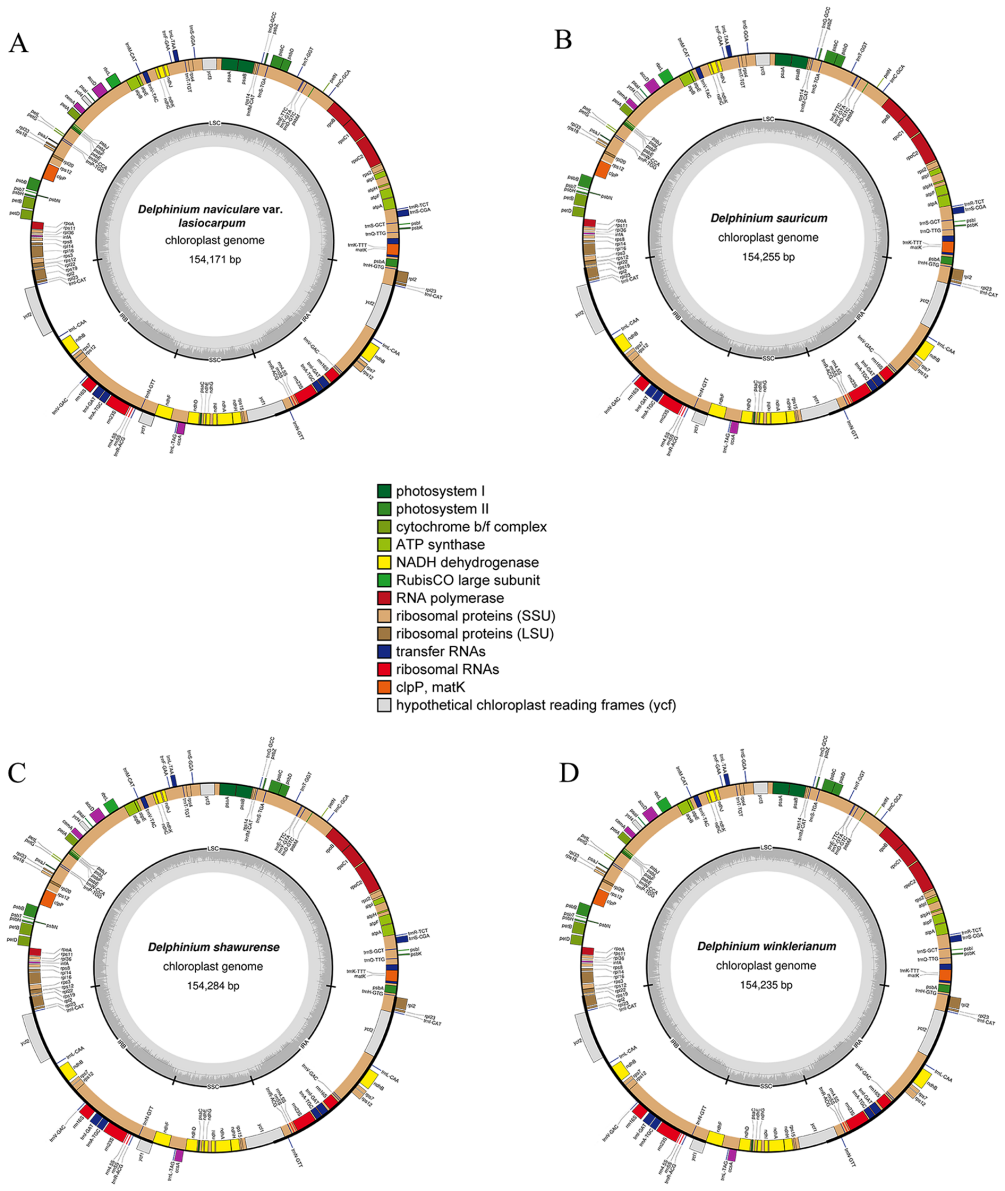
Plastomes of four *Delphinium* taxa, including *D. naviculare* var. *lasiocarpum*, *D. sauricum*, *D. shawurense* and *D. winklerianum*. The outer circle shows the genes at each locus, and inverted repeat regions are indicated with thicker lines. Genes on the outside of the outer circle are transcribed in a counterclockwise direction, while genes on the inside of the outer circle are transcribed in a clockwise direction. The inner circle indicates the range of the large single-copy (LSC), small single-copy (SSC), and the inverted repeats (IRs), and also shows a GC content graph of the genome. In the GC content graph, the dark gray lines indicate GC content, while light gray lines indicate the AT content at each locus

The chloroplast genomes of the eight taxa of *Delphinium* exhibited similar structure and organization (Table 1; Figs. 1 and 2). The length of eight cp. genomes varied from 153,979 bp in *D. mollifolium* W. T.Wang to 154,284 bp in *D. shawurense* W. T. Wang. They displayed a typical quadripartite circular structure containing a large single-copy (LSC) with lengths ranged from 84,648 bp (*D. iliense* Huth) to 85,018 bp (*D. mollifolium*), a small single-copy (SSC) with lengths varied from 16,293 bp (*D. winklerianum* Huth) to 16,342 bp (*D. shawurense*), and a pair of inverted repeats (IRs) with lengths between 26,331 bp (*D. mollifolium*) and 26,594 bp (*D. iliense*, *D. naviculare* var. *lasiocarpum* W. T. Wang and *D. sauricum* Schischk.). The total GC content was nearly close, varied from 38.25% to 38.27%.

**Table 1:** Summary of characteristics of plastome sequences used in this study, including eight new chloroplast genomes of the *Delphinium* taxa, * showing the newly

Species	GenBank numbers	Total genome size (bp)	Overall GC content (%)	LSC size (bp)	IR size (bp)	SSC size (bp)	NO. total gene (unique gene)	NO. protein-coding gene (unique gene)	NO. tRNA gene (unique gene)	NO. rRNA gene (unique gene)	Reference
*Aconitum brachypodum* Diels	MT584424	155,650	38.09	86,419	26,149	16,933	129 (111)	84 (77)	37 (30)	8 (4)	[39]
*A. delavayi* Franch.	OM289058	155,733	38.08	86,362	26,229	16,913	129 (111)	84 (77)	37 (30)	8 (4)	[50]
*Delphinium aemulans* Nevski (*)	OR263583	154,245	38.27	84,809	26,561	16,314	129 (111)	84 (77)	37 (30)	8 (4)	This study
*D. anthriscifolium* Hance	MK253461	155,077	38.14	85,871	25,977	17,252	129 (111)	84 (77)	37 (30)	8 (4)	[35]
*D. brunonianum* Royle	NC_051554	153,926	38.30	84,512	26,559	16,296	129 (111)	84 (77)	37 (30)	8 (4)	[36]
*D. candelabrum* var. *monanthum* (Hand.-Mazz.) W. T. Wang	MW246165	153,995	38.25	84,862	26,543	16,047	129 (111)	84 (77)	37 (30)	8 (4)	[36]
*D. ceratophorum* Franch.	MK253460	154,245	38.27	84,801	26,560	16,324	129 (111)	84 (77)	39 (31)	8 (4)	[35]
*D. elatum* var. *sericeum* W. T. Wang (*)	OR263584	154,219	38.28	84,780	26,561	16,317	129 (111)	84 (77)	37 (30)	8 (4)	This study
*D. iliense* Huth (*)	OR263586	154,161	38.27	84,648	26,594	16,325	129 (111)	84 (77)	37 (30)	8 (4)	This study
*D. maackianum* Regel	NC_047293	154,484	38.13	85,055	26,564	16,301	129 (111)	84 (77)	37 (30)	8 (4)	[37]
*D. mollifolium* W. T. Wang (*)	OR263588	153,979	38.25	85,018	26,331	16,299	129 (111)	84 (77)	37 (30)	8 (4)	This study
*D. naviculare* var. *lasiocarpum* W. T. Wang (*)	OR263587	154,171	38.27	84,656	26,594	16,327	129 (111)	84 (77)	37 (30)	8 (4)	This study
*D. sauricum* Schischk. (*)	OR263590	154,255	38.25	84,765	26,594	16,302	129 (111)	84 (77)	37 (30)	8 (4)	This study
*D. shawurense* W. T. Wang (*)	OR263585	154,284	38.26	84,820	26,561	16,342	129 (111)	84 (77)	37 (30)	8 (4)	This study
*D. winklerianum* Huth (*)	OR263589	154,235	38.26	84,754	26,594	16,293	129 (111)	84 (77)	37 (30)	8 (4)	This study
*D. yunnanense* Franch.	MW246156	154,053	38.26	84,639	26,551	16,312	129 (111)	84 (77)	37 (30)	8 (4)	[36]

All the eight *Delphinium* plastomes contained the same set of 129 genes, including 84 genes encoding proteins, eight ribosomal RNAs (rRNAs) and 37 transfer RNAs (tRNAs) (Table 1; * showing the new chloroplast genomes reported in this study). Seventeen unique genes contained one (*atp*F, *ndh*A, *ndh*B, *pet*B, *pet*D, *rpl*16, *rpl*2, *rpo*C1, *trn*A^(TGC)^, *trn*G^(TCC)^, *trn*I^(GAT)^, *trn*K^(TTT)^, *trn*L^(TAA)^, *trn*V^(TAC)^) or two (*rps*12, *clp*P and *ycf*3) introns (Additional File 1: Table S1; Figs. 1 and 2). In addition, these genes could be divided into three categories according to their functions (Additional File 1: Table S1). The first type of function was mainly related to photosynthesis, with 44 unique genes; the second category of function was mainly related to cp. automatic transcription and translation, with 57 unique genes; the third category had 11 unique genes, mainly involved in other biosynthetic genes and open reading frames with unknown function.

### Boundaries of IR regions, repeat structure and SSR analysis of chloroplast genomes

**Fig. 3:**
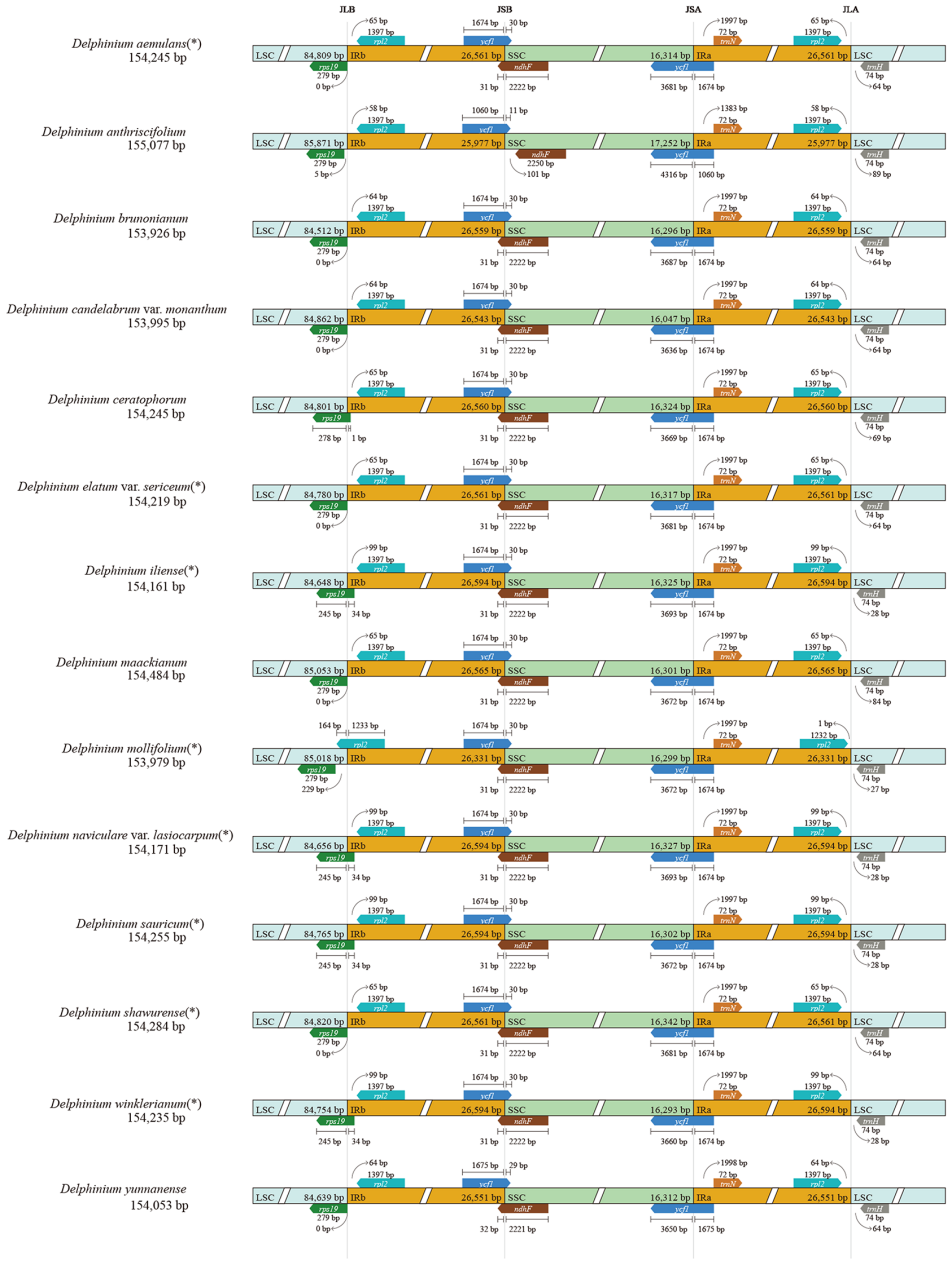
Comparison of LSC, inverted-repeats (IRs), and SSC junction positions among 14 *Delphinium* plastomes, * showing the new chloroplast genomes reported in this study

The potential expansions and contractions of IR borders was considered to be the main cause of cp. genome length changes and the evolutionary events in angiosperm, though relative conservation of IR/SC boundaries in plant plastomes [39, 40]. We compared the IR/SC boundaries together with the adjacent genes in the 14 *Delphinium* plastomes (including eight newly sequenced *Delphinium* cp. genomes; Fig. 3; * showing the new chloroplast genomes reported in this study). The IRa/SSC boundary was identified within *ycf*1 gene (with the 5′ end located in the IRa region while 3′ end located in the SSC region), with spanned 1060–1675 bp in the IRa region. Similarly, the IRb/SSC boundary was located within *ycf*1 gene (with the 5′ end located in the IRb region while 3′ end in the SSC) and *ndh*F gene (with the 5′ end located in the SSC region while 3′ end in the IRb), with the former expanded 29–30 bp in the SSC region and the latter expanded 31–32 bp in the IRb region. However, in the case of *D. anthriscifolium* Hance sample (MK253461), the IRb/SSC boundary only located within *ycf*1 gene, with an expansion length of 11 bp in the SSC region. The IRb/LSC boundary exhibited obviously varied. Four samples, including *D. ceratophorum* Franch., *D. iliense*, *D. naviculare* var. *lasiocarpum* and *D. winklerianum*, had the boundary located within the *rps*19 gene, with an expansion length of 1–34 bp. The remaining 11 samples were either 0–5 bp away from the IRb/LSC boundary, except for the *D. mollifolium* sample (OR263588), where the boundary was located in *rpl*2 gene (with the 5′ end located in the IRb region while 3′ end in the LSC) with an expansion length of 164 bp.

**Fig. 4:**
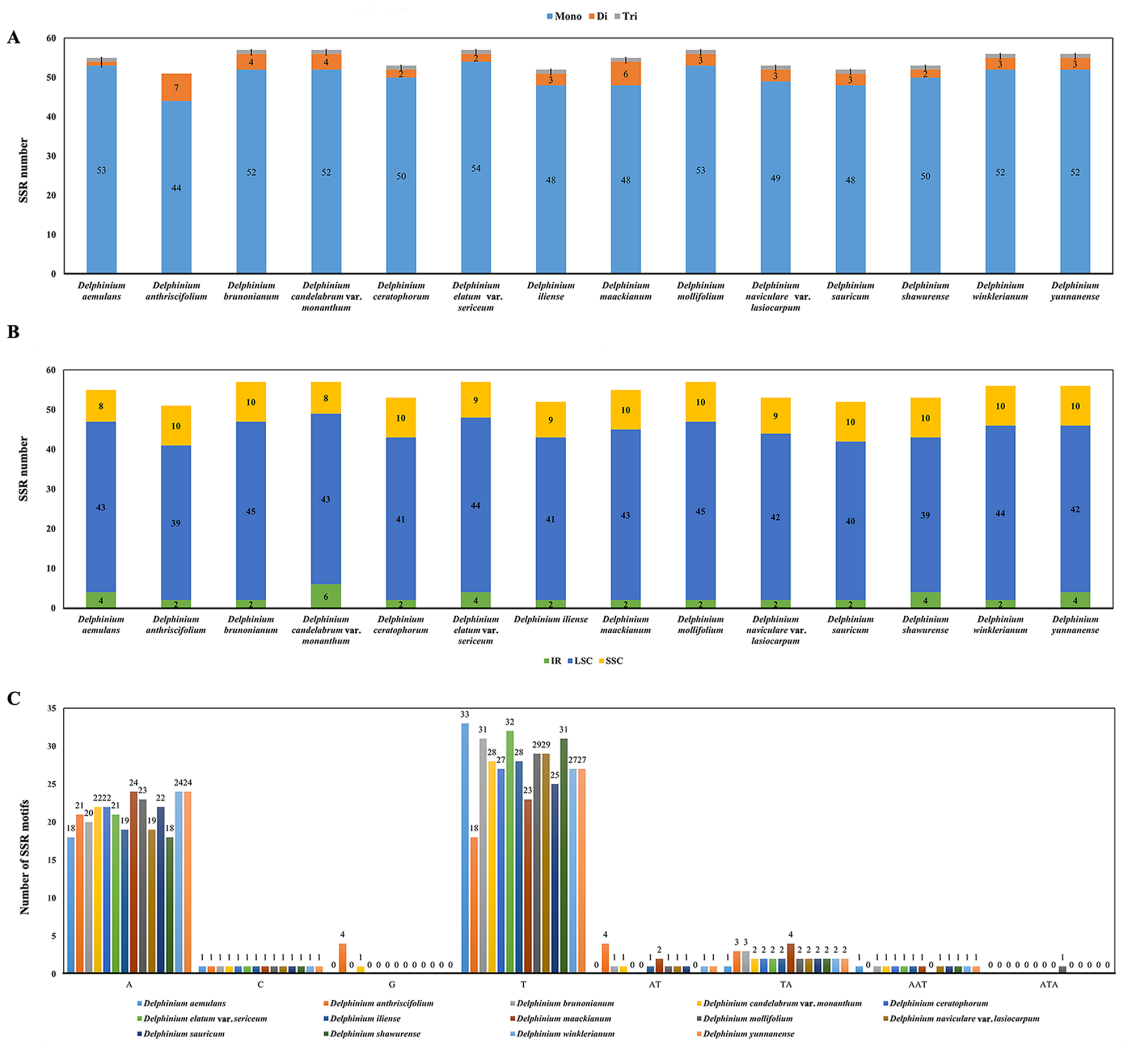
Statistics of SSRs in 14 *Delphinium* taxa samples. (**A**) Number of SSRs types. **(B)** Number of SSRs distributed in LSC, SSC and IR regions. **(C)** Distribution of different types and sizes of SSRs motifs in the plastid genomes

We detected six types of SSR (Additional File 2: Table S2; Fig. 4A) for each species in 14 *Delphinium* cp. genome, in which the number of total SSRs was from 51 (*D. anthriscifolium*) to 57 [*D. brunonianum* Royle, *D. candelabrum* var. *monanthum* (Hand.-Mazz.) W. T. Wang, *D. elatum* var. *sericeum* W. T. Wang, *D. mollifolium*]. Most cp. genome SSRs, with the proportion from 73.58% (*D. shawurense*) to 79.25% (*D. naviculare* var. *lasiocarpum*) out of the total number of SSRs, were distributed in the LSC regions. The SSRs distributed in the SSC region ranged from 14.55% (*D. aemulans* Nevski) to 19.61% (*D. anthriscifolium*) and in the IR regions varied from 3.51% (*D. mollifolium* and *D. winklerianum*) to 10.53% (*D. candelabrum* var. *monanthum*) (Fig. 4B). Among these SSRs, the mono-nucleotide A/C/G/T repeat units occupied the highest proportion with 86.27–96.36%, and the di-nucleotide repeats (AT/TA) and tri-nucleotide repeats (AAT/ATA) units accounted for 1.81–13.73% and 0–1.92% out of the total number of SSRs, respectively (Fig. 4C).

**Fig. 5:**
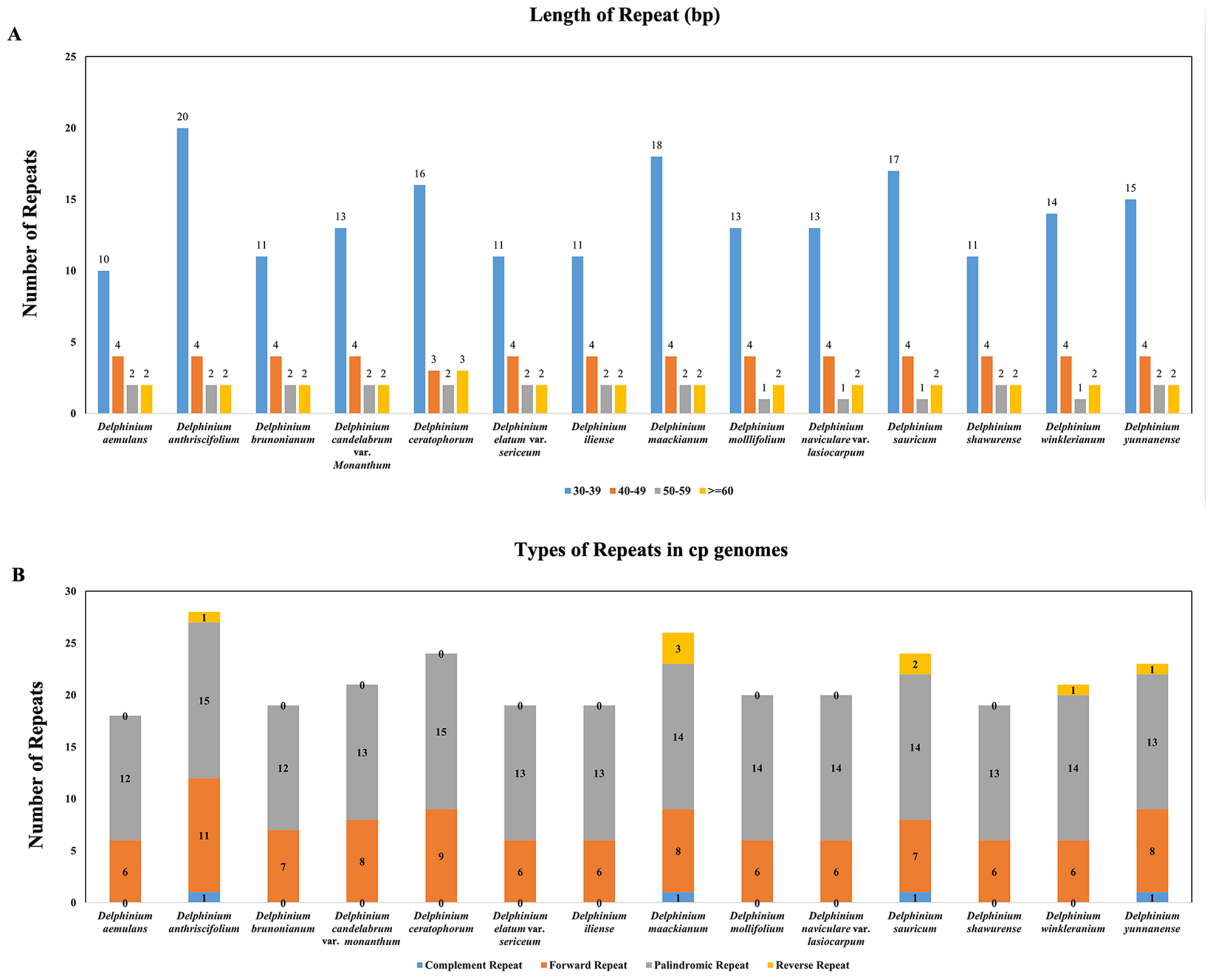
Statistics of repeats in 14 *Delphinium* taxa samples. **(A)** Number of different lengths of repeats. **(B)** Number of four types of repeats

Meanwhile, more than 30 bp of base repeats in all samples and distinct forms of these long repeats, including complementary, forward, palindromic and reversed, were also analyzed (Additional File 3: Table S3; Fig. 5). For these 14 *Delphinium* cp. genomes, the size of the top three most frequently long repeats were 30 bp, 31 bp and 42 bp. The distribution of repeats per genome, and length of repeat and number of such repeated sequences per species were shown in Fig. 5A, respectively. In each taxon, the number of long repeats ranged from 18 (*D. aemulans*) to 28 (*D. anthriscifolium*); and the number of complementary, forward, palindromic and reversed repeats were 0–1, 6–11, 12–15 and 0–3, respectively (Fig. 5B). Most long repeats were distributed in intergenic areas, and a few in shared genes or introns, such as *ycf*2 and *ycf*3-intron.

### Genome comparison, hypervariable regions

**Fig. 6:**
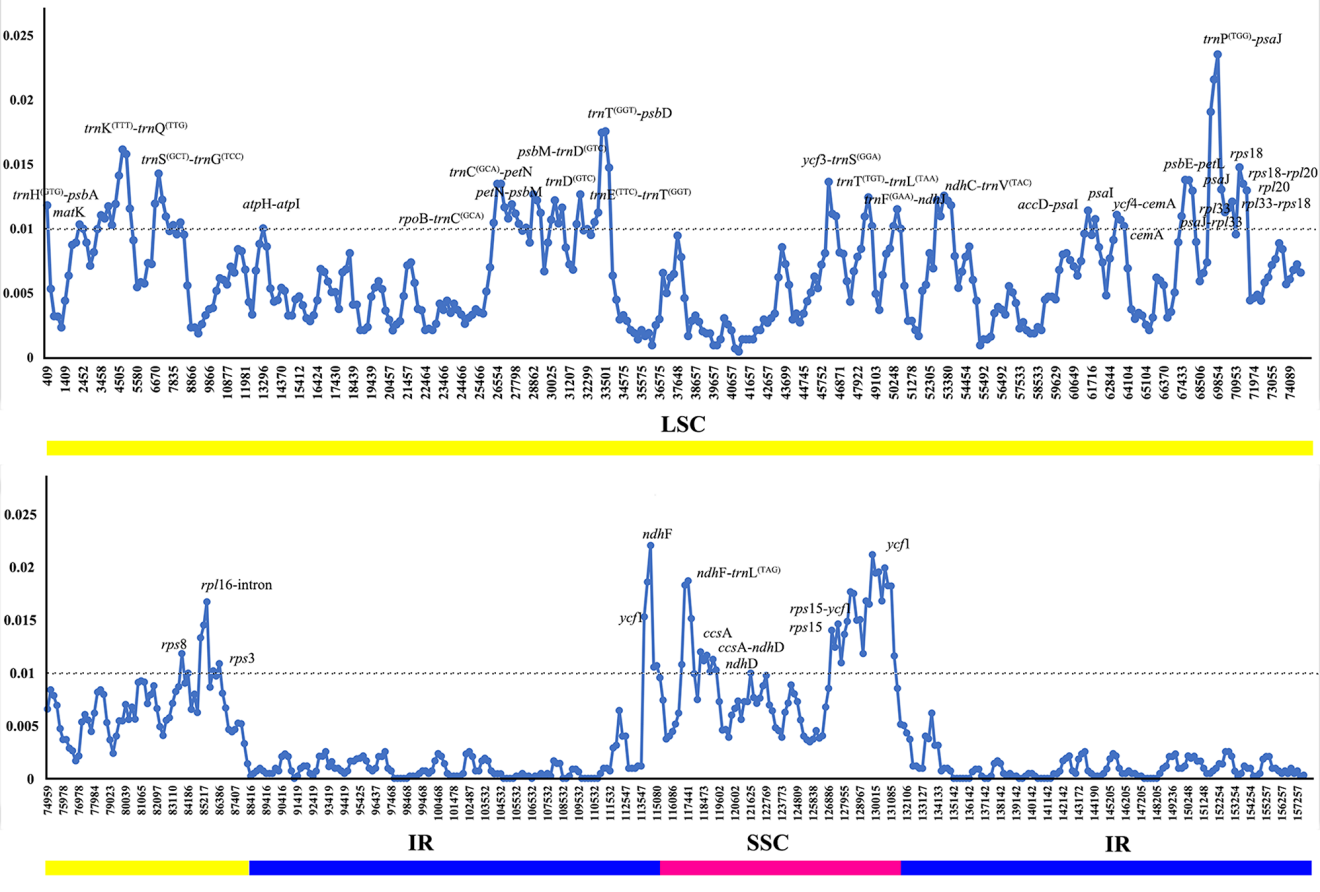
Comparison of nucleotide variability values (Pi) among 14 *Delphinium* chloroplast genomes. The x-axis indicates the position of the midpoint of a window, while the y-axis represents the nucleotide diversity of each window. The different colored lines at the bottom indicate the locations of these genes in various regions of the plastomes

The value of nucleotide variability (Pi) value among 14 *Delphinium* cp. genomes revealed that the intergenic spacer (IGS) regions were more variable than gene regions (Additional File 4: Table S4; Fig. 6). The SSC regions showed the highest average nucleotide diversity (Pi = 0.00998), followed by the LSC (Pi = 0.006619) and IR (Pi = 0.001231) regions. 32 hypervariable sites in LSC region with Pi ≥ 0.01 were screened (Fig. 6), namely *acc*D-*psa*I, *atp*H-*atp*I, *cem*A, *mat*K, *ndh*C-*trn*V^(TAC)^, *pet*N-*psb*M, *psa*I, *psa*J, *psa*J-*rpl*33, *psb*E-*pet*L, *psb*M-*trn*D^(GTC)^, *rpl*16-intron, *rpl*20, *rpl*33-*rps*18, *rpo*B-*trn*C^(GCA)^, *rps*3, *rps*8, *rps*18, *rps*18-*rpl*20, *trn*C^(GCA)^-*pet*N, *trn*D^(GTC)^, *trn*E^(TTC)^-*trn*T^(GGT)^, *trn*F^(GAA)^-*ndh*J, *trn*G^(TCC)^, *trn*K^(TTT)^-intron, *trn*K^(TTT)^-*trn*Q^(TTG)^, *trn*P^(TGG)^-*psa*J, *trn*S^(GCT)^-*trn*G^(TCC)^, *trn*T^(GGT)^-*psb*D, *trn*T^(TGT)^-*trn*L^(TAA)^, *ycf*3-*trn*S^(GGA)^, *ycf*4-*cem*A; and eight hypervariable sites with Pi ≥ 0.01 in SSC regions were also screened in Fig. 6, namely *ccs*A, *ccs*A-*ndh*D, *ndh*D, *ndh*F, *ndh*F-*trn*L^(TAG)^, *rps*15, *rps*15-*ycf*1and *ycf*1.

**Fig. 7:**
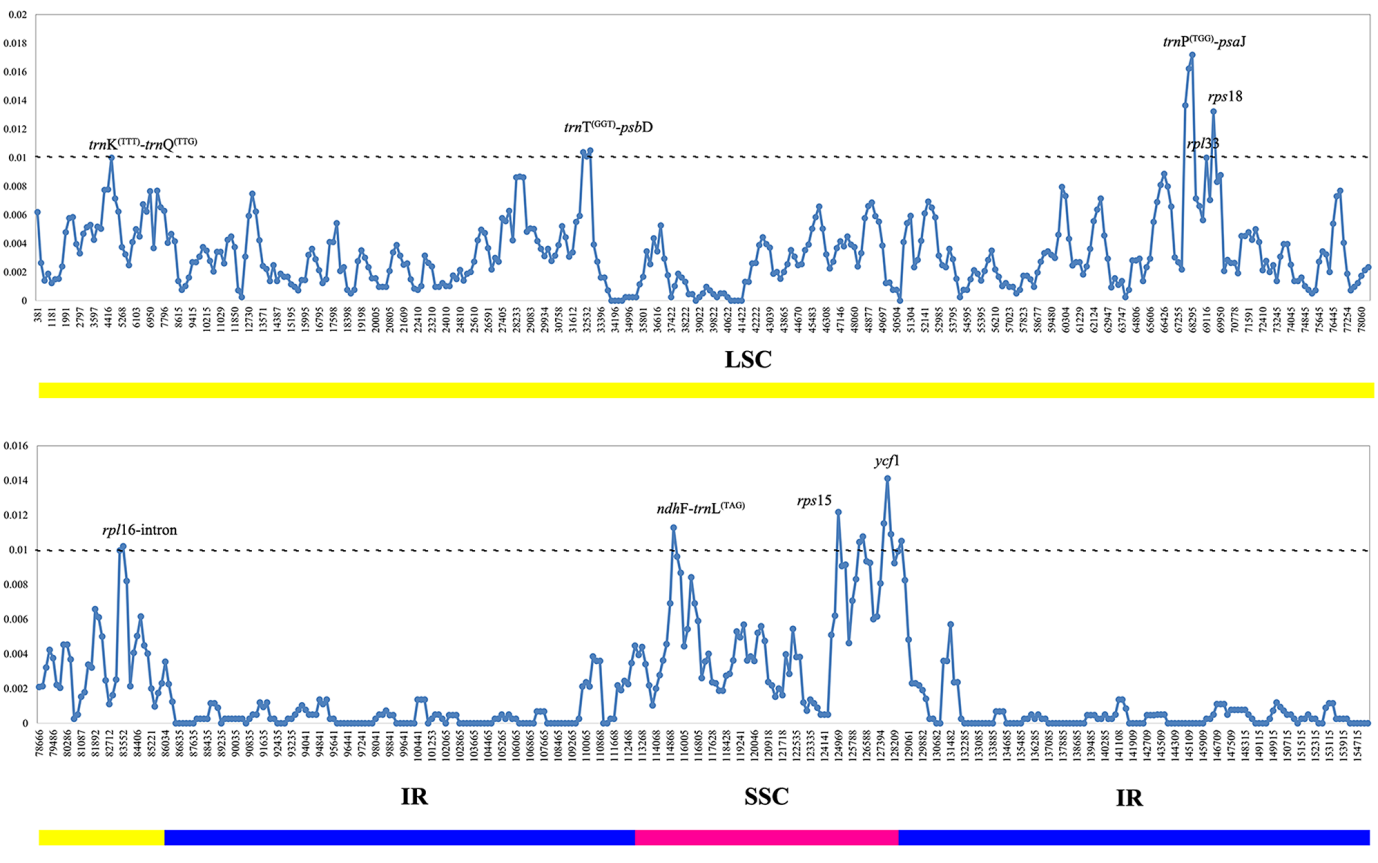
Comparison of nucleotide variability values (Pi) among 13 perennial *Delphinium* chloroplast genomes. The x-axis indicates the position of the midpoint of a window, while the y-axis represents the nucleotide diversity of each window. The different colored lines at the bottom indicate the locations of these genes in various regions of the plastomes

Meanwhile, considering the annual species, *Delphinium anthriscifolium*, was definitely different from perennial groups. The value of nucleotide variability (Pi) value among 13 perennial *Delphinium* cp. genomes was also conducted. The results showed that the IR regions were observed to have lower Pi value than LSC and SSC regions. The SSC regions showed the highest average nucleotide diversity (Pi = 0.005164), followed by the LSC (Pi = 0.003326) and IR (Pi = 0.000519) regions. Six hypervariable sites in LSC having Pi ≥ 0.01, were *rpl*16-intron, *rpl*33, *rps*18, *trn*K^(TTT)^-*trn*Q^(TTG)^, *trn*P^(TGG)^-*psa*J, *trn*T^(GGT)^-*psb*D; while three hypervariable sites in SSC regions with Pi ≥ 0.01, namely *ndh*F-*trn*L^(TAG)^, *rps*15 and *ycf*1. (Additional File 5: Table S5; Fig. 7).

**Fig. 8:**
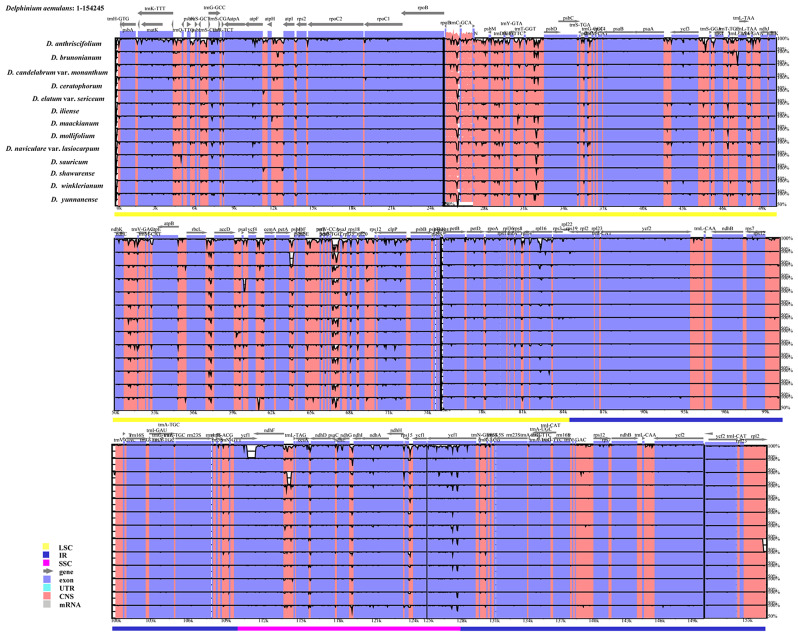
Sequence identity plot of 14 *Delphinium* species cp. genome sequences. Using *D. aemulans* sequence as a reference, grey arrows indicate the orientation of genes, red bars represent non-coding sequences, purple bars represent exons, and blue bars represent introns; vertical scale indicates the percentage identity within 50–100%. The different colored lines at the bottom indicate the locations of these genes in various regions of the plastomes

The mVISTA results showed that the non-coding regions were more variable than the coding regions, the LSC and SSC regions had higher levels of sequence divergence than the two IR regions, and the IGS regions were the most divergent regions (Fig. 8). The highly divergent regions among 14 chloroplast genomes occurred six in the IGS regions, four in the LSC regions, including *rbc*L-*acc*D, *rpo*B-*trn*C^(GCA)^, *trn*T^(GGT)^-*psb*D, *trn*P^(TGG)^-*psa*J, and two near the boundary between IRa and SSC region: *ccs*A-*ndh*D, *ndh*F-*trn*L^(TAG)^. Apart from these regions, one region *ycf*1 also showed high sequence variation (Fig. 8).

### Codon usage analysis

We detected the synonymous codon usage of 77 unique PCGs in the 14 *Delphinium* and calculated several related parameters, including the effective number codons (ENC), codon bias index (CBI) and relative synonymous codon usage (RSCU). The ENC and CBI of all these 77 unique PCGs varied a wide range, with the former ranging from 24.824 to 61 and the latter ranging from 0.268 to 0.853 (Additional File 6: Table S6). The results showed that these genes were expressed in different levels probably due to the frequency of optimal codons [41]. The PCGs contained a total of 22,525 to 22,556 codons in the 14 *Delphinium* plastomes, including stop codons. Leucine (Leu; 2328–2339) was the most abundant amino acid, while Cysteine (Cys; 252–263) showed the least abundance in the cp. genome of these taxa (Additional File 7: Table S7).

**Fig. 9:**
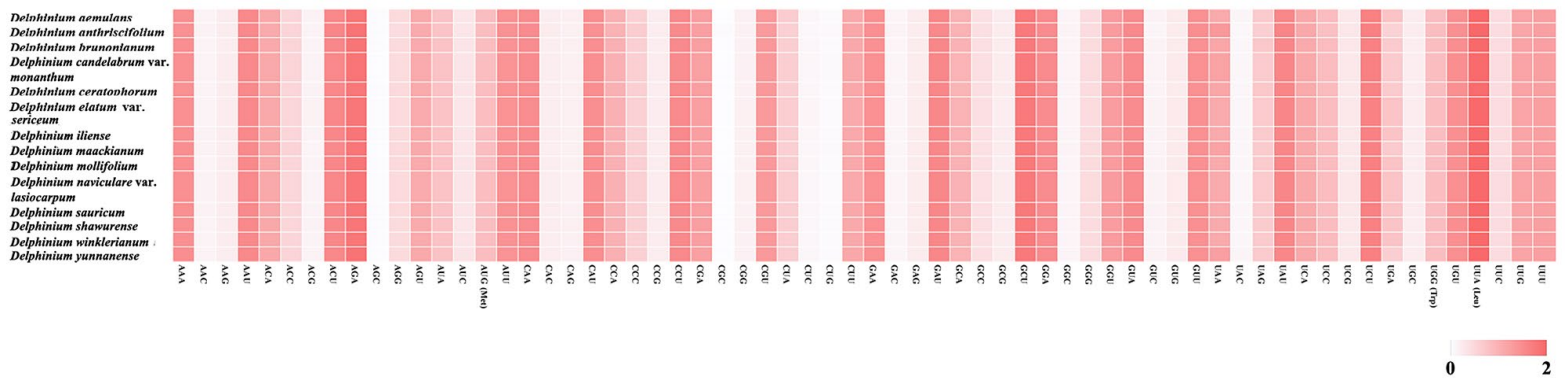
Relative synonymous codon usage (RSCU) values of all merged 77 protein‑coding genes for 14 *Delphinium* plastomes. Color key: red values indicate higher RSCU values, and white values indicate lower RSCU values. * indicates termination/stop codon; Met indicates methionine; Trp indicates tryptophan; Leu indicates Leucine

The RSCU value analysis showed that almost all amino acids were encoded by one to six synonymous codons, except methionine and tryptophan (Met and Trp; RSCU = 1). Almost Half of these codons (32/61; not including stop codon) had RSCU ≥ 1, in which most (29/32) ended with the base A or U. Meanwhile, about half of codons (29/61; not including stop codon) had RSCU < 1, in which majority (27/29) ended with the base C or G. All three stop codons were present, with UAA being the most frequently used among these 77 unique PCGs in the 14 *Delphinium* (Additional File 7: Table S7; Fig. 9).

### Selective pressure

The *ω* ratio (*d*_N_/*d*_S_) of 77 unique PCGs among these 16 species in Ranunculaceae were calculated to estimate the selective pressure. A total of six genes (*clp*P, *pet*N, *psb*J, *psb*Z, *rpl*23 and *ycf*1) in m0 model were found to be under positive selection (*ω* ratio > 1), while 35 genes (*atp*F, *atp*H, *ccs*A, *cem*A, *clp*P, *inf*A, *ndh*A, *ndh*E, *ndh*G, *ndh*H, *ndh*K, *pet*A, *pet*D, *pet*N, *psa*A, *psa*C, *psa*I, *psb*E, *psb*F, *psb*I, *psb*J, *psb*Z, *rpl*14, *rpl*20, *rpl*22, *rpl*23, *rps*3, *rps*4, *rps*7, *rps*8, *rps*15, *rps*19, *ycf*1, *ycf*2, *ycf*4) in m2 model were identified as being under positive selection. The value of *ω* ratio was significantly different (*P* < 0.05) among these taxa for two genes (*psa*A and *rpl*20) based on likelihood ratio tests (LRTs) (Additional File 8: Table S8).

### Phylogenetic analysis

**Fig. 10:**
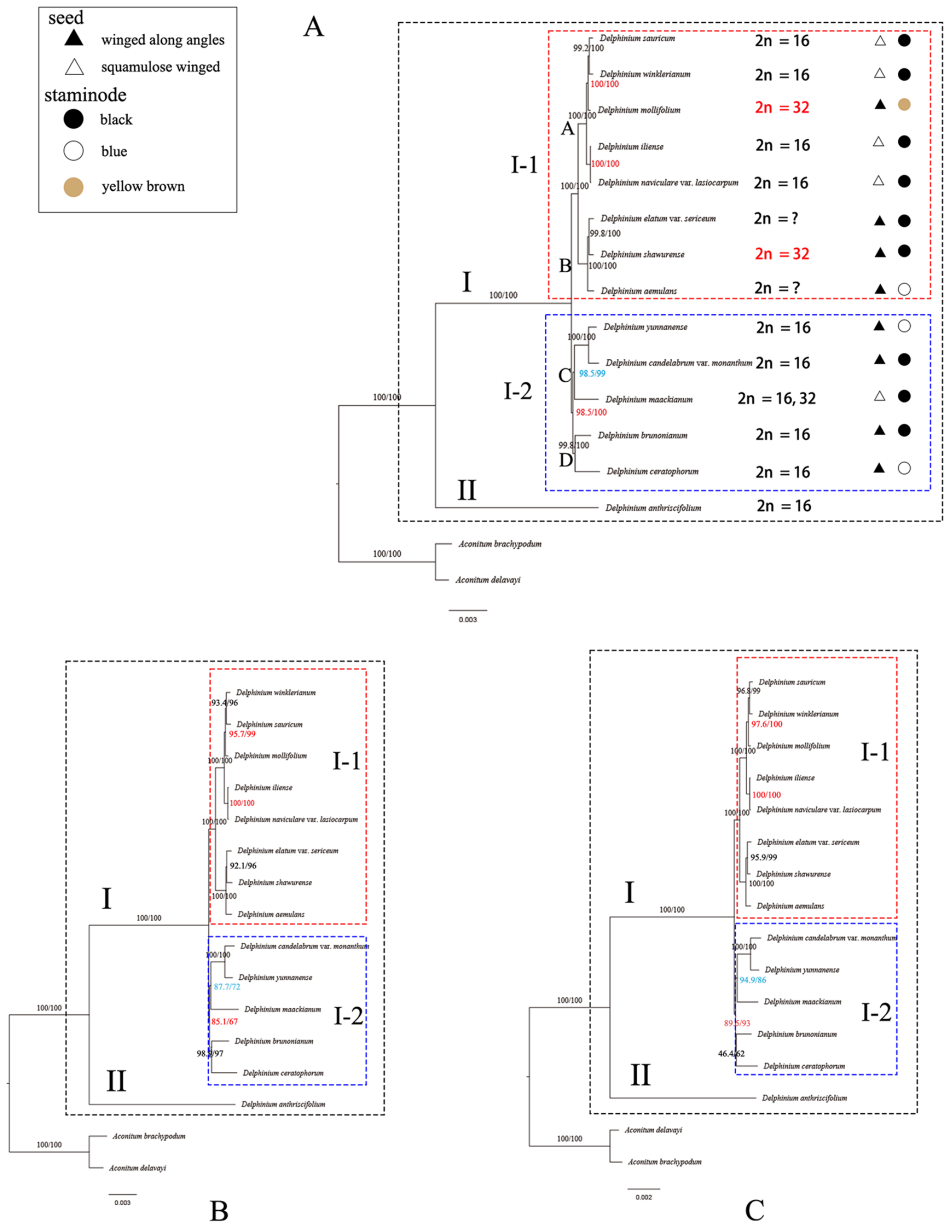
Phylogenetic trees based on complete cp. genomes **(A)**, concatenation of 132 unique IGS region **(B)**, and concatenation of 77 unique PCGs regions **(C)** resulting from the maximum likelihood (ML) analysis of 14 *Delphinium* samples and two *Aconitum* species as outgroups. The bootstrap support values in ML analysis are displayed at nodes

We used three datasets, including the whole complete plastid genome sequences, concatenation of 132 unique IGS regions, and concatenation of 77 unique PCGs regions to construct the phylogenetic relationships among the 14 *Delphinium* species, respectively, with *Aconitum brachypodum* Diels and *A. delavayi* Franch. as outgroups by using ML method. Despite minor discrepancies, the results of these three topologies were found in high congruence (Fig. 10).

**Fig. 11:**
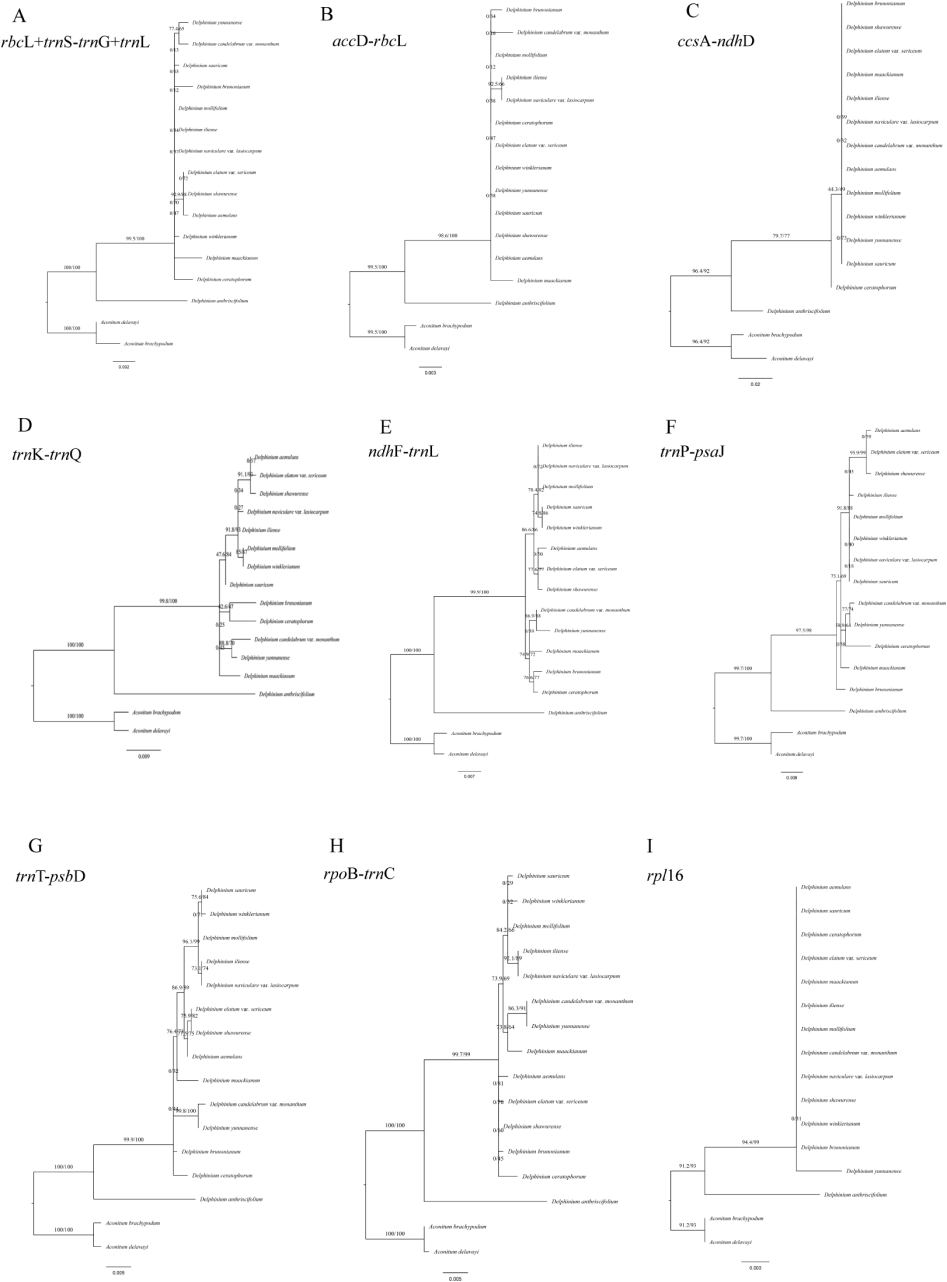
Phylogenetic trees among 14 *Delphinium* samples and two *Aconitum* species as references inferred from maximum likelihood (ML) analysis. **(A)** concatenation of *rbc*L, *trn*S^(TGA)^-*trn*G^(TCC)^ and *trn*L^(CAA)^. (**B**) *acc*D-*rbc*L. (**C**)* ccs*A-*ndh*D. (**D**) *trn*K^(TTT)^-*trn*Q^(TTG)^. (**E**) *ndh*F-*trn*L^(TAG)^. (**F**) *trn*P^(TGG)^-*psa*J. (**G**)* trn*T^(GGT)^-*psb*D. (**H**) *rpo*B-*trn*C^(GCA)^. (**I**) *rpl*16

Our results showed that the genus *Delphinium* was monophyletic with strong support and contained two major clades which were fully supported [Bootstrap Support (BS) = 100] as sister groups: clade I and II (Fig. 10). The clade I comprised all perennial *Delphinium* samples divided into two strongly supported subclades (I–1 and I–2). Subclade I–1 (BS = 100) contained eight *Delphinium* taxa all collected from Xinjiang, China. However, only slightly different supporting values were observed at nodes based on the different sequence datasets. For instance, the nodes (red showing in Fig. 10) in subclade I–1 derived from the dataset of the whole complete plastid genome showed stronger supports (BS = 100 and BS = 100; Fig. 10A) than those derived from the concatenation of 132 unique IGS regions (BS = 95.7 and BS = 100; Fig. 10B) and the concatenation of 77 unique PCGs (BS = 97.6 and BS = 100; Fig. 10C). Additionally, the supporting value (red showing in Fig. 10) of subclade I–2 derived from the whole complete plastid genome (BS = 98.5; Fig. 10A) and 77 unique PCGs (BS = 89.5; Fig. 10C) was stronger than it derived from the IGS regions (BS = 85.1; Fig. 10B), while the supporting values of one nodes (blue showing in Fig. 10) in subclade I–2 derived from the whole cp. genome (BS = 98.5; Fig. 10A) were stronger than it derived from the concatenation of 132 unique IGS regions (BS = 87.7; Fig. 10B) and concatenation of 77 unique PCGs regions (BS = 94.9; Fig. 10C). Besides, the resolution of previously used three sequence fragments, including *rbc*L, *trn*S^(TGA)^-*trn*G^(TCC)^ and *trn*L^(CAA)^ [29–31] also concatenated here was also evaluated for *Delphinium* species, which showed in poorly supporting value (Fig. 11A).

**Fig. 12:**
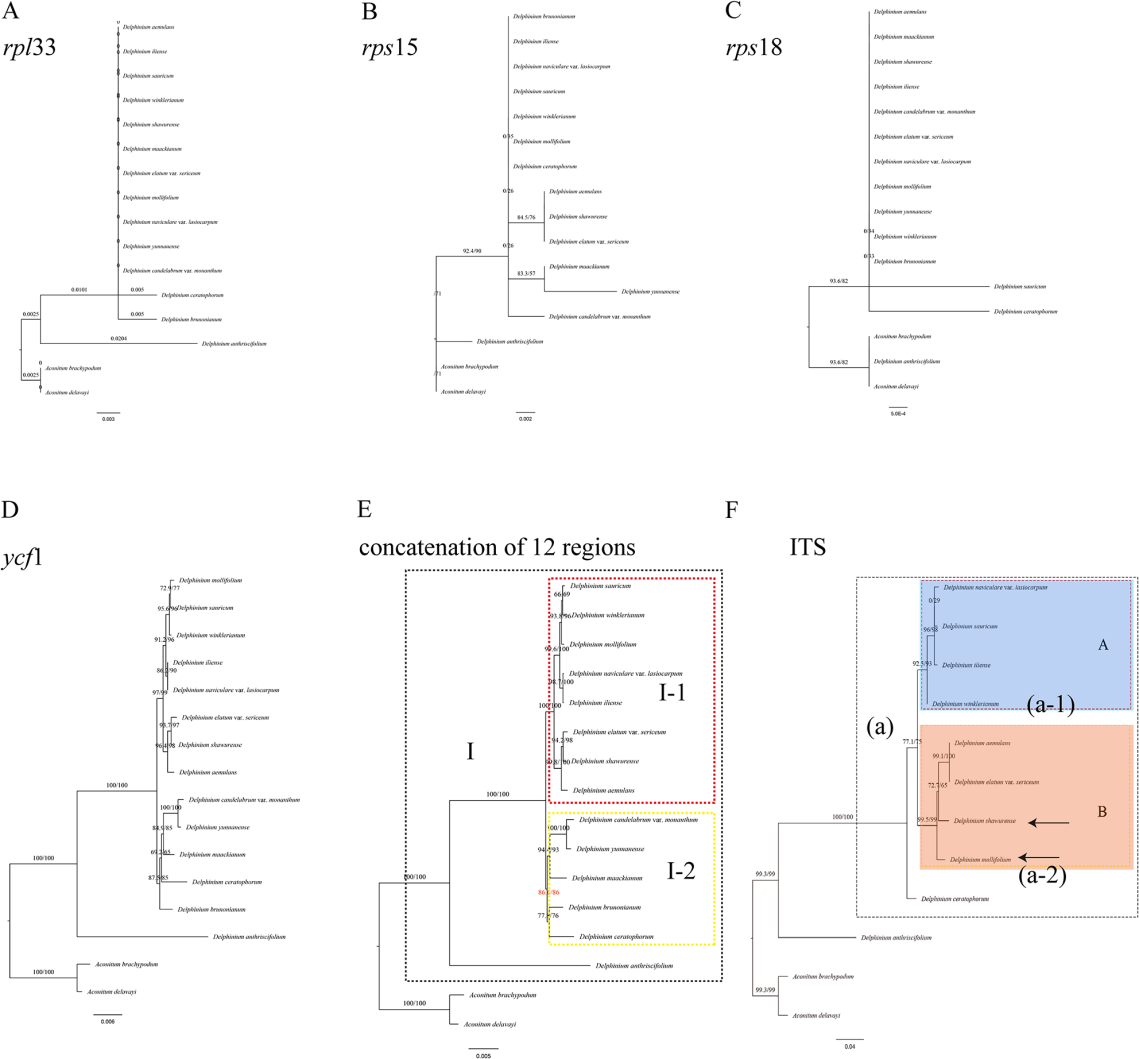
Phylogenetic trees based on *rpl*33 **(A)**, *rps*15 **(B)**, *rps*18 **(C)**, *ycf*1 **(D)** and concatenation of 12 regions **(E)** among 14 *Delphinium* samples and two *Aconitum* species as references; nrDNA ITS region **(F)** among 10 *Delphinium* samples and two *Aconitum* species as references inferred from maximum likelihood (ML) analysis. Note: the arrowed showing the tetraploid taxa, *D. mollifolium* and *D. shawurense*

A total of 12 hypervariable or high Pi value regions (*acc*D-*rbc*L, *ccs*A-*ndh*D, *ndh*F-*trn*L^(TAG)^, *rpo*B-*trn*C^(GCA)^, *rpl*16, *rpl*33, *rps*15, *rps*18, *trn*K^(TTT)^-*trn*Q^(TTG)^, *trn*P^(TGG)^-*psa*J, *trn*T^(GGT)^-*psb*D and *ycf*1) and concatenation of these 12 regions were also evaluated for phylogenetic analysis in our study (Figs. 11B–I and 12A–E). Moreover, the analysis of sequence alignments revealed the presence of parsimony-informative characters across multiple genes. Notably, *ycf*1 exhibited the highest number of parsimony-informative characters, indicating a rich phylogenetic signal. Conversely, *rpl*33 displayed a lower number of informative characters, suggesting a potential lack of phylogenetic resolution in this region. Detailed results for each gene are provided in Supplementary Table S9 (Additional File 9). However, compared to the three topological trees constructed by the whole cp. genome (Fig. 10A), two fragment sequences topological trees (Figs. 11D and E and 12D) based on *ndh*F*-trn*L^(TAG)^ and *ycf*1 performed well in dividing the perennial *Delphinium* into two groups as the whole cp. genome. In addition, the concatenation of 12 hypervariable or high Pi value regions (Fig. 12E) yielded highly similar topological results to the whole cp. genome, with different supporting values. For example, the nodes in clade I–2 derived from the dataset of a concatenation of 12 regions showed strong supports (BS = 86.6; red showing in Fig. 12E) lower than those from the whole cp. genome (BS = 98.5; red showing in Fig. 10A), concatenation of 77 unique PCGs regions (BS = 89.5; red showing in Fig. 10C).

Furthermore, the relationship was also reconstructed by using ML analysis based on nrDNA ITS herein among nine *Delphinium* and two *Aconitum* taxa (Fig. 12F). The nrITS topology was similar to the topologies inferred from the plastid genome sequences with different placement in some species. For instance, in contrast to the tree derived from plastid genome sequences, in nrITS tree, *D. aemulans* and *D. elatum* var. *sericeum* formed a strongly supported clade sister to *D. shawurense* with strongly supported (BS = 72.7; Fig. 12F) and nested within one branch with *D. mollifolium* in clade a**–**2.

## Discussion

### Plastome features in *Delphinium*

In this study, the cp. genomic structure, gene order and GC content among eight newly sequenced *Delphinium* taxa were highly conserved and nearly similar, which were also identical to other cp. genomes in angiosperms [42]. The size of the 14 *Delphinium* plastomes varied from 153,926 bp (*D. brunonianum*; NC_051554) to 155,077 bp (*D. anthriscifolium*; MK253461) (Table 1). The *Delphinium* cp. genome sequenced here, all contained a total of 129 genes (including 111 unique genes), with the total GC content ranging from 38.13% to 38.30% (Table 1). However, compared with the previously published plastomes of other seven taxa in *Delphinium* [35–37], some taxa were found to contain different numbers of genes in different samples, for instance, *D. anthriscifolium* (MK253461), *D. brunonianum* (NC_051554), *D. candelabrum* var. *monanthum* (MW246165), *D. ceratophorum* (MK253460), *D. maackianum* Regel (NC_047293) and *D. yunnanense* (MW246156) were reported contained 128, 131, 130, 128, 129 and 130 genes, respectively, whereas all annotated here contained only 129 genes. To eliminate the influences of references and annotation software used, the 14 samples were re-annotated using Plastid Genome Annotator (PGA) and Geneious Prime 2023.01.1, with *Nicotiana tabacum* L. (NC001879) and *Aconitum brachypodum* (MT584424) as the reference genome. Additionally, the tRNA genes were verified by tRNA-SE and ARAGORN. Unexpectedly, upon examining all the 14 sequences re-annotated, we found that only 129 genes and no gene loss were observed in this study (Table S1). Moreover, it should be noted that the plastome of *D. grandiflorum* (NC_049872) previously reported, which had been associated with ambiguous or incorrect information and potential misidentifications, were not included in our analysis.

The contraction and expansion of IR/SC boundary regions were usually considered as an important process involved in cp. genome variation within Ranunculaceae [35] and other angiosperm plastomes [43]. Furthermore, this phenomenon had proven to be particularly informative for evolutionary studies within specific groups [44]. However, minor variations were observed in the IR/SC boundary regions with no significant expansion or contractions among the 14 *Delphinium* plastomes (Fig. 3). The length of the IR region ranged from 25,977 bp to 26,594 bp. Only the *rpl*2 gene with an expansion length of 164 bp for *D. mollifolium* expanded to the LSC region; the remaining 13 *Delphinium* samples were entirely located within the IRb region. And the *ndh*F gene with contraction length of 101 bp away from the IRb region only in *D. anthriscifolium* (MK253461). These results were also similar to the contraction or expansion event in the cp. genome of other genera in Ranunculaceae, such as *Gymnaconitum* (Stapf) Wei Wang & Z. D. Chen, *Nigella* L., *Beesia* Balf. f. & W. W. Sm., *Actaea* L., *Souliea* Franch. and *Ranunculus* L [35, 45, 46].

Repeats and SSRs were widely analyzed in plant plastomes [47]. The variations of SSRs in cp. genomes were widely used to analysis the genome structure and diversity in population or species levels [48, 49]. Our findings indicated that mono-nucleotide repeats were the predominant type of repeat in the cp. genomes (Fig. 4), consistent with previous studies conducted in angiosperm cp. genomes [45, 50]. Among various types of SSRs, mono-nucleotide SSRs composed of A/T repeats exhibited higher abundance in the cp. genome. This observation aligned with prior reports suggesting that the prevalence of A/T repeats might be attributed to the relatively easier conversion of A/T compared to C/G in the plant cp. genome [51]. SSRs had also demonstrated their values in assessing genetic diversity within and between populations, as well as in studying the parentage of hybrid individuals in specific groups [52, 53]. Repeats variation in type, number, and location in different taxa, which were used to identify mutational hotspots and phylogenetic relationships [46, 54]. Four types of repeats (complement repeats, forward repeats, palindromic repeats and reverse repeats) were detected, among which palindromic repeats were the most common type of repeats (Fig. 5). Moreover, the number and variety of repeats in *D. anthriscifolium*, *D. maackianum*, *D. sauricum*, and *D. yunnanense* were found to exceed those present in other taxa within the genus *Delphinium*. To determine whether these repeats and SSRs could be effectively employed for phylogenetic analysis of the *Delphinium* genus, additional investigations will be required in the future.

### Potential molecular markers in *Delphinium*

The plastid genomes exhibited a high degree of conservation in terms of genetic replication mechanisms and uniparental inheritance, while displaying relatively high levels of genetic variation due to low selective pressure [55]. Consequently, the cp. genome has proven successful in resolving relationships within the Ranunculaceae family [41, 45, 46] and other angiosperms [50, 56]. Our phylogenetic analysis, based on complete cp. genomes, as well as the concatenation of 132 unique IGS regions and the concatenation of 77 unique PCGs (Fig. 10), consistently supported a well-defined clade (clade I) for perennial *Delphinium*, aligning with previous studies [9, 29–31]. Notably, the phylogenetic trees based on complete plastid genomes (Fig. 10A) exhibited stronger support compared to other concatenation sequences (Fig. 10B, C). However, when using three fragments of *rbc*L, *trn*S^(TGA)^-*trn*G^(TCC)^, and *trn*L^(CAA)^ as done in previous studies [9, 29, 30], our results indicated low resolution among the 14 *Delphinium* taxa (Fig. 11A).

Furthermore, our findings revealed that non-coding regions exhibited higher divergence compared to coding regions, consistent with patterns observed in numerous angiosperms [57]. Variable chloroplast sequences were widely used for phylogenetic analysis and taxonomic identification [58]. Therefore, we constructed 13 maximum likelihood (ML) trees using a total of 12 hypervariable or high Pi value regions and their concatenation (Figs. 11B–I and 12A–E). These trees demonstrated that only two fragment sequences (Figs. 11D and E and 12D) having higher number of parsimony informative characters, namely *ndh*F-*trn*L^(TAG)^ and *ycf*1, performed well in differentiating perennial *Delphinium* into two groups, similar to the whole cp. genome, except for the highly consistent concatenation topology (Fig. 12E). It should be noted, however, that the low resolution in the ML tree based on *rpl*16 (Fig. 11I) and *rpl*33 (Fig. 12A) may be attributed to the loss of the *rpl*32 gene in the tribe Delphinieae [37, 59], which leads to compensatory changes in the plastid-encoded *rpl* subunits, rendering them unreliable markers for phylogeny in *Delphinium*. Thus, the highly variable sequences generated in this study, especially *ndh*F-*trn*L^(TAG)^ and *ycf*1, represented promising potential molecular markers for phylogeny reconstruction and DNA barcoding identification in *Delphinium* plants.

### Positive selection among genes

It is noted that all genes are basically subjected to a certain degree for natural selection, and the highly expressed genes might be selected by the evolutionary forces [41]. Codon usage analysis played a crucial role in unravelling the evolutionary process, genome structure and selection pressure on genes [60]. In this study, the remarkable similarities observed in RSCU among 14 *Delphinium* taxa strongly suggested the presence of common environmental factors influencing their evolutionary trajectory. Additionally, a notable bias towards a lower frequency in base C or G at the third codon position, as compared to A or U, was observed (Fig. 9). These findings were consistent with previous investigations conducted on cp. genomes of other angiosperm [51, 61], lending further support to our conclusions. The degeneracy of genetic code enabled the expression of genetic variation within a gene, resulting in the production of diverse proteins across different species [62]. Meanwhile, we conducted an analysis of different codon usage frequencies on 77 unique PCGs across 16 taxa under positive pressure (*ω* ratio > 1). The results revealed an upper limit of *ω* ratio = 999, indicating a lack of synonymous substitutions along the concerned branch. This phenomenon, occurring for various reasons, requires further investigation in the future [63, 64]. However, the likelihood ratio test (LRT) value remained valid in our analysis. Our results indicated that only two genes were subject to significantly strong positive selective pressure. Specifically, one plastid gene associated with photosystem I (*psa*A) exhibited significantly strong positive selective pressure (*P* < 0.005 based on likelihood ratio tests) across two branches, with a relatively lower Codon Bias Index (CBI) value (< CBI median = 0.505). Additionally, another plastid gene related to ribosomal protein (*rpl*20) also showed significantly strong positive selective pressure (*P* < 0.005 based on likelihood ratio tests) across three *Delphinium* species, displaying a higher CBI (> CBI median = 0.505). The differential codon usage bias observed in these genes suggested varying frequencies of rare and optimal codons, potentially influencing their expression patterns and functional evolution. Furthermore, the differences between selective pressure and codon usage frequencies among these plastid genes implied potential functional divergence among the 14 *Delphinium* taxa.

### Phylogenetic relationships

In our study, we conducted a phylogenetic analysis of Chinese *Delphinium* using the entire plastid genome (Fig. 10A) and nrDNA ITS (Fig. 12F) to investigate the monophyly, infrageneric classification and assess their status. Previous research on the systematics of Chinese *Delphinium* had primarily relied on several molecular fragments [9, 29, 30] and morphological investigations [6, 8]. However, our phylogenetic trees showed inconsistencies with all the previously reported molecular phylogenetic studies. For example, Zuo [9] found that *D. elatum* var. *sericeum* (endemic to Xinjiang) formed a clade with other Xinjiang species in the cpDNA tree but was segregated in the nuclear gene tree. In contrast, our results demonstrated that the samples from Xinjiang, China, including *D. elatum* var. *sericeum*, formed a well-supported clade (clade I–1) in the plastid genome tree (Fig. 10A) and a well-supported clade (clade a) in the nrDNA ITS tree (Fig. 12F).

Analyzing the plastid topology (Fig. 10A), we observed that clade I comprised all perennial *Delphinium* taxa, while clade I–1 exclusively included samples collected from Xinjiang, China. Clade I–1 further divided into two well-supported clades, clade A and clade B. Interestingly, the status of taxa in clade A and clade B were mostly consistent with the morphology-based system based on seed morphology with a slight difference [8]. For instance, within clade A, *D. mollifolium* exhibited brown staminodes and winged seeds along angles, deviating from other taxa characterized by black staminodes and squamulose winged seeds. Similarly, in clade B, while all taxa displayed winged seeds along angles, *D. aemulans* stood out with blue staminodes as opposed to the predominant black staminodes observed in other taxa. These observations echo the findings of Zuo [9], highlighting occasional inconsistencies between molecular and morphology-based taxonomic systems. Furthermore, our analysis of the nrDNA ITS tree (Fig. 12F) revealed that all Xinjiang samples clustered within clade a, which further bifurcated into two well-supported clades, clade a–1 and clade a–2. Intriguingly, distinct seed morphology traits delineated the taxa within these clades, with clade a–1 exhibiting squamulose winging and clade a–2 displaying winging along angles. Unlike the chloroplast-based phylogenetic tree proposed by Zuo [9], which supported the morphology-based classification system primarily based on seed morphology, our nrDNA ITS tree demonstrated greater consistency with this classification scheme. Moreover, our study underscores the importance of incorporating additional samples and molecular fragments from nuclear ribosomal DNA for more comprehensive taxonomic investigations in the future.

Additionally, previous cytology research [9, 13, 65–69] reported that *D. mollifolium* and *D. shawurense* were tetraploid with a chromosome number of 2*n* = 32, while all other taxa were diploid (2*n* = 16), except for *D. aemulans* and *D. elatum* var. *sericeum*, for which chromosome number remained unknown (Additional File 10, 11: Table S10; Fig. S1). Morphologically, the staminode color in *D. mollifolium* was yellow brown, representing an intermediate character between the two crucial colors of black and blue (Fig. 10A). Moreover, the placement of *D. mollifolium* exhibited discrepancies between the plastid topology and nrDNA tree. Given the occurrence of hybridization and chloroplast capture events in Ranunculaceae, as noted by many authors [9, 70, 71], resolving the conflicting status of *D. mollifolium* would necessitate the incorporation of more nrDNA markers and samples.

Moreover, Wang and Yang [14] reported that Xinjiang province gathered the numerous basal taxa of evolutionary branches in Chinese *Delphinium* as well as taxa from Central Asia. They [14] also suggested that this area clearly represented the densest population of basal *Delphinium* species in China and even Central Asia. It encompassed both evolutionary early branching lineages and relatively basal components, along with a few more evolutionarily late branching lineages, representing low-level, middle-level, and high-level species in the phylogeny of *Delphinium*. Therefore, to accurately ascertain the status of clade I–1 and the significance of the Xinjiang groups in the context of *Delphinium* species, it was crucial to include plastomes from additional Chinese *Delphinium* samples or Central Asia.

Continuing with the analysis of the plastid topology (Fig. 10A), the samples within clade I–2 were divided into two well-supported clades: clade C and clade D. In clade C, *D. yunnanense* and *D. candelabrum* var. *monanthum*, characterized with seed winging along angles, different staminode colors, and *D. maackianum*, which with two types of chromosome numbers, characterized with squamulose winged seeds, and black staminodes, formed a fully supported clade. In clade D, *D. brunonianum* and *D. ceratophorum*, with winged seeds along angles and different staminode colors, clustered together in a well-supported clade.

As a result, excluding *D. mollifolium* and *D. maackianum*, two species with abnormal chromosome numbers, the perennial *Delphinium* (clade I) exhibited greater consistency with the morphology-based system that utilized seed morphology [8]. To further investigate the relationships among these species, especially *D. mollifolium* and *D. maackianum*, which might involve hybridization or polyploidization, future studies should increase the sample size and incorporate additional nrDNA markers. By expanding the sample size and utilizing more molecular markers, we will better understand the genetic relationships within these species. This approach will enable a comprehensive analysis of potential hybridization events or chromosomal changes, providing insights into the underlying mechanisms influencing the observed characteristics in *D. mollifolium* and *D. maackianum*.

Due to the high morphological variability, particularly in staminode color and seed morphology, two important but uncorrelated characters, taxonomic inconsistencies persist in the delimitation of taxa within the genus *Delphinium* [7, 8, 27]. For example, based on our observations of living plants in the field and examination of herbarium specimens, including type material, we previously demonstrated that *D. iliense* exhibited high variability in the indumentum of peduncles, pedicels, bracteoles, sepals, and carpels, as well as in the shape and position of bracteoles on pedicels. Consequently, we redefined this species and synonymized two names, including *D. iliense* var. *angustatum* Huth and *D. naviculare* var. *naviculare*, with it. However, we found that *D. naviculare* var. *lasiocarpum*, much like *D. iliense*, displayed high morphological variability. Although Borodina-Grabovskaya [72] synonymizing *D. naviculare* var. *lasiocarpum* with *D. naviculare* var. *naviculare* (a synonym of *D. iliense*), our plastid tree placed these two taxa together in a fully supported clade (Fig. 10A), while they revealed less proximity in the nrDNA tree (Fig. 12F). Moreover, through examination of herbarium specimens and living plants, we distinguished them based on the indumentum of stems, peduncles, and pedicels. A detailed investigation of the identity of *D. naviculare* var. *lasiocarpum* will be presented separately.

## Conclusion

This study represents the first comprehensive analysis of plastomic variations among *Delphinium* taxa, based on the examination of 14 complete plastomes. The chloroplast genome structure of *Delphinium* is similar to other angiosperms and possesses the typical quadripartite structure with the conserved genome arrangement and gene features. However, their size varies owing to the expansion/contraction of IR/SC boundaries. The variation of non-coding regions is larger than coding regions of the chloroplast genome. DNA sequence divergence across *Delphinium* plastomes and phylogenomic analyses reveal that *ndh*F-*trn*L^(TAG)^ and *ycf*1 are promising molecular markers. Therefore, these highly variable loci should be valuable for future phylogenetic and phylogeographic studies on *Delphinium*. Our phylogenomic analyses based on the whole plastomes, concatenation of 132 unique IGS regions, concatenation of 77 unique PCGs sequences and nrDNA ITS sequence, all support the monophyly of *Delphinium* and perennial taxa clusters together into one clade within this genus. These results will provide important data for systematic, phylogenomic and evolutionary research in the genus for future studies.

## Materials and methods

### Sampling, DNA extraction, chloroplast genome sequencing, assembling, and annotation

Plant materials of the eight *Delphinium* taxa were collected in the field during 2022 from Xinjiang Province in China. Fresh leaves were sampled and dried in silica gel immediately. Voucher specimens were deposited in the herbarium of Institute of Botany, Jiangsu Province and Chinese Academy of Sciences (NAS) and collection information were listed in the Additional File 12: Table S11. In addition, six complete chloroplast genomes of *Delphinium* species (Table 1) and two of *Aconitum* species (Table 1) that publicly available in NCBI GenBank were downloaded with annotations. Total genomic DNA was following a modified cetyltrimethylammonium bromide CTAB method [73]. DNA integrity was examined by electrophoresis in 1% (w/v) agarose gel, and concentration was measured by Qubit® DNA Assay Kit in Qubit® 3.0 Flurometer (Invitrogen, USA).

High-quality DNA libraries were constructed by shearing the genomic DNA into short fragments with approximately 350 bp before sequenced on Illumina platform and generated 150 bp paired-end reads at Novogene Bioinformatics Technology Co., Ltd. (Tianjin, China). Genomes assembly were performed using the GetOrganelle pipeline [74–76] based on the sequenced clean data. Bandage v.5.6.0 [77] was used to visualize and manually correct the assembly results. The annotation of the chloroplast genomes was performed in PGA [78]. Further annotation confirmation was compared with four sequences in the same tribe Delphinieae, *Aconitum brachypodum* (MT584424), *A. delavayi* (OM289058), *Delphinium anthriscifolium* (MK253461), *D. ceratophorum* (MK253460). Manual correction of start/stop codons and intron/exon boundaries was performed in Geneious Prime 2023.0.1 [79]. All transfer RNA (tRNA) genes were proofread with the web server tRNAscan-SE 2.0 (http://lowelab.ucsc.edu/tRNAscan-SE/) [80] and ARAGORN 1.2.38 (http://www.trna.se/ARAGORN/) [81]. All genome maps were drawn by Organellar Genome DRAW (OGDRAW) (http://ogdraw.mpimp-golm.mpg.de/) [82]. The complete cp. genome sequences and gene annotation of the eight newly assembled *Delphinium* taxa samples were deposited in GenBank (Table S1). Meanwhile, all the six cp. genomes in *Delphinium* reported previously were re-annotated.

### Genome comparison, codon usage analyses, plastid genomic variations and sequences repeat analysis

Using MAFFT v7.490 [83] to align the total 14 cp. genomes sequences (Table S1) for examining the divergence regions among *Delphinium* species. The aligned sequences were performed in Shuffle-LAGAN model via mVISTA program (http://genome.lbl.gov/vista/mvista/submit.shtml) with the annotated cp. genome sequence of *D. aemulans* (GenBank accession no. OR263583) as a reference genome. DnaSP v6 [84] was applied to examine the sequence divergence hotspots with conducting a sliding window analysis to calculate pi values among the cp. genomes, with windows size of 600 bp and step size of 200 bp.

IRscope software was used for the 14 *Delphinium* cp. genome sequences to visualize their IR/SC boundaries. CodonW [85] was used to analyze codon usage bias for all PCGs in the *Delphinium* plastome. Parameters such as ENC, CBI, and RSCU were calculated. ENC and CBI evaluated codon bias at the gene level, while RSCU observed and expected codon frequencies [86, 87]. Amino acid (AA) frequency was determined as the percentage of codons encoding the same AA out of the total codons. The program DnaSP v6 [84] was used for examination and complementary analysis of the codon usage bias results obtained from CodonW software [855].

SSRs were identified by Web-based simple sequence repeats finder MISA-web (https://webblastipk-gatersleben.de/misa/.), with minimum numbers of 10 repeat units for mono-, 6 repeat units for di-, 5 repeat units for tri-, tetra-, penta-, and hexa-nucleotide SSRs. The maximum length of a sequence between two SSRs was set as 10. REPuter was implemented to detect the short dispersed repeats [88], including forward, reverse, complement and palindromic, with the following parameters: a maximal repeat size of 5000, a minimal repeat size of 30, and hamming distance of 3.

### Phylogenetic analysis

A total of 16 complete cp. genome sequences were used for phylogenetic analysis, including eight newly and six previous reported *Delphinium* taxa, as well as *Aconitum brachypodum* and *A. delavayi* in Ranunculaceae as outgroups [20, 37] in this study (Table 1). Phylogenetic analyses were performed using ML method in the IQ-tree program [89] with auto substitution model and 1000 bootstrap replicates for evaluating the node support. FigTree v1.4 (http://tree.bio.ed.ac.uk/software/figtree/) was used to visualize the resulting trees. The analyses were carried out based on the following 18 datasets, including the complete plastid DNA, concatenation of 132 unique IGS regions, concatenation of 77 unique PCGs, the concatenation of two IGS regions and one gene (including *rbc*L, *trn*S^(TGA)^*-trn*G^(TCC)^ and *trn*L^(CAA)^) that previously studied in the tribe Delphinieae [29–31], 12 high pi value or hypervariable regions (*acc*D-*rbc*L, *ccs*A-*ndh*D, *ndh*F-*trn*L^(TAG)^, *rpo*B-*trn*C, *rpl*16, *rpl*33, *rps*15, *rps*18, *trn*K^(TTT)^-*trn*Q^(TTG)^, *trn*P^(TGG)^-*psa*J, *trn*T^(GGT)^*-psb*D and *ycf*1), concatenation of these 12 regions and the nrDNA ITS region.

### Selective pressure analysis

Selective pressures were examined throughout the phylogenetic tree of *Delphinium* for 77 unique PCGs. The Easy-CodeML software [90] in PAML v4 [91] was used to assess the nonsynonymous (*d*_N_) and synonymous (*d*_S_) substitution rates of each plastid gene. The *ω* ratio (*d*_N_ /*d*_S_) indicated the selection pressure on genes, *ω* less than 1 revealed purification selection, equal to 1 revealed neutral evolution, and greater than 1 revealed positive selection [92]. We tested different hypotheses via branch models, M0: the one-ratio model (m0) assumed the same *d*_N_ / *d*_S_ ratio (*ω* ratio) for all branches in the phylogeny; M2: the two-ratio model (m2) assumed the outgroup branch had *ω* ratio that differed from that throughout the rest of the tree [91]. LRTs were used to perform pairwise comparisons of these models [93].

### Electronic supplementary material

Below is the link to the electronic supplementary material.

Supplementary Material 1

Supplementary Material 2

Supplementary Material 3

Supplementary Material 4

Supplementary Material 5

Supplementary Material 6

Supplementary Material 7

Supplementary Material 8

Supplementary Material 9

Supplementary Material 10

Supplementary Material 11

Supplementary Material 12

## Data Availability

The datasets presented in this study can be found in online repositories. The names of the repository and accession number(s) can be found below: https://www.ncbi.nlm.nih.gov/genbank/, OR263583, OR263584, OR263585, OR263586, OR263587, OR263588, OR263589 and OR263590.

## Abbreviations

AA: Amino acid
bs: Bootstrap support
cp: Chloroplast
CBI: Codon bias index
CTAB: Cetyl trimethylammonium bromide
d_N_: Nonsynonymous
d_S_: Synonymous
ENC: Effective number codons
IGS: Intergenic spacers
IRs: Inverted repeats
ITS: Internal transcribed spacer of ribosomalDNA
LRTs: Likelihood ratio tests
LSC: Large singlecopy
ML: Maximumlikelihood
NCBI: National Center for Biotechnology
PCGs: Protein-coding genes
PGA: Plastid genome annotator
Pi: Nucleotide diversity/polymorphism
rRNA: Ribosomal RNA
RSCU: Relative synonymous codon usage
SDR: Short dispersed repeats
SSC: Small singlecopy
SSR: Simple sequence repeat
tRNA: Transfer RNA
