# Supplementary material for: Complete chloroplast genomes of eight Delphinium taxa (Ranunculaceae) endemic to Xinjiang, China: insights into genome structure, comparative analysis, and phylogenetic relationships
Source: BMC Plant Biol. 2024 Jun 26;24:600. doi: 10.1186/s12870-024-05279-y (PMC11201361; doi:10.1186/s12870-024-05279-y)
Supplement: Supplementary file 1 — Supplementary Material 1 [file 12870_2024_5279_MOESM1_ESM.docx]

**TABLE S1** List of annotated genes in the chloroplast genomes of eight newly sequenced *Delphinium* taxa.

| **Category** | **Gene group** | **Gene name** |
| --- | --- | --- |
| Photosynthesis | Subunits of photosystem I | *psa*A, *psa*B, *psa*C, *psa*I, *psa*J |
|  | Subunits of photosystem II | *psb*A, *psb*B, *psb*C, *psb*D, *psb*E, *psb*F, *psb*H, *psb*I, *psb*J, *psb*K, *psb*L, *psb*M, *psb*N, *psb*T, *psb*Z |
|  | Subunits of NADH dehydrogenase | *ndh*A*, *ndh*B*^(2)^, *ndh*C, *ndh*D, *ndh*E, *ndh*F, *ndh*G, *ndh*H, *ndh*I, *ndh*J, *ndh*K |
|  | Subunits of cytochrome b/f complex | *pet*A, *pet*B*, *pet*D*, *pet*G, *pet*L, *pet*N |
|  | Subunits of ATP synthase | *atp*A, *atp*B, *atp*E, *atp*F*, *atp*H, *atp*I |
|  | Large subunit of rubisco | *rbc*L |
|  | Subunits photochlorophyllide reductase | - |
| Self-replication | Proteins of large ribosomal subunit | *rpl*14, *rpl*16*, *rpl*2*^(2)^, *rpl*20, *rpl*22, *rpl*23^(2)^, *rpl*33, *rpl*36 |
|  | Proteins of small ribosomal subunit | *rps*11, *rps*12**^(2)^, *rps*14, *rps*15, *rps*18, *rps*19, *rps*2, *rps*3, *rps*4, *rps*7^(2)^, *rps*8 |
|  | Subunits of RNA polymerase | *rpo*A, *rpo*B, *rpo*C1*, *rpo*C2 |
|  | Ribosomal RNAs | *rrn*16S^(2)^, *rrn*23S^(2)^, *rrn*4.5S^(2)^, *rrn*5S^(2)^ |
|  | Transfer RNAs | *trn*A-TGC***** ^(2)^, *trn*C-GCA, *trn*D-GTC, *trn*E-TTC, *trn*F-GAA, *trn*G-GCC, *trn*G-TCC*****, *trn*H-GTG, *trn*I-CAT^(2)^, *trn*I-GAT*^(2)^, *trn*K-TTT*****, *trn*L-CAA^(2)^**,** *trn*L-TAA*****, *trn*L-TAG, *trn*M-CAT, *trn*N-GTT^(2)^, *trn*P-TGG, *trn*Q-TTG, *trn*R-ACG^(2)^, *trn*R-TCT, *trn*S-GCT, *trn*S-GGA, *trn*S-TGA, *trn*T-GGT, *trn*T-TGT, *trn*V-GAC^(2)^, *trn*V-TAC*****, *trn*W-CCA, *trn*Y-GTA, *trnf*M-CAT |
| Other genes | Maturase | *mat*K |
|  | Protease | *clp*P** |
|  | Envelope membrane protein | *cem*A |
|  | Acetyl-CoA carboxylase | *acc*D |
|  | c-type cytochrome synthesis gene | *ccs*A |
|  | Translation initiation factor | *inf*A |
|  | other | - |
| Genes of unknown function | Conserved hypothetical chloroplast ORF | #*ycf*1, *ycf*1^(2)^, *ycf*2^(2)^, *ycf*3**, *ycf*4 |

**Notes: Gene*: Gene with one introns; Gene**: Gene with two introns; #Gene: Pseudo gene; Gene^(2)^: Number of copies of multi-copy genes;**
