# Supplementary material for: Complete chloroplast genomes of eight Delphinium taxa (Ranunculaceae) endemic to Xinjiang, China: insights into genome structure, comparative analysis, and phylogenetic relationships
Source: BMC Plant Biol. 2024 Jun 26;24:600. doi: 10.1186/s12870-024-05279-y (PMC11201361; doi:10.1186/s12870-024-05279-y)
Supplement: Supplementary file 2 — Supplementary Material 2 [file 12870_2024_5279_MOESM2_ESM.docx]

**TABLE S2** The comparison of SSRs among 14 *Delphinium* taxa chloroplast genomes.

| **Taxon** | **ID** | **SSR NO.** | **SSR type** | **SSR** | **Size** | **Start** | **End** | **Location in Distribution** |
| --- | --- | --- | --- | --- | --- | --- | --- | --- |
| *Delphinium aemulans* | LHM1280 | 1 | p1 | (A)10 | 10 | 4324 | 4333 | LSC |
| *Delphinium aemulans* | LHM1280 | 2 | p1 | (T)12 | 12 | 4790 | 4801 | LSC |
| *Delphinium aemulans* | LHM1280 | 3 | p1 | (T)10 | 10 | 7354 | 7363 | LSC |
| *Delphinium aemulans* | LHM1280 | 4 | p1 | (T)17 | 17 | 7459 | 7475 | LSC |
| *Delphinium aemulans* | LHM1280 | 5 | p1 | (T)10 | 10 | 7722 | 7731 | LSC |
| *Delphinium aemulans* | LHM1280 | 6 | p1 | (T)11 | 11 | 10553 | 10563 | LSC |
| *Delphinium aemulans* | LHM1280 | 7 | p1 | (T)14 | 14 | 16699 | 16712 | LSC |
| *Delphinium aemulans* | LHM1280 | 8 | p1 | (T)10 | 10 | 20748 | 20757 | LSC |
| *Delphinium aemulans* | LHM1280 | 9 | p1 | (T)10 | 10 | 24398 | 24407 | LSC |
| *Delphinium aemulans* | LHM1280 | 10 | p3 | (AAT)5 | 15 | 27033 | 27047 | LSC |
| *Delphinium aemulans* | LHM1280 | 11 | p1 | (A)11 | 11 | 27258 | 27268 | LSC |
| *Delphinium aemulans* | LHM1280 | 12 | p1 | (T)13 | 13 | 28955 | 28967 | LSC |
| *Delphinium aemulans* | LHM1280 | 13 | p1 | (T)10 | 10 | 30738 | 30747 | LSC |
| *Delphinium aemulans* | LHM1280 | 14 | p1 | (A)11 | 11 | 32093 | 32103 | LSC |
| *Delphinium aemulans* | LHM1280 | 15 | p1 | (A)15 | 15 | 35855 | 35869 | LSC |
| *Delphinium aemulans* | LHM1280 | 16 | p1 | (A)10 | 10 | 35999 | 36008 | LSC |
| *Delphinium aemulans* | LHM1280 | 17 | p1 | (C)10 | 10 | 40006 | 40015 | LSC |
| *Delphinium aemulans* | LHM1280 | 18 | p1 | (T)13 | 13 | 41966 | 41978 | LSC |
| *Delphinium aemulans* | LHM1280 | 19 | p1 | (A)10 | 10 | 42170 | 42179 | LSC |
| *Delphinium aemulans* | LHM1280 | 20 | p1 | (A)10 | 10 | 44595 | 44604 | LSC |
| *Delphinium aemulans* | LHM1280 | 21 | p1 | (A)13 | 13 | 46411 | 46423 | LSC |
| *Delphinium aemulans* | LHM1280 | 22 | p1 | (A)11 | 11 | 51470 | 51480 | LSC |
| *Delphinium aemulans* | LHM1280 | 23 | p1 | (A)10 | 10 | 51690 | 51699 | LSC |
| *Delphinium aemulans* | LHM1280 | 24 | p1 | (T)13 | 13 | 52768 | 52780 | LSC |
| *Delphinium aemulans* | LHM1280 | 25 | p1 | (A)12 | 12 | 54835 | 54846 | LSC |
| *Delphinium aemulans* | LHM1280 | 26 | p1 | (A)10 | 10 | 59194 | 59203 | LSC |
| *Delphinium aemulans* | LHM1280 | 27 | p1 | (A)11 | 11 | 59569 | 59579 | LSC |
| *Delphinium aemulans* | LHM1280 | 28 | p1 | (T)10 | 10 | 59859 | 59868 | LSC |
| *Delphinium aemulans* | LHM1280 | 29 | p1 | (T)10 | 10 | 60088 | 60097 | LSC |
| *Delphinium aemulans* | LHM1280 | 30 | p1 | (A)10 | 10 | 61363 | 61372 | LSC |
| *Delphinium aemulans* | LHM1280 | 31 | p1 | (T)10 | 10 | 63591 | 63600 | LSC |
| *Delphinium aemulans* | LHM1280 | 32 | p1 | (T)10 | 10 | 63625 | 63634 | LSC |
| *Delphinium aemulans* | LHM1280 | 33 | p1 | (T)10 | 10 | 66683 | 66692 | LSC |
| *Delphinium aemulans* | LHM1280 | 34 | p2 | (TA)8 | 16 | 66883 | 66898 | LSC |
| *Delphinium aemulans* | LHM1280 | 35 | p1 | (T)11 | 11 | 66948 | 66958 | LSC |
| *Delphinium aemulans* | LHM1280 | 36 | p1 | (T)12 | 12 | 68654 | 68665 | LSC |
| *Delphinium aemulans* | LHM1280 | 37 | p1 | (T)10 | 10 | 70134 | 70143 | LSC |
| *Delphinium aemulans* | LHM1280 | 38 | p1 | (A)10 | 10 | 70819 | 70828 | LSC |
| *Delphinium aemulans* | LHM1280 | 39 | p1 | (A)10 | 10 | 71500 | 71509 | LSC |
| *Delphinium aemulans* | LHM1280 | 40 | p1 | (T)11 | 11 | 71540 | 71550 | LSC |
| *Delphinium aemulans* | LHM1280 | 41 | p1 | (T)10 | 10 | 80431 | 80440 | LSC |
| *Delphinium aemulans* | LHM1280 | 42 | p1 | (T)11 | 11 | 80948 | 80958 | LSC |
| *Delphinium aemulans* | LHM1280 | 43 | p1 | (T)10 | 10 | 82394 | 82403 | LSC |
| *Delphinium aemulans* | LHM1280 | 44 | p1 | (T)10 | 10 | 99363 | 99372 | IR |
| *Delphinium aemulans* | LHM1280 | 45 | p1 | (T)10 | 10 | 108814 | 108823 | IR |
| *Delphinium aemulans* | LHM1280 | 46 | p1 | (A)16 | 16 | 114179 | 114194 | SSC |
| *Delphinium aemulans* | LHM1280 | 47 | p1 | (T)10 | 10 | 118940 | 118949 | SSC |
| *Delphinium aemulans* | LHM1280 | 48 | p1 | (T)12 | 12 | 123599 | 123610 | SSC |
| *Delphinium aemulans* | LHM1280 | 49 | p1 | (T)10 | 10 | 124345 | 124354 | SSC |
| *Delphinium aemulans* | LHM1280 | 50 | p1 | (T)13 | 13 | 125194 | 125206 | SSC |
| *Delphinium aemulans* | LHM1280 | 51 | p1 | (T)12 | 28 | 126013 | 126024 | SSC |
| *Delphinium aemulans* | LHM1280 | 52 | p1 | (T)10 | 10 | 126031 | 126040 | SSC |
| *Delphinium aemulans* | LHM1280 | 53 | p1 | (T)11 | 11 | 126076 | 126086 | SSC |
| *Delphinium aemulans* | LHM1280 | 54 | p1 | (A)10 | 10 | 130232 | 130241 | IR |
| *Delphinium aemulans* | LHM1280 | 55 | p1 | (A)10 | 10 | 139683 | 139692 | IR |
| *Delphinium anthriscifolium* | MK253461 | 1 | p1 | (A)11 | 11 | 3644 | 3654 | LSC |
| *Delphinium anthriscifolium* | MK253461 | 2 | p1 | (A)14 | 14 | 4963 | 4976 | LSC |
| *Delphinium anthriscifolium* | MK253461 | 3 | p1 | (T)10 | 10 | 7518 | 7527 | LSC |
| *Delphinium anthriscifolium* | MK253461 | 4 | p1 | (T)10 | 10 | 7776 | 7785 | LSC |
| *Delphinium anthriscifolium* | MK253461 | 5 | p1 | (T)17 | 17 | 10614 | 10630 | LSC |
| *Delphinium anthriscifolium* | MK253461 | 6 | p2 | (AT)7 | 14 | 12976 | 12989 | LSC |
| *Delphinium anthriscifolium* | MK253461 | 7 | p1 | (T)14 | 14 | 16971 | 16984 | LSC |
| *Delphinium anthriscifolium* | MK253461 | 8 | p1 | (A)10 | 10 | 22059 | 22068 | LSC |
| *Delphinium anthriscifolium* | MK253461 | 9 | p1 | (T)10 | 10 | 24652 | 24661 | LSC |
| *Delphinium anthriscifolium* | MK253461 | 10 | p2 | (AT)6 | 12 | 25949 | 25960 | LSC |
| *Delphinium anthriscifolium* | MK253461 | 11 | p1 | (G)11 | 11 | 26437 | 26447 | LSC |
| *Delphinium anthriscifolium* | MK253461 | 12 | p1 | (T)11 | 11 | 28950 | 28960 | LSC |
| *Delphinium anthriscifolium* | MK253461 | 13 | p2 | (AT)7 | 14 | 29692 | 29705 | LSC |
| *Delphinium anthriscifolium* | MK253461 | 14 | p1 | (A)10 | 10 | 29887 | 29896 | LSC |
| *Delphinium anthriscifolium* | MK253461 | 15 | p2 | (TA)6 | 12 | 30955 | 30966 | LSC |
| *Delphinium anthriscifolium* | MK253461 | 16 | p1 | (A)13 | 13 | 31967 | 31979 | LSC |
| *Delphinium anthriscifolium* | MK253461 | 17 | p1 | (A)10 | 10 | 36540 | 36549 | LSC |
| *Delphinium anthriscifolium* | MK253461 | 18 | p1 | (C)10 | 10 | 40603 | 40612 | LSC |
| *Delphinium anthriscifolium* | MK253461 | 19 | p1 | (A)10 | 10 | 42786 | 42795 | LSC |
| *Delphinium anthriscifolium* | MK253461 | 20 | p1 | (A)12 | 12 | 44184 | 44195 | LSC |
| *Delphinium anthriscifolium* | MK253461 | 21 | p1 | (G)10 | 10 | 45997 | 46006 | LSC |
| *Delphinium anthriscifolium* | MK253461 | 22 | p1 | (A)14 | 14 | 47017 | 47030 | LSC |
| *Delphinium anthriscifolium* | MK253461 | 23 | p1 | (T)15 | 15 | 49447 | 49461 | LSC |
| *Delphinium anthriscifolium* | MK253461 | 24 | p1 | (A)11 | 11 | 52343 | 52353 | LSC |
| *Delphinium anthriscifolium* | MK253461 | 25 | p1 | (T)10 | 10 | 52521 | 52530 | LSC |
| *Delphinium anthriscifolium* | MK253461 | 26 | p1 | (T)10 | 10 | 53421 | 53430 | LSC |
| *Delphinium anthriscifolium* | MK253461 | 27 | p1 | (A)13 | 13 | 55525 | 55537 | LSC |
| *Delphinium anthriscifolium* | MK253461 | 28 | p1 | (A)13 | 13 | 60303 | 60315 | LSC |
| *Delphinium anthriscifolium* | MK253461 | 29 | p1 | (T)10 | 10 | 60596 | 60605 | LSC |
| *Delphinium anthriscifolium* | MK253461 | 30 | p1 | (G)11 | 11 | 62246 | 62256 | LSC |
| *Delphinium anthriscifolium* | MK253461 | 31 | p1 | (A)10 | 10 | 64264 | 64273 | LSC |
| *Delphinium anthriscifolium* | MK253461 | 32 | p2 | (TA)6 | 12 | 67313 | 67324 | LSC |
| *Delphinium anthriscifolium* | MK253461 | 33 | p1 | (T)16 | 16 | 68441 | 68456 | LSC |
| *Delphinium anthriscifolium* | MK253461 | 34 | p1 | (A)11 | 11 | 68750 | 68760 | LSC |
| *Delphinium anthriscifolium* | MK253461 | 35 | p1 | (T)18 | 18 | 70797 | 70814 | LSC |
| *Delphinium anthriscifolium* | MK253461 | 36 | p1 | (G)11 | 11 | 71675 | 71685 | LSC |
| *Delphinium anthriscifolium* | MK253461 | 37 | p1 | (T)11 | 11 | 72542 | 72552 | LSC |
| *Delphinium anthriscifolium* | MK253461 | 38 | p1 | (A)10 | 10 | 79290 | 79299 | LSC |
| *Delphinium anthriscifolium* | MK253461 | 39 | p1 | (T)14 | 14 | 82578 | 82591 | LSC |
| *Delphinium anthriscifolium* | MK253461 | 40 | p1 | (T)11 | 11 | 100449 | 100459 | IR |
| *Delphinium anthriscifolium* | MK253461 | 41 | p2 | (TA)6 | 12 | 111958 | 111969 | SSC |
| *Delphinium anthriscifolium* | MK253461 | 42 | p1 | (A)10 | 10 | 114285 | 114294 | SSC |
| *Delphinium anthriscifolium* | MK253461 | 43 | p1 | (A)10 | 10 | 114545 | 114554 | SSC |
| *Delphinium anthriscifolium* | MK253461 | 44 | p1 | (A)10 | 10 | 114804 | 114813 | SSC |
| *Delphinium anthriscifolium* | MK253461 | 45 | p1 | (A)10 | 10 | 116498 | 116507 | SSC |
| *Delphinium anthriscifolium* | MK253461 | 46 | p1 | (T)10 | 10 | 116511 | 116520 | SSC |
| *Delphinium anthriscifolium* | MK253461 | 47 | p2 | (AT)6 | 12 | 119888 | 119899 | SSC |
| *Delphinium anthriscifolium* | MK253461 | 48 | p1 | (A)12 | 12 | 122175 | 122186 | SSC |
| *Delphinium anthriscifolium* | MK253461 | 49 | p1 | (T)11 | 11 | 124397 | 124407 | SSC |
| *Delphinium anthriscifolium* | MK253461 | 50 | p1 | (T)11 | 11 | 125983 | 125993 | SSC |
| *Delphinium anthriscifolium* | MK253461 | 51 | p1 | (A)11 | 11 | 140490 | 140500 | IR |
| *Delphinium brunonianum* | NC_051554 | 1 | p1 | (A)10 | 10 | 4314 | 4323 | LSC |
| *Delphinium brunonianum* | NC_051554 | 2 | p1 | (A)15 | 15 | 4528 | 4542 | LSC |
| *Delphinium brunonianum* | NC_051554 | 3 | p1 | (A)16 | 16 | 6843 | 6858 | LSC |
| *Delphinium brunonianum* | NC_051554 | 4 | p1 | (T)10 | 10 | 7351 | 7360 | LSC |
| *Delphinium brunonianum* | NC_051554 | 5 | p1 | (T)18 | 18 | 7456 | 7473 | LSC |
| *Delphinium brunonianum* | NC_051554 | 6 | p1 | (T)11 | 11 | 7727 | 7737 | LSC |
| *Delphinium brunonianum* | NC_051554 | 7 | p1 | (T)12 | 12 | 10428 | 10439 | LSC |
| *Delphinium brunonianum* | NC_051554 | 8 | p1 | (T)13 | 13 | 10572 | 10584 | LSC |
| *Delphinium brunonianum* | NC_051554 | 9 | p1 | (T)14 | 14 | 16689 | 16702 | LSC |
| *Delphinium brunonianum* | NC_051554 | 10 | p1 | (T)10 | 10 | 20737 | 20746 | LSC |
| *Delphinium brunonianum* | NC_051554 | 11 | p1 | (T)10 | 10 | 24387 | 24396 | LSC |
| *Delphinium brunonianum* | NC_051554 | 12 | p3 | (AAT)5 | 15 | 27036 | 27050 | LSC |
| *Delphinium brunonianum* | NC_051554 | 13 | p1 | (A)11 | 11 | 27262 | 27272 | LSC |
| *Delphinium brunonianum* | NC_051554 | 14 | p1 | (A)10 | 10 | 27976 | 27985 | LSC |
| *Delphinium brunonianum* | NC_051554 | 15 | p1 | (T)10 | 10 | 28962 | 28971 | LSC |
| *Delphinium brunonianum* | NC_051554 | 16 | p1 | (T)10 | 10 | 30736 | 30745 | LSC |
| *Delphinium brunonianum* | NC_051554 | 17 | p1 | (A)10 | 10 | 32162 | 32171 | LSC |
| *Delphinium brunonianum* | NC_051554 | 18 | p1 | (A)11 | 10 | 32181 | 32191 | LSC |
| *Delphinium brunonianum* | NC_051554 | 19 | p1 | (A)10 | 10 | 36080 | 36089 | LSC |
| *Delphinium brunonianum* | NC_051554 | 20 | p1 | (C)10 | 10 | 40088 | 40097 | LSC |
| *Delphinium brunonianum* | NC_051554 | 21 | p1 | (T)12 | 12 | 42048 | 42059 | LSC |
| *Delphinium brunonianum* | NC_051554 | 22 | p1 | (A)10 | 10 | 42251 | 42260 | LSC |
| *Delphinium brunonianum* | NC_051554 | 23 | p1 | (A)12 | 12 | 46471 | 46482 | LSC |
| *Delphinium brunonianum* | NC_051554 | 24 | p1 | (T)10 | 10 | 48826 | 48835 | LSC |
| *Delphinium brunonianum* | NC_051554 | 25 | p1 | (A)12 | 12 | 59181 | 59192 | LSC |
| *Delphinium brunonianum* | NC_051554 | 26 | p1 | (T)10 | 10 | 59849 | 59858 | LSC |
| *Delphinium brunonianum* | NC_051554 | 27 | p1 | (T)10 | 10 | 60078 | 60087 | LSC |
| *Delphinium brunonianum* | NC_051554 | 28 | p1 | (A)13 | 13 | 61357 | 61369 | LSC |
| *Delphinium brunonianum* | NC_051554 | 29 | p1 | (A)11 | 11 | 63401 | 63411 | LSC |
| *Delphinium brunonianum* | NC_051554 | 30 | p1 | (A)11 | 11 | 65625 | 65635 | LSC |
| *Delphinium brunonianum* | NC_051554 | 31 | p2 | (TA)7 | 14 | 66622 | 66635 | LSC |
| *Delphinium brunonianum* | NC_051554 | 32 | p1 | (T)10 | 10 | 66723 | 66732 | LSC |
| *Delphinium brunonianum* | NC_051554 | 33 | p1 | (A)10 | 10 | 66816 | 66825 | LSC |
| *Delphinium brunonianum* | NC_051554 | 34 | p2 | (AT)7 | 14 | 67792 | 67805 | LSC |
| *Delphinium brunonianum* | NC_051554 | 35 | p1 | (T)11 | 11 | 67849 | 67859 | LSC |
| *Delphinium brunonianum* | NC_051554 | 36 | p1 | (T)15 | 15 | 68347 | 68361 | LSC |
| *Delphinium brunonianum* | NC_051554 | 37 | p1 | (T)11 | 11 | 69835 | 69845 | LSC |
| *Delphinium brunonianum* | NC_051554 | 38 | p1 | (A)11 | 11 | 70521 | 70531 | LSC |
| *Delphinium brunonianum* | NC_051554 | 39 | p1 | (A)10 | 10 | 71189 | 71198 | LSC |
| *Delphinium brunonianum* | NC_051554 | 40 | p1 | (T)10 | 10 | 71241 | 71250 | LSC |
| *Delphinium brunonianum* | NC_051554 | 41 | p1 | (T)11 | 11 | 80129 | 80139 | LSC |
| *Delphinium brunonianum* | NC_051554 | 42 | p1 | (T)11 | 11 | 80647 | 80657 | LSC |
| *Delphinium brunonianum* | NC_051554 | 43 | p1 | (T)10 | 10 | 81204 | 81213 | LSC |
| *Delphinium brunonianum* | NC_051554 | 44 | p1 | (T)10 | 10 | 82095 | 82104 | LSC |
| *Delphinium brunonianum* | NC_051554 | 45 | p1 | (A)11 | 11 | 82390 | 82400 | LSC |
| *Delphinium brunonianum* | NC_051554 | 46 | p1 | (T)10 | 10 | 99065 | 99074 | IR |
| *Delphinium brunonianum* | NC_051554 | 47 | p1 | (T)10 | 10 | 113307 | 113316 | SSC |
| *Delphinium brunonianum* | NC_051554 | 48 | p1 | (A)10 | 10 | 113875 | 113884 | SSC |
| *Delphinium brunonianum* | NC_051554 | 49 | p2 | (TA)6 | 12 | 117602 | 117613 | SSC |
| *Delphinium brunonianum* | NC_051554 | 50 | p1 | (T)10 | 10 | 118643 | 118652 | SSC |
| *Delphinium brunonianum* | NC_051554 | 51 | p2 | (TA)6 | 12 | 118786 | 118797 | SSC |
| *Delphinium brunonianum* | NC_051554 | 52 | p1 | (T)11 | 11 | 123276 | 123286 | SSC |
| *Delphinium brunonianum* | NC_051554 | 53 | p1 | (T)10 | 10 | 124022 | 124031 | SSC |
| *Delphinium brunonianum* | NC_051554 | 53 | p1 | (T)12 | 12 | 125696 | 125707 | SSC |
| *Delphinium brunonianum* | NC_051554 | 54 | p1 | (T)10 | 10 | 125714 | 125723 | SSC |
| *Delphinium brunonianum* | NC_051554 | 55 | p1 | (T)11 | 11 | 125759 | 125769 | SSC |
| *Delphinium brunonianum* | NC_051554 | 56 | p1 | (A)10 | 10 | 139365 | 139374 | IR |
| *Delphinium candelabrum* var. *monanthum* | MW246165 | 1 | p1 | (A)19 | 19 | 4525 | 4543 | LSC |
| *Delphinium candelabrum* var. *monanthum* | MW246165 | 2 | p1 | (A)12 | 12 | 6873 | 6884 | LSC |
| *Delphinium candelabrum* var. *monanthum* | MW246165 | 3 | p1 | (T)11 | 11 | 7375 | 7385 | LSC |
| *Delphinium candelabrum* var. *monanthum* | MW246165 | 4 | p1 | (T)14 | 14 | 7481 | 7494 | LSC |
| *Delphinium candelabrum* var. *monanthum* | MW246165 | 5 | p1 | (T)14 | 14 | 16699 | 16712 | LSC |
| *Delphinium candelabrum* var. *monanthum* | MW246165 | 6 | p1 | (T)10 | 10 | 24396 | 24405 | LSC |
| *Delphinium candelabrum* var. *monanthum* | MW246165 | 7 | p3 | (AAT)5 | 15 | 27057 | 27071 | LSC |
| *Delphinium candelabrum* var. *monanthum* | MW246165 | 8 | p1 | (A)10 | 10 | 27285 | 27294 | LSC |
| *Delphinium candelabrum* var. *monanthum* | MW246165 | 9 | p1 | (A)10 | 10 | 27996 | 28005 | LSC |
| *Delphinium candelabrum* var. *monanthum* | MW246165 | 10 | p1 | (T)12 | 12 | 28975 | 28986 | LSC |
| *Delphinium candelabrum* var. *monanthum* | MW246165 | 11 | p2 | (TA)6 | 12 | 31822 | 31833 | LSC |
| *Delphinium candelabrum* var. *monanthum* | MW246165 | 12 | p1 | (C)10 | 10 | 40101 | 40110 | LSC |
| *Delphinium candelabrum* var. *monanthum* | MW246165 | 13 | p1 | (T)12 | 12 | 42061 | 42072 | LSC |
| *Delphinium candelabrum* var. *monanthum* | MW246165 | 14 | p1 | (A)10 | 10 | 43633 | 43642 | LSC |
| *Delphinium candelabrum* var. *monanthum* | MW246165 | 15 | p1 | (A)10 | 10 | 46490 | 46499 | LSC |
| *Delphinium candelabrum* var. *monanthum* | MW246165 | 16 | p1 | (T)14 | 14 | 48885 | 48898 | LSC |
| *Delphinium candelabrum* var. *monanthum* | MW246165 | 17 | p1 | (A)10 | 10 | 51543 | 51552 | LSC |
| *Delphinium candelabrum* var. *monanthum* | MW246165 | 18 | p1 | (A)10 | 10 | 51762 | 51771 | LSC |
| *Delphinium candelabrum* var. *monanthum* | MW246165 | 19 | p1 | (T)10 | 10 | 52847 | 52856 | LSC |
| *Delphinium candelabrum* var. *monanthum* | MW246165 | 20 | p1 | (A)15 | 15 | 54906 | 54920 | LSC |
| *Delphinium candelabrum* var. *monanthum* | MW246165 | 21 | p1 | (A)11 | 11 | 59251 | 59261 | LSC |
| *Delphinium candelabrum* var. *monanthum* | MW246165 | 22 | p1 | (A)12 | 12 | 59630 | 59641 | LSC |
| *Delphinium candelabrum* var. *monanthum* | MW246165 | 23 | p1 | (T)11 | 11 | 59921 | 59931 | LSC |
| *Delphinium candelabrum* var. *monanthum* | MW246165 | 24 | p1 | (T)10 | 10 | 60151 | 60160 | LSC |
| *Delphinium candelabrum* var. *monanthum* | MW246165 | 25 | p1 | (G)11 | 11 | 61459 | 61469 | LSC |
| *Delphinium candelabrum* var. *monanthum* | MW246165 | 26 | p1 | (T)10 | 10 | 63686 | 63695 | LSC |
| *Delphinium candelabrum* var. *monanthum* | MW246165 | 27 | p1 | (T)11 | 11 | 63719 | 63729 | LSC |
| *Delphinium candelabrum* var. *monanthum* | MW246165 | 28 | p1 | (A)10 | 10 | 65705 | 65714 | LSC |
| *Delphinium candelabrum* var. *monanthum* | MW246165 | 29 | p1 | (A)10 | 10 | 65948 | 65957 | LSC |
| *Delphinium candelabrum* var. *monanthum* | MW246165 | 30 | p2 | (TA)8 | 16 | 66948 | 66963 | LSC |
| *Delphinium candelabrum* var. *monanthum* | MW246165 | 31 | p1 | (T)11 | 11 | 66995 | 67005 | LSC |
| *Delphinium candelabrum* var. *monanthum* | MW246165 | 32 | p1 | (T)11 | 11 | 67032 | 67042 | LSC |
| *Delphinium candelabrum* var. *monanthum* | MW246165 | 33 | p1 | (A)14 | 14 | 67171 | 67184 | LSC |
| *Delphinium candelabrum* var. *monanthum* | MW246165 | 34 | p1 | (T)11 | 11 | 67807 | 67817 | LSC |
| *Delphinium candelabrum* var. *monanthum* | MW246165 | 35 | p1 | (A)10 | 10 | 67857 | 67866 | LSC |
| *Delphinium candelabrum* var. *monanthum* | MW246165 | 36 | p2 | (AT)7 | 14 | 68150 | 68163 | LSC |
| *Delphinium candelabrum* var. *monanthum* | MW246165 | 37 | p1 | (T)11 | 11 | 68208 | 68218 | LSC |
| *Delphinium candelabrum* var. *monanthum* | MW246165 | 38 | p1 | (T)10 | 10 | 68707 | 68716 | LSC |
| *Delphinium candelabrum* var. *monanthum* | MW246165 | 39 | p1 | (A)10 | 10 | 70854 | 70863 | LSC |
| *Delphinium candelabrum* var. *monanthum* | MW246165 | 40 | p1 | (A)10 | 10 | 71521 | 71530 | LSC |
| *Delphinium candelabrum* var. *monanthum* | MW246165 | 41 | p1 | (T)11 | 11 | 71574 | 71584 | LSC |
| *Delphinium candelabrum* var. *monanthum* | MW246165 | 42 | p1 | (T)13 | 13 | 80970 | 80982 | LSC |
| *Delphinium candelabrum* var. *monanthum* | MW246165 | 43 | p1 | (T)16 | 16 | 82297 | 82312 | LSC |
| *Delphinium candelabrum* var. *monanthum* | MW246165 | 44 | p1 | (A)10 | 10 | 93144 | 93153 | IR |
| *Delphinium candelabrum* var. *monanthum* | MW246165 | 45 | p1 | (T)10 | 10 | 99415 | 99424 | IR |
| *Delphinium candelabrum* var. *monanthum* | MW246165 | 46 | p1 | (T)11 | 11 | 108848 | 108858 | IR |
| *Delphinium candelabrum* var. *monanthum* | MW246165 | 47 | p1 | (A)11 | 11 | 113950 | 113960 | SSC |
| *Delphinium candelabrum* var. *monanthum* | MW246165 | 48 | p1 | (T)10 | 10 | 115551 | 115560 | SSC |
| *Delphinium candelabrum* var. *monanthum* | MW246165 | 49 | p1 | (T)10 | 10 | 118726 | 118735 | SSC |
| *Delphinium candelabrum* var. *monanthum* | MW246165 | 50 | p2 | (TA)9 | 18 | 118876 | 118893 | SSC |
| *Delphinium candelabrum* var. *monanthum* | MW246165 | 51 | p1 | (T)14 | 14 | 123414 | 123427 | SSC |
| *Delphinium candelabrum* var. *monanthum* | MW246165 | 52 | p1 | (A)10 | 10 | 123520 | 123529 | SSC |
| *Delphinium candelabrum* var. *monanthum* | MW246165 | 53 | p1 | (T)10 | 10 | 124158 | 124167 | SSC |
| *Delphinium candelabrum* var. *monanthum* | MW246165 | 54 | p1 | (T)10 | 10 | 125829 | 125838 | SSC |
| *Delphinium candelabrum* var. *monanthum* | MW246165 | 55 | p1 | (A)11 | 11 | 130000 | 130010 | IR |
| *Delphinium candelabrum* var. *monanthum* | MW246165 | 56 | p1 | (A)10 | 10 | 139434 | 139443 | IR |
| *Delphinium candelabrum* var. *monanthum* | MW246165 | 57 | p1 | (T)10 | 10 | 145705 | 145714 | IR |
| *Delphinium ceratophorum* | MK253460 | 1 | p1 | (A)11 | 11 | 4541 | 4551 | LSC |
| *Delphinium ceratophorum* | MK253460 | 2 | p1 | (A)14 | 14 | 6880 | 6893 | LSC |
| *Delphinium ceratophorum* | MK253460 | 3 | p1 | (T)12 | 12 | 7488 | 7499 | LSC |
| *Delphinium ceratophorum* | MK253460 | 4 | p1 | (T)11 | 11 | 10585 | 10595 | LSC |
| *Delphinium ceratophorum* | MK253460 | 5 | p1 | (T)14 | 14 | 16733 | 16746 | LSC |
| *Delphinium ceratophorum* | MK253460 | 6 | p1 | (T)10 | 10 | 20778 | 20787 | LSC |
| *Delphinium ceratophorum* | MK253460 | 7 | p1 | (T)10 | 10 | 24429 | 24438 | LSC |
| *Delphinium ceratophorum* | MK253460 | 8 | p3 | (AAT)5 | 15 | 27072 | 27086 | LSC |
| *Delphinium ceratophorum* | MK253460 | 9 | p1 | (A)10 | 10 | 27300 | 27309 | LSC |
| *Delphinium ceratophorum* | MK253460 | 10 | p1 | (T)12 | 12 | 28988 | 28999 | LSC |
| *Delphinium ceratophorum* | MK253460 | 11 | p1 | (T)10 | 10 | 30351 | 30360 | LSC |
| *Delphinium ceratophorum* | MK253460 | 12 | p1 | (A)10 | 10 | 32193 | 32202 | LSC |
| *Delphinium ceratophorum* | MK253460 | 13 | p1 | (A)10 | 10 | 32211 | 32220 | LSC |
| *Delphinium ceratophorum* | MK253460 | 14 | p1 | (A)11 | 11 | 35978 | 35988 | LSC |
| *Delphinium ceratophorum* | MK253460 | 15 | p1 | (C)10 | 10 | 40124 | 40133 | LSC |
| *Delphinium ceratophorum* | MK253460 | 16 | p1 | (T)12 | 12 | 42084 | 42095 | LSC |
| *Delphinium ceratophorum* | MK253460 | 17 | p1 | (A)12 | 12 | 42287 | 42298 | LSC |
| *Delphinium ceratophorum* | MK253460 | 18 | p1 | (A)11 | 11 | 43665 | 43675 | LSC |
| *Delphinium ceratophorum* | MK253460 | 19 | p1 | (A)13 | 13 | 46517 | 46529 | LSC |
| *Delphinium ceratophorum* | MK253460 | 20 | p1 | (T)10 | 10 | 48931 | 48940 | LSC |
| *Delphinium ceratophorum* | MK253460 | 21 | p1 | (A)10 | 10 | 51605 | 51614 | LSC |
| *Delphinium ceratophorum* | MK253460 | 22 | p1 | (A)11 | 11 | 51791 | 51801 | LSC |
| *Delphinium ceratophorum* | MK253460 | 23 | p1 | (T)11 | 11 | 52909 | 52919 | LSC |
| *Delphinium ceratophorum* | MK253460 | 24 | p1 | (A)15 | 15 | 54974 | 54988 | LSC |
| *Delphinium ceratophorum* | MK253460 | 25 | p1 | (A)11 | 11 | 59321 | 59331 | LSC |
| *Delphinium ceratophorum* | MK253460 | 26 | p1 | (T)10 | 10 | 59988 | 59997 | LSC |
| *Delphinium ceratophorum* | MK253460 | 27 | p1 | (A)11 | 11 | 61383 | 61393 | LSC |
| *Delphinium ceratophorum* | MK253460 | 28 | p1 | (A)10 | 10 | 63424 | 63433 | LSC |
| *Delphinium ceratophorum* | MK253460 | 29 | p1 | (T)12 | 12 | 65465 | 65476 | LSC |
| *Delphinium ceratophorum* | MK253460 | 30 | p2 | (TA)8 | 16 | 66884 | 66899 | LSC |
| *Delphinium ceratophorum* | MK253460 | 31 | p1 | (T)10 | 10 | 66987 | 66996 | LSC |
| *Delphinium ceratophorum* | MK253460 | 32 | p1 | (A)13 | 13 | 67129 | 67141 | LSC |
| *Delphinium ceratophorum* | MK253460 | 33 | p1 | (A)10 | 10 | 67521 | 67530 | LSC |
| *Delphinium ceratophorum* | MK253460 | 34 | p1 | (T)10 | 10 | 68160 | 68169 | LSC |
| *Delphinium ceratophorum* | MK253460 | 35 | p1 | (T)12 | 12 | 68659 | 68670 | LSC |
| *Delphinium ceratophorum* | MK253460 | 36 | p1 | (T)10 | 10 | 70125 | 70134 | LSC |
| *Delphinium ceratophorum* | MK253460 | 37 | p1 | (A)12 | 12 | 70809 | 70820 | LSC |
| *Delphinium ceratophorum* | MK253460 | 38 | p1 | (A)11 | 11 | 71478 | 71488 | LSC |
| *Delphinium ceratophorum* | MK253460 | 39 | p1 | (T)13 | 13 | 71532 | 71544 | LSC |
| *Delphinium ceratophorum* | MK253460 | 40 | p1 | (T)10 | 10 | 81489 | 81498 | LSC |
| *Delphinium ceratophorum* | MK253460 | 41 | p1 | (T)18 | 18 | 82379 | 82396 | LSC |
| *Delphinium ceratophorum* | MK253460 | 42 | p1 | (T)10 | 10 | 99355 | 99364 | IR |
| *Delphinium ceratophorum* | MK253460 | 43 | p1 | (A)10 | 10 | 113924 | 113933 | SSC |
| *Delphinium ceratophorum* | MK253460 | 44 | p1 | (T)16 | 16 | 115780 | 115795 | SSC |
| *Delphinium ceratophorum* | MK253460 | 45 | p1 | (A)10 | 10 | 117406 | 117415 | SSC |
| *Delphinium ceratophorum* | MK253460 | 46 | p2 | (TA)8 | 16 | 117916 | 117931 | SSC |
| *Delphinium ceratophorum* | MK253460 | 47 | p1 | (T)12 | 12 | 118956 | 118967 | SSC |
| *Delphinium ceratophorum* | MK253460 | 48 | p1 | (T)12 | 12 | 123614 | 123625 | SSC |
| *Delphinium ceratophorum* | MK253460 | 49 | p1 | (T)10 | 10 | 123708 | 123717 | SSC |
| *Delphinium ceratophorum* | MK253460 | 50 | p1 | (T)10 | 10 | 124358 | 124367 | SSC |
| *Delphinium ceratophorum* | MK253460 | 51 | p1 | (T)11 | 11 | 126026 | 126036 | SSC |
| *Delphinium ceratophorum* | MK253460 | 52 | p1 | (T)12 | 12 | 126043 | 126054 | SSC |
| *Delphinium ceratophorum* | MK253460 | 53 | p1 | (A)10 | 10 | 139683 | 139692 | IR |
| *Delphinium elatum* var. *sericeum* | LHM1265 | 1 | p1 | (T)11 | 11 | 4783 | 4793 | LSC |
| *Delphinium elatum* var. *sericeum* | LHM1265 | 2 | p1 | (T)10 | 10 | 6752 | 6761 | LSC |
| *Delphinium elatum* var. *sericeum* | LHM1265 | 3 | p1 | (A)10 | 10 | 6848 | 6857 | LSC |
| *Delphinium elatum* var. *sericeum* | LHM1265 | 4 | p1 | (T)11 | 11 | 7349 | 7359 | LSC |
| *Delphinium elatum* var. *sericeum* | LHM1265 | 5 | p1 | (T)16 | 16 | 7455 | 7470 | LSC |
| *Delphinium elatum* var. *sericeum* | LHM1265 | 6 | p1 | (T)10 | 10 | 10547 | 10556 | LSC |
| *Delphinium elatum* var. *sericeum* | LHM1265 | 7 | p1 | (T)14 | 14 | 16673 | 16686 | LSC |
| *Delphinium elatum* var. *sericeum* | LHM1265 | 8 | p1 | (T)10 | 10 | 24372 | 24381 | LSC |
| *Delphinium elatum* var. *sericeum* | LHM1265 | 9 | p3 | (AAT)5 | 15 | 27001 | 27015 | LSC |
| *Delphinium elatum* var. *sericeum* | LHM1265 | 10 | p1 | (A)11 | 11 | 27226 | 27236 | LSC |
| *Delphinium elatum* var. *sericeum* | LHM1265 | 11 | p1 | (A)10 | 10 | 27944 | 27953 | LSC |
| *Delphinium elatum* var. *sericeum* | LHM1265 | 12 | p1 | (T)12 | 12 | 28924 | 28935 | LSC |
| *Delphinium elatum* var. *sericeum* | LHM1265 | 13 | p1 | (T)10 | 10 | 30711 | 30720 | LSC |
| *Delphinium elatum* var. *sericeum* | LHM1265 | 14 | p1 | (A)11 | 11 | 32072 | 32082 | LSC |
| *Delphinium elatum* var. *sericeum* | LHM1265 | 15 | p1 | (A)12 | 12 | 35834 | 35845 | LSC |
| *Delphinium elatum* var. *sericeum* | LHM1265 | 16 | p1 | (A)13 | 13 | 35975 | 35987 | LSC |
| *Delphinium elatum* var. *sericeum* | LHM1265 | 17 | p1 | (C)10 | 10 | 39985 | 39994 | LSC |
| *Delphinium elatum* var. *sericeum* | LHM1265 | 18 | p1 | (T)13 | 13 | 41945 | 41957 | LSC |
| *Delphinium elatum* var. *sericeum* | LHM1265 | 19 | p1 | (A)10 | 10 | 42149 | 42158 | LSC |
| *Delphinium elatum* var. *sericeum* | LHM1265 | 20 | p1 | (A)11 | 11 | 44576 | 44586 | LSC |
| *Delphinium elatum* var. *sericeum* | LHM1265 | 21 | p1 | (A)12 | 12 | 46393 | 46404 | LSC |
| *Delphinium elatum* var. *sericeum* | LHM1265 | 22 | p1 | (A)10 | 10 | 51662 | 51671 | LSC |
| *Delphinium elatum* var. *sericeum* | LHM1265 | 23 | p1 | (T)13 | 13 | 52740 | 52752 | LSC |
| *Delphinium elatum* var. *sericeum* | LHM1265 | 24 | p1 | (A)10 | 10 | 54807 | 54816 | LSC |
| *Delphinium elatum* var. *sericeum* | LHM1265 | 25 | p1 | (A)11 | 11 | 59152 | 59162 | LSC |
| *Delphinium elatum* var. *sericeum* | LHM1265 | 26 | p1 | (A)11 | 11 | 59528 | 59538 | LSC |
| *Delphinium elatum* var. *sericeum* | LHM1265 | 27 | p1 | (T)10 | 10 | 59818 | 59827 | LSC |
| *Delphinium elatum* var. *sericeum* | LHM1265 | 28 | p1 | (T)10 | 10 | 60047 | 60056 | LSC |
| *Delphinium elatum* var. *sericeum* | LHM1265 | 29 | p1 | (A)10 | 10 | 61322 | 61331 | LSC |
| *Delphinium elatum* var. *sericeum* | LHM1265 | 30 | p1 | (A)12 | 12 | 63361 | 63372 | LSC |
| *Delphinium elatum* var. *sericeum* | LHM1265 | 31 | p1 | (T)13 | 13 | 63552 | 63564 | LSC |
| *Delphinium elatum* var. *sericeum* | LHM1265 | 32 | p1 | (T)21 | 21 | 63585 | 63605 | LSC |
| *Delphinium elatum* var. *sericeum* | LHM1265 | 33 | p1 | (A)10 | 10 | 65829 | 65838 | LSC |
| *Delphinium elatum* var. *sericeum* | LHM1265 | 34 | p1 | (T)10 | 10 | 66125 | 66134 | LSC |
| *Delphinium elatum* var. *sericeum* | LHM1265 | 35 | p2 | (TA)8 | 16 | 66854 | 66869 | LSC |
| *Delphinium elatum* var. *sericeum* | LHM1265 | 36 | p1 | (T)13 | 13 | 68619 | 68631 | LSC |
| *Delphinium elatum* var. *sericeum* | LHM1265 | 37 | p1 | (T)10 | 10 | 70101 | 70110 | LSC |
| *Delphinium elatum* var. *sericeum* | LHM1265 | 38 | p1 | (A)11 | 11 | 70787 | 70797 | LSC |
| *Delphinium elatum* var. *sericeum* | LHM1265 | 39 | p1 | (T)12 | 12 | 71508 | 71519 | LSC |
| *Delphinium elatum* var. *sericeum* | LHM1265 | 40 | p1 | (A)11 | 11 | 75705 | 75715 | LSC |
| *Delphinium elatum* var. *sericeum* | LHM1265 | 41 | p1 | (T)11 | 11 | 80400 | 80410 | LSC |
| *Delphinium elatum* var. *sericeum* | LHM1265 | 42 | p1 | (T)10 | 10 | 80919 | 80928 | LSC |
| *Delphinium elatum* var. *sericeum* | LHM1265 | 43 | p1 | (T)10 | 10 | 81555 | 81564 | LSC |
| *Delphinium elatum* var. *sericeum* | LHM1265 | 44 | p1 | (T)10 | 10 | 82365 | 82374 | LSC |
| *Delphinium elatum* var. *sericeum* | LHM1265 | 45 | p1 | (T)10 | 10 | 99334 | 99343 | IR |
| *Delphinium elatum* var. *sericeum* | LHM1265 | 46 | p1 | (T)10 | 10 | 108785 | 108794 | IR |
| *Delphinium elatum* var. *sericeum* | LHM1265 | 47 | p1 | (A)11 | 11 | 114145 | 114155 | SSC |
| *Delphinium elatum* var. *sericeum* | LHM1265 | 48 | p2 | (TA)7 | 14 | 117873 | 117886 | SSC |
| *Delphinium elatum* var. *sericeum* | LHM1265 | 49 | p1 | (T)11 | 11 | 118909 | 118919 | SSC |
| *Delphinium elatum* var. *sericeum* | LHM1265 | 50 | p1 | (T)10 | 10 | 123575 | 123584 | SSC |
| *Delphinium elatum* var. *sericeum* | LHM1265 | 51 | p1 | (T)10 | 10 | 124319 | 124328 | SSC |
| *Delphinium elatum* var. *sericeum* | LHM1265 | 52 | p1 | (T)13 | 13 | 125168 | 125180 | SSC |
| *Delphinium elatum* var. *sericeum* | LHM1265 | 53 | p1 | (T)12 | 12 | 125987 | 125976 | SSC |
| *Delphinium elatum* var. *sericeum* | LHM1265 | 54 | p1 | (T)10 | 10 | 126005 | 126014 | SSC |
| *Delphinium elatum* var. *sericeum* | LHM1265 | 55 | p1 | (T)11 | 11 | 126050 | 126060 | SSC |
| *Delphinium elatum* var. *sericeum* | LHM1265 | 56 | p1 | (A)10 | 10 | 130206 | 130215 | IR |
| *Delphinium elatum* var. *sericeum* | LHM1265 | 57 | p1 | (A)10 | 10 | 139657 | 139666 | IR |
| *Delphinium iliense* | LHM1285 | 1 | p1 | (A)11 | 11 | 6808 | 6818 | LSC |
| *Delphinium iliense* | LHM1285 | 2 | p1 | (T)10 | 10 | 7308 | 7317 | LSC |
| *Delphinium iliense* | LHM1285 | 3 | p1 | (T)17 | 17 | 7413 | 7429 | LSC |
| *Delphinium iliense* | LHM1285 | 4 | p1 | (T)10 | 10 | 7676 | 7685 | LSC |
| *Delphinium iliense* | LHM1285 | 5 | p1 | (T)11 | 11 | 10381 | 10391 | LSC |
| *Delphinium iliense* | LHM1285 | 6 | p1 | (T)10 | 10 | 10524 | 10533 | LSC |
| *Delphinium iliense* | LHM1285 | 7 | p1 | (T)14 | 14 | 16653 | 16666 | LSC |
| *Delphinium iliense* | LHM1285 | 8 | p1 | (T)11 | 11 | 20702 | 20712 | LSC |
| *Delphinium iliense* | LHM1285 | 9 | p1 | (T)10 | 10 | 24353 | 24362 | LSC |
| *Delphinium iliense* | LHM1285 | 10 | p3 | (AAT)5 | 15 | 26905 | 26919 | LSC |
| *Delphinium iliense* | LHM1285 | 11 | p1 | (A)13 | 13 | 27130 | 27142 | LSC |
| *Delphinium iliense* | LHM1285 | 12 | p1 | (A)11 | 11 | 27847 | 27857 | LSC |
| *Delphinium iliense* | LHM1285 | 13 | p1 | (T)12 | 12 | 28825 | 28836 | LSC |
| *Delphinium iliense* | LHM1285 | 14 | p1 | (A)11 | 11 | 29299 | 29309 | LSC |
| *Delphinium iliense* | LHM1285 | 15 | p1 | (A)14 | 14 | 35739 | 35752 | LSC |
| *Delphinium iliense* | LHM1285 | 16 | p1 | (A)10 | 10 | 35882 | 35891 | LSC |
| *Delphinium iliense* | LHM1285 | 17 | p1 | (C)10 | 10 | 39889 | 39898 | LSC |
| *Delphinium iliense* | LHM1285 | 18 | p1 | (T)14 | 14 | 41854 | 41867 | LSC |
| *Delphinium iliense* | LHM1285 | 19 | p1 | (A)10 | 10 | 42059 | 42068 | LSC |
| *Delphinium iliense* | LHM1285 | 20 | p1 | (A)11 | 11 | 44484 | 44494 | LSC |
| *Delphinium iliense* | LHM1285 | 21 | p1 | (A)12 | 12 | 46291 | 46302 | LSC |
| *Delphinium iliense* | LHM1285 | 22 | p1 | (A)10 | 10 | 51580 | 51589 | LSC |
| *Delphinium iliense* | LHM1285 | 23 | p1 | (T)10 | 10 | 52660 | 52669 | LSC |
| *Delphinium iliense* | LHM1285 | 24 | p1 | (A)14 | 14 | 54724 | 54737 | LSC |
| *Delphinium iliense* | LHM1285 | 25 | p1 | (A)11 | 11 | 59073 | 59083 | LSC |
| *Delphinium iliense* | LHM1285 | 26 | p1 | (T)10 | 10 | 59742 | 59751 | LSC |
| *Delphinium iliense* | LHM1285 | 27 | p1 | (T)11 | 11 | 63471 | 63481 | LSC |
| *Delphinium iliense* | LHM1285 | 28 | p1 | (T)11 | 11 | 63506 | 63516 | LSC |
| *Delphinium iliense* | LHM1285 | 29 | p1 | (T)10 | 10 | 64667 | 64676 | LSC |
| *Delphinium iliense* | LHM1285 | 30 | p1 | (T)10 | 10 | 65326 | 65335 | LSC |
| *Delphinium iliense* | LHM1285 | 31 | p1 | (A)11 | 11 | 65739 | 65749 | LSC |
| *Delphinium iliense* | LHM1285 | 32 | p1 | (T)10 | 10 | 66564 | 66573 | LSC |
| *Delphinium iliense* | LHM1285 | 33 | p2 | (TA)8 | 16 | 66742 | 66757 | LSC |
| *Delphinium iliense* | LHM1285 | 34 | p1 | (T)10 | 10 | 66840 | 66849 | LSC |
| *Delphinium iliense* | LHM1285 | 35 | p1 | (A)10 | 10 | 67366 | 67375 | LSC |
| *Delphinium iliense* | LHM1285 | 36 | p2 | (AT)7 | 14 | 67951 | 67964 | LSC |
| *Delphinium iliense* | LHM1285 | 37 | p1 | (T)11 | 11 | 68506 | 68516 | LSC |
| *Delphinium iliense* | LHM1285 | 38 | p1 | (A)14 | 14 | 70670 | 70683 | LSC |
| *Delphinium iliense* | LHM1285 | 39 | p1 | (T)10 | 10 | 71393 | 71402 | LSC |
| *Delphinium iliense* | LHM1285 | 40 | p1 | (T)12 | 12 | 80289 | 80300 | LSC |
| *Delphinium iliense* | LHM1285 | 41 | p1 | (T)11 | 11 | 80809 | 80819 | LSC |
| *Delphinium iliense* | LHM1285 | 42 | p1 | (T)10 | 10 | 99236 | 99245 | IR |
| *Delphinium iliense* | LHM1285 | 43 | p1 | (A)10 | 10 | 113782 | 113791 | SSC |
| *Delphinium iliense* | LHM1285 | 44 | p1 | (A)13 | 13 | 114045 | 114057 | SSC |
| *Delphinium iliense* | LHM1285 | 45 | p2 | (TA)6 | 12 | 118953 | 118964 | SSC |
| *Delphinium iliense* | LHM1285 | 46 | p1 | (T)11 | 11 | 123475 | 123485 | SSC |
| *Delphinium iliense* | LHM1285 | 47 | p1 | (A)10 | 10 | 123578 | 123587 | SSC |
| *Delphinium iliense* | LHM1285 | 48 | p1 | (T)10 | 10 | 124216 | 124225 | SSC |
| *Delphinium iliense* | LHM1285 | 49 | p1 | (T)13 | 13 | 125065 | 125077 | SSC |
| *Delphinium iliense* | LHM1285 | 50 | p1 | (T)12 | 12 | 125884 | 125895 | SSC |
| *Delphinium iliense* | LHM1285 | 51 | p1 | (T)11 | 11 | 126464 | 126474 | SSC |
| *Delphinium iliense* | LHM1285 | 52 | p1 | (A)10 | 10 | 139565 | 139574 | IR |
| *Delphinium maackianum* | NC_047293 | 1 | p1 | (A)11 | 11 | 4339 | 4349 | LSC |
| *Delphinium maackianum* | NC_047293 | 2 | p1 | (A)10 | 10 | 5704 | 5713 | LSC |
| *Delphinium maackianum* | NC_047293 | 3 | p1 | (A)14 | 14 | 6886 | 6899 | LSC |
| *Delphinium maackianum* | NC_047293 | 4 | p2 | (AT)6 | 12 | 7388 | 7399 | LSC |
| *Delphinium maackianum* | NC_047293 | 5 | p1 | (T)16 | 16 | 7502 | 7517 | LSC |
| *Delphinium maackianum* | NC_047293 | 6 | p1 | (T)11 | 11 | 10604 | 10614 | LSC |
| *Delphinium maackianum* | NC_047293 | 7 | p1 | (T)14 | 14 | 16770 | 16783 | LSC |
| *Delphinium maackianum* | NC_047293 | 8 | p1 | (T)10 | 10 | 24464 | 24473 | LSC |
| *Delphinium maackianum* | NC_047293 | 9 | p1 | (A)10 | 10 | 26066 | 26075 | LSC |
| *Delphinium maackianum* | NC_047293 | 10 | p1 | (A)12 | 12 | 26147 | 26158 | LSC |
| *Delphinium maackianum* | NC_047293 | 11 | p3 | (AAT)5 | 15 | 27119 | 27133 | LSC |
| *Delphinium maackianum* | NC_047293 | 12 | p1 | (A)14 | 14 | 27345 | 27358 | LSC |
| *Delphinium maackianum* | NC_047293 | 13 | p1 | (T)10 | 10 | 27495 | 27504 | LSC |
| *Delphinium maackianum* | NC_047293 | 14 | p1 | (T)11 | 11 | 29041 | 29051 | LSC |
| *Delphinium maackianum* | NC_047293 | 15 | p1 | (T)10 | 10 | 30827 | 30836 | LSC |
| *Delphinium maackianum* | NC_047293 | 16 | p2 | (TA)6 | 12 | 31893 | 31904 | LSC |
| *Delphinium maackianum* | NC_047293 | 17 | p1 | (A)10 | 10 | 32279 | 32288 | LSC |
| *Delphinium maackianum* | NC_047293 | 18 | p1 | (A)15 | 15 | 36039 | 36053 | LSC |
| *Delphinium maackianum* | NC_047293 | 19 | p1 | (A)11 | 11 | 36183 | 36193 | LSC |
| *Delphinium maackianum* | NC_047293 | 20 | p1 | (C)10 | 10 | 40191 | 40200 | LSC |
| *Delphinium maackianum* | NC_047293 | 21 | p1 | (T)13 | 13 | 42156 | 42168 | LSC |
| *Delphinium maackianum* | NC_047293 | 22 | p1 | (A)11 | 11 | 42359 | 42369 | LSC |
| *Delphinium maackianum* | NC_047293 | 23 | p1 | (A)10 | 10 | 43742 | 43751 | LSC |
| *Delphinium maackianum* | NC_047293 | 24 | p1 | (A)12 | 12 | 46608 | 46619 | LSC |
| *Delphinium maackianum* | NC_047293 | 25 | p1 | (T)11 | 11 | 49016 | 49026 | LSC |
| *Delphinium maackianum* | NC_047293 | 26 | p1 | (T)12 | 12 | 49710 | 49721 | LSC |
| *Delphinium maackianum* | NC_047293 | 27 | p1 | (T)10 | 10 | 52997 | 53006 | LSC |
| *Delphinium maackianum* | NC_047293 | 28 | p1 | (A)13 | 13 | 55061 | 55073 | LSC |
| *Delphinium maackianum* | NC_047293 | 29 | p1 | (A)11 | 11 | 59413 | 59423 | LSC |
| *Delphinium maackianum* | NC_047293 | 30 | p1 | (A)10 | 10 | 59792 | 59801 | LSC |
| *Delphinium maackianum* | NC_047293 | 31 | p1 | (A)10 | 10 | 61584 | 61593 | LSC |
| *Delphinium maackianum* | NC_047293 | 32 | p1 | (A)11 | 11 | 63611 | 63621 | LSC |
| *Delphinium maackianum* | NC_047293 | 33 | p1 | (A)10 | 10 | 66070 | 66079 | LSC |
| *Delphinium maackianum* | NC_047293 | 34 | p2 | (TA)11 | 22 | 67075 | 67096 | LSC |
| *Delphinium maackianum* | NC_047293 | 35 | p1 | (T)12 | 12 | 67150 | 67161 | LSC |
| *Delphinium maackianum* | NC_047293 | 36 | p1 | (A)22 | 22 | 67349 | 67370 | LSC |
| *Delphinium maackianum* | NC_047293 | 37 | p2 | (AT)7 | 14 | 68334 | 68347 | LSC |
| *Delphinium maackianum* | NC_047293 | 38 | p1 | (T)16 | 16 | 68889 | 68904 | LSC |
| *Delphinium maackianum* | NC_047293 | 39 | p1 | (A)10 | 10 | 71729 | 71738 | LSC |
| *Delphinium maackianum* | NC_047293 | 40 | p1 | (T)10 | 10 | 71782 | 71791 | LSC |
| *Delphinium maackianum* | NC_047293 | 41 | p1 | (A)10 | 10 | 75973 | 75982 | LSC |
| *Delphinium maackianum* | NC_047293 | 42 | p1 | (T)10 | 10 | 81182 | 81191 | LSC |
| *Delphinium maackianum* | NC_047293 | 43 | p1 | (T)10 | 10 | 81738 | 81747 | LSC |
| *Delphinium maackianum* | NC_047293 | 44 | p1 | (T)10 | 10 | 109063 | 109072 | IR |
| *Delphinium maackianum* | NC_047293 | 45 | p1 | (T)11 | 11 | 113855 | 113865 | SSC |
| *Delphinium maackianum* | NC_047293 | 46 | p1 | (A)12 | 12 | 114154 | 114165 | SSC |
| *Delphinium maackianum* | NC_047293 | 47 | p1 | (A)10 | 10 | 114419 | 114428 | SSC |
| *Delphinium maackianum* | NC_047293 | 48 | p2 | (TA)7 | 14 | 118147 | 118160 | SSC |
| *Delphinium maackianum* | NC_047293 | 49 | p1 | (T)11 | 11 | 119187 | 119197 | SSC |
| *Delphinium maackianum* | NC_047293 | 50 | p2 | (TA)8 | 16 | 119349 | 119364 | SSC |
| *Delphinium maackianum* | NC_047293 | 51 | p1 | (T)11 | 11 | 123850 | 123860 | SSC |
| *Delphinium maackianum* | NC_047293 | 52 | p1 | (T)10 | 10 | 124590 | 124599 | SSC |
| *Delphinium maackianum* | NC_047293 | 53 | p1 | (T)12 | 12 | 126258 | 126269 | SSC |
| *Delphinium maackianum* | NC_047293 | 54 | p1 | (T)10 | 10 | 126276 | 126285 | SSC |
| *Delphinium maackianum* | NC_047293 | 55 | p1 | (A)10 | 10 | 130468 | 130477 | IR |
| *Delphinium mollifolium* | LHM1295 | 1 | p1 | (A)12 | 12 | 6804 | 6815 | LSC |
| *Delphinium mollifolium* | LHM1295 | 2 | p1 | (T)12 | 12 | 7305 | 7316 | LSC |
| *Delphinium mollifolium* | LHM1295 | 3 | p1 | (T)17 | 17 | 7412 | 7428 | LSC |
| *Delphinium mollifolium* | LHM1295 | 4 | p1 | (T)10 | 10 | 7675 | 7684 | LSC |
| *Delphinium mollifolium* | LHM1295 | 5 | p1 | (T)11 | 11 | 10380 | 10390 | LSC |
| *Delphinium mollifolium* | LHM1295 | 6 | p1 | (T)10 | 10 | 10523 | 10532 | LSC |
| *Delphinium mollifolium* | LHM1295 | 7 | p1 | (T)14 | 14 | 16659 | 16672 | LSC |
| *Delphinium mollifolium* | LHM1295 | 8 | p1 | (T)11 | 11 | 20708 | 20718 | LSC |
| *Delphinium mollifolium* | LHM1295 | 9 | p1 | (T)10 | 10 | 24359 | 24368 | LSC |
| *Delphinium mollifolium* | LHM1295 | 10 | p1 | (A)11 | 11 | 25961 | 25971 | LSC |
| *Delphinium mollifolium* | LHM1295 | 11 | p1 | (A)13 | 13 | 27257 | 27269 | LSC |
| *Delphinium mollifolium* | LHM1295 | 12 | p1 | (A)11 | 11 | 27975 | 27985 | LSC |
| *Delphinium mollifolium* | LHM1295 | 13 | p1 | (T)12 | 12 | 28953 | 28964 | LSC |
| *Delphinium mollifolium* | LHM1295 | 14 | p1 | (A)10 | 10 | 29427 | 29436 | LSC |
| *Delphinium mollifolium* | LHM1295 | 15 | p1 | (A)10 | 10 | 32060 | 32069 | LSC |
| *Delphinium mollifolium* | LHM1295 | 16 | p1 | (A)14 | 14 | 35830 | 35843 | LSC |
| *Delphinium mollifolium* | LHM1295 | 17 | p1 | (A)10 | 10 | 35973 | 35982 | LSC |
| *Delphinium mollifolium* | LHM1295 | 18 | p1 | (C)10 | 10 | 39980 | 39989 | LSC |
| *Delphinium mollifolium* | LHM1295 | 19 | p1 | (T)14 | 14 | 41945 | 41958 | LSC |
| *Delphinium mollifolium* | LHM1295 | 20 | p1 | (A)11 | 11 | 42150 | 42160 | LSC |
| *Delphinium mollifolium* | LHM1295 | 21 | p1 | (A)12 | 12 | 44582 | 44593 | LSC |
| *Delphinium mollifolium* | LHM1295 | 22 | p1 | (A)12 | 12 | 46408 | 46419 | LSC |
| *Delphinium mollifolium* | LHM1295 | 23 | p1 | (A)11 | 11 | 51664 | 51674 | LSC |
| *Delphinium mollifolium* | LHM1295 | 24 | p1 | (T)10 | 10 | 52777 | 52786 | LSC |
| *Delphinium mollifolium* | LHM1295 | 25 | p1 | (A)13 | 13 | 54841 | 54853 | LSC |
| *Delphinium mollifolium* | LHM1295 | 26 | p1 | (A)11 | 11 | 59189 | 59199 | LSC |
| *Delphinium mollifolium* | LHM1295 | 27 | p1 | (T)11 | 11 | 59845 | 59855 | LSC |
| *Delphinium mollifolium* | LHM1295 | 28 | p1 | (A)10 | 10 | 63386 | 63395 | LSC |
| *Delphinium mollifolium* | LHM1295 | 29 | p1 | (T)11 | 11 | 63575 | 63585 | LSC |
| *Delphinium mollifolium* | LHM1295 | 30 | p1 | (T)11 | 11 | 63610 | 63620 | LSC |
| *Delphinium mollifolium* | LHM1295 | 31 | p1 | (T)10 | 10 | 64771 | 64780 | LSC |
| *Delphinium mollifolium* | LHM1295 | 32 | p1 | (T)10 | 10 | 65430 | 65439 | LSC |
| *Delphinium mollifolium* | LHM1295 | 33 | p1 | (A)12 | 12 | 65843 | 65854 | LSC |
| *Delphinium mollifolium* | LHM1295 | 34 | p1 | (T)10 | 10 | 66669 | 66678 | LSC |
| *Delphinium mollifolium* | LHM1295 | 35 | p2 | (TA)8 | 16 | 66847 | 66862 | LSC |
| *Delphinium mollifolium* | LHM1295 | 36 | p1 | (T)10 | 10 | 66951 | 66960 | LSC |
| *Delphinium mollifolium* | LHM1295 | 37 | p1 | (A)11 | 11 | 67088 | 67098 | LSC |
| *Delphinium mollifolium* | LHM1295 | 38 | p1 | (A)10 | 10 | 67479 | 67488 | LSC |
| *Delphinium mollifolium* | LHM1295 | 39 | p3 | (ATA)4 | 12 | 68058 | 68069 | LSC |
| *Delphinium mollifolium* | LHM1295 | 40 | p2 | (AT)6 | 12 | 68072 | 68083 | LSC |
| *Delphinium mollifolium* | LHM1295 | 41 | p1 | (T)10 | 10 | 68625 | 68634 | LSC |
| *Delphinium mollifolium* | LHM1295 | 42 | p1 | (A)14 | 14 | 70788 | 70801 | LSC |
| *Delphinium mollifolium* | LHM1295 | 43 | p1 | (T)10 | 10 | 80407 | 80416 | LSC |
| *Delphinium mollifolium* | LHM1295 | 44 | p1 | (T)13 | 13 | 80925 | 80937 | LSC |
| *Delphinium mollifolium* | LHM1295 | 45 | p1 | (T)10 | 10 | 81484 | 81493 | LSC |
| *Delphinium mollifolium* | LHM1295 | 46 | p1 | (T)10 | 10 | 99343 | 99352 | IR |
| *Delphinium mollifolium* | LHM1295 | 47 | p1 | (A)10 | 10 | 113889 | 113898 | SSC |
| *Delphinium mollifolium* | LHM1295 | 48 | p1 | (A)10 | 10 | 114152 | 114161 | SSC |
| *Delphinium mollifolium* | LHM1295 | 49 | p1 | (T)11 | 11 | 118909 | 118919 | SSC |
| *Delphinium mollifolium* | LHM1295 | 50 | p2 | (TA)6 | 12 | 119054 | 119065 | SSC |
| *Delphinium mollifolium* | LHM1295 | 51 | p1 | (T)12 | 12 | 123576 | 123587 | SSC |
| *Delphinium mollifolium* | LHM1295 | 52 | p1 | (A)10 | 10 | 123680 | 123689 | SSC |
| *Delphinium mollifolium* | LHM1295 | 53 | p1 | (T)10 | 10 | 124318 | 124327 | SSC |
| *Delphinium mollifolium* | LHM1295 | 54 | p1 | (T)13 | 13 | 125167 | 125179 | SSC |
| *Delphinium mollifolium* | LHM1295 | 55 | p1 | (T)12 | 12 | 125986 | 125997 | SSC |
| *Delphinium mollifolium* | LHM1295 | 56 | p1 | (T)11 | 11 | 126566 | 126576 | SSC |
| *Delphinium mollifolium* | LHM1295 | 57 | p1 | (A)10 | 10 | 139646 | 139655 | IR |
| *Delphinium naviculare* var. *lasiocarpum* | LHM1293 | 1 | p1 | (A)11 | 11 | 6813 | 6823 | LSC |
| *Delphinium naviculare* var. *lasiocarpum* | LHM1293 | 2 | p1 | (T)11 | 11 | 7313 | 7323 | LSC |
| *Delphinium naviculare* var. *lasiocarpum* | LHM1293 | 3 | p1 | (T)17 | 17 | 7419 | 7435 | LSC |
| *Delphinium naviculare* var. *lasiocarpum* | LHM1293 | 4 | p1 | (T)11 | 11 | 7682 | 7692 | LSC |
| *Delphinium naviculare* var. *lasiocarpum* | LHM1293 | 5 | p1 | (T)11 | 11 | 10388 | 10398 | LSC |
| *Delphinium naviculare* var. *lasiocarpum* | LHM1293 | 6 | p1 | (T)10 | 10 | 10531 | 10540 | LSC |
| *Delphinium naviculare* var. *lasiocarpum* | LHM1293 | 7 | p1 | (T)14 | 14 | 16667 | 16680 | LSC |
| *Delphinium naviculare* var. *lasiocarpum* | LHM1293 | 8 | p1 | (T)11 | 11 | 20716 | 20726 | LSC |
| *Delphinium naviculare* var. *lasiocarpum* | LHM1293 | 9 | p1 | (T)10 | 10 | 24367 | 24376 | LSC |
| *Delphinium naviculare* var. *lasiocarpum* | LHM1293 | 10 | p3 | (AAT)5 | 15 | 26919 | 26933 | LSC |
| *Delphinium naviculare* var. *lasiocarpum* | LHM1293 | 11 | p1 | (A)13 | 13 | 27144 | 27156 | LSC |
| *Delphinium naviculare* var. *lasiocarpum* | LHM1293 | 12 | p1 | (A)11 | 11 | 27861 | 27871 | LSC |
| *Delphinium naviculare* var. *lasiocarpum* | LHM1293 | 13 | p1 | (T)12 | 12 | 28839 | 28850 | LSC |
| *Delphinium naviculare* var. *lasiocarpum* | LHM1293 | 14 | p1 | (A)11 | 11 | 29313 | 29323 | LSC |
| *Delphinium naviculare* var. *lasiocarpum* | LHM1293 | 15 | p1 | (A)14 | 14 | 35753 | 35766 | LSC |
| *Delphinium naviculare* var. *lasiocarpum* | LHM1293 | 16 | p1 | (A)10 | 10 | 35896 | 35905 | LSC |
| *Delphinium naviculare* var. *lasiocarpum* | LHM1293 | 17 | p1 | (C)10 | 10 | 39903 | 39912 | LSC |
| *Delphinium naviculare* var. *lasiocarpum* | LHM1293 | 18 | p1 | (T)14 | 14 | 41868 | 41881 | LSC |
| *Delphinium naviculare* var. *lasiocarpum* | LHM1293 | 19 | p1 | (A)10 | 10 | 42073 | 42082 | LSC |
| *Delphinium naviculare* var. *lasiocarpum* | LHM1293 | 20 | p1 | (A)11 | 11 | 44498 | 44508 | LSC |
| *Delphinium naviculare* var. *lasiocarpum* | LHM1293 | 21 | p1 | (A)12 | 12 | 46305 | 46316 | LSC |
| *Delphinium naviculare* var. *lasiocarpum* | LHM1293 | 22 | p1 | (A)10 | 10 | 51376 | 51385 | LSC |
| *Delphinium naviculare* var. *lasiocarpum* | LHM1293 | 23 | p1 | (T)10 | 10 | 52674 | 52683 | LSC |
| *Delphinium naviculare* var. *lasiocarpum* | LHM1293 | 24 | p1 | (A)14 | 14 | 54738 | 54751 | LSC |
| *Delphinium naviculare* var. *lasiocarpum* | LHM1293 | 25 | p1 | (A)11 | 11 | 59087 | 59097 | LSC |
| *Delphinium naviculare* var. *lasiocarpum* | LHM1293 | 26 | p1 | (T)10 | 10 | 59756 | 59765 | LSC |
| *Delphinium naviculare* var. *lasiocarpum* | LHM1293 | 27 | p1 | (T)10 | 10 | 63485 | 63494 | LSC |
| *Delphinium naviculare* var. *lasiocarpum* | LHM1293 | 28 | p1 | (T)11 | 11 | 63519 | 63529 | LSC |
| *Delphinium naviculare* var. *lasiocarpum* | LHM1293 | 29 | p1 | (T)10 | 10 | 64680 | 64689 | LSC |
| *Delphinium naviculare* var. *lasiocarpum* | LHM1293 | 30 | p1 | (T)10 | 10 | 65339 | 65348 | LSC |
| *Delphinium naviculare* var. *lasiocarpum* | LHM1293 | 31 | p1 | (A)11 | 11 | 65752 | 65762 | LSC |
| *Delphinium naviculare* var. *lasiocarpum* | LHM1293 | 32 | p1 | (T)10 | 10 | 66577 | 66586 | LSC |
| *Delphinium naviculare* var. *lasiocarpum* | LHM1293 | 33 | p2 | (TA)8 | 16 | 66755 | 66770 | LSC |
| *Delphinium naviculare* var. *lasiocarpum* | LHM1293 | 34 | p1 | (T)12 | 12 | 66851 | 66862 | LSC |
| *Delphinium naviculare* var. *lasiocarpum* | LHM1293 | 35 | p1 | (A)10 | 10 | 67379 | 67388 | LSC |
| *Delphinium naviculare* var. *lasiocarpum* | LHM1293 | 36 | p2 | (AT)7 | 14 | 67964 | 67977 | LSC |
| *Delphinium naviculare* var. *lasiocarpum* | LHM1293 | 37 | p1 | (T)11 | 11 | 68519 | 68529 | LSC |
| *Delphinium naviculare* var. *lasiocarpum* | LHM1293 | 38 | p1 | (A)14 | 14 | 70683 | 70696 | LSC |
| *Delphinium naviculare* var. *lasiocarpum* | LHM1293 | 39 | p1 | (T)10 | 10 | 71406 | 71415 | LSC |
| *Delphinium naviculare* var. *lasiocarpum* | LHM1293 | 40 | p1 | (T)12 | 12 | 80301 | 80312 | LSC |
| *Delphinium naviculare* var. *lasiocarpum* | LHM1293 | 41 | p1 | (T)11 | 11 | 80821 | 80831 | LSC |
| *Delphinium naviculare* var. *lasiocarpum* | LHM1293 | 42 | p1 | (T)10 | 10 | 82274 | 82283 | LSC |
| *Delphinium naviculare* var. *lasiocarpum* | LHM1293 | 43 | p1 | (T)10 | 10 | 99244 | 99253 | IR |
| *Delphinium naviculare* var. *lasiocarpum* | LHM1293 | 44 | p1 | (A)10 | 10 | 113790 | 113799 | SSC |
| *Delphinium naviculare* var. *lasiocarpum* | LHM1293 | 45 | p1 | (A)15 | 15 | 114053 | 114067 | SSC |
| *Delphinium naviculare* var. *lasiocarpum* | LHM1293 | 46 | p2 | (TA)6 | 12 | 118963 | 118974 | SSC |
| *Delphinium naviculare* var. *lasiocarpum* | LHM1293 | 47 | p1 | (T)11 | 11 | 123485 | 123495 | SSC |
| *Delphinium naviculare* var. *lasiocarpum* | LHM1293 | 48 | p1 | (A)10 | 10 | 123588 | 123597 | SSC |
| *Delphinium naviculare* var. *lasiocarpum* | LHM1293 | 49 | p1 | (T)10 | 10 | 124226 | 124235 | SSC |
| *Delphinium naviculare* var. *lasiocarpum* | LHM1293 | 50 | p1 | (T)13 | 13 | 125075 | 125087 | SSC |
| *Delphinium naviculare* var. *lasiocarpum* | LHM1293 | 51 | p1 | (T)12 | 12 | 125894 | 125905 | SSC |
| *Delphinium naviculare* var. *lasiocarpum* | LHM1293 | 52 | p1 | (T)11 | 11 | 126474 | 126484 | SSC |
| *Delphinium naviculare* var. *lasiocarpum* | LHM1293 | 53 | p1 | (A)10 | 10 | 139575 | 139584 | IR |
| *Delphinium sauricum* | LHM1266 | 1 | p1 | (A)10 | 10 | 4490 | 4499 | LSC |
| *Delphinium sauricum* | LHM1266 | 2 | p1 | (A)12 | 12 | 6825 | 6836 | LSC |
| *Delphinium sauricum* | LHM1266 | 3 | p1 | (T)12 | 12 | 7326 | 7337 | LSC |
| *Delphinium sauricum* | LHM1266 | 4 | p1 | (T)17 | 17 | 7433 | 7449 | LSC |
| *Delphinium sauricum* | LHM1266 | 5 | p1 | (T)11 | 11 | 10400 | 10410 | LSC |
| *Delphinium sauricum* | LHM1266 | 6 | p1 | (T)10 | 10 | 10543 | 10552 | LSC |
| *Delphinium sauricum* | LHM1266 | 7 | p1 | (T)14 | 14 | 16679 | 16692 | LSC |
| *Delphinium sauricum* | LHM1266 | 8 | p1 | (T)11 | 11 | 20728 | 20738 | LSC |
| *Delphinium sauricum* | LHM1266 | 9 | p1 | (T)10 | 10 | 24380 | 24389 | LSC |
| *Delphinium sauricum* | LHM1266 | 10 | p1 | (A)10 | 10 | 25982 | 25991 | LSC |
| *Delphinium sauricum* | LHM1266 | 11 | p3 | (AAT)5 | 15 | 27036 | 27050 | LSC |
| *Delphinium sauricum* | LHM1266 | 12 | p1 | (A)13 | 13 | 27261 | 27273 | LSC |
| *Delphinium sauricum* | LHM1266 | 13 | p1 | (A)11 | 11 | 27979 | 27989 | LSC |
| *Delphinium sauricum* | LHM1266 | 14 | p1 | (T)11 | 11 | 28957 | 28967 | LSC |
| *Delphinium sauricum* | LHM1266 | 15 | p1 | (A)12 | 12 | 32062 | 32073 | LSC |
| *Delphinium sauricum* | LHM1266 | 16 | p1 | (A)11 | 11 | 35834 | 35844 | LSC |
| *Delphinium sauricum* | LHM1266 | 17 | p1 | (A)10 | 10 | 35974 | 35983 | LSC |
| *Delphinium sauricum* | LHM1266 | 18 | p1 | (C)10 | 10 | 39981 | 39990 | LSC |
| *Delphinium sauricum* | LHM1266 | 19 | p1 | (T)15 | 15 | 41946 | 41960 | LSC |
| *Delphinium sauricum* | LHM1266 | 20 | p1 | (A)10 | 10 | 44576 | 44585 | LSC |
| *Delphinium sauricum* | LHM1266 | 21 | p1 | (A)13 | 13 | 46385 | 46397 | LSC |
| *Delphinium sauricum* | LHM1266 | 22 | p1 | (A)11 | 11 | 51642 | 51652 | LSC |
| *Delphinium sauricum* | LHM1266 | 23 | p1 | (A)15 | 15 | 54818 | 54832 | LSC |
| *Delphinium sauricum* | LHM1266 | 24 | p1 | (A)11 | 11 | 59168 | 59178 | LSC |
| *Delphinium sauricum* | LHM1266 | 25 | p1 | (A)12 | 12 | 63372 | 63383 | LSC |
| *Delphinium sauricum* | LHM1266 | 26 | p1 | (T)11 | 11 | 63563 | 63573 | LSC |
| *Delphinium sauricum* | LHM1266 | 27 | p1 | (T)11 | 11 | 63598 | 63608 | LSC |
| *Delphinium sauricum* | LHM1266 | 28 | p1 | (T)10 | 10 | 65417 | 65426 | LSC |
| *Delphinium sauricum* | LHM1266 | 29 | p1 | (A)11 | 11 | 65830 | 65840 | LSC |
| *Delphinium sauricum* | LHM1266 | 30 | p1 | (T)11 | 11 | 66655 | 66665 | LSC |
| *Delphinium sauricum* | LHM1266 | 31 | p2 | (TA)8 | 16 | 66834 | 66849 | LSC |
| *Delphinium sauricum* | LHM1266 | 32 | p1 | (T)10 | 10 | 66954 | 66963 | LSC |
| *Delphinium sauricum* | LHM1266 | 33 | p1 | (A)11 | 11 | 67097 | 67107 | LSC |
| *Delphinium sauricum* | LHM1266 | 34 | p1 | (A)10 | 10 | 67488 | 67497 | LSC |
| *Delphinium sauricum* | LHM1266 | 35 | p2 | (AT)8 | 16 | 68073 | 68088 | LSC |
| *Delphinium sauricum* | LHM1266 | 36 | p1 | (T)10 | 10 | 70108 | 70117 | LSC |
| *Delphinium sauricum* | LHM1266 | 37 | p1 | (A)14 | 14 | 70793 | 70806 | LSC |
| *Delphinium sauricum* | LHM1266 | 38 | p1 | (T)10 | 10 | 80414 | 80423 | LSC |
| *Delphinium sauricum* | LHM1266 | 39 | p1 | (T)11 | 11 | 80932 | 80942 | LSC |
| *Delphinium sauricum* | LHM1266 | 40 | p1 | (T)10 | 10 | 81489 | 81498 | LSC |
| *Delphinium sauricum* | LHM1266 | 41 | p1 | (T)10 | 10 | 99353 | 99362 | IR |
| *Delphinium sauricum* | LHM1266 | 42 | p1 | (A)10 | 10 | 113900 | 113909 | SSC |
| *Delphinium sauricum* | LHM1266 | 43 | p1 | (A)12 | 12 | 114163 | 114174 | SSC |
| *Delphinium sauricum* | LHM1266 | 44 | p1 | (T)11 | 11 | 118922 | 118932 | SSC |
| *Delphinium sauricum* | LHM1266 | 45 | p2 | (TA)6 | 12 | 119067 | 119078 | SSC |
| *Delphinium sauricum* | LHM1266 | 46 | p1 | (T)12 | 12 | 123589 | 123600 | SSC |
| *Delphinium sauricum* | LHM1266 | 47 | p1 | (A)10 | 10 | 123693 | 123702 | SSC |
| *Delphinium sauricum* | LHM1266 | 48 | p1 | (T)10 | 10 | 124331 | 124340 | SSC |
| *Delphinium sauricum* | LHM1266 | 49 | p1 | (T)13 | 13 | 125180 | 125192 | SSC |
| *Delphinium sauricum* | LHM1266 | 50 | p1 | (T)12 | 12 | 125999 | 126010 | SSC |
| *Delphinium sauricum* | LHM1266 | 51 | p1 | (T)11 | 11 | 126579 | 126589 | SSC |
| *Delphinium sauricum* | LHM1266 | 52 | p1 | (A)10 | 10 | 139659 | 139668 | IR |
| *Delphinium shawurense* | LHM1271 | 1 | p1 | (A)11 | 11 | 4319 | 4329 | LSC |
| *Delphinium shawurense* | LHM1271 | 2 | p1 | (A)10 | 10 | 4531 | 4540 | LSC |
| *Delphinium shawurense* | LHM1271 | 3 | p1 | (T)11 | 11 | 4788 | 4798 | LSC |
| *Delphinium shawurense* | LHM1271 | 4 | p1 | (A)11 | 11 | 6851 | 6861 | LSC |
| *Delphinium shawurense* | LHM1271 | 5 | p1 | (T)10 | 10 | 7353 | 7362 | LSC |
| *Delphinium shawurense* | LHM1271 | 6 | p1 | (T)15 | 15 | 7458 | 7472 | LSC |
| *Delphinium shawurense* | LHM1271 | 7 | p1 | (T)11 | 11 | 10549 | 10559 | LSC |
| *Delphinium shawurense* | LHM1271 | 8 | p1 | (T)14 | 14 | 16700 | 16713 | LSC |
| *Delphinium shawurense* | LHM1271 | 9 | p1 | (T)10 | 10 | 24399 | 24408 | LSC |
| *Delphinium shawurense* | LHM1271 | 10 | p3 | (AAT)5 | 15 | 27033 | 27047 | LSC |
| *Delphinium shawurense* | LHM1271 | 11 | p1 | (T)10 | 10 | 27408 | 27417 | LSC |
| *Delphinium shawurense* | LHM1271 | 12 | p1 | (A)11 | 11 | 27976 | 27986 | LSC |
| *Delphinium shawurense* | LHM1271 | 13 | p1 | (T)13 | 13 | 28957 | 28969 | LSC |
| *Delphinium shawurense* | LHM1271 | 14 | p1 | (T)10 | 10 | 30750 | 30759 | LSC |
| *Delphinium shawurense* | LHM1271 | 15 | p1 | (A)11 | 11 | 32105 | 32115 | LSC |
| *Delphinium shawurense* | LHM1271 | 16 | p1 | (A)15 | 15 | 35867 | 35881 | LSC |
| *Delphinium shawurense* | LHM1271 | 17 | p1 | (A)10 | 10 | 36011 | 36020 | LSC |
| *Delphinium shawurense* | LHM1271 | 18 | p1 | (C)10 | 10 | 40018 | 40027 | LSC |
| *Delphinium shawurense* | LHM1271 | 19 | p1 | (T)12 | 12 | 41978 | 41989 | LSC |
| *Delphinium shawurense* | LHM1271 | 20 | p1 | (A)12 | 12 | 46421 | 46432 | LSC |
| *Delphinium shawurense* | LHM1271 | 21 | p1 | (A)10 | 10 | 51472 | 51481 | LSC |
| *Delphinium shawurense* | LHM1271 | 22 | p1 | (A)10 | 10 | 51690 | 51699 | LSC |
| *Delphinium shawurense* | LHM1271 | 23 | p1 | (T)13 | 13 | 52774 | 52786 | LSC |
| *Delphinium shawurense* | LHM1271 | 24 | p1 | (A)12 | 12 | 54841 | 54852 | LSC |
| *Delphinium shawurense* | LHM1271 | 25 | p1 | (A)11 | 11 | 59197 | 59207 | LSC |
| *Delphinium shawurense* | LHM1271 | 26 | p1 | (A)11 | 11 | 59573 | 59583 | LSC |
| *Delphinium shawurense* | LHM1271 | 27 | p1 | (T)11 | 11 | 59863 | 59873 | LSC |
| *Delphinium shawurense* | LHM1271 | 28 | p1 | (T)11 | 11 | 63593 | 63603 | LSC |
| *Delphinium shawurense* | LHM1271 | 29 | p1 | (T)16 | 16 | 63624 | 63639 | LSC |
| *Delphinium shawurense* | LHM1271 | 30 | p1 | (A)11 | 11 | 65857 | 65867 | LSC |
| *Delphinium shawurense* | LHM1271 | 31 | p1 | (T)16 | 16 | 66154 | 66169 | LSC |
| *Delphinium shawurense* | LHM1271 | 32 | p2 | (TA)8 | 16 | 66889 | 66904 | LSC |
| *Delphinium shawurense* | LHM1271 | 33 | p1 | (T)11 | 11 | 66954 | 66964 | LSC |
| *Delphinium shawurense* | LHM1271 | 34 | p1 | (T)10 | 10 | 70150 | 70159 | LSC |
| *Delphinium shawurense* | LHM1271 | 35 | p1 | (A)11 | 11 | 70835 | 70845 | LSC |
| *Delphinium shawurense* | LHM1271 | 36 | p1 | (T)11 | 11 | 71557 | 71567 | LSC |
| *Delphinium shawurense* | LHM1271 | 37 | p1 | (T)11 | 11 | 80449 | 80459 | LSC |
| *Delphinium shawurense* | LHM1271 | 38 | p1 | (T)10 | 10 | 80968 | 80977 | LSC |
| *Delphinium shawurense* | LHM1271 | 39 | p1 | (T)11 | 11 | 82417 | 82427 | LSC |
| *Delphinium shawurense* | LHM1271 | 40 | p1 | (T)10 | 10 | 99374 | 99383 | IR |
| *Delphinium shawurense* | LHM1271 | 41 | p1 | (T)10 | 10 | 108825 | 108834 | IR |
| *Delphinium shawurense* | LHM1271 | 42 | p1 | (T)10 | 10 | 113617 | 113626 | SSC |
| *Delphinium shawurense* | LHM1271 | 43 | p1 | (A)15 | 15 | 114191 | 114205 | SSC |
| *Delphinium shawurense* | LHM1271 | 44 | p2 | (TA)7 | 14 | 117930 | 117943 | SSC |
| *Delphinium shawurense* | LHM1271 | 45 | p1 | (T)11 | 11 | 118966 | 118976 | SSC |
| *Delphinium shawurense* | LHM1271 | 46 | p1 | (T)11 | 11 | 123632 | 123642 | SSC |
| *Delphinium shawurense* | LHM1271 | 47 | p1 | (T)10 | 10 | 124384 | 124393 | SSC |
| *Delphinium shawurense* | LHM1271 | 48 | p1 | (T)13 | 13 | 125233 | 125245 | SSC |
| *Delphinium shawurense* | LHM1271 | 49 | p1 | (T)12 | 12 | 126052 | 126063 | SSC |
| *Delphinium shawurense* | LHM1271 | 50 | p1 | (T)10 | 10 | 126070 | 126079 | SSC |
| *Delphinium shawurense* | LHM1271 | 51 | p1 | (T)11 | 11 | 126115 | 126125 | SSC |
| *Delphinium shawurense* | LHM1271 | 52 | p1 | (A)10 | 10 | 130271 | 130280 | IR |
| *Delphinium shawurense* | LHM1271 | 53 | p1 | (A)10 | 10 | 139722 | 139731 | IR |
| *Delphinium winklerianum* | LHM1299 | 1 | p1 | (A)14 | 14 | 6804 | 6817 | LSC |
| *Delphinium winklerianum* | LHM1299 | 2 | p1 | (T)11 | 11 | 7307 | 7317 | LSC |
| *Delphinium winklerianum* | LHM1299 | 3 | p1 | (T)15 | 15 | 7413 | 7427 | LSC |
| *Delphinium winklerianum* | LHM1299 | 4 | p1 | (T)10 | 10 | 7674 | 7683 | LSC |
| *Delphinium winklerianum* | LHM1299 | 5 | p1 | (T)10 | 10 | 10379 | 10388 | LSC |
| *Delphinium winklerianum* | LHM1299 | 6 | p1 | (T)11 | 11 | 10521 | 10531 | LSC |
| *Delphinium winklerianum* | LHM1299 | 7 | p1 | (T)14 | 14 | 16658 | 16671 | LSC |
| *Delphinium winklerianum* | LHM1299 | 8 | p1 | (T)11 | 11 | 20707 | 20717 | LSC |
| *Delphinium winklerianum* | LHM1299 | 9 | p1 | (T)10 | 10 | 24358 | 24367 | LSC |
| *Delphinium winklerianum* | LHM1299 | 10 | p3 | (AAT)5 | 15 | 27014 | 27028 | LSC |
| *Delphinium winklerianum* | LHM1299 | 11 | p1 | (A)12 | 12 | 27239 | 27250 | LSC |
| *Delphinium winklerianum* | LHM1299 | 12 | p1 | (A)11 | 11 | 27956 | 27966 | LSC |
| *Delphinium winklerianum* | LHM1299 | 13 | p1 | (T)13 | 13 | 28934 | 28946 | LSC |
| *Delphinium winklerianum* | LHM1299 | 14 | p1 | (A)10 | 10 | 29416 | 29425 | LSC |
| *Delphinium winklerianum* | LHM1299 | 15 | p1 | (T)10 | 10 | 30326 | 30335 | LSC |
| *Delphinium winklerianum* | LHM1299 | 16 | p1 | (A)10 | 10 | 32050 | 32059 | LSC |
| *Delphinium winklerianum* | LHM1299 | 17 | p1 | (A)14 | 14 | 35819 | 35832 | LSC |
| *Delphinium winklerianum* | LHM1299 | 18 | p1 | (A)10 | 10 | 35962 | 35971 | LSC |
| *Delphinium winklerianum* | LHM1299 | 19 | p1 | (C)10 | 10 | 39969 | 39978 | LSC |
| *Delphinium winklerianum* | LHM1299 | 20 | p1 | (T)14 | 14 | 41934 | 41947 | LSC |
| *Delphinium winklerianum* | LHM1299 | 21 | p1 | (A)10 | 10 | 42139 | 42148 | LSC |
| *Delphinium winklerianum* | LHM1299 | 22 | p1 | (A)10 | 10 | 44557 | 44566 | LSC |
| *Delphinium winklerianum* | LHM1299 | 23 | p1 | (A)13 | 13 | 46367 | 46379 | LSC |
| *Delphinium winklerianum* | LHM1299 | 24 | p1 | (A)11 | 11 | 47149 | 47159 | LSC |
| *Delphinium winklerianum* | LHM1299 | 25 | p1 | (A)10 | 10 | 51432 | 51441 | LSC |
| *Delphinium winklerianum* | LHM1299 | 26 | p1 | (A)11 | 11 | 51618 | 51628 | LSC |
| *Delphinium winklerianum* | LHM1299 | 27 | p1 | (A)14 | 14 | 54794 | 54807 | LSC |
| *Delphinium winklerianum* | LHM1299 | 28 | p1 | (A)11 | 11 | 59143 | 59153 | LSC |
| *Delphinium winklerianum* | LHM1299 | 29 | p1 | (T)10 | 10 | 59812 | 59821 | LSC |
| *Delphinium winklerianum* | LHM1299 | 30 | p1 | (A)10 | 10 | 63352 | 63361 | LSC |
| *Delphinium winklerianum* | LHM1299 | 31 | p1 | (T)10 | 10 | 63541 | 63550 | LSC |
| *Delphinium winklerianum* | LHM1299 | 32 | p1 | (T)13 | 13 | 63575 | 63587 | LSC |
| *Delphinium winklerianum* | LHM1299 | 33 | p1 | (T)10 | 10 | 64738 | 64747 | LSC |
| *Delphinium winklerianum* | LHM1299 | 34 | p1 | (T)10 | 10 | 65397 | 65406 | LSC |
| *Delphinium winklerianum* | LHM1299 | 35 | p1 | (A)12 | 12 | 65810 | 65821 | LSC |
| *Delphinium winklerianum* | LHM1299 | 36 | p1 | (T)10 | 10 | 66640 | 66649 | LSC |
| *Delphinium winklerianum* | LHM1299 | 37 | p2 | (TA)8 | 16 | 66818 | 66833 | LSC |
| *Delphinium winklerianum* | LHM1299 | 38 | p1 | (A)12 | 12 | 67086 | 67097 | LSC |
| *Delphinium winklerianum* | LHM1299 | 39 | p1 | (A)10 | 10 | 67478 | 67487 | LSC |
| *Delphinium winklerianum* | LHM1299 | 40 | p2 | (AT)7 | 14 | 68063 | 68076 | LSC |
| *Delphinium winklerianum* | LHM1299 | 41 | p1 | (A)15 | 15 | 70785 | 70799 | LSC |
| *Delphinium winklerianum* | LHM1299 | 42 | p1 | (A)10 | 10 | 71457 | 71466 | LSC |
| *Delphinium winklerianum* | LHM1299 | 43 | p1 | (T)10 | 10 | 80406 | 80415 | LSC |
| *Delphinium winklerianum* | LHM1299 | 44 | p1 | (T)12 | 12 | 80924 | 80935 | LSC |
| *Delphinium winklerianum* | LHM1299 | 45 | p1 | (T)10 | 10 | 99342 | 99351 | IR |
| *Delphinium winklerianum* | LHM1299 | 46 | p1 | (A)10 | 10 | 113889 | 113898 | SSC |
| *Delphinium winklerianum* | LHM1299 | 47 | p1 | (A)16 | 16 | 114152 | 114167 | SSC |
| *Delphinium winklerianum* | LHM1299 | 48 | p1 | (T)11 | 11 | 118915 | 118925 | SSC |
| *Delphinium winklerianum* | LHM1299 | 49 | p2 | (TA)6 | 12 | 119060 | 119071 | SSC |
| *Delphinium winklerianum* | LHM1299 | 50 | p1 | (T)12 | 12 | 123582 | 123593 | SSC |
| *Delphinium winklerianum* | LHM1299 | 51 | p1 | (T)10 | 10 | 124323 | 124332 | SSC |
| *Delphinium winklerianum* | LHM1299 | 52 | p1 | (T)13 | 13 | 125172 | 125184 | SSC |
| *Delphinium winklerianum* | LHM1299 | 53 | p1 | (T)12 | 12 | 125991 | 126002 | SSC |
| *Delphinium winklerianum* | LHM1299 | 54 | p1 | (T)10 | 10 | 126009 | 126018 | SSC |
| *Delphinium winklerianum* | LHM1299 | 55 | p1 | (T)11 | 11 | 126571 | 126581 | SSC |
| *Delphinium winklerianum* | LHM1299 | 56 | p1 | (A)10 | 10 | 139639 | 139648 | IR |
| *Delphinium yunnanense* | MW246156 | 1 | p1 | (A)11 | 11 | 4317 | 4327 | LSC |
| *Delphinium yunnanense* | MW246156 | 2 | p1 | (A)10 | 10 | 4532 | 4541 | LSC |
| *Delphinium yunnanense* | MW246156 | 3 | p1 | (A)14 | 14 | 6858 | 6871 | LSC |
| *Delphinium yunnanense* | MW246156 | 4 | p1 | (T)11 | 11 | 7367 | 7377 | LSC |
| *Delphinium yunnanense* | MW246156 | 5 | p1 | (T)10 | 10 | 7474 | 7483 | LSC |
| *Delphinium yunnanense* | MW246156 | 6 | p1 | (T)14 | 14 | 16700 | 16713 | LSC |
| *Delphinium yunnanense* | MW246156 | 7 | p1 | (T)10 | 10 | 20748 | 20757 | LSC |
| *Delphinium yunnanense* | MW246156 | 8 | p1 | (T)10 | 10 | 24398 | 24407 | LSC |
| *Delphinium yunnanense* | MW246156 | 9 | p3 | (AAT)5 | 15 | 26989 | 27003 | LSC |
| *Delphinium yunnanense* | MW246156 | 10 | p1 | (A)12 | 12 | 27217 | 27228 | LSC |
| *Delphinium yunnanense* | MW246156 | 11 | p1 | (A)10 | 10 | 27933 | 27942 | LSC |
| *Delphinium yunnanense* | MW246156 | 12 | p1 | (T)11 | 11 | 28912 | 28922 | LSC |
| *Delphinium yunnanense* | MW246156 | 13 | p1 | (A)10 | 10 | 29367 | 29376 | LSC |
| *Delphinium yunnanense* | MW246156 | 14 | p1 | (T)10 | 10 | 30683 | 30692 | LSC |
| *Delphinium yunnanense* | MW246156 | 15 | p2 | (TA)6 | 12 | 31748 | 31759 | LSC |
| *Delphinium yunnanense* | MW246156 | 16 | p1 | (A)10 | 10 | 35889 | 35898 | LSC |
| *Delphinium yunnanense* | MW246156 | 17 | p1 | (A)10 | 10 | 36028 | 36037 | LSC |
| *Delphinium yunnanense* | MW246156 | 18 | p1 | (C)10 | 10 | 40035 | 40044 | LSC |
| *Delphinium yunnanense* | MW246156 | 19 | p1 | (T)12 | 12 | 41995 | 42006 | LSC |
| *Delphinium yunnanense* | MW246156 | 20 | p1 | (A)12 | 12 | 42198 | 42209 | LSC |
| *Delphinium yunnanense* | MW246156 | 21 | p1 | (A)12 | 12 | 43576 | 43587 | LSC |
| *Delphinium yunnanense* | MW246156 | 22 | p1 | (A)10 | 10 | 46436 | 46445 | LSC |
| *Delphinium yunnanense* | MW246156 | 23 | p1 | (T)11 | 11 | 48838 | 48848 | LSC |
| *Delphinium yunnanense* | MW246156 | 24 | p1 | (A)10 | 10 | 51711 | 51720 | LSC |
| *Delphinium yunnanense* | MW246156 | 25 | p1 | (A)11 | 11 | 54848 | 54858 | LSC |
| *Delphinium yunnanense* | MW246156 | 26 | p1 | (A)12 | 12 | 59181 | 59192 | LSC |
| *Delphinium yunnanense* | MW246156 | 27 | p1 | (A)15 | 15 | 59561 | 59575 | LSC |
| *Delphinium yunnanense* | MW246156 | 28 | p1 | (T)10 | 10 | 59855 | 59864 | LSC |
| *Delphinium yunnanense* | MW246156 | 29 | p1 | (T)11 | 11 | 60084 | 60094 | LSC |
| *Delphinium yunnanense* | MW246156 | 30 | p1 | (A)11 | 11 | 63343 | 63353 | LSC |
| *Delphinium yunnanense* | MW246156 | 31 | p1 | (T)11 | 11 | 63540 | 63550 | LSC |
| *Delphinium yunnanense* | MW246156 | 32 | p1 | (T)10 | 10 | 63574 | 63583 | LSC |
| *Delphinium yunnanense* | MW246156 | 33 | p1 | (A)10 | 10 | 65549 | 65558 | LSC |
| *Delphinium yunnanense* | MW246156 | 34 | p1 | (A)10 | 10 | 65792 | 65801 | LSC |
| *Delphinium yunnanense* | MW246156 | 35 | p1 | (T)11 | 11 | 66856 | 66866 | LSC |
| *Delphinium yunnanense* | MW246156 | 36 | p1 | (A)15 | 15 | 66993 | 67007 | LSC |
| *Delphinium yunnanense* | MW246156 | 37 | p2 | (AT)7 | 14 | 67973 | 67986 | LSC |
| *Delphinium yunnanense* | MW246156 | 38 | p1 | (T)10 | 10 | 68529 | 68538 | LSC |
| *Delphinium yunnanense* | MW246156 | 39 | p1 | (A)11 | 11 | 70676 | 70686 | LSC |
| *Delphinium yunnanense* | MW246156 | 40 | p1 | (T)10 | 10 | 71417 | 71426 | LSC |
| *Delphinium yunnanense* | MW246156 | 41 | p1 | (T)10 | 10 | 78090 | 78099 | LSC |
| *Delphinium yunnanense* | MW246156 | 42 | p1 | (T)13 | 13 | 82214 | 82226 | LSC |
| *Delphinium yunnanense* | MW246156 | 43 | p1 | (T)10 | 10 | 99186 | 99195 | IR |
| *Delphinium yunnanense* | MW246156 | 44 | p1 | (T)12 | 12 | 108631 | 108642 | IR |
| *Delphinium yunnanense* | MW246156 | 45 | p1 | (T)10 | 10 | 113425 | 113434 | SSC |
| *Delphinium yunnanense* | MW246156 | 46 | p1 | (A)12 | 12 | 113998 | 114009 | SSC |
| *Delphinium yunnanense* | MW246156 | 47 | p1 | (T)13 | 13 | 115593 | 115605 | SSC |
| *Delphinium yunnanense* | MW246156 | 48 | p2 | (TA)7 | 14 | 117729 | 117742 | SSC |
| *Delphinium yunnanense* | MW246156 | 49 | p1 | (T)10 | 10 | 118765 | 118774 | SSC |
| *Delphinium yunnanense* | MW246156 | 50 | p1 | (T)14 | 14 | 123449 | 123462 | SSC |
| *Delphinium yunnanense* | MW246156 | 51 | p1 | (A)11 | 11 | 123555 | 123565 | SSC |
| *Delphinium yunnanense* | MW246156 | 52 | p1 | (T)10 | 10 | 124194 | 124203 | SSC |
| *Delphinium yunnanense* | MW246156 | 53 | p1 | (T)12 | 12 | 125862 | 125873 | SSC |
| *Delphinium yunnanense* | MW246156 | 54 | p1 | (T)10 | 10 | 125880 | 125889 | SSC |
| *Delphinium yunnanense* | MW246156 | 55 | p1 | (A)12 | 12 | 130051 | 130062 | IR |
| *Delphinium yunnanense* | MW246156 | 56 | p1 | (A)10 | 10 | 139498 | 139507 | IR |
