# Supplementary material for: Complete chloroplast genomes of eight Delphinium taxa (Ranunculaceae) endemic to Xinjiang, China: insights into genome structure, comparative analysis, and phylogenetic relationships
Source: BMC Plant Biol. 2024 Jun 26;24:600. doi: 10.1186/s12870-024-05279-y (PMC11201361; doi:10.1186/s12870-024-05279-y)
Supplement: Supplementary file 3 — Supplementary Material 3 [file 12870_2024_5279_MOESM3_ESM.docx]

**TABLE S3** Comparison of dispersed repeats among 14 *Delphinium* taxa chloroplast genomes.

| **Taxon** | **ID** | **Repeat length of the first part** | **Starting position of the first part** | **Match direction** | **Repeat length of the second part** | **Starting position of the second part** | **Distance of this repeat** | **Calculated e-value of this repeat** |
| --- | --- | --- | --- | --- | --- | --- | --- | --- |
| *Delphinium aemulans* | LHM1280 | 26561 | 84809 | P | 26561 | 127684 | 0 | 0.00E+00 |
| *Delphinium aemulans* | LHM1280 | 74 | 63706 | P | 74 | 63706 | 0 | 1.88E-35 |
| *Delphinium aemulans* | LHM1280 | 57 | 74602 | P | 57 | 74602 | -3 | 2.55E-19 |
| *Delphinium aemulans* | LHM1280 | 52 | 38402 | F | 52 | 40626 | -3 | 1.97E-16 |
| *Delphinium aemulans* | LHM1280 | 42 | 93579 | P | 42 | 93579 | 0 | 3.46E-16 |
| *Delphinium aemulans* | LHM1280 | 42 | 93579 | F | 42 | 145433 | 0 | 3.46E-16 |
| *Delphinium aemulans* | LHM1280 | 42 | 145433 | P | 42 | 145433 | 0 | 3.46E-16 |
| *Delphinium aemulans* | LHM1280 | 48 | 78126 | P | 48 | 78126 | -2 | 8.57E-16 |
| *Delphinium aemulans* | LHM1280 | 39 | 43359 | F | 39 | 98988 | 0 | 2.21E-14 |
| *Delphinium aemulans* | LHM1280 | 39 | 43359 | P | 39 | 140027 | 0 | 2.21E-14 |
| *Delphinium aemulans* | LHM1280 | 30 | 6315 | P | 30 | 45090 | -1 | 5.22E-07 |
| *Delphinium aemulans* | LHM1280 | 32 | 6262 | P | 32 | 6262 | -2 | 1.62E-06 |
| *Delphinium aemulans* | LHM1280 | 30 | 4444 | P | 30 | 4444 | -2 | 2.27E-05 |
| *Delphinium aemulans* | LHM1280 | 30 | 6312 | F | 30 | 35186 | -2 | 2.27E-05 |
| *Delphinium aemulans* | LHM1280 | 31 | 91548 | F | 31 | 91566 | -3 | 1.76E-04 |
| *Delphinium aemulans* | LHM1280 | 31 | 91548 | P | 31 | 147457 | -3 | 1.76E-04 |
| *Delphinium aemulans* | LHM1280 | 31 | 91566 | P | 31 | 147475 | -3 | 1.76E-04 |
| *Delphinium aemulans* | LHM1280 | 31 | 147457 | F | 31 | 147475 | -3 | 1.76E-04 |
| *Delphinium anthriscifolium* | MK253461 | 25977 | 85871 | P | 25977 | 129100 | 0 | 0.00E+00 |
| *Delphinium anthriscifolium* | MK253461 | 62 | 64579 | P | 62 | 64579 | -2 | 5.41E-24 |
| *Delphinium anthriscifolium* | MK253461 | 48 | 79135 | P | 48 | 79135 | 0 | 8.54E-20 |
| *Delphinium anthriscifolium* | MK253461 | 57 | 75611 | P | 57 | 75611 | -3 | 2.57E-19 |
| *Delphinium anthriscifolium* | MK253461 | 52 | 38999 | F | 52 | 41223 | -3 | 1.99E-16 |
| *Delphinium anthriscifolium* | MK253461 | 42 | 94649 | P | 42 | 94649 | 0 | 3.50E-16 |
| *Delphinium anthriscifolium* | MK253461 | 42 | 94649 | F | 42 | 146257 | 0 | 3.50E-16 |
| *Delphinium anthriscifolium* | MK253461 | 42 | 146257 | P | 42 | 146257 | 0 | 3.50E-16 |
| *Delphinium anthriscifolium* | MK253461 | 39 | 43995 | F | 39 | 100078 | 0 | 2.24E-14 |
| *Delphinium anthriscifolium* | MK253461 | 39 | 43995 | P | 39 | 140831 | 0 | 2.24E-14 |
| *Delphinium anthriscifolium* | MK253461 | 33 | 67765 | F | 33 | 67794 | 0 | 9.17E-11 |
| *Delphinium anthriscifolium* | MK253461 | 30 | 4477 | P | 30 | 4477 | 0 | 5.87E-09 |
| *Delphinium anthriscifolium* | MK253461 | 30 | 6349 | P | 30 | 45687 | 0 | 5.87E-09 |
| *Delphinium anthriscifolium* | MK253461 | 30 | 57741 | F | 30 | 57767 | -1 | 5.28E-07 |
| *Delphinium anthriscifolium* | MK253461 | 32 | 6296 | P | 32 | 6296 | -2 | 1.64E-06 |
| *Delphinium anthriscifolium* | MK253461 | 31 | 11302 | C | 31 | 67697 | -2 | 6.14E-06 |
| *Delphinium anthriscifolium* | MK253461 | 31 | 111519 | F | 31 | 111540 | -2 | 6.14E-06 |
| *Delphinium anthriscifolium* | MK253461 | 31 | 111519 | P | 31 | 129377 | -2 | 6.14E-06 |
| *Delphinium anthriscifolium* | MK253461 | 31 | 111540 | P | 31 | 129398 | -2 | 6.14E-06 |
| *Delphinium anthriscifolium* | MK253461 | 31 | 129377 | F | 31 | 129398 | -2 | 6.14E-06 |
| *Delphinium anthriscifolium* | MK253461 | 33 | 31953 | R | 33 | 60295 | -3 | 1.35E-05 |
| *Delphinium anthriscifolium* | MK253461 | 30 | 6346 | F | 30 | 35789 | -2 | 2.30E-05 |
| *Delphinium anthriscifolium* | MK253461 | 30 | 26068 | F | 30 | 26092 | -2 | 2.30E-05 |
| *Delphinium anthriscifolium* | MK253461 | 30 | 27410 | P | 30 | 27410 | -2 | 2.30E-05 |
| *Delphinium anthriscifolium* | MK253461 | 31 | 92612 | F | 31 | 92630 | -3 | 1.78E-04 |
| *Delphinium anthriscifolium* | MK253461 | 31 | 92612 | P | 31 | 148287 | -3 | 1.78E-04 |
| *Delphinium anthriscifolium* | MK253461 | 31 | 92630 | P | 31 | 148305 | -3 | 1.78E-04 |
| *Delphinium anthriscifolium* | MK253461 | 31 | 148287 | F | 31 | 148305 | -3 | 1.78E-04 |
| *Delphinium brunonianum* | NC_051554 | 26559 | 84512 | P | 26559 | 127367 | 0 | 0.00E+00 |
| *Delphinium brunonianum* | NC_051554 | 74 | 63476 | P | 74 | 63476 | -2 | 4.54E-31 |
| *Delphinium brunonianum* | NC_051554 | 57 | 74302 | P | 57 | 74302 | -3 | 2.53E-19 |
| *Delphinium brunonianum* | NC_051554 | 52 | 38484 | F | 52 | 40708 | -3 | 1.96E-16 |
| *Delphinium brunonianum* | NC_051554 | 42 | 93281 | P | 42 | 93281 | 0 | 3.45E-16 |
| *Delphinium brunonianum* | NC_051554 | 42 | 93281 | F | 42 | 145115 | 0 | 3.45E-16 |
| *Delphinium brunonianum* | NC_051554 | 42 | 145115 | P | 42 | 145115 | 0 | 3.45E-16 |
| *Delphinium brunonianum* | NC_051554 | 48 | 77819 | P | 48 | 77819 | -2 | 8.54E-16 |
| *Delphinium brunonianum* | NC_051554 | 39 | 43431 | F | 39 | 98690 | 0 | 2.20E-14 |
| *Delphinium brunonianum* | NC_051554 | 39 | 43431 | P | 39 | 139709 | 0 | 2.20E-14 |
| *Delphinium brunonianum* | NC_051554 | 33 | 31761 | F | 33 | 31794 | -1 | 8.94E-09 |
| *Delphinium brunonianum* | NC_051554 | 30 | 6310 | P | 30 | 45162 | -1 | 5.20E-07 |
| *Delphinium brunonianum* | NC_051554 | 32 | 6257 | P | 32 | 6257 | -2 | 1.61E-06 |
| *Delphinium brunonianum* | NC_051554 | 30 | 4438 | P | 30 | 4438 | -2 | 2.26E-05 |
| *Delphinium brunonianum* | NC_051554 | 30 | 6307 | F | 30 | 35273 | -2 | 2.26E-05 |
| *Delphinium brunonianum* | NC_051554 | 31 | 91250 | F | 31 | 91268 | -3 | 1.75E-04 |
| *Delphinium brunonianum* | NC_051554 | 31 | 91250 | P | 31 | 147139 | -3 | 1.75E-04 |
| *Delphinium brunonianum* | NC_051554 | 31 | 91268 | P | 31 | 147157 | -3 | 1.75E-04 |
| *Delphinium brunonianum* | NC_051554 | 31 | 147139 | F | 31 | 147157 | -3 | 1.75E-04 |
| *Delphinium candelabrum* var. *monanthum* | MW246165 | 26543 | 84862 | P | 26543 | 127452 | 0 | 0.00E+00 |
| *Delphinium candelabrum* var. *monanthum* | MW246165 | 74 | 63801 | P | 74 | 63801 | 0 | 1.87E-35 |
| *Delphinium candelabrum* var. *monanthum* | MW246165 | 57 | 74637 | P | 57 | 74637 | -3 | 2.54E-19 |
| *Delphinium candelabrum* var. *monanthum* | MW246165 | 52 | 38497 | F | 52 | 40721 | -3 | 1.96E-16 |
| *Delphinium candelabrum* var. *monanthum* | MW246165 | 42 | 93631 | P | 42 | 93631 | 0 | 3.45E-16 |
| *Delphinium candelabrum* var. *monanthum* | MW246165 | 42 | 93631 | F | 42 | 145184 | 0 | 3.45E-16 |
| *Delphinium candelabrum* var. *monanthum* | MW246165 | 42 | 145184 | P | 42 | 145184 | 0 | 3.45E-16 |
| *Delphinium candelabrum* var. *monanthum* | MW246165 | 48 | 78148 | P | 48 | 78148 | -2 | 8.55E-16 |
| *Delphinium candelabrum* var. *monanthum* | MW246165 | 39 | 43445 | F | 39 | 99040 | 0 | 2.21E-14 |
| *Delphinium candelabrum* var. *monanthum* | MW246165 | 39 | 43445 | P | 39 | 139778 | 0 | 2.21E-14 |
| *Delphinium candelabrum* var. *monanthum* | MW246165 | 30 | 4440 | P | 30 | 4440 | 0 | 5.79E-09 |
| *Delphinium candelabrum* var. *monanthum* | MW246165 | 30 | 6344 | P | 30 | 45179 | -1 | 5.21E-07 |
| *Delphinium candelabrum* var. *monanthum* | MW246165 | 32 | 6291 | P | 32 | 6291 | -2 | 1.61E-06 |
| *Delphinium candelabrum* var. *monanthum* | MW246165 | 30 | 6341 | F | 30 | 35287 | -2 | 2.26E-05 |
| *Delphinium candelabrum* var. *monanthum* | MW246165 | 32 | 31788 | F | 32 | 31825 | -3 | 4.84E-05 |
| *Delphinium candelabrum* var. *monanthum* | MW246165 | 31 | 4520 | F | 31 | 54901 | -3 | 1.76E-04 |
| *Delphinium candelabrum* var. *monanthum* | MW246165 | 31 | 91600 | F | 31 | 91618 | -3 | 1.76E-04 |
| *Delphinium candelabrum* var. *monanthum* | MW246165 | 31 | 91600 | P | 31 | 147208 | -3 | 1.76E-04 |
| *Delphinium candelabrum* var. *monanthum* | MW246165 | 31 | 91618 | P | 31 | 147226 | -3 | 1.76E-04 |
| *Delphinium candelabrum* var. *monanthum* | MW246165 | 31 | 147208 | F | 31 | 147226 | -3 | 1.76E-04 |
| *Delphinium candelabrum* var. *monanthum* | MW246165 | 30 | 54903 | P | 30 | 82282 | -3 | 6.34E-04 |
| *Delphinium ceratophorum* | MK253460 | 26560 | 84801 | P | 26560 | 127685 | 0 | 0.00E+00 |
| *Delphinium ceratophorum* | MK253460 | 74 | 63727 | P | 74 | 63727 | 0 | 1.88E-35 |
| *Delphinium ceratophorum* | MK253460 | 68 | 66857 | P | 68 | 66857 | 0 | 7.68E-32 |
| *Delphinium ceratophorum* | MK253460 | 57 | 74596 | P | 57 | 74596 | -3 | 2.55E-19 |
| *Delphinium ceratophorum* | MK253460 | 52 | 38520 | F | 52 | 40744 | -3 | 1.97E-16 |
| *Delphinium ceratophorum* | MK253460 | 42 | 93571 | P | 42 | 93571 | 0 | 3.46E-16 |
| *Delphinium ceratophorum* | MK253460 | 42 | 93571 | F | 42 | 145433 | 0 | 3.46E-16 |
| *Delphinium ceratophorum* | MK253460 | 42 | 145433 | P | 42 | 145433 | 0 | 3.46E-16 |
| *Delphinium ceratophorum* | MK253460 | 39 | 43477 | F | 39 | 98980 | 0 | 2.21E-14 |
| *Delphinium ceratophorum* | MK253460 | 39 | 43477 | P | 39 | 140027 | 0 | 2.21E-14 |
| *Delphinium ceratophorum* | MK253460 | 30 | 4451 | P | 30 | 4451 | 0 | 5.80E-09 |
| *Delphinium ceratophorum* | MK253460 | 33 | 31793 | F | 33 | 31826 | -1 | 8.98E-09 |
| *Delphinium ceratophorum* | MK253460 | 33 | 119099 | P | 33 | 119099 | -1 | 8.98E-09 |
| *Delphinium ceratophorum* | MK253460 | 34 | 78122 | P | 34 | 78122 | -2 | 1.14E-07 |
| *Delphinium ceratophorum* | MK253460 | 30 | 6331 | P | 30 | 45210 | -1 | 5.22E-07 |
| *Delphinium ceratophorum* | MK253460 | 32 | 6278 | P | 32 | 6278 | -2 | 1.62E-06 |
| *Delphinium ceratophorum* | MK253460 | 30 | 6328 | F | 30 | 35303 | -2 | 2.27E-05 |
| *Delphinium ceratophorum* | MK253460 | 31 | 59307 | P | 31 | 126018 | -3 | 1.76E-04 |
| *Delphinium ceratophorum* | MK253460 | 31 | 91540 | F | 31 | 91558 | -3 | 1.76E-04 |
| *Delphinium ceratophorum* | MK253460 | 31 | 91540 | P | 31 | 147457 | -3 | 1.76E-04 |
| *Delphinium ceratophorum* | MK253460 | 31 | 91558 | P | 31 | 147475 | -3 | 1.76E-04 |
| *Delphinium ceratophorum* | MK253460 | 31 | 113822 | F | 31 | 113843 | -3 | 1.76E-04 |
| *Delphinium ceratophorum* | MK253460 | 31 | 127276 | F | 31 | 127285 | -3 | 1.76E-04 |
| *Delphinium ceratophorum* | MK253460 | 31 | 147457 | F | 31 | 147475 | -3 | 1.76E-04 |
| *Delphinium elatum* var. *sericeum* | LHM1265 | 26561 | 84780 | P | 26561 | 127658 | 0 | 0.00E+00 |
| *Delphinium elatum* var. *sericeum* | LHM1265 | 80 | 63674 | P | 80 | 63674 | 0.00E+00 | 4.58E-39 |
| *Delphinium elatum* var. *sericeum* | LHM1265 | 57 | 74571 | P | 57 | 74571 | -3.00E+00 | 2.54E-19 |
| *Delphinium elatum* var. *sericeum* | LHM1265 | 52 | 38381 | F | 52 | 40605 | -3.00E+00 | 1.97E-16 |
| *Delphinium elatum* var. *sericeum* | LHM1265 | 42 | 93550 | P | 42 | 93550 | 0.00E+00 | 3.46E-16 |
| *Delphinium elatum* var. *sericeum* | LHM1265 | 42 | 93550 | F | 42 | 145407 | 0.00E+00 | 3.46E-16 |
| *Delphinium elatum* var. *sericeum* | LHM1265 | 42 | 145407 | P | 42 | 145407 | 0.00E+00 | 3.46E-16 |
| *Delphinium elatum* var. *sericeum* | LHM1265 | 48 | 78095 | P | 48 | 78095 | -2.00E+00 | 8.57E-16 |
| *Delphinium elatum* var. *sericeum* | LHM1265 | 39 | 43340 | F | 39 | 98959 | 0.00E+00 | 2.21E-14 |
| *Delphinium elatum* var. *sericeum* | LHM1265 | 39 | 43340 | P | 39 | 140001 | 0.00E+00 | 2.21E-14 |
| *Delphinium elatum* var. *sericeum* | LHM1265 | 33 | 119054 | P | 33 | 119054 | -1.00E+00 | 8.97E-09 |
| *Delphinium elatum* var. *sericeum* | LHM1265 | 30 | 6308 | P | 30 | 45072 | -1.00E+00 | 5.22E-07 |
| *Delphinium elatum* var. *sericeum* | LHM1265 | 32 | 6255 | P | 32 | 6255 | -2.00E+00 | 1.62E-06 |
| *Delphinium elatum* var. *sericeum* | LHM1265 | 30 | 4437 | P | 30 | 4437 | -2.00E+00 | 2.27E-05 |
| *Delphinium elatum* var. *sericeum* | LHM1265 | 30 | 6305 | F | 30 | 35165 | -2.00E+00 | 2.27E-05 |
| *Delphinium elatum* var. *sericeum* | LHM1265 | 31 | 91519 | F | 31 | 91537 | -3.00E+00 | 1.76E-04 |
| *Delphinium elatum* var. *sericeum* | LHM1265 | 31 | 91519 | P | 31 | 147431 | -3.00E+00 | 1.76E-04 |
| *Delphinium elatum* var. *sericeum* | LHM1265 | 31 | 91537 | P | 31 | 147449 | -3.00E+00 | 1.76E-04 |
| *Delphinium elatum* var. *sericeum* | LHM1265 | 31 | 147431 | F | 31 | 147449 | -3.00E+00 | 1.76E-04 |
| *Delphinium iliense* | LHM1285 | 26561 | 84780 | P | 26561 | 127658 | 0 | 0.00E+00 |
| *Delphinium iliense* | LHM1285 | 80 | 63674 | P | 80 | 63674 | 0 | 4.58E-39 |
| *Delphinium iliense* | LHM1285 | 57 | 74571 | P | 57 | 74571 | -3 | 2.54E-19 |
| *Delphinium iliense* | LHM1285 | 52 | 38381 | F | 52 | 40605 | -3 | 1.97E-16 |
| *Delphinium iliense* | LHM1285 | 42 | 93550 | P | 42 | 93550 | 0 | 3.46E-16 |
| *Delphinium iliense* | LHM1285 | 42 | 93550 | F | 42 | 145407 | 0 | 3.46E-16 |
| *Delphinium iliense* | LHM1285 | 42 | 145407 | P | 42 | 145407 | 0 | 3.46E-16 |
| *Delphinium iliense* | LHM1285 | 48 | 78095 | P | 48 | 78095 | -2 | 8.57E-16 |
| *Delphinium iliense* | LHM1285 | 39 | 43340 | F | 39 | 98959 | 0 | 2.21E-14 |
| *Delphinium iliense* | LHM1285 | 39 | 43340 | P | 39 | 140001 | 0 | 2.21E-14 |
| *Delphinium iliense* | LHM1285 | 33 | 119054 | P | 33 | 119054 | -1 | 8.97E-09 |
| *Delphinium iliense* | LHM1285 | 30 | 6308 | P | 30 | 45072 | -1 | 5.22E-07 |
| *Delphinium iliense* | LHM1285 | 32 | 6255 | P | 32 | 6255 | -2 | 1.62E-06 |
| *Delphinium iliense* | LHM1285 | 30 | 4437 | P | 30 | 4437 | -2 | 2.27E-05 |
| *Delphinium iliense* | LHM1285 | 30 | 6305 | F | 30 | 35165 | -2 | 2.27E-05 |
| *Delphinium iliense* | LHM1285 | 31 | 91519 | F | 31 | 91537 | -3 | 1.76E-04 |
| *Delphinium iliense* | LHM1285 | 31 | 91519 | P | 31 | 147431 | -3 | 1.76E-04 |
| *Delphinium iliense* | LHM1285 | 31 | 91537 | P | 31 | 147449 | -3 | 1.76E-04 |
| *Delphinium iliense* | LHM1285 | 31 | 147431 | F | 31 | 147449 | -3 | 1.76E-04 |
| *Delphinium maackianum* | NC_047293 | 26564 | 85055 | P | 26564 | 127920 | 0 | 0.00E+00 |
| *Delphinium maackianum* | NC_047293 | 74 | 63916 | P | 74 | 63916 | 0 | 1.88E-35 |
| *Delphinium maackianum* | NC_047293 | 57 | 74839 | P | 57 | 74839 | -3 | 2.55E-19 |
| *Delphinium maackianum* | NC_047293 | 52 | 38587 | F | 52 | 40811 | -3 | 1.97E-16 |
| *Delphinium maackianum* | NC_047293 | 42 | 93824 | P | 42 | 93824 | 0 | 3.47E-16 |
| *Delphinium maackianum* | NC_047293 | 42 | 93824 | F | 42 | 145673 | 0 | 3.47E-16 |
| *Delphinium maackianum* | NC_047293 | 42 | 145673 | P | 42 | 145673 | 0 | 3.47E-16 |
| *Delphinium maackianum* | NC_047293 | 48 | 78350 | P | 48 | 78350 | -2 | 8.60E-16 |
| *Delphinium maackianum* | NC_047293 | 39 | 43554 | F | 39 | 99233 | 0 | 2.22E-14 |
| *Delphinium maackianum* | NC_047293 | 39 | 43554 | P | 39 | 140267 | 0 | 2.22E-14 |
| *Delphinium maackianum* | NC_047293 | 30 | 4464 | P | 30 | 4464 | 0 | 5.82E-09 |
| *Delphinium maackianum* | NC_047293 | 32 | 31859 | F | 32 | 31896 | -1 | 3.49E-08 |
| *Delphinium maackianum* | NC_047293 | 30 | 6343 | P | 30 | 45288 | -1 | 5.24E-07 |
| *Delphinium maackianum* | NC_047293 | 30 | 67343 | R | 30 | 67345 | -1 | 5.24E-07 |
| *Delphinium maackianum* | NC_047293 | 32 | 6290 | P | 32 | 6290 | -2 | 1.62E-06 |
| *Delphinium maackianum* | NC_047293 | 33 | 119331 | P | 33 | 119331 | -3 | 1.34E-05 |
| *Delphinium maackianum* | NC_047293 | 30 | 6340 | F | 30 | 35371 | -2 | 2.28E-05 |
| *Delphinium maackianum* | NC_047293 | 30 | 67143 | R | 30 | 67143 | -2 | 2.28E-05 |
| *Delphinium maackianum* | NC_047293 | 30 | 71059 | P | 30 | 71059 | -2 | 2.28E-05 |
| *Delphinium maackianum* | NC_047293 | 31 | 91793 | F | 31 | 91811 | -3 | 1.77E-04 |
| *Delphinium maackianum* | NC_047293 | 31 | 91793 | P | 31 | 147697 | -3 | 1.77E-04 |
| *Delphinium maackianum* | NC_047293 | 31 | 91811 | P | 31 | 147715 | -3 | 1.77E-04 |
| *Delphinium maackianum* | NC_047293 | 31 | 147697 | F | 31 | 147715 | -3 | 1.77E-04 |
| *Delphinium maackianum* | NC_047293 | 30 | 4534 | C | 30 | 99588 | -3 | 6.38E-04 |
| *Delphinium maackianum* | NC_047293 | 30 | 4534 | R | 30 | 139921 | -3 | 6.38E-04 |
| *Delphinium maackianum* | NC_047293 | 30 | 46847 | F | 30 | 82511 | -3 | 6.38E-04 |
| *Delphinium mollifolium* | LHM1295 | 26331 | 85018 | P | 26331 | 127648 | 0 | 0.00E+00 |
| *Delphinium mollifolium* | LHM1295 | 74 | 63692 | P | 74 | 63692 | 0 | 1.87E-35 |
| *Delphinium mollifolium* | LHM1295 | 52 | 38376 | F | 52 | 40600 | -3 | 1.96E-16 |
| *Delphinium mollifolium* | LHM1295 | 42 | 93559 | P | 42 | 93559 | 0 | 3.45E-16 |
| *Delphinium mollifolium* | LHM1295 | 42 | 93559 | F | 42 | 145396 | 0 | 3.45E-16 |
| *Delphinium mollifolium* | LHM1295 | 42 | 145396 | P | 42 | 145396 | 0 | 3.45E-16 |
| *Delphinium mollifolium* | LHM1295 | 48 | 78096 | P | 48 | 78096 | -2 | 8.54E-16 |
| *Delphinium mollifolium* | LHM1295 | 39 | 43345 | F | 39 | 98968 | 0 | 2.21E-14 |
| *Delphinium mollifolium* | LHM1295 | 39 | 43345 | P | 39 | 139990 | 0 | 2.21E-14 |
| *Delphinium mollifolium* | LHM1295 | 33 | 119055 | P | 33 | 119055 | -1 | 8.95E-09 |
| *Delphinium mollifolium* | LHM1295 | 37 | 74583 | P | 37 | 74583 | -3 | 7.41E-08 |
| *Delphinium mollifolium* | LHM1295 | 36 | 74573 | P | 36 | 74594 | -3 | 2.72E-07 |
| *Delphinium mollifolium* | LHM1295 | 30 | 6268 | P | 30 | 45079 | -1 | 5.21E-07 |
| *Delphinium mollifolium* | LHM1295 | 32 | 6215 | P | 32 | 6215 | -2 | 1.61E-06 |
| *Delphinium mollifolium* | LHM1295 | 30 | 4399 | P | 30 | 4399 | -2 | 2.26E-05 |
| *Delphinium mollifolium* | LHM1295 | 30 | 6265 | F | 30 | 35154 | -2 | 2.26E-05 |
| *Delphinium mollifolium* | LHM1295 | 31 | 91528 | F | 31 | 91546 | -3 | 1.75E-04 |
| *Delphinium mollifolium* | LHM1295 | 31 | 91528 | P | 31 | 147420 | -3 | 1.75E-04 |
| *Delphinium mollifolium* | LHM1295 | 31 | 91546 | P | 31 | 147438 | -3 | 1.75E-04 |
| *Delphinium mollifolium* | LHM1295 | 31 | 147420 | F | 31 | 147438 | -3 | 1.75E-04 |
| *Delphinium sauricum* | LHM1266 | 26594 | 84765 | P | 26594 | 127661 | 0 | 0.00E+00 |
| *Delphinium sauricum* | LHM1266 | 74 | 63680 | P | 74 | 63680 | 0 | 1.88E-35 |
| *Delphinium sauricum* | LHM1266 | 52 | 38377 | F | 52 | 40601 | -3 | 1.97E-16 |
| *Delphinium sauricum* | LHM1266 | 42 | 93569 | P | 42 | 93569 | 0 | 3.46E-16 |
| *Delphinium sauricum* | LHM1266 | 42 | 93569 | F | 42 | 145409 | 0 | 3.46E-16 |
| *Delphinium sauricum* | LHM1266 | 42 | 145409 | P | 42 | 145409 | 0 | 3.46E-16 |
| *Delphinium sauricum* | LHM1266 | 48 | 78103 | P | 48 | 78103 | -2 | 8.58E-16 |
| *Delphinium sauricum* | LHM1266 | 39 | 43339 | F | 39 | 98978 | 0 | 2.21E-14 |
| *Delphinium sauricum* | LHM1266 | 39 | 43339 | P | 39 | 140003 | 0 | 2.21E-14 |
| *Delphinium sauricum* | LHM1266 | 34 | 66856 | R | 34 | 66856 | 0 | 2.27E-11 |
| *Delphinium sauricum* | LHM1266 | 30 | 4400 | P | 30 | 4400 | 0 | 5.80E-09 |
| *Delphinium sauricum* | LHM1266 | 33 | 119068 | P | 33 | 119068 | -1 | 8.98E-09 |
| *Delphinium sauricum* | LHM1266 | 37 | 74588 | P | 37 | 74588 | -3 | 7.43E-08 |
| *Delphinium sauricum* | LHM1266 | 36 | 74578 | P | 36 | 74599 | -3 | 2.73E-07 |
| *Delphinium sauricum* | LHM1266 | 30 | 6289 | P | 30 | 45071 | -1 | 5.22E-07 |
| *Delphinium sauricum* | LHM1266 | 32 | 4900 | F | 32 | 4921 | -2 | 1.62E-06 |
| *Delphinium sauricum* | LHM1266 | 32 | 6236 | P | 32 | 6236 | -2 | 1.62E-06 |
| *Delphinium sauricum* | LHM1266 | 30 | 6286 | F | 30 | 35158 | -2 | 2.27E-05 |
| *Delphinium sauricum* | LHM1266 | 31 | 91538 | F | 31 | 91556 | -3 | 1.76E-04 |
| *Delphinium sauricum* | LHM1266 | 31 | 91538 | P | 31 | 147433 | -3 | 1.76E-04 |
| *Delphinium sauricum* | LHM1266 | 31 | 91556 | P | 31 | 147451 | -3 | 1.76E-04 |
| *Delphinium sauricum* | LHM1266 | 31 | 147433 | F | 31 | 147451 | -3 | 1.76E-04 |
| *Delphinium sauricum* | LHM1266 | 30 | 4470 | C | 30 | 99333 | -3 | 6.36E-04 |
| *Delphinium sauricum* | LHM1266 | 30 | 4470 | R | 30 | 139657 | -3 | 6.36E-04 |
| *Delphinium shawurense* | LHM1271 | 26561 | 84820 | P | 26561 | 127723 | 0 | 0.00E+00 |
| *Delphinium shawurense* | LHM1271 | 68 | 63714 | P | 68 | 63714 | 0 | 7.69E-32 |
| *Delphinium shawurense* | LHM1271 | 57 | 74620 | P | 57 | 74620 | -1 | 5.51E-23 |
| *Delphinium shawurense* | LHM1271 | 52 | 38414 | F | 52 | 40638 | -3 | 1.97E-16 |
| *Delphinium shawurense* | LHM1271 | 42 | 93590 | P | 42 | 93590 | 0 | 3.46E-16 |
| *Delphinium shawurense* | LHM1271 | 42 | 93590 | F | 42 | 145472 | 0 | 3.46E-16 |
| *Delphinium shawurense* | LHM1271 | 42 | 145472 | P | 42 | 145472 | 0 | 3.46E-16 |
| *Delphinium shawurense* | LHM1271 | 48 | 78143 | P | 48 | 78143 | -2 | 8.58E-16 |
| *Delphinium shawurense* | LHM1271 | 39 | 43370 | F | 39 | 98999 | 0 | 2.22E-14 |
| *Delphinium shawurense* | LHM1271 | 39 | 43370 | P | 39 | 140066 | 0 | 2.22E-14 |
| *Delphinium shawurense* | LHM1271 | 30 | 6313 | P | 30 | 45100 | -1 | 5.23E-07 |
| *Delphinium shawurense* | LHM1271 | 32 | 6260 | P | 32 | 6260 | -2 | 1.62E-06 |
| *Delphinium shawurense* | LHM1271 | 33 | 119111 | P | 33 | 119111 | -3 | 1.34E-05 |
| *Delphinium shawurense* | LHM1271 | 30 | 4440 | P | 30 | 4440 | -2 | 2.27E-05 |
| *Delphinium shawurense* | LHM1271 | 30 | 6310 | F | 30 | 35198 | -2 | 2.27E-05 |
| *Delphinium shawurense* | LHM1271 | 31 | 91559 | F | 31 | 91577 | -3 | 1.76E-04 |
| *Delphinium shawurense* | LHM1271 | 31 | 91559 | P | 31 | 147496 | -3 | 1.76E-04 |
| *Delphinium shawurense* | LHM1271 | 31 | 91577 | P | 31 | 147514 | -3 | 1.76E-04 |
| *Delphinium shawurense* | LHM1271 | 31 | 147496 | F | 31 | 147514 | -3 | 1.76E-04 |
| *Delphinium winklerianum* | LHM1299 | 26594 | 84754 | P | 26594 | 127641 | 0 | 0.00E+00 |
| *Delphinium winklerianum* | LHM1299 | 74 | 63659 | P | 74 | 63659 | 0 | 1.88E-35 |
| *Delphinium winklerianum* | LHM1299 | 52 | 38365 | F | 52 | 40589 | -3 | 1.97E-16 |
| *Delphinium winklerianum* | LHM1299 | 42 | 93558 | P | 42 | 93558 | 0 | 3.46E-16 |
| *Delphinium winklerianum* | LHM1299 | 42 | 93558 | F | 42 | 145389 | 0 | 3.46E-16 |
| *Delphinium winklerianum* | LHM1299 | 42 | 145389 | P | 42 | 145389 | 0 | 3.46E-16 |
| *Delphinium winklerianum* | LHM1299 | 48 | 78095 | P | 48 | 78095 | -2 | 8.57E-16 |
| *Delphinium winklerianum* | LHM1299 | 39 | 43320 | F | 39 | 98967 | 0 | 2.21E-14 |
| *Delphinium winklerianum* | LHM1299 | 39 | 43320 | P | 39 | 139983 | 0 | 2.21E-14 |
| *Delphinium winklerianum* | LHM1299 | 34 | 66852 | R | 34 | 66852 | 0 | 2.27E-11 |
| *Delphinium winklerianum* | LHM1299 | 33 | 119061 | P | 33 | 119061 | -1 | 8.98E-09 |
| *Delphinium winklerianum* | LHM1299 | 37 | 74582 | P | 37 | 74582 | -3 | 7.43E-08 |
| *Delphinium winklerianum* | LHM1299 | 36 | 74572 | P | 36 | 74593 | -3 | 2.73E-07 |
| *Delphinium winklerianum* | LHM1299 | 30 | 6268 | P | 30 | 45052 | -1 | 5.22E-07 |
| *Delphinium winklerianum* | LHM1299 | 32 | 6215 | P | 32 | 6215 | -2 | 1.62E-06 |
| *Delphinium winklerianum* | LHM1299 | 30 | 4400 | P | 30 | 4400 | -2 | 2.27E-05 |
| *Delphinium winklerianum* | LHM1299 | 30 | 6265 | F | 30 | 35143 | -2 | 2.27E-05 |
| *Delphinium winklerianum* | LHM1299 | 31 | 91527 | F | 31 | 91545 | -3 | 1.76E-04 |
| *Delphinium winklerianum* | LHM1299 | 31 | 91527 | P | 31 | 147413 | -3 | 1.76E-04 |
| *Delphinium winklerianum* | LHM1299 | 31 | 91545 | P | 31 | 147431 | -3 | 1.76E-04 |
| *Delphinium winklerianum* | LHM1299 | 31 | 147413 | F | 31 | 147431 | -3 | 1.76E-04 |
| *Delphinium yunnanense* | MW246156 | 26551 | 84639 | P | 26551 | 127502 | 0 | 0.00E+00 |
| *Delphinium yunnanense* | MW246156 | 74 | 63655 | P | 74 | 63655 | 0 | 1.87E-35 |
| *Delphinium yunnanense* | MW246156 | 57 | 74463 | P | 57 | 74463 | -3 | 2.54E-19 |
| *Delphinium yunnanense* | MW246156 | 52 | 38431 | F | 52 | 40655 | -3 | 1.96E-16 |
| *Delphinium yunnanense* | MW246156 | 42 | 93402 | P | 42 | 93402 | 0 | 3.45E-16 |
| *Delphinium yunnanense* | MW246156 | 42 | 93402 | F | 42 | 145248 | 0 | 3.45E-16 |
| *Delphinium yunnanense* | MW246156 | 42 | 145248 | P | 42 | 145248 | 0 | 3.45E-16 |
| *Delphinium yunnanense* | MW246156 | 48 | 77979 | P | 48 | 77979 | -2 | 8.55E-16 |
| *Delphinium yunnanense* | MW246156 | 39 | 43388 | F | 39 | 98811 | 0 | 2.21E-14 |
| *Delphinium yunnanense* | MW246156 | 39 | 43388 | P | 39 | 139842 | 0 | 2.21E-14 |
| *Delphinium yunnanense* | MW246156 | 30 | 4442 | P | 30 | 4442 | 0 | 5.79E-09 |
| *Delphinium yunnanense* | MW246156 | 33 | 118941 | P | 33 | 118941 | -1 | 8.96E-09 |
| *Delphinium yunnanense* | MW246156 | 30 | 6321 | P | 30 | 45123 | -1 | 5.21E-07 |
| *Delphinium yunnanense* | MW246156 | 32 | 6268 | P | 32 | 6268 | -2 | 1.62E-06 |
| *Delphinium yunnanense* | MW246156 | 30 | 6318 | F | 30 | 35220 | -2 | 2.27E-05 |
| *Delphinium yunnanense* | MW246156 | 32 | 31714 | F | 32 | 31751 | -3 | 4.85E-05 |
| *Delphinium yunnanense* | MW246156 | 31 | 91377 | F | 31 | 91395 | -3 | 1.76E-04 |
| *Delphinium yunnanense* | MW246156 | 31 | 91377 | P | 31 | 147266 | -3 | 1.76E-04 |
| *Delphinium yunnanense* | MW246156 | 31 | 91395 | P | 31 | 147284 | -3 | 1.76E-04 |
| *Delphinium yunnanense* | MW246156 | 31 | 147266 | F | 31 | 147284 | -3 | 1.76E-04 |
| *Delphinium yunnanense* | MW246156 | 30 | 4512 | C | 30 | 99166 | -3 | 6.35E-04 |
| *Delphinium yunnanense* | MW246156 | 30 | 4512 | R | 30 | 139496 | -3 | 6.35E-04 |
| *Delphinium yunnanense* | MW246156 | 30 | 118897 | F | 30 | 118922 | -3 | 6.35E-04 |
