# Supplementary material for: Complete chloroplast genomes of eight Delphinium taxa (Ranunculaceae) endemic to Xinjiang, China: insights into genome structure, comparative analysis, and phylogenetic relationships
Source: BMC Plant Biol. 2024 Jun 26;24:600. doi: 10.1186/s12870-024-05279-y (PMC11201361; doi:10.1186/s12870-024-05279-y)
Supplement: Supplementary file 4 — Supplementary Material 4 [file 12870_2024_5279_MOESM4_ESM.docx]

**TABLE S4** The nucleotide variability (Pi) of 14 *Delphinium* taxa in whole chloroplast genomes.

| **Window** | **Midpoint** | **Pi** | **Theta** | **S** |
| --- | --- | --- | --- | --- |
| 1-709 | 409 | 0.01183 | 0.01992 | 38 |
| 310-909 | 609 | 0.00533 | 0.01048 | 20 |
| 510-1109 | 809 | 0.00322 | 0.00629 | 12 |
| 710-1309 | 1009 | 0.00319 | 0.00576 | 11 |
| 910-1509 | 1209 | 0.00234 | 0.00472 | 9 |
| 1110-1722 | 1409 | 0.00441 | 0.00839 | 16 |
| 1310-1924 | 1609 | 0.00636 | 0.0131 | 25 |
| 1510-2152 | 1824 | 0.00875 | 0.01677 | 32 |
| 1723-2352 | 2039 | 0.00894 | 0.01572 | 30 |
| 1925-2552 | 2252 | 0.01033 | 0.01834 | 35 |
| 2153-2752 | 2452 | 0.01 | 0.01834 | 35 |
| 2353-2958 | 2652 | 0.00894 | 0.01834 | 35 |
| 2553-3158 | 2858 | 0.00714 | 0.01363 | 26 |
| 2753-3358 | 3058 | 0.00819 | 0.0152 | 29 |
| 2959-3558 | 3258 | 0.00998 | 0.01834 | 35 |
| 3159-3775 | 3458 | 0.01106 | 0.02149 | 41 |
| 3359-3982 | 3658 | 0.01081 | 0.02254 | 43 |
| 3559-4189 | 3881 | 0.01176 | 0.02254 | 43 |
| 3776-4389 | 4088 | 0.01029 | 0.01834 | 35 |
| 3983-4605 | 4289 | 0.01194 | 0.01834 | 35 |
| 4190-4844 | 4505 | 0.01414 | 0.02306 | 44 |
| 4390-5050 | 4721 | 0.01617 | 0.02725 | 52 |
| 4606-5280 | 4950 | 0.01581 | 0.02882 | 55 |
| 4845-5480 | 5180 | 0.01158 | 0.02254 | 43 |
| 5051-5680 | 5380 | 0.00912 | 0.01625 | 31 |
| 5281-5894 | 5580 | 0.00546 | 0.00943 | 18 |
| 5481-6148 | 5780 | 0.00586 | 0.00891 | 17 |
| 5681-6357 | 6010 | 0.00575 | 0.00996 | 19 |
| 5895-6570 | 6252 | 0.00733 | 0.01101 | 21 |
| 6149-6809 | 6470 | 0.00727 | 0.01101 | 21 |
| 6358-7018 | 6670 | 0.01196 | 0.02044 | 39 |
| 6571-7289 | 6913 | 0.01429 | 0.02673 | 51 |
| 6810-7489 | 7168 | 0.01227 | 0.02358 | 45 |
| 7019-7720 | 7389 | 0.01095 | 0.01939 | 37 |
| 7290-7935 | 7594 | 0.00982 | 0.01677 | 32 |
| 7490-8140 | 7835 | 0.01029 | 0.01782 | 34 |
| 7721-8346 | 8035 | 0.00956 | 0.01782 | 34 |
| 7936-8561 | 8240 | 0.01049 | 0.01782 | 34 |
| 8141-8766 | 8449 | 0.00954 | 0.01572 | 30 |
| 8347-8966 | 8666 | 0.0056 | 0.00891 | 17 |
| 8562-9166 | 8866 | 0.00234 | 0.00472 | 9 |
| 8767-9366 | 9066 | 0.00234 | 0.00472 | 9 |
| 8967-9566 | 9266 | 0.00187 | 0.00367 | 7 |
| 9167-9766 | 9466 | 0.0026 | 0.00314 | 6 |
| 9367-9966 | 9666 | 0.00328 | 0.00419 | 8 |
| 9567-10166 | 9866 | 0.00375 | 0.00524 | 10 |
| 9767-10372 | 10066 | 0.00386 | 0.00681 | 13 |
| 9967-10572 | 10272 | 0.0052 | 0.00891 | 17 |
| 10167-10777 | 10472 | 0.00615 | 0.01101 | 21 |
| 10373-10993 | 10672 | 0.00604 | 0.01153 | 22 |
| 10573-11216 | 10877 | 0.00568 | 0.01153 | 22 |
| 10778-11422 | 11098 | 0.00705 | 0.01258 | 24 |
| 10994-11625 | 11316 | 0.00659 | 0.01205 | 23 |
| 11217-11863 | 11522 | 0.00841 | 0.01467 | 28 |
| 11423-12086 | 11753 | 0.00826 | 0.01363 | 26 |
| 11626-12286 | 11981 | 0.00683 | 0.01048 | 20 |
| 11864-12728 | 12186 | 0.00432 | 0.00681 | 13 |
| 12087-12968 | 12414 | 0.00333 | 0.00734 | 14 |
| 12287-13195 | 12828 | 0.00676 | 0.01258 | 24 |
| 12729-13448 | 13094 | 0.00883 | 0.01572 | 30 |
| 12969-13660 | 13296 | 0.01002 | 0.01834 | 35 |
| 13196-13860 | 13560 | 0.00863 | 0.01572 | 30 |
| 13449-14060 | 13760 | 0.00537 | 0.00996 | 19 |
| 13661-14260 | 13960 | 0.00434 | 0.00629 | 12 |
| 13861-14481 | 14160 | 0.00447 | 0.00681 | 13 |
| 14061-14687 | 14370 | 0.00542 | 0.00891 | 17 |
| 14261-14887 | 14587 | 0.00518 | 0.00839 | 16 |
| 14482-15087 | 14787 | 0.00326 | 0.00576 | 11 |
| 14688-15290 | 14987 | 0.00326 | 0.00576 | 11 |
| 14888-15518 | 15187 | 0.00452 | 0.00839 | 16 |
| 15088-15718 | 15412 | 0.00476 | 0.00891 | 17 |
| 15291-15918 | 15618 | 0.00405 | 0.00734 | 14 |
| 15519-16118 | 15818 | 0.00306 | 0.00629 | 12 |
| 15719-16318 | 16018 | 0.00282 | 0.00576 | 11 |
| 15919-16524 | 16218 | 0.0033 | 0.00681 | 13 |
| 16119-16724 | 16424 | 0.00445 | 0.00891 | 17 |
| 16319-16930 | 16624 | 0.00689 | 0.01205 | 23 |
| 16525-17130 | 16824 | 0.00665 | 0.01153 | 22 |
| 16725-17330 | 17030 | 0.0059 | 0.00996 | 19 |
| 16931-17530 | 17230 | 0.00509 | 0.00996 | 19 |
| 17131-17733 | 17430 | 0.00509 | 0.00996 | 19 |
| 17331-17939 | 17630 | 0.00377 | 0.00786 | 15 |
| 17531-18139 | 17839 | 0.00661 | 0.00996 | 19 |
| 17734-18339 | 18039 | 0.00685 | 0.01048 | 20 |
| 17940-18539 | 18239 | 0.00811 | 0.01153 | 22 |
| 18140-18739 | 18439 | 0.00412 | 0.00734 | 14 |
| 18340-18939 | 18639 | 0.00412 | 0.00734 | 14 |
| 18540-19139 | 18839 | 0.00214 | 0.00472 | 9 |
| 18740-19339 | 19039 | 0.00214 | 0.00472 | 9 |
| 18940-19539 | 19239 | 0.00238 | 0.00524 | 10 |
| 19140-19750 | 19439 | 0.00473 | 0.00996 | 19 |
| 19340-19957 | 19639 | 0.00544 | 0.01153 | 22 |
| 19540-20157 | 19857 | 0.00592 | 0.01258 | 24 |
| 19751-20357 | 20057 | 0.00531 | 0.00996 | 19 |
| 19958-20557 | 20257 | 0.00364 | 0.00629 | 12 |
| 20158-20757 | 20457 | 0.00293 | 0.00472 | 9 |
| 20358-20957 | 20657 | 0.00211 | 0.00419 | 8 |
| 20558-21157 | 20857 | 0.00258 | 0.00524 | 10 |
| 20758-21357 | 21057 | 0.00282 | 0.00576 | 11 |
| 20958-21563 | 21257 | 0.00478 | 0.00734 | 14 |
| 21158-21764 | 21457 | 0.00716 | 0.01258 | 24 |
| 21358-21964 | 21664 | 0.00738 | 0.01258 | 24 |
| 21564-22164 | 21864 | 0.00581 | 0.01101 | 21 |
| 21765-22364 | 22064 | 0.00379 | 0.00576 | 11 |
| 21965-22564 | 22264 | 0.00368 | 0.00472 | 9 |
| 22165-22766 | 22464 | 0.00214 | 0.00262 | 5 |
| 22365-22966 | 22666 | 0.00225 | 0.00367 | 7 |
| 22565-23166 | 22866 | 0.00214 | 0.00472 | 9 |
| 22767-23366 | 23066 | 0.00262 | 0.00576 | 11 |
| 22967-23566 | 23266 | 0.00418 | 0.00734 | 14 |
| 23167-23766 | 23466 | 0.0037 | 0.00629 | 12 |
| 23367-23966 | 23666 | 0.00441 | 0.00786 | 15 |
| 23567-24166 | 23866 | 0.00346 | 0.00681 | 13 |
| 23767-24366 | 24066 | 0.00418 | 0.00839 | 16 |
| 23967-24566 | 24266 | 0.0037 | 0.00734 | 14 |
| 24167-24766 | 24466 | 0.00333 | 0.00734 | 14 |
| 24367-24966 | 24666 | 0.00262 | 0.00576 | 11 |
| 24567-25166 | 24866 | 0.00308 | 0.00629 | 12 |
| 24767-25366 | 25066 | 0.00332 | 0.00681 | 13 |
| 24967-25566 | 25266 | 0.00374 | 0.00629 | 12 |
| 25167-25766 | 25466 | 0.00352 | 0.00629 | 12 |
| 25367-25967 | 25666 | 0.00341 | 0.00524 | 10 |
| 25567-26167 | 25867 | 0.00513 | 0.01048 | 20 |
| 25767-26443 | 26067 | 0.007 | 0.01415 | 27 |
| 25968-26685 | 26279 | 0.01046 | 0.01939 | 37 |
| 26168-27058 | 26554 | 0.0135 | 0.02516 | 48 |
| 26444-27266 | 26785 | 0.01348 | 0.02463 | 47 |
| 26686-27479 | 27162 | 0.01168 | 0.02306 | 44 |
| 27059-27698 | 27366 | 0.01079 | 0.02201 | 42 |
| 27267-27903 | 27586 | 0.0119 | 0.02306 | 44 |
| 27480-28109 | 27798 | 0.01119 | 0.02149 | 41 |
| 27699-28320 | 28003 | 0.0104 | 0.01677 | 32 |
| 27904-28540 | 28209 | 0.00985 | 0.01572 | 30 |
| 28110-28755 | 28425 | 0.01009 | 0.01625 | 31 |
| 28321-28968 | 28645 | 0.00894 | 0.01625 | 31 |
| 28541-29238 | 28862 | 0.01266 | 0.01992 | 38 |
| 28756-29469 | 29099 | 0.01222 | 0.01887 | 36 |
| 28969-29700 | 29362 | 0.01123 | 0.01625 | 31 |
| 29239-29906 | 29581 | 0.0067 | 0.00996 | 19 |
| 29470-30136 | 29800 | 0.00892 | 0.01415 | 27 |
| 29701-30398 | 30025 | 0.01071 | 0.01729 | 33 |
| 29907-30636 | 30250 | 0.0122 | 0.02044 | 39 |
| 30137-30843 | 30511 | 0.01042 | 0.01729 | 33 |
| 30399-31087 | 30737 | 0.01165 | 0.01992 | 38 |
| 30637-31307 | 30964 | 0.00855 | 0.01625 | 31 |
| 30844-31507 | 31207 | 0.00723 | 0.01415 | 27 |
| 31088-31726 | 31407 | 0.00683 | 0.01415 | 27 |
| 31308-31964 | 31612 | 0.01037 | 0.01939 | 37 |
| 31508-32198 | 31864 | 0.01267 | 0.02358 | 45 |
| 31727-32399 | 32088 | 0.00989 | 0.01834 | 35 |
| 31965-32622 | 32299 | 0.00998 | 0.01939 | 37 |
| 32199-32827 | 32504 | 0.00951 | 0.01782 | 34 |
| 32400-33034 | 32722 | 0.01053 | 0.01834 | 35 |
| 32623-33343 | 32927 | 0.01125 | 0.01939 | 37 |
| 32828-33657 | 33150 | 0.01747 | 0.02725 | 52 |
| 33035-33872 | 33501 | 0.01756 | 0.0283 | 54 |
| 33344-34075 | 33772 | 0.01476 | 0.02306 | 44 |
| 33658-34275 | 33975 | 0.00636 | 0.01101 | 21 |
| 33873-34475 | 34175 | 0.00449 | 0.00734 | 14 |
| 34076-34675 | 34375 | 0.00295 | 0.00524 | 10 |
| 34276-34875 | 34575 | 0.0033 | 0.00681 | 13 |
| 34476-35075 | 34775 | 0.00286 | 0.00629 | 12 |
| 34676-35275 | 34975 | 0.00214 | 0.00472 | 9 |
| 34876-35475 | 35175 | 0.0019 | 0.00419 | 8 |
| 35076-35675 | 35375 | 0.00143 | 0.00314 | 6 |
| 35276-35875 | 35575 | 0.00214 | 0.00472 | 9 |
| 35476-36075 | 35775 | 0.00167 | 0.00367 | 7 |
| 35676-36275 | 35975 | 0.0019 | 0.00419 | 8 |
| 35876-36475 | 36175 | 0.00095 | 0.0021 | 4 |
| 36076-36680 | 36375 | 0.00251 | 0.00472 | 9 |
| 36276-36884 | 36575 | 0.00299 | 0.00576 | 11 |
| 36476-37119 | 36780 | 0.00658 | 0.01205 | 23 |
| 36681-37319 | 36996 | 0.00502 | 0.00943 | 18 |
| 36885-37543 | 37219 | 0.00621 | 0.01205 | 23 |
| 37120-37754 | 37429 | 0.0065 | 0.01258 | 24 |
| 37320-37957 | 37648 | 0.00947 | 0.01782 | 34 |
| 37544-38157 | 37854 | 0.0078 | 0.01415 | 27 |
| 37755-38357 | 38057 | 0.00463 | 0.00891 | 17 |
| 37958-38557 | 38257 | 0.00167 | 0.00367 | 7 |
| 38158-38757 | 38457 | 0.00286 | 0.00629 | 12 |
| 38358-38957 | 38657 | 0.00326 | 0.00576 | 11 |
| 38558-39157 | 38857 | 0.00278 | 0.00472 | 9 |
| 38758-39357 | 39057 | 0.00203 | 0.00262 | 5 |
| 38958-39557 | 39257 | 0.00187 | 0.00367 | 7 |
| 39158-39757 | 39457 | 0.00187 | 0.00367 | 7 |
| 39358-39957 | 39657 | 0.00095 | 0.0021 | 4 |
| 39558-40157 | 39857 | 0.00095 | 0.0021 | 4 |
| 39758-40357 | 40057 | 0.00143 | 0.00314 | 6 |
| 39958-40557 | 40257 | 0.00306 | 0.00629 | 12 |
| 40158-40757 | 40457 | 0.00258 | 0.00524 | 10 |
| 40358-40957 | 40657 | 0.00211 | 0.00419 | 8 |
| 40558-41157 | 40857 | 0.00071 | 0.00157 | 3 |
| 40758-41357 | 41057 | 0.00048 | 0.00105 | 2 |
| 40958-41557 | 41257 | 0.00143 | 0.00314 | 6 |
| 41158-41757 | 41457 | 0.00143 | 0.00314 | 6 |
| 41358-41957 | 41657 | 0.00143 | 0.00314 | 6 |
| 41558-42157 | 41857 | 0.00143 | 0.00314 | 6 |
| 41758-42357 | 42057 | 0.00214 | 0.00472 | 9 |
| 41958-42557 | 42257 | 0.00214 | 0.00472 | 9 |
| 42158-42757 | 42457 | 0.00297 | 0.00524 | 10 |
| 42358-42957 | 42657 | 0.00269 | 0.00419 | 8 |
| 42558-43172 | 42857 | 0.00306 | 0.00419 | 8 |
| 42758-43381 | 43057 | 0.00342 | 0.00419 | 8 |
| 42958-43597 | 43272 | 0.00625 | 0.00996 | 19 |
| 43173-43803 | 43486 | 0.00857 | 0.01415 | 27 |
| 43382-44003 | 43699 | 0.00727 | 0.01258 | 24 |
| 43598-44211 | 43903 | 0.00564 | 0.00943 | 18 |
| 43804-44411 | 44111 | 0.00295 | 0.00524 | 10 |
| 44004-44628 | 44311 | 0.00341 | 0.00576 | 11 |
| 44212-44845 | 44511 | 0.00273 | 0.00472 | 9 |
| 44412-45045 | 44745 | 0.00341 | 0.00576 | 11 |
| 44629-45251 | 44945 | 0.00438 | 0.00734 | 14 |
| 44846-45451 | 45146 | 0.00505 | 0.00839 | 16 |
| 45046-45652 | 45351 | 0.00628 | 0.01153 | 22 |
| 45252-45853 | 45551 | 0.0054 | 0.01101 | 21 |
| 45452-46065 | 45752 | 0.00722 | 0.01415 | 27 |
| 45653-46287 | 45955 | 0.0081 | 0.0152 | 29 |
| 45854-46558 | 46165 | 0.01364 | 0.02358 | 45 |
| 46066-46760 | 46450 | 0.01115 | 0.01939 | 37 |
| 46288-46989 | 46660 | 0.01099 | 0.01992 | 38 |
| 46559-47215 | 46871 | 0.00817 | 0.0152 | 29 |
| 46761-47415 | 47111 | 0.00806 | 0.01415 | 27 |
| 47001-47615 | 47315 | 0.00592 | 0.00943 | 18 |
| 47216-47820 | 47515 | 0.00434 | 0.00734 | 14 |
| 47416-48023 | 47715 | 0.0067 | 0.01205 | 23 |
| 47616-48270 | 47922 | 0.00784 | 0.0131 | 25 |
| 47821-48495 | 48170 | 0.00844 | 0.01363 | 26 |
| 48024-48721 | 48384 | 0.01095 | 0.01729 | 33 |
| 48271-49003 | 48612 | 0.01244 | 0.02201 | 42 |
| 48496-49209 | 48821 | 0.0102 | 0.01887 | 36 |
| 48722-49419 | 49103 | 0.00496 | 0.01048 | 20 |
| 49004-49623 | 49319 | 0.0037 | 0.00681 | 13 |
| 49210-49859 | 49519 | 0.00641 | 0.01101 | 21 |
| 49420-50117 | 49729 | 0.00806 | 0.01415 | 27 |
| 49624-50353 | 50017 | 0.00846 | 0.01467 | 28 |
| 49860-50572 | 50248 | 0.01022 | 0.01572 | 30 |
| 50118-50778 | 50471 | 0.01152 | 0.01782 | 34 |
| 50354-50978 | 50672 | 0.01 | 0.01572 | 30 |
| 50573-51178 | 50878 | 0.00557 | 0.01101 | 21 |
| 50779-51378 | 51078 | 0.00286 | 0.00629 | 12 |
| 50979-51591 | 51278 | 0.00286 | 0.00629 | 12 |
| 51179-51791 | 51491 | 0.00214 | 0.00472 | 9 |
| 51379-51991 | 51691 | 0.00167 | 0.00367 | 7 |
| 51592-52205 | 51891 | 0.00518 | 0.00681 | 13 |
| 51792-52405 | 52091 | 0.00566 | 0.00786 | 15 |
| 51992-52605 | 52305 | 0.0081 | 0.01101 | 21 |
| 52206-52814 | 52505 | 0.00692 | 0.01258 | 24 |
| 52406-53038 | 52705 | 0.01223 | 0.02254 | 43 |
| 52606-53265 | 52914 | 0.01099 | 0.02201 | 42 |
| 52815-53492 | 53150 | 0.01258 | 0.02254 | 43 |
| 53039-53724 | 53380 | 0.01218 | 0.01939 | 37 |
| 53266-53940 | 53594 | 0.01183 | 0.01782 | 34 |
| 53493-54141 | 53831 | 0.00789 | 0.01258 | 24 |
| 53725-54349 | 54040 | 0.00542 | 0.00891 | 17 |
| 53941-54555 | 54243 | 0.00668 | 0.01205 | 23 |
| 54142-54767 | 54454 | 0.00784 | 0.01415 | 27 |
| 54350-54992 | 54655 | 0.00863 | 0.01572 | 30 |
| 54556-55192 | 54877 | 0.00604 | 0.01048 | 20 |
| 54768-55392 | 55092 | 0.00441 | 0.00734 | 14 |
| 54993-55592 | 55292 | 0.00095 | 0.0021 | 4 |
| 55193-55792 | 55492 | 0.00143 | 0.00314 | 6 |
| 55393-55992 | 55692 | 0.00143 | 0.00314 | 6 |
| 55593-56192 | 55892 | 0.00163 | 0.00314 | 6 |
| 55793-56392 | 56092 | 0.00344 | 0.00524 | 10 |
| 55993-56592 | 56292 | 0.00392 | 0.00629 | 12 |
| 56193-56792 | 56492 | 0.00372 | 0.00629 | 12 |
| 56393-57004 | 56692 | 0.00333 | 0.00734 | 14 |
| 56593-57215 | 56904 | 0.00555 | 0.00996 | 19 |
| 56793-57420 | 57104 | 0.00507 | 0.00891 | 17 |
| 57005-57633 | 57320 | 0.00425 | 0.00629 | 12 |
| 57216-57833 | 57533 | 0.00227 | 0.00419 | 8 |
| 57421-58033 | 57733 | 0.00275 | 0.00524 | 10 |
| 57634-58233 | 57933 | 0.00211 | 0.00419 | 8 |
| 57834-58433 | 58133 | 0.00187 | 0.00367 | 7 |
| 58034-58633 | 58333 | 0.00187 | 0.00367 | 7 |
| 58234-58833 | 58533 | 0.00238 | 0.00524 | 10 |
| 58434-59037 | 58733 | 0.00214 | 0.00472 | 9 |
| 58634-59269 | 58933 | 0.00449 | 0.00943 | 18 |
| 58834-59529 | 59163 | 0.00473 | 0.00996 | 19 |
| 59038-59739 | 59399 | 0.00473 | 0.00996 | 19 |
| 59270-59946 | 59629 | 0.00452 | 0.00996 | 19 |
| 59530-60146 | 59846 | 0.00681 | 0.01363 | 26 |
| 59740-60349 | 60046 | 0.008 | 0.01625 | 31 |
| 59947-60549 | 60249 | 0.00811 | 0.0152 | 29 |
| 60147-60749 | 60449 | 0.00756 | 0.01363 | 26 |
| 60350-60949 | 60649 | 0.00709 | 0.01258 | 24 |
| 60550-61149 | 60849 | 0.00637 | 0.01101 | 21 |
| 60750-61362 | 61049 | 0.00749 | 0.01153 | 22 |
| 60950-61616 | 61249 | 0.00962 | 0.01363 | 26 |
| 61150-61828 | 61507 | 0.01141 | 0.01572 | 30 |
| 61363-62030 | 61716 | 0.00951 | 0.0152 | 29 |
| 61617-62344 | 61930 | 0.01075 | 0.01782 | 34 |
| 61829-62544 | 62242 | 0.00857 | 0.01572 | 30 |
| 62031-62744 | 62444 | 0.00738 | 0.0131 | 25 |
| 62345-62944 | 62644 | 0.00484 | 0.00891 | 17 |
| 62545-63261 | 62844 | 0.00771 | 0.0152 | 29 |
| 62745-63570 | 63044 | 0.00914 | 0.01834 | 35 |
| 62945-63785 | 63451 | 0.01108 | 0.02096 | 40 |
| 63262-63998 | 63673 | 0.0107 | 0.01887 | 36 |
| 63571-64204 | 63898 | 0.01022 | 0.01572 | 30 |
| 63799-64404 | 64104 | 0.00694 | 0.01101 | 21 |
| 63999-64604 | 64304 | 0.00374 | 0.00524 | 10 |
| 64205-64804 | 64504 | 0.00302 | 0.00576 | 11 |
| 64405-65004 | 64704 | 0.00346 | 0.00576 | 11 |
| 64605-65204 | 64904 | 0.00326 | 0.00576 | 11 |
| 64805-65404 | 65104 | 0.00255 | 0.00419 | 8 |
| 65005-65604 | 65304 | 0.00214 | 0.00472 | 9 |
| 65205-66064 | 65504 | 0.0031 | 0.00681 | 13 |
| 65405-66270 | 65704 | 0.00621 | 0.01153 | 22 |
| 65605-66471 | 66170 | 0.00597 | 0.01101 | 21 |
| 66065-66671 | 66370 | 0.00562 | 0.00943 | 18 |
| 66271-66871 | 66571 | 0.00311 | 0.00524 | 10 |
| 66472-67071 | 66771 | 0.00355 | 0.00576 | 11 |
| 66672-67290 | 66971 | 0.00505 | 0.00943 | 18 |
| 66872-67549 | 67179 | 0.00896 | 0.01729 | 33 |
| 67072-67761 | 67433 | 0.01097 | 0.02044 | 39 |
| 67291-67977 | 67661 | 0.01379 | 0.02516 | 48 |
| 67550-68203 | 67861 | 0.01377 | 0.02358 | 45 |
| 67762-68406 | 68092 | 0.01295 | 0.02306 | 44 |
| 67978-68606 | 68303 | 0.00897 | 0.01625 | 31 |
| 68204-68819 | 68506 | 0.00592 | 0.01258 | 24 |
| 68407-69036 | 68719 | 0.00656 | 0.01363 | 26 |
| 68607-69293 | 68929 | 0.0074 | 0.01467 | 28 |
| 68820-69718 | 69136 | 0.01907 | 0.03197 | 61 |
| 69037-69954 | 69443 | 0.02158 | 0.03564 | 68 |
| 69294-70170 | 69854 | 0.02353 | 0.03826 | 73 |
| 69719-70391 | 70055 | 0.01306 | 0.02358 | 45 |
| 69955-70620 | 70279 | 0.0113 | 0.01939 | 37 |
| 70173-70853 | 70520 | 0.01159 | 0.01834 | 35 |
| 70392-71053 | 70720 | 0.01212 | 0.0152 | 29 |
| 70621-71253 | 70953 | 0.00958 | 0.01258 | 24 |
| 70854-71474 | 71153 | 0.01478 | 0.01729 | 33 |
| 71054-71674 | 71369 | 0.0135 | 0.01834 | 35 |
| 71254-71874 | 71574 | 0.01299 | 0.01677 | 32 |
| 71475-72091 | 71774 | 0.00445 | 0.00891 | 17 |
| 71675-72317 | 71974 | 0.00462 | 0.00681 | 13 |
| 71875-72522 | 72211 | 0.00489 | 0.00786 | 15 |
| 72092-72743 | 72417 | 0.00441 | 0.00681 | 13 |
| 72318-72949 | 72643 | 0.00582 | 0.01048 | 20 |
| 72523-73155 | 72847 | 0.00623 | 0.00996 | 19 |
| 72744-73365 | 73055 | 0.00718 | 0.01205 | 23 |
| 72950-73570 | 73260 | 0.00766 | 0.01101 | 21 |
| 73156-73778 | 73469 | 0.00888 | 0.01467 | 28 |
| 73366-73989 | 73678 | 0.00841 | 0.01363 | 26 |
| 73571-74189 | 73889 | 0.0057 | 0.01101 | 21 |
| 73779-74429 | 74089 | 0.00608 | 0.00996 | 19 |
| 73990-74636 | 74312 | 0.00679 | 0.01153 | 22 |
| 74190-74859 | 74535 | 0.00725 | 0.01363 | 26 |
| 74430-75064 | 74738 | 0.00659 | 0.0131 | 25 |
| 74637-75272 | 74959 | 0.00656 | 0.01258 | 24 |
| 74860-75472 | 75164 | 0.00839 | 0.01363 | 26 |
| 75065-75678 | 75372 | 0.00782 | 0.01258 | 24 |
| 75273-75878 | 75578 | 0.0069 | 0.01101 | 21 |
| 75473-76078 | 75778 | 0.00471 | 0.00786 | 15 |
| 75679-76278 | 75978 | 0.00368 | 0.00681 | 13 |
| 75879-76478 | 76178 | 0.00368 | 0.00681 | 13 |
| 76079-76678 | 76378 | 0.00286 | 0.00629 | 12 |
| 76279-76878 | 76578 | 0.00262 | 0.00576 | 11 |
| 76479-77078 | 76778 | 0.00167 | 0.00367 | 7 |
| 76679-77278 | 76978 | 0.00214 | 0.00472 | 9 |
| 76879-77484 | 77178 | 0.00533 | 0.00891 | 17 |
| 77079-77684 | 77384 | 0.00604 | 0.01048 | 20 |
| 77279-77884 | 77584 | 0.00553 | 0.00891 | 17 |
| 77485-78091 | 77784 | 0.00445 | 0.00891 | 17 |
| 77685-78303 | 77984 | 0.00619 | 0.01101 | 21 |
| 77885-78513 | 78193 | 0.00815 | 0.0131 | 25 |
| 78092-78718 | 78408 | 0.00839 | 0.01101 | 21 |
| 78304-78923 | 78615 | 0.00793 | 0.01048 | 20 |
| 78514-79123 | 78823 | 0.00529 | 0.00734 | 14 |
| 78719-79323 | 79023 | 0.00366 | 0.00681 | 13 |
| 78924-79523 | 79223 | 0.00238 | 0.00524 | 10 |
| 79124-79739 | 79423 | 0.00401 | 0.00839 | 16 |
| 79324-79939 | 79639 | 0.00544 | 0.01153 | 22 |
| 79524-80145 | 79839 | 0.00544 | 0.01153 | 22 |
| 79740-80360 | 80039 | 0.007 | 0.01363 | 26 |
| 79940-80560 | 80245 | 0.00557 | 0.01048 | 20 |
| 80146-80760 | 80460 | 0.00676 | 0.0131 | 25 |
| 80361-80960 | 80660 | 0.0056 | 0.01153 | 22 |
| 80561-81197 | 80860 | 0.00907 | 0.01677 | 32 |
| 80761-81397 | 81065 | 0.00923 | 0.01625 | 31 |
| 80961-81597 | 81297 | 0.0091 | 0.01677 | 32 |
| 81198-81797 | 81497 | 0.00707 | 0.01467 | 28 |
| 81398-81997 | 81697 | 0.00791 | 0.01467 | 28 |
| 81598-82197 | 81897 | 0.00875 | 0.01415 | 27 |
| 81798-82404 | 82097 | 0.00661 | 0.00943 | 18 |
| 81998-82604 | 82304 | 0.00489 | 0.00839 | 16 |
| 82198-82804 | 82504 | 0.00405 | 0.00891 | 17 |
| 82405-83005 | 82704 | 0.00549 | 0.01101 | 21 |
| 82605-83210 | 82904 | 0.00573 | 0.01153 | 22 |
| 82805-83414 | 83110 | 0.00709 | 0.01363 | 26 |
| 83006-83614 | 83310 | 0.00821 | 0.01415 | 27 |
| 83211-83814 | 83514 | 0.00868 | 0.0152 | 29 |
| 83415-84086 | 83714 | 0.01181 | 0.01782 | 34 |
| 83615-84286 | 83957 | 0.00901 | 0.01467 | 28 |
| 83815-84492 | 84186 | 0.00996 | 0.01677 | 32 |
| 84087-84696 | 84386 | 0.00654 | 0.0131 | 25 |
| 84287-84896 | 84596 | 0.00797 | 0.01625 | 31 |
| 84493-85103 | 84796 | 0.00623 | 0.01101 | 21 |
| 84697-85403 | 84996 | 0.0133 | 0.02149 | 41 |
| 84897-85623 | 85217 | 0.01449 | 0.02411 | 46 |
| 85104-85841 | 85523 | 0.0167 | 0.0304 | 58 |
| 85404-86075 | 85733 | 0.00863 | 0.01729 | 33 |
| 85624-86284 | 85951 | 0.01018 | 0.01939 | 37 |
| 85842-86486 | 86177 | 0.00967 | 0.01782 | 34 |
| 86076-86686 | 86386 | 0.01084 | 0.01939 | 37 |
| 86287-86892 | 86586 | 0.00806 | 0.01415 | 27 |
| 86487-87107 | 86792 | 0.00667 | 0.01153 | 22 |
| 86687-87307 | 86992 | 0.00462 | 0.00891 | 17 |
| 86893-87507 | 87207 | 0.00441 | 0.00891 | 17 |
| 87108-87714 | 87407 | 0.00463 | 0.00891 | 17 |
| 87308-87914 | 87607 | 0.00522 | 0.01101 | 21 |
| 87508-88116 | 87814 | 0.00518 | 0.01048 | 20 |
| 87715-88316 | 88016 | 0.0033 | 0.00681 | 13 |
| 87915-88516 | 88216 | 0.00139 | 0.00262 | 5 |
| 88117-88716 | 88416 | 0.00024 | 0.00052 | 1 |
| 88317-88916 | 88616 | 0.00048 | 0.00105 | 2 |
| 88517-89116 | 88816 | 0.00071 | 0.00157 | 3 |
| 88717-89316 | 89016 | 0.00095 | 0.0021 | 4 |
| 88917-89516 | 89216 | 0.00071 | 0.00157 | 3 |
| 89117-89716 | 89416 | 0.00048 | 0.00105 | 2 |
| 89317-89916 | 89616 | 0.00048 | 0.00105 | 2 |
| 89517-90116 | 89816 | 0.00048 | 0.00105 | 2 |
| 89717-90316 | 90016 | 0.00095 | 0.0021 | 4 |
| 89917-90516 | 90216 | 0.00071 | 0.00157 | 3 |
| 90117-90716 | 90416 | 0.00207 | 0.00314 | 6 |
| 90317-90916 | 90616 | 0.00231 | 0.00367 | 7 |
| 90517-91116 | 90816 | 0.00207 | 0.00314 | 6 |
| 90717-91319 | 91016 | 0.00071 | 0.00157 | 3 |
| 90917-91519 | 91219 | 0 | 0 | 0 |
| 91117-91719 | 91419 | 0.00024 | 0.00052 | 1 |
| 91320-91919 | 91619 | 0.00095 | 0.0021 | 4 |
| 91520-92119 | 91819 | 0.00119 | 0.00262 | 5 |
| 91720-92319 | 92019 | 0.00119 | 0.00262 | 5 |
| 91920-92519 | 92219 | 0.00048 | 0.00105 | 2 |
| 92120-92719 | 92419 | 0.00024 | 0.00052 | 1 |
| 92320-92919 | 92619 | 0.00068 | 0.00105 | 2 |
| 92520-93119 | 92819 | 0.00211 | 0.00419 | 8 |
| 92720-93319 | 93019 | 0.00211 | 0.00419 | 8 |
| 92920-93519 | 93219 | 0.00255 | 0.00472 | 9 |
| 93120-93719 | 93419 | 0.00112 | 0.00157 | 3 |
| 93320-93919 | 93619 | 0.00159 | 0.00262 | 5 |
| 93520-94119 | 93819 | 0.00095 | 0.0021 | 4 |
| 93720-94319 | 94019 | 0.00095 | 0.0021 | 4 |
| 93920-94519 | 94219 | 0.00071 | 0.00157 | 3 |
| 94120-94725 | 94419 | 0.00048 | 0.00105 | 2 |
| 94320-94925 | 94619 | 0.00071 | 0.00157 | 3 |
| 94520-95125 | 94825 | 0.00163 | 0.00314 | 6 |
| 94726-95325 | 95025 | 0.00163 | 0.00314 | 6 |
| 94926-95525 | 95225 | 0.00187 | 0.00367 | 7 |
| 95126-95725 | 95425 | 0.0019 | 0.00419 | 8 |
| 95326-95937 | 95625 | 0.00214 | 0.00472 | 9 |
| 95526-96137 | 95837 | 0.00167 | 0.00367 | 7 |
| 95726-96337 | 96037 | 0.00095 | 0.0021 | 4 |
| 95938-96537 | 96237 | 0.00071 | 0.00157 | 3 |
| 96138-96737 | 96437 | 0.00095 | 0.0021 | 4 |
| 96338-96947 | 96637 | 0.00207 | 0.00314 | 6 |
| 96538-97154 | 96847 | 0.00207 | 0.00314 | 6 |
| 96738-97360 | 97054 | 0.00255 | 0.00419 | 8 |
| 96948-97568 | 97254 | 0.00095 | 0.0021 | 4 |
| 97155-97768 | 97468 | 0.00071 | 0.00157 | 3 |
| 97361-97968 | 97668 | 0 | 0 | 0 |
| 97569-98168 | 97868 | 0 | 0 | 0 |
| 97769-98368 | 98068 | 0 | 0 | 0 |
| 97969-98568 | 98268 | 0 | 0 | 0 |
| 98169-98768 | 98468 | 0 | 0 | 0 |
| 98369-98968 | 98668 | 0.00024 | 0.00052 | 1 |
| 98569-99168 | 98868 | 0.00024 | 0.00052 | 1 |
| 98769-99368 | 99068 | 0.00024 | 0.00052 | 1 |
| 98969-99568 | 99268 | 0.00048 | 0.00105 | 2 |
| 99169-99768 | 99468 | 0.00071 | 0.00157 | 3 |
| 99369-99968 | 99668 | 0.00071 | 0.00157 | 3 |
| 99569-100168 | 99868 | 0.00048 | 0.00105 | 2 |
| 99769-100368 | 100068 | 0.00071 | 0.00157 | 3 |
| 99969-100568 | 100268 | 0.00167 | 0.00367 | 7 |
| 100169-100773 | 100468 | 0.00234 | 0.00472 | 9 |
| 100369-100973 | 100668 | 0.00211 | 0.00419 | 8 |
| 100569-101173 | 100873 | 0.00139 | 0.00262 | 5 |
| 100774-101378 | 101073 | 0.00048 | 0.00105 | 2 |
| 100974-101583 | 101278 | 0.00024 | 0.00052 | 1 |
| 101174-101783 | 101478 | 0.00024 | 0.00052 | 1 |
| 101379-101983 | 101683 | 0.00024 | 0.00052 | 1 |
| 101584-102183 | 101883 | 0.00024 | 0.00052 | 1 |
| 101784-102383 | 102083 | 0.00048 | 0.00105 | 2 |
| 101984-102596 | 102283 | 0.00231 | 0.00367 | 7 |
| 102184-102809 | 102487 | 0.00255 | 0.00419 | 8 |
| 102384-103014 | 102701 | 0.00207 | 0.00314 | 6 |
| 102597-103220 | 102914 | 0.00071 | 0.00157 | 3 |
| 102810-103432 | 103120 | 0.00071 | 0.00157 | 3 |
| 103015-103632 | 103332 | 0.00167 | 0.00367 | 7 |
| 103221-103832 | 103532 | 0.0019 | 0.00419 | 8 |
| 103433-104032 | 103732 | 0.00167 | 0.00367 | 7 |
| 103633-104232 | 103932 | 0.00071 | 0.00157 | 3 |
| 103833-104432 | 104132 | 0.00044 | 0.00052 | 1 |
| 104033-104632 | 104332 | 0.00044 | 0.00052 | 1 |
| 104233-104832 | 104532 | 0.00044 | 0.00052 | 1 |
| 104433-105032 | 104732 | 0 | 0 | 0 |
| 104633-105232 | 104932 | 0 | 0 | 0 |
| 104833-105432 | 105132 | 0 | 0 | 0 |
| 105033-105632 | 105332 | 0 | 0 | 0 |
| 105233-105832 | 105532 | 0.00024 | 0.00052 | 1 |
| 105433-106032 | 105732 | 0.00024 | 0.00052 | 1 |
| 105633-106232 | 105932 | 0.00048 | 0.00105 | 2 |
| 105833-106432 | 106132 | 0.00024 | 0.00052 | 1 |
| 106033-106632 | 106332 | 0.00024 | 0.00052 | 1 |
| 106233-106832 | 106532 | 0 | 0 | 0 |
| 106433-107032 | 106732 | 0.00024 | 0.00052 | 1 |
| 106633-107232 | 106932 | 0.00024 | 0.00052 | 1 |
| 106833-107432 | 107132 | 0.00048 | 0.00105 | 2 |
| 107033-107632 | 107332 | 0.00024 | 0.00052 | 1 |
| 107233-107832 | 107532 | 0.00048 | 0.00105 | 2 |
| 107433-108032 | 107732 | 0.00024 | 0.00052 | 1 |
| 107633-108232 | 107932 | 0.00167 | 0.00367 | 7 |
| 107833-108432 | 108132 | 0.00143 | 0.00314 | 6 |
| 108033-108632 | 108332 | 0.00143 | 0.00314 | 6 |
| 108233-108832 | 108532 | 0 | 0 | 0 |
| 108433-109032 | 108732 | 0 | 0 | 0 |
| 108633-109232 | 108932 | 0.00024 | 0.00052 | 1 |
| 108833-109432 | 109132 | 0.00088 | 0.00105 | 2 |
| 109033-109632 | 109332 | 0.00088 | 0.00105 | 2 |
| 109233-109832 | 109532 | 0.00064 | 0.00052 | 1 |
| 109433-110032 | 109732 | 0 | 0 | 0 |
| 109633-110232 | 109932 | 0 | 0 | 0 |
| 109833-110432 | 110132 | 0 | 0 | 0 |
| 110033-110632 | 110332 | 0 | 0 | 0 |
| 110233-110832 | 110532 | 0 | 0 | 0 |
| 110433-111032 | 110732 | 0 | 0 | 0 |
| 110633-111232 | 110932 | 0.00048 | 0.00105 | 2 |
| 110833-111432 | 111132 | 0.00095 | 0.0021 | 4 |
| 111033-111640 | 111332 | 0.00095 | 0.0021 | 4 |
| 111233-111841 | 111532 | 0.00071 | 0.00157 | 3 |
| 111433-112047 | 111741 | 0.00289 | 0.00419 | 8 |
| 111641-112247 | 111942 | 0.00313 | 0.00472 | 9 |
| 111842-112447 | 112147 | 0.00641 | 0.00629 | 12 |
| 112048-112647 | 112347 | 0.00399 | 0.00314 | 6 |
| 112248-112847 | 112547 | 0.00399 | 0.00314 | 6 |
| 112448-113047 | 112747 | 0.00095 | 0.0021 | 4 |
| 112648-113247 | 112947 | 0.00095 | 0.0021 | 4 |
| 112848-113447 | 113147 | 0.00095 | 0.0021 | 4 |
| 113048-113668 | 113347 | 0.00119 | 0.00262 | 5 |
| 113248-113868 | 113547 | 0.00119 | 0.00262 | 5 |
| 113448-114580 | 113768 | 0.01527 | 0.03145 | 60 |
| 113669-114780 | 114225 | 0.01855 | 0.03773 | 72 |
| 113869-114980 | 114680 | 0.02201 | 0.04455 | 85 |
| 114581-115180 | 114880 | 0.01051 | 0.02096 | 40 |
| 114781-115386 | 115080 | 0.01066 | 0.02044 | 39 |
| 114981-115586 | 115286 | 0.00951 | 0.01782 | 34 |
| 115181-115786 | 115486 | 0.0074 | 0.01363 | 26 |
| 115387-115986 | 115686 | 0.00374 | 0.00734 | 14 |
| 115587-116186 | 115886 | 0.00401 | 0.00839 | 16 |
| 115787-116386 | 116086 | 0.00443 | 0.00786 | 15 |
| 115987-116586 | 116286 | 0.00515 | 0.00943 | 18 |
| 116187-116786 | 116486 | 0.00619 | 0.00996 | 19 |
| 116387-117023 | 116686 | 0.01077 | 0.01887 | 36 |
| 116587-117547 | 116908 | 0.01826 | 0.02725 | 52 |
| 116787-117766 | 117441 | 0.01868 | 0.0304 | 58 |
| 117024-117973 | 117666 | 0.01511 | 0.02516 | 48 |
| 117548-118173 | 117872 | 0.00989 | 0.01992 | 38 |
| 117767-118373 | 118073 | 0.00744 | 0.0131 | 25 |
| 117974-118573 | 118273 | 0.01196 | 0.01992 | 38 |
| 118174-118791 | 118473 | 0.01112 | 0.01782 | 34 |
| 118374-119000 | 118673 | 0.01163 | 0.01992 | 38 |
| 118574-119302 | 118891 | 0.01009 | 0.01834 | 35 |
| 118792-119502 | 119201 | 0.01125 | 0.02096 | 40 |
| 119001-119702 | 119402 | 0.01026 | 0.01782 | 34 |
| 119303-119902 | 119602 | 0.00727 | 0.01258 | 24 |
| 119503-120102 | 119802 | 0.00458 | 0.00786 | 15 |
| 119703-120302 | 120002 | 0.00462 | 0.00891 | 17 |
| 119903-120502 | 120202 | 0.0039 | 0.00734 | 14 |
| 120103-120702 | 120402 | 0.00599 | 0.01048 | 20 |
| 120303-120904 | 120602 | 0.00661 | 0.01101 | 21 |
| 120503-121104 | 120804 | 0.00733 | 0.01258 | 24 |
| 120703-121316 | 121004 | 0.00559 | 0.01101 | 21 |
| 120905-121525 | 121216 | 0.00727 | 0.01363 | 26 |
| 121105-121739 | 121425 | 0.00727 | 0.01363 | 26 |
| 121317-121987 | 121625 | 0.00995 | 0.01572 | 30 |
| 121526-122187 | 121887 | 0.00764 | 0.01258 | 24 |
| 121740-122387 | 122087 | 0.00711 | 0.01048 | 20 |
| 121988-122668 | 122287 | 0.00758 | 0.01205 | 23 |
| 122188-122873 | 122509 | 0.00877 | 0.01467 | 28 |
| 122388-123073 | 122769 | 0.00973 | 0.0152 | 29 |
| 122669-123273 | 122973 | 0.00694 | 0.01153 | 22 |
| 122874-123473 | 123173 | 0.00639 | 0.00996 | 19 |
| 123074-123673 | 123373 | 0.00478 | 0.00891 | 17 |
| 123274-123873 | 123573 | 0.00451 | 0.00786 | 15 |
| 123474-124078 | 123773 | 0.0039 | 0.00734 | 14 |
| 123674-124294 | 123973 | 0.00625 | 0.01205 | 23 |
| 123874-124507 | 124182 | 0.00714 | 0.0131 | 25 |
| 124079-124709 | 124407 | 0.00883 | 0.01363 | 26 |
| 124295-124921 | 124609 | 0.008 | 0.01101 | 21 |
| 124508-125126 | 124809 | 0.00725 | 0.01153 | 22 |
| 124710-125326 | 125026 | 0.00553 | 0.01048 | 20 |
| 124922-125538 | 125226 | 0.00401 | 0.00839 | 16 |
| 125127-125738 | 125426 | 0.00366 | 0.00681 | 13 |
| 125327-125938 | 125638 | 0.00346 | 0.00681 | 13 |
| 125539-126138 | 125838 | 0.0037 | 0.00734 | 14 |
| 125739-126338 | 126038 | 0.00452 | 0.00996 | 19 |
| 125939-126538 | 126238 | 0.00381 | 0.00839 | 16 |
| 126139-126739 | 126438 | 0.00403 | 0.00839 | 16 |
| 126339-126986 | 126638 | 0.00674 | 0.00943 | 18 |
| 126539-127195 | 126886 | 0.0085 | 0.01205 | 23 |
| 126740-127448 | 127086 | 0.01399 | 0.01939 | 37 |
| 126987-127649 | 127316 | 0.0124 | 0.01992 | 38 |
| 127196-127849 | 127549 | 0.0146 | 0.02463 | 47 |
| 127449-128061 | 127749 | 0.01092 | 0.02044 | 39 |
| 127650-128261 | 127955 | 0.01363 | 0.02254 | 43 |
| 127850-128461 | 128161 | 0.01484 | 0.02358 | 45 |
| 128062-128661 | 128361 | 0.01764 | 0.0283 | 54 |
| 128262-128867 | 128561 | 0.01747 | 0.02778 | 53 |
| 128462-129073 | 128767 | 0.01495 | 0.02358 | 45 |
| 128668-129279 | 128967 | 0.015 | 0.02306 | 44 |
| 128868-129500 | 129173 | 0.01181 | 0.02096 | 40 |
| 129074-129715 | 129385 | 0.01676 | 0.0304 | 58 |
| 129280-129915 | 129600 | 0.01648 | 0.02778 | 53 |
| 129501-130115 | 129815 | 0.02114 | 0.03407 | 65 |
| 129716-130321 | 130015 | 0.01941 | 0.0283 | 54 |
| 129916-130539 | 130215 | 0.01952 | 0.03145 | 60 |
| 130116-130739 | 130421 | 0.01678 | 0.0262 | 50 |
| 130322-130985 | 130639 | 0.01989 | 0.03407 | 65 |
| 130540-131185 | 130855 | 0.01822 | 0.03249 | 62 |
| 130740-131406 | 131085 | 0.01817 | 0.03459 | 66 |
| 130986-131606 | 131285 | 0.01158 | 0.02411 | 46 |
| 131186-131806 | 131506 | 0.00852 | 0.01782 | 34 |
| 131407-132006 | 131706 | 0.00511 | 0.00996 | 19 |
| 131607-132206 | 131906 | 0.005 | 0.00891 | 17 |
| 131807-132427 | 132106 | 0.00429 | 0.00734 | 14 |
| 132007-132627 | 132306 | 0.0037 | 0.00734 | 14 |
| 132207-132827 | 132527 | 0.00119 | 0.00262 | 5 |
| 132428-133027 | 132727 | 0.00119 | 0.00262 | 5 |
| 132628-133227 | 132927 | 0.00095 | 0.0021 | 4 |
| 132828-133427 | 133127 | 0.00095 | 0.0021 | 4 |
| 133028-133627 | 133327 | 0.00399 | 0.00314 | 6 |
| 133228-133827 | 133527 | 0.00375 | 0.00262 | 5 |
| 133428-134032 | 133727 | 0.00617 | 0.00576 | 11 |
| 133628-134234 | 133927 | 0.00313 | 0.00472 | 9 |
| 133828-134442 | 134133 | 0.00313 | 0.00472 | 9 |
| 134033-134642 | 134334 | 0.00071 | 0.00157 | 3 |
| 134235-134842 | 134542 | 0.00095 | 0.0021 | 4 |
| 134443-135042 | 134742 | 0.00095 | 0.0021 | 4 |
| 134643-135242 | 134942 | 0.00071 | 0.00157 | 3 |
| 134843-135442 | 135142 | 0 | 0 | 0 |
| 135043-135642 | 135342 | 0 | 0 | 0 |
| 135243-135842 | 135542 | 0 | 0 | 0 |
| 135443-136042 | 135742 | 0 | 0 | 0 |
| 135643-136242 | 135942 | 0 | 0 | 0 |
| 135843-136442 | 136142 | 0 | 0 | 0 |
| 136043-136642 | 136342 | 0.00064 | 0.00052 | 1 |
| 136243-136842 | 136542 | 0.00088 | 0.00105 | 2 |
| 136443-137042 | 136742 | 0.00088 | 0.00105 | 2 |
| 136643-137242 | 136942 | 0.00024 | 0.00052 | 1 |
| 136843-137442 | 137142 | 0 | 0 | 0 |
| 137043-137642 | 137342 | 0 | 0 | 0 |
| 137243-137842 | 137542 | 0.00024 | 0.00052 | 1 |
| 137443-138042 | 137742 | 0.00143 | 0.00314 | 6 |
| 137643-138242 | 137942 | 0.00167 | 0.00367 | 7 |
| 137843-138442 | 138142 | 0.00143 | 0.00314 | 6 |
| 138043-138642 | 138342 | 0.00048 | 0.00105 | 2 |
| 138243-138842 | 138542 | 0.00024 | 0.00052 | 1 |
| 138443-139042 | 138742 | 0.00048 | 0.00105 | 2 |
| 138643-139242 | 138942 | 0.00024 | 0.00052 | 1 |
| 138843-139442 | 139142 | 0.00024 | 0.00052 | 1 |
| 139043-139642 | 139342 | 0 | 0 | 0 |
| 139243-139842 | 139542 | 0 | 0 | 0 |
| 139443-140042 | 139742 | 0.00024 | 0.00052 | 1 |
| 139643-140242 | 139942 | 0.00048 | 0.00105 | 2 |
| 139843-140442 | 140142 | 0.00048 | 0.00105 | 2 |
| 140043-140642 | 140342 | 0.00024 | 0.00052 | 1 |
| 140243-140842 | 140542 | 0 | 0 | 0 |
| 140443-141042 | 140742 | 0 | 0 | 0 |
| 140643-141242 | 140942 | 0 | 0 | 0 |
| 140843-141442 | 141142 | 0 | 0 | 0 |
| 141043-141642 | 141342 | 0 | 0 | 0 |
| 141243-141842 | 141542 | 0.00044 | 0.00052 | 1 |
| 141443-142042 | 141742 | 0.00044 | 0.00052 | 1 |
| 141643-142242 | 141942 | 0.00068 | 0.00105 | 2 |
| 141843-142442 | 142142 | 0.00167 | 0.00367 | 7 |
| 142043-142654 | 142342 | 0.0019 | 0.00419 | 8 |
| 142243-142860 | 142542 | 0.00214 | 0.00472 | 9 |
| 142443-143060 | 142754 | 0.00071 | 0.00157 | 3 |
| 142655-143273 | 142960 | 0.00048 | 0.00105 | 2 |
| 142861-143486 | 143172 | 0.00183 | 0.00262 | 5 |
| 143061-143690 | 143383 | 0.00231 | 0.00367 | 7 |
| 143274-143890 | 143590 | 0.00255 | 0.00419 | 8 |
| 143487-144090 | 143790 | 0.00071 | 0.00157 | 3 |
| 143691-144290 | 143990 | 0.00048 | 0.00105 | 2 |
| 143891-144495 | 144190 | 0.00024 | 0.00052 | 1 |
| 144091-144700 | 144395 | 0.00024 | 0.00052 | 1 |
| 144291-144900 | 144595 | 0.00024 | 0.00052 | 1 |
| 144496-145100 | 144800 | 0.00048 | 0.00105 | 2 |
| 144701-145305 | 145000 | 0.00092 | 0.00157 | 3 |
| 144901-145505 | 145205 | 0.00187 | 0.00367 | 7 |
| 145101-145705 | 145405 | 0.00234 | 0.00472 | 9 |
| 145306-145905 | 145605 | 0.00214 | 0.00472 | 9 |
| 145506-146105 | 145805 | 0.00095 | 0.0021 | 4 |
| 145706-146305 | 146005 | 0.00048 | 0.00105 | 2 |
| 145906-146505 | 146205 | 0.00048 | 0.00105 | 2 |
| 146106-146705 | 146405 | 0.00071 | 0.00157 | 3 |
| 146306-146905 | 146605 | 0.00048 | 0.00105 | 2 |
| 146506-147105 | 146805 | 0.00048 | 0.00105 | 2 |
| 146706-147305 | 147005 | 0.00024 | 0.00052 | 1 |
| 146906-147505 | 147205 | 0.00024 | 0.00052 | 1 |
| 147106-147705 | 147405 | 0 | 0 | 0 |
| 147306-147905 | 147605 | 0 | 0 | 0 |
| 147506-148105 | 147805 | 0 | 0 | 0 |
| 147706-148305 | 148005 | 0 | 0 | 0 |
| 147906-148505 | 148205 | 0 | 0 | 0 |
| 148106-148719 | 148405 | 0.00048 | 0.00105 | 2 |
| 148306-148925 | 148613 | 0.00095 | 0.0021 | 4 |
| 148506-149126 | 148819 | 0.00119 | 0.00262 | 5 |
| 148720-149336 | 149026 | 0.00207 | 0.00314 | 6 |
| 148926-149536 | 149236 | 0.00207 | 0.00314 | 6 |
| 149127-149736 | 149436 | 0.00231 | 0.00367 | 7 |
| 149337-149936 | 149636 | 0.00095 | 0.0021 | 4 |
| 149537-150142 | 149836 | 0.00095 | 0.0021 | 4 |
| 149737-150348 | 150036 | 0.00119 | 0.00262 | 5 |
| 149937-150548 | 150248 | 0.00214 | 0.00472 | 9 |
| 150149-150748 | 150448 | 0.0019 | 0.00419 | 8 |
| 150349-150948 | 150648 | 0.00211 | 0.00419 | 8 |
| 150549-151148 | 150848 | 0.00163 | 0.00314 | 6 |
| 150749-151354 | 151048 | 0.00163 | 0.00314 | 6 |
| 150949-151554 | 151248 | 0.00095 | 0.0021 | 4 |
| 151149-151754 | 151454 | 0.00048 | 0.00105 | 2 |
| 151355-151954 | 151654 | 0.00048 | 0.00105 | 2 |
| 151555-152154 | 151854 | 0.00071 | 0.00157 | 3 |
| 151755-152354 | 152054 | 0.00095 | 0.0021 | 4 |
| 151955-152554 | 152254 | 0.00139 | 0.00262 | 5 |
| 152155-152754 | 152454 | 0.00136 | 0.0021 | 4 |
| 152355-152954 | 152654 | 0.00255 | 0.00472 | 9 |
| 152555-153154 | 152854 | 0.00255 | 0.00472 | 9 |
| 152755-153354 | 153054 | 0.00211 | 0.00419 | 8 |
| 152955-153554 | 153254 | 0.00068 | 0.00105 | 2 |
| 153155-153754 | 153454 | 0.00024 | 0.00052 | 1 |
| 153355-153954 | 153654 | 0.00048 | 0.00105 | 2 |
| 153555-154154 | 153854 | 0.00119 | 0.00262 | 5 |
| 153755-154354 | 154054 | 0.00095 | 0.0021 | 4 |
| 153955-154554 | 154254 | 0.00095 | 0.0021 | 4 |
| 154155-154757 | 154454 | 0.00024 | 0.00052 | 1 |
| 154355-154957 | 154654 | 0.00024 | 0.00052 | 1 |
| 154555-155157 | 154857 | 0.00048 | 0.00105 | 2 |
| 154758-155357 | 155057 | 0.00183 | 0.00262 | 5 |
| 154958-155557 | 155257 | 0.00207 | 0.00314 | 6 |
| 155158-155757 | 155457 | 0.00207 | 0.00314 | 6 |
| 155358-155957 | 155657 | 0.00095 | 0.0021 | 4 |
| 155558-156157 | 155857 | 0.00095 | 0.0021 | 4 |
| 155758-156357 | 156057 | 0.00071 | 0.00157 | 3 |
| 155958-156557 | 156257 | 0.00048 | 0.00105 | 2 |
| 156158-156757 | 156457 | 0.00071 | 0.00157 | 3 |
| 156358-156957 | 156657 | 0.00048 | 0.00105 | 2 |
| 156558-157157 | 156857 | 0.00095 | 0.0021 | 4 |
| 156758-157357 | 157057 | 0.00048 | 0.00105 | 2 |
| 156958-157557 | 157257 | 0.00071 | 0.00157 | 3 |
| 157158-157757 | 157457 | 0.00024 | 0.00052 | 1 |
| 157358-158063 | 157579 | 0.00032 | 0.00071 | 1 |
