# Supplementary material for: Complete chloroplast genomes of eight Delphinium taxa (Ranunculaceae) endemic to Xinjiang, China: insights into genome structure, comparative analysis, and phylogenetic relationships
Source: BMC Plant Biol. 2024 Jun 26;24:600. doi: 10.1186/s12870-024-05279-y (PMC11201361; doi:10.1186/s12870-024-05279-y)
Supplement: Supplementary file 5 — Supplementary Material 5 [file 12870_2024_5279_MOESM5_ESM.docx]

**Table S5**. The nucleotide variability (Pi) of 13 *Delphinium* taxa in whole chloroplast genomes.

| Window | Midpoint | Pi | Theta | S |
| --- | --- | --- | --- | --- |
| 1-681 | 381 | 0.0062 | 0.00752 | 14 |
| 282-881 | 581 | 0.00265 | 0.0043 | 8 |
| 482-1081 | 781 | 0.00141 | 0.00215 | 4 |
| 682-1281 | 981 | 0.00188 | 0.00269 | 5 |
| 882-1481 | 1181 | 0.00124 | 0.00215 | 4 |
| 1082-1683 | 1381 | 0.0015 | 0.00269 | 5 |
| 1282-1884 | 1581 | 0.00154 | 0.00322 | 6 |
| 1482-2091 | 1784 | 0.00239 | 0.00376 | 7 |
| 1684-2291 | 1991 | 0.00479 | 0.00644 | 12 |
| 1885-2491 | 2191 | 0.00577 | 0.00806 | 15 |
| 2092-2691 | 2391 | 0.00585 | 0.00859 | 16 |
| 2292-2897 | 2591 | 0.00397 | 0.00698 | 13 |
| 2492-3097 | 2797 | 0.00331 | 0.00483 | 9 |
| 2692-3297 | 2997 | 0.00468 | 0.00698 | 13 |
| 2898-3497 | 3197 | 0.00515 | 0.00752 | 14 |
| 3098-3702 | 3397 | 0.0053 | 0.00859 | 16 |
| 3298-3903 | 3597 | 0.00427 | 0.00806 | 15 |
| 3498-4109 | 3802 | 0.00517 | 0.00752 | 14 |
| 3703-4310 | 4003 | 0.00504 | 0.00644 | 12 |
| 3904-4521 | 4210 | 0.00774 | 0.00859 | 16 |
| 4110-4741 | 4416 | 0.00778 | 0.0102 | 19 |
| 4311-4947 | 4631 | 0.01 | 0.01396 | 26 |
| 4522-5168 | 4841 | 0.00714 | 0.0102 | 19 |
| 4742-5368 | 5068 | 0.00624 | 0.00967 | 18 |
| 4948-5568 | 5268 | 0.00376 | 0.00537 | 10 |
| 5169-5768 | 5468 | 0.00325 | 0.0043 | 8 |
| 5369-5999 | 5668 | 0.00248 | 0.00269 | 5 |
| 5569-6203 | 5888 | 0.0041 | 0.00483 | 9 |
| 5769-6404 | 6103 | 0.005 | 0.00591 | 11 |
| 6000-6615 | 6303 | 0.00449 | 0.00483 | 9 |
| 6204-6838 | 6504 | 0.00675 | 0.00913 | 17 |
| 6405-7062 | 6729 | 0.00624 | 0.00967 | 18 |
| 6616-7267 | 6950 | 0.00765 | 0.01182 | 22 |
| 6839-7467 | 7167 | 0.00368 | 0.00644 | 12 |
| 7063-7696 | 7367 | 0.00769 | 0.01074 | 20 |
| 7268-7896 | 7581 | 0.00654 | 0.00913 | 17 |
| 7468-8098 | 7796 | 0.00628 | 0.00859 | 16 |
| 7697-8306 | 7998 | 0.00406 | 0.00537 | 10 |
| 7897-8515 | 8205 | 0.00466 | 0.00537 | 10 |
| 8099-8715 | 8415 | 0.00415 | 0.0043 | 8 |
| 8307-8915 | 8615 | 0.00137 | 0.00161 | 3 |
| 8516-9115 | 8815 | 0.00077 | 0.00161 | 3 |
| 8716-9315 | 9015 | 0.00103 | 0.00215 | 4 |
| 8916-9515 | 9215 | 0.00162 | 0.00215 | 4 |
| 9116-9715 | 9415 | 0.00269 | 0.00269 | 5 |
| 9316-9915 | 9615 | 0.00269 | 0.00269 | 5 |
| 9516-10115 | 9815 | 0.00308 | 0.0043 | 8 |
| 9716-10315 | 10015 | 0.00376 | 0.00537 | 10 |
| 9916-10515 | 10215 | 0.0035 | 0.00483 | 9 |
| 10116-10719 | 10415 | 0.00278 | 0.00376 | 7 |
| 10316-10929 | 10615 | 0.00205 | 0.0043 | 8 |
| 10516-11129 | 10824 | 0.00342 | 0.00591 | 11 |
| 10720-11335 | 11029 | 0.00342 | 0.00591 | 11 |
| 10930-11549 | 11235 | 0.00261 | 0.00376 | 7 |
| 11130-11750 | 11435 | 0.00427 | 0.00537 | 10 |
| 11336-11950 | 11650 | 0.00449 | 0.00537 | 10 |
| 11550-12174 | 11850 | 0.00376 | 0.0043 | 8 |
| 11751-12392 | 12050 | 0.00073 | 0.00107 | 2 |
| 11951-12630 | 12278 | 0.00026 | 0.00054 | 1 |
| 12175-12830 | 12502 | 0.00308 | 0.0043 | 8 |
| 12393-13069 | 12730 | 0.00594 | 0.00859 | 16 |
| 12631-13271 | 12930 | 0.00748 | 0.01182 | 22 |
| 12831-13471 | 13171 | 0.00624 | 0.00967 | 18 |
| 13070-13671 | 13371 | 0.00423 | 0.00591 | 11 |
| 13272-13871 | 13571 | 0.00244 | 0.00215 | 4 |
| 13472-14081 | 13771 | 0.00222 | 0.00215 | 4 |
| 13672-14287 | 13971 | 0.00137 | 0.00161 | 3 |
| 13872-14487 | 14187 | 0.00248 | 0.00269 | 5 |
| 14082-14687 | 14387 | 0.00137 | 0.00161 | 3 |
| 14288-14888 | 14587 | 0.00188 | 0.00269 | 5 |
| 14488-15095 | 14787 | 0.00167 | 0.00269 | 5 |
| 14688-15295 | 14995 | 0.00167 | 0.00269 | 5 |
| 14889-15495 | 15195 | 0.00115 | 0.00161 | 3 |
| 15096-15695 | 15395 | 0.00098 | 0.00161 | 3 |
| 15296-15895 | 15595 | 0.00073 | 0.00107 | 2 |
| 15496-16095 | 15795 | 0.00145 | 0.00215 | 4 |
| 15696-16295 | 15995 | 0.00145 | 0.00215 | 4 |
| 15896-16495 | 16195 | 0.00321 | 0.00376 | 7 |
| 16096-16695 | 16395 | 0.00363 | 0.0043 | 8 |
| 16296-16895 | 16595 | 0.00291 | 0.00322 | 6 |
| 16496-17095 | 16795 | 0.00214 | 0.00322 | 6 |
| 16696-17298 | 16995 | 0.00124 | 0.00215 | 4 |
| 16896-17498 | 17198 | 0.0015 | 0.00269 | 5 |
| 17096-17698 | 17398 | 0.0041 | 0.00483 | 9 |
| 17299-17898 | 17598 | 0.0041 | 0.00483 | 9 |
| 17499-18098 | 17798 | 0.00543 | 0.00591 | 11 |
| 17699-18298 | 17998 | 0.00209 | 0.00269 | 5 |
| 17899-18498 | 18198 | 0.00235 | 0.00322 | 6 |
| 18099-18698 | 18398 | 0.00077 | 0.00161 | 3 |
| 18299-18898 | 18598 | 0.00051 | 0.00107 | 2 |
| 18499-19098 | 18798 | 0.00077 | 0.00161 | 3 |
| 18699-19298 | 18998 | 0.00276 | 0.00483 | 9 |
| 18899-19505 | 19198 | 0.00353 | 0.00644 | 12 |
| 19099-19705 | 19405 | 0.00301 | 0.00537 | 10 |
| 19299-19905 | 19605 | 0.00235 | 0.00322 | 6 |
| 19506-20105 | 19805 | 0.00158 | 0.00161 | 3 |
| 19706-20305 | 20005 | 0.00158 | 0.00161 | 3 |
| 19906-20505 | 20205 | 0.00098 | 0.00161 | 3 |
| 20106-20705 | 20405 | 0.00098 | 0.00161 | 3 |
| 20306-20905 | 20605 | 0.00098 | 0.00161 | 3 |
| 20506-21109 | 20805 | 0.00209 | 0.00269 | 5 |
| 20706-21309 | 21005 | 0.00338 | 0.00537 | 10 |
| 20906-21509 | 21209 | 0.00389 | 0.00644 | 12 |
| 21110-21709 | 21409 | 0.00316 | 0.00537 | 10 |
| 21310-21909 | 21609 | 0.00252 | 0.00322 | 6 |
| 21510-22109 | 21809 | 0.00261 | 0.00215 | 4 |
| 21710-22310 | 22009 | 0.0015 | 0.00107 | 2 |
| 21910-22510 | 22210 | 0.00085 | 0.00054 | 1 |
| 22110-22710 | 22410 | 0.00077 | 0.00161 | 3 |
| 22311-22910 | 22610 | 0.00103 | 0.00215 | 4 |
| 22511-23110 | 22810 | 0.00316 | 0.00483 | 9 |
| 22711-23310 | 23010 | 0.00265 | 0.00376 | 7 |
| 22911-23510 | 23210 | 0.00239 | 0.00322 | 6 |
| 23111-23710 | 23410 | 0.00098 | 0.00161 | 3 |
| 23311-23910 | 23610 | 0.00098 | 0.00161 | 3 |
| 23511-24110 | 23810 | 0.00124 | 0.00215 | 4 |
| 23711-24310 | 24010 | 0.00103 | 0.00215 | 4 |
| 23911-24510 | 24210 | 0.00103 | 0.00215 | 4 |
| 24111-24710 | 24410 | 0.00177 | 0.00322 | 6 |
| 24311-24910 | 24610 | 0.00152 | 0.00269 | 5 |
| 24511-25110 | 24810 | 0.00216 | 0.00269 | 5 |
| 24711-25310 | 25010 | 0.00141 | 0.00161 | 3 |
| 24911-25510 | 25210 | 0.00188 | 0.00215 | 4 |
| 25111-25710 | 25410 | 0.00201 | 0.00376 | 7 |
| 25311-25922 | 25610 | 0.00274 | 0.00483 | 9 |
| 25511-26129 | 25822 | 0.00423 | 0.00591 | 11 |
| 25711-26487 | 26022 | 0.00496 | 0.00698 | 13 |
| 25923-26691 | 26229 | 0.00474 | 0.00698 | 13 |
| 26130-26892 | 26591 | 0.00368 | 0.00644 | 12 |
| 26488-27100 | 26792 | 0.00218 | 0.00376 | 7 |
| 26693-27305 | 26994 | 0.00299 | 0.00376 | 7 |
| 26893-27511 | 27200 | 0.00274 | 0.00322 | 6 |
| 27101-27717 | 27405 | 0.00577 | 0.00591 | 11 |
| 27306-27933 | 27611 | 0.00556 | 0.00591 | 11 |
| 27512-28133 | 27821 | 0.00628 | 0.00698 | 13 |
| 27718-28339 | 28033 | 0.00423 | 0.00591 | 11 |
| 27934-28555 | 28233 | 0.00863 | 0.0102 | 19 |
| 28134-28783 | 28443 | 0.00868 | 0.0102 | 19 |
| 28340-28983 | 28683 | 0.00863 | 0.00967 | 18 |
| 28556-29183 | 28883 | 0.00483 | 0.00537 | 10 |
| 28784-29393 | 29083 | 0.00506 | 0.00591 | 11 |
| 28984-29618 | 29289 | 0.00502 | 0.00591 | 11 |
| 29184-29824 | 29507 | 0.00417 | 0.00537 | 10 |
| 29394-30034 | 29718 | 0.00363 | 0.0043 | 8 |
| 29619-30240 | 29934 | 0.00312 | 0.00322 | 6 |
| 29825-30458 | 30140 | 0.00363 | 0.0043 | 8 |
| 30035-30658 | 30352 | 0.00278 | 0.00376 | 7 |
| 30241-30861 | 30558 | 0.00316 | 0.00537 | 10 |
| 30459-31097 | 30758 | 0.00389 | 0.00644 | 12 |
| 30659-31312 | 30976 | 0.00521 | 0.00752 | 14 |
| 30862-31512 | 31210 | 0.00444 | 0.00591 | 11 |
| 31098-31717 | 31412 | 0.00308 | 0.0043 | 8 |
| 31313-31929 | 31612 | 0.00338 | 0.00537 | 10 |
| 31513-32129 | 31829 | 0.00551 | 0.00752 | 14 |
| 31718-32413 | 32029 | 0.00594 | 0.00752 | 14 |
| 31930-32670 | 32234 | 0.01038 | 0.01128 | 21 |
| 32130-32893 | 32532 | 0.01009 | 0.01128 | 21 |
| 32414-33096 | 32793 | 0.01051 | 0.01182 | 22 |
| 32671-33296 | 32993 | 0.00393 | 0.00483 | 9 |
| 32894-33496 | 33196 | 0.00274 | 0.00322 | 6 |
| 33097-33696 | 33396 | 0.00162 | 0.00215 | 4 |
| 33297-33896 | 33596 | 0.00162 | 0.00215 | 4 |
| 33497-34096 | 33796 | 0.00073 | 0.00107 | 2 |
| 33697-34296 | 33996 | 0 | 0 | 0 |
| 33897-34496 | 34196 | 0 | 0 | 0 |
| 34097-34696 | 34396 | 0 | 0 | 0 |
| 34297-34896 | 34596 | 0 | 0 | 0 |
| 34497-35096 | 34796 | 0.00026 | 0.00054 | 1 |
| 34697-35296 | 34996 | 0.00026 | 0.00054 | 1 |
| 34897-35496 | 35196 | 0.00026 | 0.00054 | 1 |
| 35097-35696 | 35396 | 0.00026 | 0.00054 | 1 |
| 35297-35901 | 35596 | 0.00115 | 0.00161 | 3 |
| 35497-36101 | 35801 | 0.00167 | 0.00269 | 5 |
| 35697-36308 | 36001 | 0.00346 | 0.00483 | 9 |
| 35902-36508 | 36208 | 0.00256 | 0.00376 | 7 |
| 36102-36721 | 36408 | 0.00436 | 0.00752 | 14 |
| 36309-36921 | 36616 | 0.00346 | 0.00644 | 12 |
| 36509-37122 | 36821 | 0.00526 | 0.00913 | 17 |
| 36722-37322 | 37021 | 0.00295 | 0.0043 | 8 |
| 36922-37522 | 37222 | 0.00179 | 0.00269 | 5 |
| 37123-37722 | 37422 | 0.00026 | 0.00054 | 1 |
| 37323-37922 | 37622 | 0.00103 | 0.00215 | 4 |
| 37523-38122 | 37822 | 0.00188 | 0.00269 | 5 |
| 37723-38322 | 38022 | 0.00162 | 0.00215 | 4 |
| 37923-38522 | 38222 | 0.00132 | 0.00107 | 2 |
| 38123-38722 | 38422 | 0.00047 | 0.00054 | 1 |
| 38323-38922 | 38622 | 0.00047 | 0.00054 | 1 |
| 38523-39122 | 38822 | 0 | 0 | 0 |
| 38723-39322 | 39022 | 0.00026 | 0.00054 | 1 |
| 38923-39522 | 39222 | 0.00051 | 0.00107 | 2 |
| 39123-39722 | 39422 | 0.00098 | 0.00161 | 3 |
| 39323-39922 | 39622 | 0.00073 | 0.00107 | 2 |
| 39523-40122 | 39822 | 0.00047 | 0.00054 | 1 |
| 39723-40322 | 40022 | 0.00026 | 0.00054 | 1 |
| 39923-40522 | 40222 | 0.00051 | 0.00107 | 2 |
| 40123-40722 | 40422 | 0.00051 | 0.00107 | 2 |
| 40323-40922 | 40622 | 0.00026 | 0.00054 | 1 |
| 40523-41122 | 40822 | 0 | 0 | 0 |
| 40723-41322 | 41022 | 0 | 0 | 0 |
| 40923-41522 | 41222 | 0 | 0 | 0 |
| 41123-41722 | 41422 | 0 | 0 | 0 |
| 41323-41922 | 41622 | 0.00132 | 0.00107 | 2 |
| 41523-42122 | 41822 | 0.00132 | 0.00107 | 2 |
| 41723-42327 | 42022 | 0.00261 | 0.00215 | 4 |
| 41923-42532 | 42222 | 0.00265 | 0.00269 | 5 |
| 42123-42735 | 42427 | 0.00389 | 0.00483 | 9 |
| 42328-42939 | 42632 | 0.00444 | 0.00591 | 11 |
| 42533-43140 | 42835 | 0.00397 | 0.00537 | 10 |
| 42736-43346 | 43039 | 0.00372 | 0.00483 | 9 |
| 42940-43546 | 43246 | 0.00188 | 0.00269 | 5 |
| 43141-43765 | 43446 | 0.00201 | 0.00269 | 5 |
| 43347-43965 | 43647 | 0.00154 | 0.00215 | 4 |
| 43547-44165 | 43865 | 0.00201 | 0.00269 | 5 |
| 43766-44370 | 44065 | 0.00256 | 0.00322 | 6 |
| 43966-44570 | 44270 | 0.00355 | 0.00483 | 9 |
| 44166-44770 | 44470 | 0.00308 | 0.0043 | 8 |
| 44371-44971 | 44670 | 0.00248 | 0.0043 | 8 |
| 44571-45171 | 44870 | 0.00256 | 0.00322 | 6 |
| 44771-45381 | 45071 | 0.00355 | 0.00483 | 9 |
| 44972-45583 | 45275 | 0.00393 | 0.00483 | 9 |
| 45172-45784 | 45483 | 0.00504 | 0.00591 | 11 |
| 45382-45990 | 45684 | 0.00585 | 0.00806 | 15 |
| 45584-46208 | 45890 | 0.00658 | 0.00913 | 17 |
| 45785-46408 | 46104 | 0.00504 | 0.00806 | 15 |
| 45991-46608 | 46308 | 0.00325 | 0.0043 | 8 |
| 46209-46809 | 46508 | 0.00226 | 0.00269 | 5 |
| 46409-47015 | 46708 | 0.00274 | 0.00322 | 6 |
| 46609-47254 | 46915 | 0.00368 | 0.00376 | 7 |
| 46810-47467 | 47146 | 0.00415 | 0.0043 | 8 |
| 47016-47684 | 47361 | 0.0038 | 0.00483 | 9 |
| 47255-47959 | 47584 | 0.00449 | 0.00644 | 12 |
| 47468-48161 | 47784 | 0.00393 | 0.00698 | 13 |
| 47685-48366 | 48060 | 0.00376 | 0.00537 | 10 |
| 47960-48566 | 48261 | 0.00239 | 0.00376 | 7 |
| 48162-48777 | 48466 | 0.00333 | 0.00483 | 9 |
| 48367-48982 | 48666 | 0.00577 | 0.00806 | 15 |
| 48567-49185 | 48877 | 0.00662 | 0.00859 | 16 |
| 48778-49391 | 49082 | 0.00686 | 0.00752 | 14 |
| 48983-49591 | 49290 | 0.00592 | 0.00537 | 10 |
| 49186-49797 | 49491 | 0.00553 | 0.00537 | 10 |
| 49392-49997 | 49697 | 0.00385 | 0.0043 | 8 |
| 49592-50197 | 49897 | 0.00124 | 0.00215 | 4 |
| 49798-50404 | 50097 | 0.00128 | 0.00269 | 5 |
| 49998-50604 | 50297 | 0.00077 | 0.00161 | 3 |
| 50198-50804 | 50504 | 0.00077 | 0.00161 | 3 |
| 50405-51004 | 50704 | 0 | 0 | 0 |
| 50605-51204 | 50904 | 0.0041 | 0.0043 | 8 |
| 50805-51404 | 51104 | 0.00543 | 0.00537 | 10 |
| 51005-51604 | 51304 | 0.00594 | 0.00644 | 12 |
| 51205-51804 | 51504 | 0.00235 | 0.00322 | 6 |
| 51405-52031 | 51704 | 0.00286 | 0.0043 | 8 |
| 51605-52251 | 51921 | 0.00419 | 0.00537 | 10 |
| 51805-52456 | 52141 | 0.00611 | 0.00644 | 12 |
| 52032-52684 | 52354 | 0.00694 | 0.00698 | 13 |
| 52252-52885 | 52574 | 0.00652 | 0.00698 | 13 |
| 52457-53086 | 52785 | 0.00583 | 0.00644 | 12 |
| 52685-53292 | 52985 | 0.00316 | 0.00376 | 7 |
| 52886-53492 | 53192 | 0.00248 | 0.00269 | 5 |
| 53087-53695 | 53392 | 0.00235 | 0.00322 | 6 |
| 53293-53895 | 53592 | 0.00363 | 0.00483 | 9 |
| 53493-54095 | 53795 | 0.00291 | 0.00376 | 7 |
| 53696-54295 | 53995 | 0.00154 | 0.00215 | 4 |
| 53896-54495 | 54195 | 0.00026 | 0.00054 | 1 |
| 54096-54695 | 54395 | 0.00077 | 0.00161 | 3 |
| 54296-54895 | 54595 | 0.00077 | 0.00161 | 3 |
| 54496-55095 | 54795 | 0.0015 | 0.00269 | 5 |
| 54696-55295 | 54995 | 0.00214 | 0.00269 | 5 |
| 54896-55495 | 55195 | 0.00188 | 0.00215 | 4 |
| 55096-55700 | 55395 | 0.00141 | 0.00161 | 3 |
| 55296-55906 | 55595 | 0.00209 | 0.00215 | 4 |
| 55496-56110 | 55806 | 0.00286 | 0.00376 | 7 |
| 55701-56323 | 56006 | 0.0035 | 0.0043 | 8 |
| 55907-56523 | 56210 | 0.00218 | 0.00376 | 7 |
| 56111-56723 | 56423 | 0.00167 | 0.00269 | 5 |
| 56324-56923 | 56623 | 0.00103 | 0.00215 | 4 |
| 56524-57123 | 56823 | 0.00124 | 0.00215 | 4 |
| 56724-57323 | 57023 | 0.00098 | 0.00161 | 3 |
| 56924-57523 | 57223 | 0.00098 | 0.00161 | 3 |
| 57124-57723 | 57423 | 0.00051 | 0.00107 | 2 |
| 57324-57927 | 57623 | 0.00077 | 0.00161 | 3 |
| 57524-58144 | 57823 | 0.00175 | 0.00322 | 6 |
| 57724-58373 | 58027 | 0.00175 | 0.00322 | 6 |
| 57928-58577 | 58250 | 0.0015 | 0.00269 | 5 |
| 58145-58777 | 58473 | 0.00098 | 0.00161 | 3 |
| 58374-58977 | 58677 | 0.00197 | 0.00322 | 6 |
| 58578-59180 | 58877 | 0.00274 | 0.00483 | 9 |
| 58778-59380 | 59080 | 0.00333 | 0.00483 | 9 |
| 58978-59580 | 59280 | 0.00346 | 0.0043 | 8 |
| 59181-59780 | 59480 | 0.00321 | 0.00376 | 7 |
| 59381-59980 | 59680 | 0.00299 | 0.00376 | 7 |
| 59581-60183 | 59880 | 0.00462 | 0.00483 | 9 |
| 59781-60412 | 60080 | 0.00795 | 0.00752 | 14 |
| 59981-60624 | 60304 | 0.00733 | 0.00698 | 13 |
| 60184-60828 | 60519 | 0.00434 | 0.0043 | 8 |
| 60413-61129 | 60726 | 0.00246 | 0.00215 | 4 |
| 60625-61329 | 61027 | 0.00269 | 0.00269 | 5 |
| 60829-61529 | 61229 | 0.00269 | 0.00269 | 5 |
| 61130-61729 | 61429 | 0.00184 | 0.00215 | 4 |
| 61330-61945 | 61629 | 0.00239 | 0.00376 | 7 |
| 61530-62224 | 61834 | 0.00363 | 0.00591 | 11 |
| 61730-62447 | 62124 | 0.00556 | 0.00859 | 16 |
| 61946-62647 | 62327 | 0.00637 | 0.00859 | 16 |
| 62225-62847 | 62547 | 0.00714 | 0.00859 | 16 |
| 62448-63047 | 62747 | 0.00457 | 0.00537 | 10 |
| 62648-63247 | 62947 | 0.00295 | 0.00322 | 6 |
| 62848-63447 | 63147 | 0.00094 | 0.00107 | 2 |
| 63048-63647 | 63347 | 0.00158 | 0.00161 | 3 |
| 63248-63847 | 63547 | 0.00111 | 0.00107 | 2 |
| 63448-64047 | 63747 | 0.00137 | 0.00161 | 3 |
| 63648-64247 | 63947 | 0.00026 | 0.00054 | 1 |
| 63848-64700 | 64147 | 0.00077 | 0.00161 | 3 |
| 64048-64906 | 64347 | 0.00282 | 0.00376 | 7 |
| 64248-65106 | 64806 | 0.00282 | 0.00376 | 7 |
| 64701-65306 | 65006 | 0.00295 | 0.00322 | 6 |
| 64907-65506 | 65206 | 0.00137 | 0.00161 | 3 |
| 65107-65706 | 65406 | 0.00235 | 0.00322 | 6 |
| 65307-65914 | 65606 | 0.00295 | 0.00483 | 9 |
| 65507-66115 | 65814 | 0.00551 | 0.00913 | 17 |
| 65707-66326 | 66015 | 0.0069 | 0.01074 | 20 |
| 65915-66537 | 66226 | 0.0081 | 0.01235 | 23 |
| 66116-66739 | 66426 | 0.00887 | 0.01289 | 24 |
| 66327-66942 | 66639 | 0.00799 | 0.01235 | 23 |
| 66538-67142 | 66839 | 0.00658 | 0.01074 | 20 |
| 66740-67355 | 67042 | 0.00303 | 0.00591 | 11 |
| 66943-67560 | 67255 | 0.00269 | 0.00483 | 9 |
| 67143-67793 | 67460 | 0.00218 | 0.00376 | 7 |
| 67356-68163 | 67660 | 0.01365 | 0.0188 | 35 |
| 67561-68395 | 67894 | 0.01622 | 0.02202 | 41 |
| 67794-68595 | 68295 | 0.0172 | 0.02202 | 41 |
| 68164-68798 | 68495 | 0.00714 | 0.00913 | 17 |
| 68396-69016 | 68698 | 0.00662 | 0.00859 | 16 |
| 68596-69216 | 68913 | 0.00564 | 0.00859 | 16 |
| 68799-69440 | 69116 | 0.01 | 0.0102 | 19 |
| 69017-69640 | 69340 | 0.00705 | 0.00644 | 12 |
| 69217-69850 | 69540 | 0.01323 | 0.01128 | 21 |
| 69441-70050 | 69740 | 0.00831 | 0.00806 | 15 |
| 69641-70250 | 69950 | 0.00878 | 0.00859 | 16 |
| 69851-70450 | 70150 | 0.00209 | 0.00269 | 5 |
| 70051-70678 | 70350 | 0.00286 | 0.00269 | 5 |
| 70251-70878 | 70570 | 0.00265 | 0.00269 | 5 |
| 70451-71083 | 70778 | 0.00265 | 0.00269 | 5 |
| 70679-71283 | 70978 | 0.00192 | 0.00322 | 6 |
| 70879-71491 | 71183 | 0.00453 | 0.00537 | 10 |
| 71084-71696 | 71391 | 0.00453 | 0.00537 | 10 |
| 71284-71902 | 71591 | 0.00479 | 0.00537 | 10 |
| 71492-72109 | 71801 | 0.00427 | 0.00483 | 9 |
| 71697-72310 | 72009 | 0.005 | 0.00591 | 11 |
| 71903-72510 | 72209 | 0.0041 | 0.0043 | 8 |
| 72110-72715 | 72410 | 0.00214 | 0.00215 | 4 |
| 72311-72928 | 72610 | 0.00278 | 0.00269 | 5 |
| 72511-73129 | 72827 | 0.00201 | 0.00215 | 4 |
| 72718-73345 | 73029 | 0.00248 | 0.00269 | 5 |
| 72929-73545 | 73245 | 0.00137 | 0.00161 | 3 |
| 73130-73745 | 73445 | 0.00308 | 0.0043 | 8 |
| 73346-73945 | 73645 | 0.00397 | 0.00537 | 10 |
| 73546-74145 | 73845 | 0.00397 | 0.00537 | 10 |
| 73746-74345 | 74045 | 0.00252 | 0.00322 | 6 |
| 73946-74545 | 74245 | 0.00137 | 0.00161 | 3 |
| 74146-74745 | 74445 | 0.00137 | 0.00161 | 3 |
| 74346-74945 | 74645 | 0.00162 | 0.00215 | 4 |
| 74546-75145 | 74845 | 0.00103 | 0.00215 | 4 |
| 74746-75345 | 75045 | 0.00077 | 0.00161 | 3 |
| 74946-75545 | 75245 | 0.00051 | 0.00107 | 2 |
| 75146-75745 | 75445 | 0.00073 | 0.00107 | 2 |
| 75346-75945 | 75645 | 0.00274 | 0.00322 | 6 |
| 75546-76145 | 75845 | 0.00346 | 0.0043 | 8 |
| 75746-76345 | 76045 | 0.00325 | 0.0043 | 8 |
| 75946-76545 | 76245 | 0.00201 | 0.00376 | 7 |
| 76146-76745 | 76445 | 0.00538 | 0.00752 | 14 |
| 76346-76952 | 76645 | 0.00731 | 0.00859 | 16 |
| 76546-77154 | 76850 | 0.00769 | 0.00859 | 16 |
| 76746-77354 | 77054 | 0.00406 | 0.0043 | 8 |
| 76953-77554 | 77254 | 0.00188 | 0.00269 | 5 |
| 77155-77754 | 77454 | 0.00073 | 0.00107 | 2 |
| 77355-77960 | 77654 | 0.00098 | 0.00161 | 3 |
| 77555-78160 | 77854 | 0.00124 | 0.00215 | 4 |
| 77755-78360 | 78060 | 0.00175 | 0.00322 | 6 |
| 77961-78560 | 78260 | 0.00214 | 0.00322 | 6 |
| 78161-78766 | 78460 | 0.00235 | 0.00322 | 6 |
| 78361-78966 | 78666 | 0.00209 | 0.00269 | 5 |
| 78561-79166 | 78866 | 0.00214 | 0.00322 | 6 |
| 78767-79367 | 79066 | 0.00321 | 0.0043 | 8 |
| 78967-79586 | 79266 | 0.00423 | 0.00644 | 12 |
| 79167-79786 | 79486 | 0.00376 | 0.00537 | 10 |
| 79368-79986 | 79686 | 0.00222 | 0.00376 | 7 |
| 79587-80186 | 79886 | 0.00205 | 0.00215 | 4 |
| 79787-80386 | 80086 | 0.00453 | 0.00483 | 9 |
| 79987-80587 | 80286 | 0.00453 | 0.00483 | 9 |
| 80187-80787 | 80486 | 0.00368 | 0.0043 | 8 |
| 80387-80987 | 80687 | 0.00026 | 0.00054 | 1 |
| 80588-81187 | 80887 | 0.00051 | 0.00107 | 2 |
| 80788-81388 | 81087 | 0.00154 | 0.00215 | 4 |
| 80988-81588 | 81288 | 0.00179 | 0.00269 | 5 |
| 81188-81792 | 81488 | 0.00338 | 0.0043 | 8 |
| 81389-81992 | 81692 | 0.00321 | 0.00376 | 7 |
| 81589-82199 | 81892 | 0.00658 | 0.00806 | 15 |
| 81793-82412 | 82092 | 0.00611 | 0.00752 | 14 |
| 81993-82612 | 82312 | 0.005 | 0.00644 | 12 |
| 82200-82820 | 82512 | 0.00248 | 0.00269 | 5 |
| 82413-83020 | 82712 | 0.00111 | 0.00107 | 2 |
| 82613-83220 | 82920 | 0.00162 | 0.00215 | 4 |
| 82821-83420 | 83120 | 0.00252 | 0.00322 | 6 |
| 83021-83677 | 83320 | 0.00996 | 0.01343 | 25 |
| 83221-83877 | 83552 | 0.01021 | 0.01396 | 26 |
| 83421-84088 | 83777 | 0.00821 | 0.01182 | 22 |
| 83678-84306 | 83987 | 0.00214 | 0.00322 | 6 |
| 83878-84515 | 84188 | 0.00406 | 0.00591 | 11 |
| 84089-84715 | 84406 | 0.00504 | 0.00752 | 14 |
| 84307-84915 | 84615 | 0.00615 | 0.00859 | 16 |
| 84516-85121 | 84815 | 0.00449 | 0.00644 | 12 |
| 84716-85327 | 85021 | 0.00402 | 0.00591 | 11 |
| 84916-85527 | 85221 | 0.00201 | 0.00376 | 7 |
| 85122-85727 | 85427 | 0.00098 | 0.00161 | 3 |
| 85328-85934 | 85627 | 0.00175 | 0.00322 | 6 |
| 85528-86135 | 85834 | 0.00231 | 0.00483 | 9 |
| 85728-86335 | 86034 | 0.00355 | 0.00698 | 13 |
| 85935-86535 | 86235 | 0.00226 | 0.0043 | 8 |
| 86136-86735 | 86435 | 0.00124 | 0.00215 | 4 |
| 86336-86935 | 86635 | 0 | 0 | 0 |
| 86536-87135 | 86835 | 0 | 0 | 0 |
| 86736-87335 | 87035 | 0 | 0 | 0 |
| 86936-87535 | 87235 | 0 | 0 | 0 |
| 87136-87735 | 87435 | 0 | 0 | 0 |
| 87336-87935 | 87635 | 0 | 0 | 0 |
| 87536-88135 | 87835 | 0.00026 | 0.00054 | 1 |
| 87736-88335 | 88035 | 0.00026 | 0.00054 | 1 |
| 87936-88535 | 88235 | 0.00026 | 0.00054 | 1 |
| 88136-88735 | 88435 | 0.00026 | 0.00054 | 1 |
| 88336-88935 | 88635 | 0.00115 | 0.00107 | 2 |
| 88536-89135 | 88835 | 0.00115 | 0.00107 | 2 |
| 88736-89335 | 89035 | 0.0009 | 0.00054 | 1 |
| 88936-89535 | 89235 | 0 | 0 | 0 |
| 89136-89735 | 89435 | 0.00026 | 0.00054 | 1 |
| 89336-89935 | 89635 | 0.00026 | 0.00054 | 1 |
| 89536-90135 | 89835 | 0.00026 | 0.00054 | 1 |
| 89736-90335 | 90035 | 0.00026 | 0.00054 | 1 |
| 89936-90535 | 90235 | 0.00026 | 0.00054 | 1 |
| 90136-90735 | 90435 | 0.00026 | 0.00054 | 1 |
| 90336-90935 | 90635 | 0 | 0 | 0 |
| 90536-91135 | 90835 | 0.00026 | 0.00054 | 1 |
| 90736-91335 | 91035 | 0.00051 | 0.00107 | 2 |
| 90936-91535 | 91235 | 0.00051 | 0.00107 | 2 |
| 91136-91735 | 91435 | 0.0012 | 0.00161 | 3 |
| 91336-91935 | 91635 | 0.00094 | 0.00107 | 2 |
| 91536-92135 | 91835 | 0.0012 | 0.00161 | 3 |
| 91736-92335 | 92035 | 0.00026 | 0.00054 | 1 |
| 91936-92535 | 92235 | 0.00026 | 0.00054 | 1 |
| 92136-92735 | 92435 | 0 | 0 | 0 |
| 92336-92935 | 92635 | 0 | 0 | 0 |
| 92536-93135 | 92835 | 0 | 0 | 0 |
| 92736-93335 | 93035 | 0.00026 | 0.00054 | 1 |
| 92936-93535 | 93235 | 0.00026 | 0.00054 | 1 |
| 93136-93735 | 93435 | 0.00051 | 0.00107 | 2 |
| 93336-93935 | 93635 | 0.00077 | 0.00161 | 3 |
| 93536-94141 | 93835 | 0.00103 | 0.00215 | 4 |
| 93736-94341 | 94041 | 0.00077 | 0.00161 | 3 |
| 93936-94541 | 94241 | 0.00051 | 0.00107 | 2 |
| 94142-94741 | 94441 | 0.00051 | 0.00107 | 2 |
| 94342-94941 | 94641 | 0.00051 | 0.00107 | 2 |
| 94542-95141 | 94841 | 0.00137 | 0.00161 | 3 |
| 94742-95341 | 95041 | 0.00111 | 0.00107 | 2 |
| 94942-95541 | 95241 | 0.00137 | 0.00161 | 3 |
| 95142-95741 | 95441 | 0.00026 | 0.00054 | 1 |
| 95342-95941 | 95641 | 0.00026 | 0.00054 | 1 |
| 95542-96141 | 95841 | 0 | 0 | 0 |
| 95742-96341 | 96041 | 0 | 0 | 0 |
| 95942-96541 | 96241 | 0 | 0 | 0 |
| 96142-96741 | 96441 | 0 | 0 | 0 |
| 96342-96941 | 96641 | 0 | 0 | 0 |
| 96542-97141 | 96841 | 0 | 0 | 0 |
| 96742-97341 | 97041 | 0 | 0 | 0 |
| 96942-97541 | 97241 | 0 | 0 | 0 |
| 97142-97741 | 97441 | 0 | 0 | 0 |
| 97342-97941 | 97641 | 0 | 0 | 0 |
| 97542-98141 | 97841 | 0 | 0 | 0 |
| 97742-98341 | 98041 | 0.00026 | 0.00054 | 1 |
| 97942-98541 | 98241 | 0.00051 | 0.00107 | 2 |
| 98142-98741 | 98441 | 0.00051 | 0.00107 | 2 |
| 98342-98941 | 98641 | 0.00073 | 0.00107 | 2 |
| 98542-99141 | 98841 | 0.00047 | 0.00054 | 1 |
| 98742-99341 | 99041 | 0.00047 | 0.00054 | 1 |
| 98942-99541 | 99241 | 0 | 0 | 0 |
| 99142-99741 | 99441 | 0 | 0 | 0 |
| 99342-99941 | 99641 | 0 | 0 | 0 |
| 99542-100141 | 99841 | 0 | 0 | 0 |
| 99742-100341 | 100041 | 0 | 0 | 0 |
| 99942-100541 | 100241 | 0 | 0 | 0 |
| 100142-100742 | 100441 | 0.00137 | 0.00161 | 3 |
| 100342-100942 | 100641 | 0.00137 | 0.00161 | 3 |
| 100542-101147 | 100842 | 0.00137 | 0.00161 | 3 |
| 100743-101353 | 101047 | 0 | 0 | 0 |
| 100943-101565 | 101253 | 0.00026 | 0.00054 | 1 |
| 101148-101765 | 101465 | 0.00051 | 0.00107 | 2 |
| 101354-101965 | 101665 | 0.00051 | 0.00107 | 2 |
| 101566-102165 | 101865 | 0.00026 | 0.00054 | 1 |
| 101766-102365 | 102065 | 0 | 0 | 0 |
| 101966-102565 | 102265 | 0.00047 | 0.00054 | 1 |
| 102166-102765 | 102465 | 0.00047 | 0.00054 | 1 |
| 102366-102965 | 102665 | 0.00047 | 0.00054 | 1 |
| 102566-103165 | 102865 | 0 | 0 | 0 |
| 102766-103365 | 103065 | 0 | 0 | 0 |
| 102966-103565 | 103265 | 0 | 0 | 0 |
| 103166-103765 | 103465 | 0 | 0 | 0 |
| 103366-103965 | 103665 | 0 | 0 | 0 |
| 103566-104165 | 103865 | 0 | 0 | 0 |
| 103766-104365 | 104065 | 0 | 0 | 0 |
| 103966-104565 | 104265 | 0 | 0 | 0 |
| 104166-104765 | 104465 | 0 | 0 | 0 |
| 104366-104965 | 104665 | 0 | 0 | 0 |
| 104566-105165 | 104865 | 0.00026 | 0.00054 | 1 |
| 104766-105365 | 105065 | 0.00026 | 0.00054 | 1 |
| 104966-105565 | 105265 | 0.00051 | 0.00107 | 2 |
| 105166-105765 | 105465 | 0.00026 | 0.00054 | 1 |
| 105366-105965 | 105665 | 0.00051 | 0.00107 | 2 |
| 105566-106165 | 105865 | 0.00026 | 0.00054 | 1 |
| 105766-106365 | 106065 | 0.00026 | 0.00054 | 1 |
| 105966-106565 | 106265 | 0 | 0 | 0 |
| 106166-106765 | 106465 | 0 | 0 | 0 |
| 106366-106965 | 106665 | 0 | 0 | 0 |
| 106566-107165 | 106865 | 0 | 0 | 0 |
| 106766-107365 | 107065 | 0 | 0 | 0 |
| 106966-107565 | 107265 | 0.00068 | 0.00054 | 1 |
| 107166-107765 | 107465 | 0.00068 | 0.00054 | 1 |
| 107366-107965 | 107665 | 0.00068 | 0.00054 | 1 |
| 107566-108165 | 107865 | 0 | 0 | 0 |
| 107766-108365 | 108065 | 0 | 0 | 0 |
| 107966-108565 | 108265 | 0 | 0 | 0 |
| 108166-108765 | 108465 | 0 | 0 | 0 |
| 108366-108965 | 108665 | 0 | 0 | 0 |
| 108566-109165 | 108865 | 0 | 0 | 0 |
| 108766-109365 | 109065 | 0 | 0 | 0 |
| 108966-109565 | 109265 | 0 | 0 | 0 |
| 109166-109765 | 109465 | 0 | 0 | 0 |
| 109366-109965 | 109665 | 0.00026 | 0.00054 | 1 |
| 109566-110168 | 109865 | 0.00212 | 0.00269 | 5 |
| 109766-110368 | 110065 | 0.00237 | 0.00322 | 6 |
| 109966-110568 | 110268 | 0.00212 | 0.00269 | 5 |
| 110169-110768 | 110468 | 0.00385 | 0.00269 | 5 |
| 110369-110968 | 110668 | 0.00359 | 0.00215 | 4 |
| 110569-111168 | 110868 | 0.00359 | 0.00215 | 4 |
| 110769-111368 | 111068 | 0 | 0 | 0 |
| 110969-111568 | 111268 | 0 | 0 | 0 |
| 111169-111768 | 111468 | 0.00026 | 0.00054 | 1 |
| 111369-111968 | 111668 | 0.00026 | 0.00054 | 1 |
| 111569-112168 | 111868 | 0.00218 | 0.00269 | 5 |
| 111769-112368 | 112068 | 0.00192 | 0.00215 | 4 |
| 111969-112568 | 112268 | 0.00244 | 0.00322 | 6 |
| 112169-112768 | 112468 | 0.00226 | 0.0043 | 8 |
| 112369-112968 | 112668 | 0.00348 | 0.00591 | 11 |
| 112569-113168 | 112868 | 0.00447 | 0.00752 | 14 |
| 112769-113368 | 113068 | 0.00395 | 0.00644 | 12 |
| 112969-113568 | 113268 | 0.0044 | 0.00752 | 14 |
| 113169-113768 | 113468 | 0.00342 | 0.00591 | 11 |
| 113369-113968 | 113668 | 0.00218 | 0.00376 | 7 |
| 113569-114168 | 113868 | 0.00103 | 0.00215 | 4 |
| 113769-114368 | 114068 | 0.00201 | 0.00376 | 7 |
| 113969-114568 | 114268 | 0.00278 | 0.0043 | 8 |
| 114169-114768 | 114468 | 0.00363 | 0.00483 | 9 |
| 114369-114977 | 114668 | 0.00457 | 0.00591 | 11 |
| 114569-115213 | 114868 | 0.00692 | 0.00859 | 16 |
| 114769-115700 | 115092 | 0.01128 | 0.01343 | 25 |
| 114978-115905 | 115593 | 0.00962 | 0.01128 | 21 |
| 115214-116105 | 115805 | 0.00868 | 0.01128 | 21 |
| 115701-116305 | 116005 | 0.00444 | 0.00752 | 14 |
| 115906-116505 | 116205 | 0.00543 | 0.00752 | 14 |
| 116106-116705 | 116405 | 0.00842 | 0.01074 | 20 |
| 116306-116905 | 116605 | 0.00692 | 0.00806 | 15 |
| 116506-117112 | 116805 | 0.0059 | 0.00752 | 14 |
| 116706-117328 | 117012 | 0.00261 | 0.00376 | 7 |
| 116906-117528 | 117228 | 0.00357 | 0.00483 | 9 |
| 117113-117728 | 117428 | 0.004 | 0.00483 | 9 |
| 117329-117928 | 117628 | 0.00237 | 0.00269 | 5 |
| 117529-118128 | 117828 | 0.00231 | 0.00269 | 5 |
| 117729-118328 | 118028 | 0.00188 | 0.00269 | 5 |
| 117929-118528 | 118228 | 0.00188 | 0.00269 | 5 |
| 118129-118728 | 118428 | 0.00274 | 0.00483 | 9 |
| 118329-118930 | 118628 | 0.00286 | 0.0043 | 8 |
| 118529-119135 | 118830 | 0.00363 | 0.00591 | 11 |
| 118729-119346 | 119030 | 0.0053 | 0.00644 | 12 |
| 118931-119546 | 119241 | 0.00496 | 0.00698 | 13 |
| 119136-119746 | 119446 | 0.00568 | 0.00644 | 12 |
| 119347-119946 | 119646 | 0.00363 | 0.0043 | 8 |
| 119547-120146 | 119846 | 0.00385 | 0.0043 | 8 |
| 119747-120352 | 120046 | 0.00359 | 0.00537 | 10 |
| 119947-120616 | 120246 | 0.00521 | 0.00698 | 13 |
| 120147-120818 | 120459 | 0.0056 | 0.00698 | 13 |
| 120353-121018 | 120718 | 0.00474 | 0.00483 | 9 |
| 120617-121218 | 120918 | 0.00239 | 0.00215 | 4 |
| 120819-121418 | 121118 | 0.00218 | 0.00215 | 4 |
| 121019-121618 | 121318 | 0.00154 | 0.00161 | 3 |
| 121219-121818 | 121518 | 0.00201 | 0.00215 | 4 |
| 121419-122023 | 121718 | 0.00162 | 0.00215 | 4 |
| 121619-122235 | 121918 | 0.00397 | 0.00537 | 10 |
| 121819-122435 | 122123 | 0.00286 | 0.0043 | 8 |
| 122024-122635 | 122335 | 0.00545 | 0.00591 | 11 |
| 122236-122835 | 122535 | 0.00382 | 0.00376 | 7 |
| 122436-123035 | 122735 | 0.00382 | 0.00376 | 7 |
| 122636-123235 | 122935 | 0.0012 | 0.00161 | 3 |
| 122836-123441 | 123135 | 0.00073 | 0.00107 | 2 |
| 123036-123641 | 123335 | 0.00137 | 0.00161 | 3 |
| 123236-123841 | 123541 | 0.00115 | 0.00161 | 3 |
| 123442-124041 | 123741 | 0.0009 | 0.00107 | 2 |
| 123642-124241 | 123941 | 0.00051 | 0.00107 | 2 |
| 123842-124441 | 124141 | 0.00051 | 0.00107 | 2 |
| 124042-124641 | 124341 | 0.00051 | 0.00107 | 2 |
| 124242-124869 | 124541 | 0.00509 | 0.00591 | 11 |
| 124442-125071 | 124769 | 0.0062 | 0.00698 | 13 |
| 124642-125288 | 124969 | 0.01218 | 0.0145 | 27 |
| 124870-125488 | 125188 | 0.00906 | 0.01182 | 22 |
| 125072-125688 | 125388 | 0.00915 | 0.01235 | 23 |
| 125289-125888 | 125588 | 0.00462 | 0.00698 | 13 |
| 125489-126088 | 125788 | 0.00707 | 0.00967 | 18 |
| 125689-126288 | 125988 | 0.00831 | 0.0102 | 19 |
| 125889-126488 | 126188 | 0.01045 | 0.01343 | 25 |
| 126089-126688 | 126388 | 0.01077 | 0.01289 | 24 |
| 126289-126888 | 126588 | 0.00934 | 0.01128 | 21 |
| 126489-127088 | 126788 | 0.00925 | 0.01074 | 20 |
| 126689-127294 | 126988 | 0.006 | 0.00806 | 15 |
| 126889-127494 | 127188 | 0.00615 | 0.00913 | 17 |
| 127089-127709 | 127394 | 0.00806 | 0.0102 | 19 |
| 127295-127909 | 127609 | 0.01152 | 0.01289 | 24 |
| 127495-128109 | 127809 | 0.01412 | 0.0145 | 27 |
| 127710-128318 | 128009 | 0.0109 | 0.01128 | 21 |
| 127910-128518 | 128209 | 0.00923 | 0.0102 | 19 |
| 128110-128734 | 128418 | 0.00991 | 0.0145 | 27 |
| 128319-128961 | 128618 | 0.01051 | 0.01719 | 32 |
| 128519-129161 | 128861 | 0.00825 | 0.01504 | 28 |
| 128735-129382 | 129061 | 0.00483 | 0.00967 | 18 |
| 128962-129582 | 129282 | 0.00231 | 0.00483 | 9 |
| 129162-129782 | 129482 | 0.00231 | 0.00376 | 7 |
| 129383-129982 | 129682 | 0.00218 | 0.00269 | 5 |
| 129583-130182 | 129882 | 0.00192 | 0.00215 | 4 |
| 129783-130382 | 130082 | 0.00141 | 0.00215 | 4 |
| 129983-130582 | 130282 | 0.00026 | 0.00054 | 1 |
| 130183-130782 | 130482 | 0.00026 | 0.00054 | 1 |
| 130383-130982 | 130682 | 0 | 0 | 0 |
| 130583-131182 | 130882 | 0 | 0 | 0 |
| 130783-131382 | 131082 | 0.00359 | 0.00215 | 4 |
| 130983-131582 | 131282 | 0.00359 | 0.00215 | 4 |
| 131183-131785 | 131482 | 0.00571 | 0.00483 | 9 |
| 131383-131985 | 131682 | 0.00237 | 0.00322 | 6 |
| 131583-132185 | 131885 | 0.00237 | 0.00322 | 6 |
| 131786-132385 | 132085 | 0.00026 | 0.00054 | 1 |
| 131986-132585 | 132285 | 0 | 0 | 0 |
| 132186-132785 | 132485 | 0 | 0 | 0 |
| 132386-132985 | 132685 | 0 | 0 | 0 |
| 132586-133185 | 132885 | 0 | 0 | 0 |
| 132786-133385 | 133085 | 0 | 0 | 0 |
| 132986-133585 | 133285 | 0 | 0 | 0 |
| 133186-133785 | 133485 | 0 | 0 | 0 |
| 133386-133985 | 133685 | 0 | 0 | 0 |
| 133586-134185 | 133885 | 0 | 0 | 0 |
| 133786-134385 | 134085 | 0.00068 | 0.00054 | 1 |
| 133986-134585 | 134285 | 0.00068 | 0.00054 | 1 |
| 134186-134785 | 134485 | 0.00068 | 0.00054 | 1 |
| 134386-134985 | 134685 | 0 | 0 | 0 |
| 134586-135185 | 134885 | 0 | 0 | 0 |
| 134786-135385 | 135085 | 0 | 0 | 0 |
| 134986-135585 | 135285 | 0 | 0 | 0 |
| 135186-135785 | 135485 | 0 | 0 | 0 |
| 135386-135985 | 135685 | 0.00026 | 0.00054 | 1 |
| 135586-136185 | 135885 | 0.00026 | 0.00054 | 1 |
| 135786-136385 | 136085 | 0.00051 | 0.00107 | 2 |
| 135986-136585 | 136285 | 0.00026 | 0.00054 | 1 |
| 136186-136785 | 136485 | 0.00051 | 0.00107 | 2 |
| 136386-136985 | 136685 | 0.00026 | 0.00054 | 1 |
| 136586-137185 | 136885 | 0.00026 | 0.00054 | 1 |
| 136786-137385 | 137085 | 0 | 0 | 0 |
| 136986-137585 | 137285 | 0 | 0 | 0 |
| 137186-137785 | 137485 | 0 | 0 | 0 |
| 137386-137985 | 137685 | 0 | 0 | 0 |
| 137586-138185 | 137885 | 0 | 0 | 0 |
| 137786-138385 | 138085 | 0 | 0 | 0 |
| 137986-138585 | 138285 | 0 | 0 | 0 |
| 138186-138785 | 138485 | 0 | 0 | 0 |
| 138386-138985 | 138685 | 0 | 0 | 0 |
| 138586-139185 | 138885 | 0 | 0 | 0 |
| 138786-139385 | 139085 | 0 | 0 | 0 |
| 138986-139585 | 139285 | 0.00047 | 0.00054 | 1 |
| 139186-139785 | 139485 | 0.00047 | 0.00054 | 1 |
| 139386-139985 | 139685 | 0.00047 | 0.00054 | 1 |
| 139586-140185 | 139885 | 0.00026 | 0.00054 | 1 |
| 139786-140385 | 140085 | 0.00026 | 0.00054 | 1 |
| 139986-140597 | 140285 | 0.00051 | 0.00107 | 2 |
| 140186-140803 | 140497 | 0.00026 | 0.00054 | 1 |
| 140386-141008 | 140703 | 0.00026 | 0.00054 | 1 |
| 140598-141209 | 140908 | 0.00051 | 0.00107 | 2 |
| 140804-141409 | 141108 | 0.00137 | 0.00161 | 3 |
| 141009-141609 | 141309 | 0.00137 | 0.00161 | 3 |
| 141210-141809 | 141509 | 0.00085 | 0.00054 | 1 |
| 141410-142009 | 141709 | 0 | 0 | 0 |
| 141610-142209 | 141909 | 0 | 0 | 0 |
| 141810-142409 | 142109 | 0 | 0 | 0 |
| 142010-142609 | 142309 | 0 | 0 | 0 |
| 142210-142809 | 142509 | 0 | 0 | 0 |
| 142410-143009 | 142709 | 0.00047 | 0.00054 | 1 |
| 142610-143209 | 142909 | 0.00047 | 0.00054 | 1 |
| 142810-143409 | 143109 | 0.00047 | 0.00054 | 1 |
| 143010-143609 | 143309 | 0.00051 | 0.00107 | 2 |
| 143210-143809 | 143509 | 0.00051 | 0.00107 | 2 |
| 143410-144009 | 143709 | 0.00051 | 0.00107 | 2 |
| 143610-144209 | 143909 | 0 | 0 | 0 |
| 143810-144409 | 144109 | 0 | 0 | 0 |
| 144010-144609 | 144309 | 0 | 0 | 0 |
| 144210-144809 | 144509 | 0 | 0 | 0 |
| 144410-145009 | 144709 | 0 | 0 | 0 |
| 144610-145209 | 144909 | 0 | 0 | 0 |
| 144810-145409 | 145109 | 0 | 0 | 0 |
| 145010-145609 | 145309 | 0 | 0 | 0 |
| 145210-145809 | 145509 | 0 | 0 | 0 |
| 145410-146009 | 145709 | 0 | 0 | 0 |
| 145610-146209 | 145909 | 0 | 0 | 0 |
| 145810-146409 | 146109 | 0.00026 | 0.00054 | 1 |
| 146010-146609 | 146309 | 0.00026 | 0.00054 | 1 |
| 146210-146809 | 146509 | 0.00051 | 0.00107 | 2 |
| 146410-147009 | 146709 | 0.00111 | 0.00107 | 2 |
| 146610-147209 | 146909 | 0.00111 | 0.00107 | 2 |
| 146810-147409 | 147109 | 0.00111 | 0.00107 | 2 |
| 147010-147609 | 147309 | 0.00051 | 0.00107 | 2 |
| 147210-147809 | 147509 | 0.00077 | 0.00161 | 3 |
| 147410-148015 | 147709 | 0.00077 | 0.00161 | 3 |
| 147610-148215 | 147915 | 0.00077 | 0.00161 | 3 |
| 147810-148415 | 148115 | 0.00077 | 0.00161 | 3 |
| 148016-148615 | 148315 | 0.00077 | 0.00161 | 3 |
| 148216-148815 | 148515 | 0.00051 | 0.00107 | 2 |
| 148416-149015 | 148715 | 0.00026 | 0.00054 | 1 |
| 148616-149215 | 148915 | 0 | 0 | 0 |
| 148816-149415 | 149115 | 0 | 0 | 0 |
| 149016-149615 | 149315 | 0 | 0 | 0 |
| 149216-149815 | 149515 | 0 | 0 | 0 |
| 149416-150015 | 149715 | 0.00026 | 0.00054 | 1 |
| 149616-150215 | 149915 | 0.00073 | 0.00107 | 2 |
| 149816-150415 | 150115 | 0.0012 | 0.00161 | 3 |
| 150016-150615 | 150315 | 0.00094 | 0.00107 | 2 |
| 150216-150815 | 150515 | 0.00073 | 0.00107 | 2 |
| 150416-151015 | 150715 | 0.00051 | 0.00107 | 2 |
| 150616-151215 | 150915 | 0.00051 | 0.00107 | 2 |
| 150816-151415 | 151115 | 0.00026 | 0.00054 | 1 |
| 151016-151615 | 151315 | 0 | 0 | 0 |
| 151216-151815 | 151515 | 0.00026 | 0.00054 | 1 |
| 151416-152015 | 151715 | 0.00026 | 0.00054 | 1 |
| 151616-152215 | 151915 | 0.00051 | 0.00107 | 2 |
| 151816-152415 | 152115 | 0.00026 | 0.00054 | 1 |
| 152016-152615 | 152315 | 0.00026 | 0.00054 | 1 |
| 152216-152815 | 152515 | 0 | 0 | 0 |
| 152416-153015 | 152715 | 0 | 0 | 0 |
| 152616-153215 | 152915 | 0.0009 | 0.00054 | 1 |
| 152816-153415 | 153115 | 0.00115 | 0.00107 | 2 |
| 153016-153615 | 153315 | 0.00115 | 0.00107 | 2 |
| 153216-153815 | 153515 | 0.00026 | 0.00054 | 1 |
| 153416-154015 | 153715 | 0.00026 | 0.00054 | 1 |
| 153616-154215 | 153915 | 0.00026 | 0.00054 | 1 |
| 153816-154415 | 154115 | 0.00026 | 0.00054 | 1 |
| 154016-154615 | 154315 | 0 | 0 | 0 |
| 154216-154815 | 154515 | 0 | 0 | 0 |
| 154416-155015 | 154715 | 0 | 0 | 0 |
| 154616-155215 | 154915 | 0 | 0 | 0 |
| 154816-155415 | 155115 | 0 | 0 | 0 |
| 155016-155796 | 155274 | 0 | 0 | 0 |
