# Supplementary material for: Complete chloroplast genomes of eight Delphinium taxa (Ranunculaceae) endemic to Xinjiang, China: insights into genome structure, comparative analysis, and phylogenetic relationships
Source: BMC Plant Biol. 2024 Jun 26;24:600. doi: 10.1186/s12870-024-05279-y (PMC11201361; doi:10.1186/s12870-024-05279-y)
Supplement: Supplementary file 6 — Supplementary Material 6 [file 12870_2024_5279_MOESM6_ESM.docx]

**TABLE S6** The codon usage information of 77 protein-coding genes in the 14 *Delphinium* taxa.

| **Samples** | **Genes** | **ENC** | **CBI** | **SChi2** | **G+C2** | **G+C3s** | **G+Cc** | **G+C** |
| --- | --- | --- | --- | --- | --- | --- | --- | --- |
| *Aconitum brachypodum*_MT584424 | *acc*D | 50.955 | 0.403 | 0.329 | 0.356 | 0.285 | 0.363 | 0.363 |
| *Aconitum delavayi*_OM289058 | *acc*D | 51.19 | 0.402 | 0.325 | 0.356 | 0.287 | 0.364 | 0.364 |
| *Delphinium aemulans*_LHM12800 | *acc*D | 50.327 | 0.418 | 0.358 | 0.359 | 0.279 | 0.357 | 0.357 |
| *Delphinium anthriscifolium*_MK253461 | *acc*D | 51.918 | 0.388 | 0.321 | 0.357 | 0.287 | 0.365 | 0.365 |
| *Delphinium brunonianum*_NC_051554 | *acc*D | 50.249 | 0.422 | 0.357 | 0.359 | 0.275 | 0.355 | 0.355 |
| *Delphinium candelabrum* var. *monanthum*_MW246165 | *acc*D | 50.037 | 0.423 | 0.358 | 0.361 | 0.275 | 0.357 | 0.357 |
| *Delphinium ceratophorum*_MK253460 | *acc*D | 50.005 | 0.419 | 0.357 | 0.359 | 0.274 | 0.357 | 0.357 |
| *Delphinium elatum* var. *sericeum*_LHM1265 | *acc*D | 50.305 | 0.418 | 0.359 | 0.359 | 0.277 | 0.357 | 0.357 |
| *Delphinium iliense*_LHM1285 | *acc*D | 50.206 | 0.418 | 0.357 | 0.357 | 0.275 | 0.355 | 0.355 |
| *Delphinium maackianum*_NC_047293 | *acc*D | 50.129 | 0.428 | 0.366 | 0.357 | 0.273 | 0.355 | 0.355 |
| *Delphinium mollifolium*_LHM1295 | *acc*D | 50.266 | 0.416 | 0.355 | 0.359 | 0.275 | 0.356 | 0.356 |
| *Delphinium naviculare* var. *lasiocarpum*_LHM1293 | *acc*D | 50.203 | 0.416 | 0.357 | 0.357 | 0.277 | 0.356 | 0.356 |
| *Delphinium sauricum*_LHM1266 | *acc*D | 50.19 | 0.416 | 0.355 | 0.359 | 0.275 | 0.356 | 0.356 |
| *Delphinium shawurense*_LHM1271 | *acc*D | 50.305 | 0.418 | 0.359 | 0.359 | 0.277 | 0.357 | 0.357 |
| *Delphinium winklerianum*_LHM1299 | *acc*D | 50.252 | 0.416 | 0.356 | 0.359 | 0.275 | 0.355 | 0.355 |
| *Delphinium yunnanense*_MW246156 | *acc*D | 50.154 | 0.423 | 0.359 | 0.361 | 0.275 | 0.357 | 0.357 |
| *Aconitum brachypodum*_MT584424 | *atp*A | 49.332 | 0.393 | 0.306 | 0.402 | 0.289 | 0.423 | 0.422 |
| *Aconitum delavayi*_OM289058 | *atp*A | 49.332 | 0.393 | 0.306 | 0.402 | 0.289 | 0.423 | 0.422 |
| *Delphinium aemulans*_LHM1280 | *atp*A | 49.344 | 0.404 | 0.309 | 0.402 | 0.291 | 0.422 | 0.421 |
| *Delphinium anthriscifolium*_MK253461 | *atp*A | 48.672 | 0.418 | 0.325 | 0.402 | 0.281 | 0.419 | 0.419 |
| *Delphinium brunonianum*_NC_051554 | *atp*A | 49.344 | 0.404 | 0.309 | 0.402 | 0.291 | 0.422 | 0.421 |
| *Delphinium candelabrum* var. *monanthum*_MW246165 | *atp*A | 49.45 | 0.4 | 0.302 | 0.402 | 0.293 | 0.423 | 0.422 |
| *Delphinium ceratophorum*_MK253460 | *atp*A | 49.49 | 0.399 | 0.305 | 0.402 | 0.293 | 0.423 | 0.422 |
| *Delphinium elatum* var. *sericeum*_LHM1265 | *atp*A | 49.344 | 0.404 | 0.309 | 0.402 | 0.291 | 0.422 | 0.421 |
| *Delphinium iliense*_LHM1285 | *atp*A | 49.515 | 0.399 | 0.302 | 0.402 | 0.295 | 0.423 | 0.423 |
| *Delphinium maackianum*_NC_047293 | *atp*A | 49.004 | 0.407 | 0.312 | 0.402 | 0.291 | 0.422 | 0.421 |
| *Delphinium mollifolium*_LHM1295 | *atp*A | 49.685 | 0.398 | 0.3 | 0.402 | 0.295 | 0.423 | 0.423 |
| *Delphinium naviculare* var. *lasiocarpum*_LHM1293 | *atp*A | 49.515 | 0.399 | 0.302 | 0.402 | 0.295 | 0.423 | 0.423 |
| *Delphinium sauricum*_LHM1266 | *atp*A | 49.856 | 0.394 | 0.298 | 0.402 | 0.299 | 0.425 | 0.424 |
| *Delphinium shawurense*_LHM1271 | *atp*A | 49.344 | 0.404 | 0.309 | 0.402 | 0.291 | 0.422 | 0.421 |
| *Delphinium winklerianum*_LHM1299 | *atp*A | 49.515 | 0.399 | 0.302 | 0.402 | 0.295 | 0.423 | 0.423 |
| Delphinium yunnanense_MW246156 | *atp*A | 49.45 | 0.4 | 0.302 | 0.402 | 0.293 | 0.423 | 0.422 |
| *Aconitum brachypodum*_MT584424 | *atp*B | 47.479 | 0.415 | 0.332 | 0.414 | 0.273 | 0.426 | 0.426 |
| *Aconitum delavayi*_OM289058 | *atp*B | 47.126 | 0.418 | 0.34 | 0.414 | 0.273 | 0.426 | 0.426 |
| *Delphinium aemulans*_LHM1280 | *atp*B | 48.471 | 0.408 | 0.316 | 0.42 | 0.282 | 0.43 | 0.43 |
| *Delphinium anthriscifolium*_MK253461 | *atp*B | 47.789 | 0.411 | 0.323 | 0.414 | 0.282 | 0.428 | 0.428 |
| *Delphinium brunonianum*_NC_051554 | *atp*B | 48.526 | 0.408 | 0.314 | 0.418 | 0.282 | 0.429 | 0.429 |
| *Delphinium candelabrum* var. *monanthum*_MW246165 | *atp*B | 48.583 | 0.407 | 0.313 | 0.418 | 0.284 | 0.43 | 0.43 |
| *Delphinium ceratophorum*_MK253460 | *atp*B | 48.441 | 0.408 | 0.315 | 0.418 | 0.282 | 0.428 | 0.428 |
| *Delphinium elatum* var. *sericeum*_LHM1265 | *atp*B | 48.756 | 0.404 | 0.31 | 0.418 | 0.284 | 0.43 | 0.43 |
| *Delphinium iliense*_LHM1285 | *atp*B | 48.575 | 0.407 | 0.312 | 0.418 | 0.28 | 0.428 | 0.428 |
| *Delphinium maackianum*_NC_047293 | *atp*B | 48.664 | 0.404 | 0.31 | 0.418 | 0.284 | 0.43 | 0.43 |
| *Delphinium mollifolium*_LHM1295 | *atp*B | 48.575 | 0.407 | 0.312 | 0.418 | 0.28 | 0.428 | 0.428 |
| *Delphinium naviculare* var. *lasiocarpum*_LHM1293 | *atp*B | 48.575 | 0.407 | 0.312 | 0.418 | 0.28 | 0.428 | 0.428 |
| *Delphinium sauricum*_LHM1266 | *atp*B | 48.36 | 0.41 | 0.319 | 0.418 | 0.277 | 0.428 | 0.428 |
| *Delphinium shawurense*_LHM1271 | *atp*B | 48.526 | 0.408 | 0.314 | 0.418 | 0.282 | 0.429 | 0.429 |
| *Delphinium winklerianum*_LHM1299 | *atp*B | 48.36 | 0.41 | 0.319 | 0.418 | 0.277 | 0.428 | 0.428 |
| Delphinium yunnanense_MW246156 | *atp*B | 48.526 | 0.408 | 0.314 | 0.418 | 0.282 | 0.429 | 0.429 |
| *Aconitum brachypodum*_MT584424 | *atp*E | 49.651 | 0.583 | 0.624 | 0.406 | 0.24 | 0.388 | 0.388 |
| *Aconitum delavayi*_OM289058 | *atp*E | 49.651 | 0.583 | 0.624 | 0.406 | 0.24 | 0.388 | 0.388 |
| *Delphinium aemulans*_LHM1280 | *atp*E | 46.636 | 0.587 | 0.638 | 0.421 | 0.24 | 0.391 | 0.388 |
| *Delphinium anthriscifolium*_MK253461 | *atp*E | 46.636 | 0.587 | 0.638 | 0.421 | 0.24 | 0.391 | 0.388 |
| *Delphinium brunonianum*_NC_051554 | *atp*E | 46.636 | 0.587 | 0.638 | 0.421 | 0.24 | 0.391 | 0.388 |
| *Delphinium candelabrum* var. *monanthum*_MW246165 | *atp*E | 46.636 | 0.587 | 0.638 | 0.421 | 0.24 | 0.391 | 0.388 |
| *Delphinium ceratophorum*_MK253460 | *atp*E | 46.636 | 0.587 | 0.638 | 0.421 | 0.24 | 0.391 | 0.388 |
| *Delphinium elatum* var. *sericeum*_LHM1265 | *atp*E | 46.636 | 0.587 | 0.638 | 0.421 | 0.24 | 0.391 | 0.388 |
| *Delphinium iliense*_LHM1285 | *atp*E | 46.636 | 0.587 | 0.638 | 0.421 | 0.24 | 0.391 | 0.388 |
| *Delphinium maackianum*_NC_047293 | *atp*E | 46.636 | 0.587 | 0.638 | 0.421 | 0.24 | 0.391 | 0.388 |
| *Delphinium mollifolium*_LHM1295 | *atp*E | 46.636 | 0.587 | 0.638 | 0.421 | 0.24 | 0.391 | 0.388 |
| *Delphinium naviculare* var. *lasiocarpum*_LHM1293 | *atp*E | 46.636 | 0.587 | 0.638 | 0.421 | 0.24 | 0.391 | 0.388 |
| *Delphinium sauricum*_LHM1266 | *atp*E | 46.636 | 0.587 | 0.638 | 0.421 | 0.24 | 0.391 | 0.388 |
| *Delphinium shawurense*_LHM1271 | *atp*E | 46.636 | 0.587 | 0.638 | 0.421 | 0.24 | 0.391 | 0.388 |
| *Delphinium winklerianum*_LHM1299 | *atp*E | 46.636 | 0.587 | 0.638 | 0.421 | 0.24 | 0.391 | 0.388 |
| *Delphinium yunnanense*_MW246156 | *atp*E | 46.636 | 0.587 | 0.638 | 0.421 | 0.24 | 0.391 | 0.388 |
| *Aconitum brachypodum*_MT584424 | *atp*F | 42.458 | 0.491 | 0.51 | 0.332 | 0.317 | 0.375 | 0.375 |
| *Aconitum delavayi*_OM289058 | *atp*F | 42.458 | 0.491 | 0.51 | 0.332 | 0.317 | 0.375 | 0.375 |
| *Delphinium aemulans*_LHM1280 | *atp*F | 42.592 | 0.515 | 0.522 | 0.333 | 0.309 | 0.373 | 0.373 |
| *Delphinium anthriscifolium*_MK253461 | *atp*F | 43.334 | 0.513 | 0.496 | 0.337 | 0.289 | 0.366 | 0.366 |
| *Delphinium brunonianum*_NC_051554 | *atp*F | 42.364 | 0.528 | 0.533 | 0.333 | 0.304 | 0.371 | 0.371 |
| *Delphinium candelabrum* var. *monanthum*_MW246165 | *atp*F | 42.592 | 0.515 | 0.522 | 0.333 | 0.309 | 0.373 | 0.373 |
| *Delphinium ceratophorum*_MK253460 | *atp*F | 43.095 | 0.509 | 0.507 | 0.333 | 0.315 | 0.375 | 0.374 |
| *Delphinium elatum* var. *sericeum*_LHM1265 | *atp*F | 42.592 | 0.515 | 0.522 | 0.333 | 0.309 | 0.373 | 0.373 |
| *Delphinium iliense*_LHM1285 | *atp*F | 42.488 | 0.522 | 0.526 | 0.333 | 0.309 | 0.373 | 0.373 |
| *Delphinium maackianum*_NC_047293 | *atp*F | 42.311 | 0.527 | 0.538 | 0.333 | 0.304 | 0.369 | 0.369 |
| *Delphinium mollifolium*_LHM1295 | *atp*F | 43.702 | 0.514 | 0.501 | 0.333 | 0.315 | 0.375 | 0.374 |
| *Delphinium naviculare* var. *lasiocarpum*_LHM1293 | *atp*F | 42.488 | 0.522 | 0.526 | 0.333 | 0.309 | 0.373 | 0.373 |
| *Delphinium sauricum*_LHM1266 | *atp*F | 43.702 | 0.514 | 0.501 | 0.333 | 0.315 | 0.375 | 0.374 |
| *Delphinium shawurense*_LHM1271 | *atp*F | 42.592 | 0.515 | 0.522 | 0.333 | 0.309 | 0.373 | 0.373 |
| *Delphinium winklerianum*_LHM1299 | *atp*F | 43.702 | 0.514 | 0.501 | 0.333 | 0.315 | 0.375 | 0.374 |
| *Delphinium yunnanense*_MW246156 | *atp*F | 42.592 | 0.515 | 0.522 | 0.333 | 0.309 | 0.373 | 0.373 |
| *Aconitum brachypodum*_MT584424 | *atp*H | 48.505 | 0.585 | 0.726 | 0.494 | 0.203 | 0.453 | 0.447 |
| *Aconitum delavayi*_OM289058 | *atp*H | 48.505 | 0.585 | 0.726 | 0.494 | 0.203 | 0.453 | 0.447 |
| *Delphinium aemulans*_LHM1280 | *atp*H | 48.676 | 0.588 | 0.745 | 0.494 | 0.203 | 0.449 | 0.443 |
| *Delphinium anthriscifolium*_MK253461 | *atp*H | 49.165 | 0.581 | 0.714 | 0.494 | 0.203 | 0.449 | 0.443 |
| *Delphinium brunonianum*_NC_051554 | *atp*H | 48.676 | 0.588 | 0.745 | 0.494 | 0.203 | 0.449 | 0.443 |
| *Delphinium candelabrum* var. *monanthum*_MW246165 | *atp*H | 48.676 | 0.588 | 0.745 | 0.494 | 0.203 | 0.449 | 0.443 |
| *Delphinium ceratophorum*_MK253460 | *atp*H | 48.676 | 0.588 | 0.745 | 0.494 | 0.203 | 0.449 | 0.443 |
| *Delphinium elatum* var. *sericeum*_LHM1265 | *atp*H | 48.676 | 0.588 | 0.745 | 0.494 | 0.203 | 0.449 | 0.443 |
| *Delphinium iliense*_LHM1285 | *atp*H | 48.676 | 0.588 | 0.745 | 0.494 | 0.19 | 0.444 | 0.439 |
| *Delphinium maackianum*_NC_047293 | *atp*H | 48.676 | 0.588 | 0.745 | 0.494 | 0.203 | 0.449 | 0.443 |
| *Delphinium mollifolium*_LHM1295 | *atp*H | 48.676 | 0.588 | 0.745 | 0.494 | 0.203 | 0.449 | 0.443 |
| *Delphinium naviculare* var. *lasiocarpum*_LHM1293 | *atp*H | 48.676 | 0.588 | 0.745 | 0.494 | 0.19 | 0.444 | 0.439 |
| *Delphinium sauricum*_LHM1266 | *atp*H | 48.676 | 0.588 | 0.745 | 0.494 | 0.203 | 0.449 | 0.443 |
| *Delphinium shawurense*_LHM1271 | *atp*H | 48.676 | 0.588 | 0.745 | 0.494 | 0.203 | 0.449 | 0.443 |
| *Delphinium winklerianum*_LHM1299 | *atp*H | 48.676 | 0.588 | 0.745 | 0.494 | 0.203 | 0.449 | 0.443 |
| *Delphinium yunnanense*_MW246156 | *atp*H | 48.676 | 0.588 | 0.745 | 0.494 | 0.203 | 0.449 | 0.443 |
| *Aconitum brachypodum*_MT584424 | *atp*I | 45.852 | 0.503 | 0.544 | 0.377 | 0.231 | 0.374 | 0.374 |
| *Aconitum delavayi*_OM289058 | *atp*I | 45.852 | 0.503 | 0.544 | 0.377 | 0.231 | 0.374 | 0.374 |
| *Delphinium aemulans*_LHM1280 | *atp*I | 47.256 | 0.472 | 0.493 | 0.381 | 0.252 | 0.381 | 0.38 |
| *Delphinium anthriscifolium*_MK253461 | *atp*I | 47.759 | 0.459 | 0.5 | 0.377 | 0.244 | 0.377 | 0.376 |
| *Delphinium brunonianum*_NC_051554 | *atp*I | 47.283 | 0.478 | 0.497 | 0.381 | 0.252 | 0.382 | 0.382 |
| *Delphinium candelabrum* var. *monanthum*_MW246165 | *atp*I | 47.255 | 0.477 | 0.495 | 0.381 | 0.252 | 0.382 | 0.382 |
| *Delphinium ceratophorum*_MK253460 | *atp*I | 47.283 | 0.478 | 0.497 | 0.381 | 0.252 | 0.382 | 0.382 |
| *Delphinium elatum* var. *sericeum*_LHM1265 | *atp*I | 47.256 | 0.472 | 0.493 | 0.381 | 0.252 | 0.381 | 0.38 |
| *Delphinium iliense*_LHM1285 | *atp*I | 46.732 | 0.479 | 0.507 | 0.381 | 0.248 | 0.379 | 0.379 |
| *Delphinium maackianum*_NC_047293 | *atp*I | 47.283 | 0.478 | 0.497 | 0.381 | 0.252 | 0.382 | 0.382 |
| *Delphinium mollifolium*_LHM1295 | *atp*I | 46.732 | 0.479 | 0.507 | 0.381 | 0.248 | 0.379 | 0.379 |
| *Delphinium naviculare* var. *lasiocarpum*_LHM1293 | *atp*I | 46.732 | 0.479 | 0.507 | 0.381 | 0.248 | 0.379 | 0.379 |
| *Delphinium sauricum*_LHM1266 | *atp*I | 46.732 | 0.479 | 0.507 | 0.381 | 0.248 | 0.379 | 0.379 |
| *Delphinium shawurense*_LHM1271 | *atp*I | 47.256 | 0.472 | 0.493 | 0.381 | 0.256 | 0.382 | 0.382 |
| *Delphinium winklerianum*_LHM1299 | *atp*I | 46.732 | 0.479 | 0.507 | 0.381 | 0.248 | 0.379 | 0.379 |
| *Delphinium yunnanense*_MW246156 | *atp*I | 47.075 | 0.479 | 0.501 | 0.381 | 0.248 | 0.381 | 0.38 |
| *Aconitum brachypodum*_MT584424 | *ccs*A | 44.814 | 0.538 | 0.558 | 0.36 | 0.197 | 0.326 | 0.326 |
| *Aconitum delavayi*_OM289058 | *ccs*A | 44.653 | 0.536 | 0.562 | 0.36 | 0.197 | 0.325 | 0.325 |
| *Delphinium aemulans*_LHM1280 | *ccs*A | 46.254 | 0.525 | 0.528 | 0.354 | 0.203 | 0.321 | 0.321 |
| *Delphinium anthriscifolium*_MK253461 | *ccs*A | 45.962 | 0.527 | 0.524 | 0.365 | 0.19 | 0.321 | 0.321 |
| *Delphinium brunonianum*_NC_051554 | *ccs*A | 46.581 | 0.53 | 0.532 | 0.357 | 0.2 | 0.321 | 0.321 |
| *Delphinium candelabrum* var. *monanthum*_MW246165 | *ccs*A | 45.895 | 0.53 | 0.531 | 0.354 | 0.203 | 0.32 | 0.32 |
| *Delphinium ceratophorum*_MK253460 | *ccs*A | 47.067 | 0.517 | 0.515 | 0.354 | 0.203 | 0.321 | 0.321 |
| *Delphinium elatum* var. *sericeum*_LHM1265 | *ccs*A | 46.254 | 0.525 | 0.528 | 0.354 | 0.203 | 0.321 | 0.321 |
| *Delphinium iliense*_LHM1285 | *ccs*A | 46.225 | 0.539 | 0.55 | 0.351 | 0.193 | 0.318 | 0.318 |
| *Delphinium maackianum*_NC_047293 | *ccs*A | 46.188 | 0.528 | 0.53 | 0.354 | 0.2 | 0.32 | 0.32 |
| *Delphinium mollifolium*_LHM1295 | *ccs*A | 46.449 | 0.536 | 0.541 | 0.351 | 0.197 | 0.319 | 0.319 |
| *Delphinium naviculare* var. *lasiocarpum*_LHM1293 | *ccs*A | 46.225 | 0.539 | 0.55 | 0.351 | 0.193 | 0.318 | 0.318 |
| *Delphinium sauricum*_LHM1266 | *ccs*A | 46.028 | 0.54 | 0.554 | 0.351 | 0.193 | 0.317 | 0.317 |
| *Delphinium shawurense*_LHM1271 | *ccs*A | 46.254 | 0.525 | 0.528 | 0.354 | 0.203 | 0.321 | 0.321 |
| *Delphinium winklerianum*_LHM1299 | *ccs*A | 46.225 | 0.539 | 0.55 | 0.351 | 0.193 | 0.318 | 0.318 |
| *Delphinium yunnanense*_MW246156 | *ccs*A | 46.739 | 0.53 | 0.529 | 0.354 | 0.196 | 0.318 | 0.318 |
| *Aconitum brachypodum*_MT584424 | *cem*A | 47.17 | 0.373 | 0.368 | 0.279 | 0.267 | 0.326 | 0.325 |
| *Aconitum delavayi*_OM289058 | *cem*A | 49.249 | 0.367 | 0.355 | 0.279 | 0.267 | 0.325 | 0.323 |
| *Delphinium aemulans*_LHM1280 | *cem*A | 52.163 | 0.366 | 0.341 | 0.277 | 0.269 | 0.325 | 0.323 |
| *Delphinium anthriscifolium*_MK253461 | *cem*A | 49.561 | 0.385 | 0.38 | 0.275 | 0.266 | 0.322 | 0.32 |
| *Delphinium brunonianum*_NC_051554 | *cem*A | 51.393 | 0.368 | 0.343 | 0.277 | 0.269 | 0.325 | 0.323 |
| *Delphinium candelabrum* var. *monanthum*_MW246165 | *cem*A | 51.091 | 0.369 | 0.344 | 0.281 | 0.269 | 0.328 | 0.326 |
| *Delphinium ceratophorum*_MK253460 | *cem*A | 51.393 | 0.368 | 0.343 | 0.277 | 0.269 | 0.325 | 0.323 |
| *Delphinium elatum* var. *sericeum*_LHM1265 | *cem*A | 52.246 | 0.363 | 0.335 | 0.277 | 0.274 | 0.326 | 0.325 |
| *Delphinium iliense*_LHM1285 | *cem*A | 49.423 | 0.366 | 0.343 | 0.273 | 0.274 | 0.325 | 0.323 |
| *Delphinium maackianum*_NC_047293 | *cem*A | 51.393 | 0.368 | 0.343 | 0.277 | 0.269 | 0.325 | 0.323 |
| *Delphinium mollifolium*_LHM1295 | *cem*A | 49.423 | 0.366 | 0.343 | 0.273 | 0.274 | 0.325 | 0.323 |
| *Delphinium naviculare* var. *lasiocarpum*_LHM1293 | *cem*A | 49.357 | 0.365 | 0.349 | 0.273 | 0.274 | 0.323 | 0.322 |
| *Delphinium sauricum*_LHM1266 | *cem*A | 49.423 | 0.366 | 0.343 | 0.273 | 0.274 | 0.325 | 0.323 |
| *Delphinium shawurense*_LHM1271 | *cem*A | 54.937 | 0.365 | 0.322 | 0.281 | 0.274 | 0.328 | 0.326 |
| *Delphinium winklerianum*_LHM1299 | *cem*A | 49.229 | 0.362 | 0.342 | 0.277 | 0.274 | 0.326 | 0.325 |
| *Delphinium yunnanense*_MW246156 | *cem*A | 51.108 | 0.368 | 0.348 | 0.281 | 0.269 | 0.326 | 0.325 |
| *Aconitum brachypodum*_MT584424 | *clp*P | 56.338 | 0.433 | 0.36 | 0.373 | 0.296 | 0.43 | 0.427 |
| *Aconitum delavayi*_OM289058 | *clp*P | 54.98 | 0.446 | 0.384 | 0.378 | 0.296 | 0.431 | 0.429 |
| *Delphinium aemulans*_LHM1280 | *clp*P | 55.907 | 0.42 | 0.355 | 0.379 | 0.297 | 0.437 | 0.436 |
| *Delphinium anthriscifolium*_MK253461 | *clp*P | 59.372 | 0.39 | 0.322 | 0.368 | 0.303 | 0.435 | 0.433 |
| *Delphinium brunonianum*_NC_051554 | *clp*P | 55.915 | 0.411 | 0.344 | 0.378 | 0.293 | 0.434 | 0.432 |
| *Delphinium candelabrum* var. *monanthum*_MW246165 | *clp*P | 55.915 | 0.411 | 0.344 | 0.378 | 0.293 | 0.434 | 0.432 |
| *Delphinium ceratophorum*_MK253460 | *clp*P | 56.102 | 0.411 | 0.342 | 0.378 | 0.293 | 0.435 | 0.433 |
| *Delphinium elatum* var. *sericeum*_LHM1265 | *clp*P | 55.907 | 0.42 | 0.355 | 0.379 | 0.297 | 0.437 | 0.436 |
| *Delphinium iliense*_LHM1285 | *clp*P | 55.716 | 0.42 | 0.358 | 0.379 | 0.297 | 0.435 | 0.435 |
| *Delphinium maackianum*_NC_047293 | *clp*P | 55.927 | 0.402 | 0.344 | 0.37 | 0.29 | 0.43 | 0.428 |
| *Delphinium mollifolium*_LHM1295 | *clp*P | 55.716 | 0.42 | 0.358 | 0.379 | 0.297 | 0.435 | 0.435 |
| *Delphinium naviculare* var. *lasiocarpum*_LHM1293 | *clp*P | 55.716 | 0.42 | 0.358 | 0.379 | 0.297 | 0.435 | 0.435 |
| *Delphinium sauricum*_LHM1266 | *clp*P | 55.716 | 0.42 | 0.358 | 0.379 | 0.297 | 0.435 | 0.435 |
| *Delphinium shawurense*_LHM1271 | *clp*P | 55.907 | 0.42 | 0.355 | 0.379 | 0.297 | 0.437 | 0.436 |
| *Delphinium winklerianum*_LHM1299 | *clp*P | 55.716 | 0.42 | 0.358 | 0.379 | 0.297 | 0.435 | 0.435 |
| *Delphinium yunnanense*_MW246156 | *clp*P | 55.915 | 0.411 | 0.344 | 0.378 | 0.293 | 0.434 | 0.432 |
| *Aconitum brachypodum*_MT584424 | *inf*A | 50.873 | 0.573 | 0.623 | 0.39 | 0.23 | 0.381 | 0.376 |
| *Aconitum delavayi*_OM289058 | *inf*A | 50.873 | 0.573 | 0.623 | 0.39 | 0.23 | 0.381 | 0.376 |
| *Delphinium aemulans*_LHM1280 | *inf*A | 49.703 | 0.587 | 0.65 | 0.39 | 0.243 | 0.385 | 0.38 |
| *Delphinium anthriscifolium*_MK253461 | *inf*A | 50.962 | 0.6 | 0.64 | 0.39 | 0.203 | 0.368 | 0.363 |
| *Delphinium brunonianum*_NC_051554 | *inf*A | 52.493 | 0.584 | 0.623 | 0.39 | 0.257 | 0.39 | 0.385 |
| *Delphinium candelabrum* var. *monanthum*_MW246165 | *inf*A | 49.703 | 0.587 | 0.65 | 0.39 | 0.243 | 0.385 | 0.38 |
| *Delphinium ceratophorum*_MK253460 | *inf*A | 49.703 | 0.587 | 0.65 | 0.39 | 0.243 | 0.385 | 0.38 |
| *Delphinium elatum* var. *sericeum*_LHM1265 | *inf*A | 49.703 | 0.587 | 0.65 | 0.39 | 0.243 | 0.385 | 0.38 |
| *Delphinium iliense*_LHM1285 | *inf*A | 49.703 | 0.587 | 0.65 | 0.39 | 0.243 | 0.385 | 0.38 |
| *Delphinium maackianum*_NC_047293 | *inf*A | 49.703 | 0.587 | 0.65 | 0.39 | 0.243 | 0.385 | 0.38 |
| *Delphinium mollifolium*_LHM1295 | *inf*A | 49.703 | 0.587 | 0.65 | 0.39 | 0.243 | 0.385 | 0.38 |
| *Delphinium naviculare* var. *lasiocarpum*_LHM1293 | *inf*A | 49.703 | 0.587 | 0.65 | 0.39 | 0.243 | 0.385 | 0.38 |
| *Delphinium sauricum*_LHM1266 | *inf*A | 49.703 | 0.587 | 0.65 | 0.39 | 0.243 | 0.385 | 0.38 |
| *Delphinium shawurense*_LHM1271 | *inf*A | 49.703 | 0.587 | 0.65 | 0.39 | 0.243 | 0.385 | 0.38 |
| *Delphinium winklerianum*_LHM1299 | *inf*A | 49.703 | 0.587 | 0.65 | 0.39 | 0.243 | 0.385 | 0.38 |
| *Delphinium yunnanense*_MW246156 | *inf*A | 49.703 | 0.587 | 0.65 | 0.39 | 0.243 | 0.385 | 0.38 |
| *Aconitum brachypodum*_MT584424 | *mat*K | 46.454 | 0.495 | 0.401 | 0.292 | 0.224 | 0.31 | 0.311 |
| *Aconitum delavayi*_OM289058 | *mat*K | 46.565 | 0.49 | 0.397 | 0.294 | 0.226 | 0.312 | 0.313 |
| *Delphinium aemulans*_LHM1280 | *mat*K | 47.958 | 0.483 | 0.376 | 0.298 | 0.23 | 0.317 | 0.317 |
| *Delphinium anthriscifolium*_MK253461 | *mat*K | 47.32 | 0.46 | 0.355 | 0.308 | 0.244 | 0.32 | 0.321 |
| *Delphinium brunonianum*_NC_051554 | *mat*K | 47.696 | 0.486 | 0.383 | 0.294 | 0.235 | 0.316 | 0.317 |
| *Delphinium candelabrum* var. *monanthum*_MW246165 | *mat*K | 48.144 | 0.495 | 0.387 | 0.294 | 0.233 | 0.314 | 0.315 |
| *Delphinium ceratophorum*_MK253460 | *mat*K | 48 | 0.476 | 0.37 | 0.296 | 0.239 | 0.318 | 0.319 |
| *Delphinium elatum* var. *sericeum*_LHM1265 | *mat*K | 47.8 | 0.492 | 0.384 | 0.296 | 0.229 | 0.317 | 0.318 |
| *Delphinium iliense*_LHM1285 | *mat*K | 47.797 | 0.485 | 0.381 | 0.296 | 0.235 | 0.318 | 0.319 |
| *Delphinium maackianum*_NC_047293 | *mat*K | 47.777 | 0.491 | 0.385 | 0.298 | 0.235 | 0.318 | 0.319 |
| *Delphinium mollifolium*_LHM1295 | *mat*K | 47.802 | 0.485 | 0.381 | 0.296 | 0.235 | 0.318 | 0.318 |
| *Delphinium naviculare* var. *lasiocarpum*_LHM1293 | *mat*K | 47.797 | 0.485 | 0.381 | 0.296 | 0.235 | 0.318 | 0.319 |
| *Delphinium sauricum*_LHM1266 | *mat*K | 47.802 | 0.485 | 0.381 | 0.296 | 0.235 | 0.318 | 0.318 |
| *Delphinium shawurense*_LHM1271 | *mat*K | 47.688 | 0.491 | 0.385 | 0.296 | 0.229 | 0.316 | 0.317 |
| *Delphinium winklerianum*_LHM1299 | *mat*K | 47.735 | 0.49 | 0.384 | 0.294 | 0.233 | 0.316 | 0.317 |
| *Delphinium yunnanense*_MW246156 | *mat*K | 48.11 | 0.493 | 0.385 | 0.296 | 0.231 | 0.316 | 0.317 |
| *Aconitum brachypodum*_MT584424 | *ndh*A | 41.517 | 0.469 | 0.466 | 0.402 | 0.21 | 0.36 | 0.359 |
| *Aconitum delavayi*_OM289058 | *ndh*A | 42.217 | 0.456 | 0.455 | 0.402 | 0.216 | 0.361 | 0.36 |
| *Delphinium aemulans*_LHM1280 | *ndh*A | 41.953 | 0.462 | 0.47 | 0.398 | 0.209 | 0.359 | 0.358 |
| *Delphinium anthriscifolium*_MK253461 | *ndh*A | 43.298 | 0.464 | 0.441 | 0.402 | 0.213 | 0.359 | 0.358 |
| *Delphinium brunonianum*_NC_051554 | *ndh*A | 41.733 | 0.469 | 0.478 | 0.398 | 0.206 | 0.358 | 0.357 |
| *Delphinium candelabrum* var. *monanthum*_MW246165 | *ndh*A | 41.855 | 0.465 | 0.469 | 0.398 | 0.211 | 0.36 | 0.359 |
| *Delphinium ceratophorum*_MK253460 | *ndh*A | 41.752 | 0.469 | 0.477 | 0.398 | 0.206 | 0.359 | 0.358 |
| *Delphinium elatum* var. *sericeum*_LHM1265 | *ndh*A | 41.953 | 0.462 | 0.47 | 0.398 | 0.209 | 0.359 | 0.358 |
| *Delphinium iliense*_LHM1285 | *ndh*A | 41.733 | 0.469 | 0.478 | 0.398 | 0.206 | 0.358 | 0.357 |
| *Delphinium maackianum*_NC_047293 | *ndh*A | 41.676 | 0.47 | 0.484 | 0.398 | 0.203 | 0.357 | 0.356 |
| *Delphinium mollifolium*_LHM1295 | *ndh*A | 41.733 | 0.469 | 0.478 | 0.398 | 0.206 | 0.358 | 0.357 |
| *Delphinium naviculare* var. *lasiocarpum*_LHM1293 | *ndh*A | 41.733 | 0.469 | 0.478 | 0.398 | 0.206 | 0.358 | 0.357 |
| *Delphinium sauricum*_LHM1266 | *ndh*A | 41.733 | 0.469 | 0.478 | 0.398 | 0.206 | 0.358 | 0.357 |
| *Delphinium shawurense*_LHM1271 | *ndh*A | 41.953 | 0.462 | 0.47 | 0.398 | 0.209 | 0.359 | 0.358 |
| *Delphinium winklerianum*_LHM1299 | *ndh*A | 41.733 | 0.469 | 0.478 | 0.398 | 0.206 | 0.358 | 0.357 |
| *Delphinium yunnanense*_MW246156 | *ndh*A | 41.769 | 0.468 | 0.474 | 0.398 | 0.212 | 0.36 | 0.359 |
| *Aconitum brachypodum*_MT584424 | *ndh*B | 47.191 | 0.359 | 0.315 | 0.388 | 0.271 | 0.373 | 0.372 |
| *Aconitum delavayi*_OM289058 | *ndh*B | 47.152 | 0.36 | 0.316 | 0.388 | 0.271 | 0.373 | 0.373 |
| *Delphinium aemulans*_LHM1280 | *ndh*B | 47.477 | 0.347 | 0.306 | 0.388 | 0.277 | 0.375 | 0.375 |
| *Delphinium anthriscifolium*_MK253461 | *ndh*B | 47.177 | 0.358 | 0.314 | 0.384 | 0.273 | 0.373 | 0.372 |
| *Delphinium brunonianum*_NC_051554 | *ndh*B | 47.477 | 0.347 | 0.306 | 0.388 | 0.277 | 0.375 | 0.375 |
| *Delphinium candelabrum* var. *monanthum*_MW246165 | *ndh*B | 47.477 | 0.347 | 0.306 | 0.388 | 0.277 | 0.375 | 0.375 |
| *Delphinium ceratophorum*_MK253460 | *ndh*B | 47.477 | 0.347 | 0.306 | 0.388 | 0.277 | 0.375 | 0.375 |
| *Delphinium elatum* var. *sericeum*_LHM1265 | *ndh*B | 47.477 | 0.347 | 0.306 | 0.388 | 0.277 | 0.375 | 0.375 |
| *Delphinium iliense*_LHM1285 | *ndh*B | 47.477 | 0.347 | 0.306 | 0.388 | 0.277 | 0.375 | 0.375 |
| *Delphinium maackianum*_NC_047293 | *ndh*B | 47.575 | 0.345 | 0.302 | 0.388 | 0.279 | 0.376 | 0.376 |
| *Delphinium mollifolium*_LHM1295 | *ndh*B | 47.414 | 0.351 | 0.309 | 0.388 | 0.275 | 0.375 | 0.374 |
| *Delphinium naviculare* var. *lasiocarpum*_LHM1293 | *ndh*B | 47.477 | 0.347 | 0.306 | 0.388 | 0.277 | 0.375 | 0.375 |
| *Delphinium sauricum*_LHM1266 | *ndh*B | 47.477 | 0.347 | 0.306 | 0.388 | 0.277 | 0.375 | 0.375 |
| *Delphinium shawurense*_LHM1271 | *ndh*B | 47.477 | 0.347 | 0.306 | 0.388 | 0.277 | 0.375 | 0.375 |
| *Delphinium winklerianum*_LHM1299 | *ndh*B | 47.477 | 0.347 | 0.306 | 0.388 | 0.277 | 0.375 | 0.375 |
| *Delphinium yunnanense*_MW246156 | *ndh*B | 47.477 | 0.347 | 0.306 | 0.388 | 0.277 | 0.375 | 0.375 |
| *Aconitum brachypodum*_MT584424 | *ndh*C | 45.356 | 0.533 | 0.605 | 0.325 | 0.216 | 0.364 | 0.364 |
| *Aconitum delavayi*_OM289058 | *ndh*C | 45.356 | 0.533 | 0.605 | 0.325 | 0.216 | 0.364 | 0.364 |
| *Delphinium aemulans*_LHM1280 | *ndh*C | 43.557 | 0.546 | 0.631 | 0.325 | 0.207 | 0.361 | 0.361 |
| *Delphinium anthriscifolium*_MK253461 | *ndh*C | 44.025 | 0.546 | 0.642 | 0.325 | 0.216 | 0.367 | 0.366 |
| *Delphinium brunonianum*_NC_051554 | *ndh*C | 43.557 | 0.546 | 0.631 | 0.325 | 0.216 | 0.364 | 0.364 |
| *Delphinium candelabrum* var. *monanthum*_MW246165 | *ndh*C | 43.373 | 0.567 | 0.646 | 0.325 | 0.207 | 0.361 | 0.361 |
| *Delphinium ceratophorum*_MK253460 | *ndh*C | 43.547 | 0.537 | 0.628 | 0.325 | 0.225 | 0.367 | 0.366 |
| *Delphinium elatum* var. *sericeum*_LHM1265 | *ndh*C | 43.557 | 0.546 | 0.631 | 0.325 | 0.207 | 0.361 | 0.361 |
| *Delphinium iliense*_LHM1285 | *ndh*C | 43.434 | 0.551 | 0.636 | 0.325 | 0.207 | 0.364 | 0.364 |
| *Delphinium maackianum*_NC_047293 | *ndh*C | 43.557 | 0.546 | 0.631 | 0.325 | 0.216 | 0.364 | 0.364 |
| *Delphinium mollifolium*_LHM1295 | *ndh*C | 43.557 | 0.546 | 0.631 | 0.325 | 0.207 | 0.361 | 0.361 |
| *Delphinium naviculare* var. *lasiocarpum*_LHM1293 | *ndh*C | 43.434 | 0.551 | 0.636 | 0.325 | 0.207 | 0.364 | 0.364 |
| *Delphinium sauricum*_LHM1266 | *ndh*C | 43.557 | 0.546 | 0.631 | 0.325 | 0.207 | 0.361 | 0.361 |
| *Delphinium shawurense*_LHM1271 | *ndh*C | 43.557 | 0.546 | 0.631 | 0.325 | 0.207 | 0.361 | 0.361 |
| *Delphinium winklerianum*_LHM1299 | *ndh*C | 43.557 | 0.546 | 0.631 | 0.325 | 0.207 | 0.361 | 0.361 |
| *Delphinium yunnanense*_MW246156 | *ndh*C | 43.557 | 0.546 | 0.631 | 0.325 | 0.216 | 0.364 | 0.364 |
| *Aconitum brachypodum*_MT584424 | *ndh*D | 47.082 | 0.4 | 0.34 | 0.365 | 0.244 | 0.356 | 0.356 |
| *Aconitum delavayi*_OM289058 | *ndh*D | 48.284 | 0.402 | 0.329 | 0.367 | 0.246 | 0.357 | 0.357 |
| *Delphinium aemulans*_LHM1280 | *ndh*D | 48.799 | 0.374 | 0.313 | 0.371 | 0.26 | 0.363 | 0.363 |
| *Delphinium anthriscifolium*_MK253461 | *ndh*D | 50.437 | 0.36 | 0.282 | 0.367 | 0.273 | 0.369 | 0.369 |
| *Delphinium brunonianum*_NC_051554 | *ndh*D | 48.987 | 0.374 | 0.309 | 0.371 | 0.262 | 0.363 | 0.363 |
| *Delphinium candelabrum* var. *monanthum*_MW246165 | *ndh*D | 48.381 | 0.375 | 0.317 | 0.371 | 0.26 | 0.363 | 0.363 |
| *Delphinium ceratophorum*_MK253460 | *ndh*D | 48.808 | 0.373 | 0.312 | 0.371 | 0.26 | 0.363 | 0.363 |
| *Delphinium elatum* var. *sericeum*_LHM1265 | *ndh*D | 49.095 | 0.373 | 0.311 | 0.371 | 0.26 | 0.365 | 0.365 |
| *Delphinium iliense*_LHM1285 | *ndh*D | 48.8 | 0.375 | 0.313 | 0.371 | 0.26 | 0.363 | 0.363 |
| *Delphinium maackianum*_NC_047293 | *ndh*D | 48.565 | 0.389 | 0.325 | 0.371 | 0.254 | 0.361 | 0.361 |
| *Delphinium mollifolium*_LHM1295 | *ndh*D | 48.8 | 0.375 | 0.313 | 0.371 | 0.26 | 0.363 | 0.363 |
| *Delphinium naviculare* var. *lasiocarpum*_LHM1293 | *ndh*D | 48.8 | 0.375 | 0.313 | 0.371 | 0.26 | 0.363 | 0.363 |
| *Delphinium sauricum*_LHM1266 | *ndh*D | 48.8 | 0.375 | 0.313 | 0.371 | 0.26 | 0.363 | 0.363 |
| *Delphinium shawurense*_LHM1271 | *ndh*D | 48.799 | 0.374 | 0.313 | 0.371 | 0.26 | 0.363 | 0.363 |
| *Delphinium winklerianum*_LHM1299 | *ndh*D | 48.8 | 0.375 | 0.313 | 0.371 | 0.26 | 0.363 | 0.363 |
| *Delphinium yunnanense*_MW246156 | *ndh*D | 49.19 | 0.371 | 0.308 | 0.371 | 0.26 | 0.363 | 0.363 |
| *Aconitum brachypodum*_MT584424 | *ndh*E | 44.547 | 0.603 | 0.69 | 0.337 | 0.175 | 0.323 | 0.32 |
| *Aconitum delavayi*_OM289058 | *ndh*E | 44.547 | 0.603 | 0.69 | 0.337 | 0.175 | 0.323 | 0.32 |
| *Delphinium aemulans*_LHM1280 | *ndh*E | 43.62 | 0.547 | 0.643 | 0.327 | 0.196 | 0.327 | 0.327 |
| *Delphinium anthriscifolium*_MK253461 | *ndh*E | 42.738 | 0.581 | 0.662 | 0.327 | 0.175 | 0.32 | 0.32 |
| *Delphinium brunonianum*_NC_051554 | *ndh*E | 43.62 | 0.547 | 0.643 | 0.327 | 0.196 | 0.327 | 0.327 |
| *Delphinium candelabrum* var. *monanthum*_MW246165 | *ndh*E | 43.62 | 0.547 | 0.643 | 0.327 | 0.196 | 0.327 | 0.327 |
| *Delphinium ceratophorum*_MK253460 | *ndh*E | 45.644 | 0.531 | 0.615 | 0.327 | 0.206 | 0.33 | 0.33 |
| *Delphinium elatum* var. *sericeum*_LHM1265 | *ndh*E | 42.761 | 0.549 | 0.657 | 0.327 | 0.206 | 0.33 | 0.33 |
| *Delphinium iliense*_LHM1285 | *ndh*E | 43.62 | 0.547 | 0.643 | 0.327 | 0.196 | 0.327 | 0.327 |
| *Delphinium maackianum*_NC_047293 | *ndh*E | 43.62 | 0.547 | 0.643 | 0.327 | 0.196 | 0.327 | 0.327 |
| *Delphinium mollifolium*_LHM1295 | *ndh*E | 43.62 | 0.547 | 0.643 | 0.327 | 0.196 | 0.327 | 0.327 |
| *Delphinium naviculare* var. *lasiocarpum*_LHM1293 | *ndh*E | 43.62 | 0.547 | 0.643 | 0.327 | 0.196 | 0.327 | 0.327 |
| *Delphinium sauricum*_LHM1266 | *ndh*E | 43.62 | 0.547 | 0.643 | 0.327 | 0.196 | 0.327 | 0.327 |
| *Delphinium shawurense*_LHM1271 | *ndh*E | 43.62 | 0.547 | 0.643 | 0.327 | 0.196 | 0.327 | 0.327 |
| *Delphinium winklerianum*_LHM1299 | *ndh*E | 43.62 | 0.547 | 0.643 | 0.327 | 0.196 | 0.327 | 0.327 |
| *Delphinium yunnanense*_MW246156 | *ndh*E | 43.62 | 0.547 | 0.643 | 0.327 | 0.196 | 0.327 | 0.327 |
| *Aconitum brachypodum*_MT584424 | *ndh*F | 44.729 | 0.513 | 0.457 | 0.365 | 0.194 | 0.326 | 0.327 |
| *Aconitum delavayi*_OM289058 | *ndh*F | 44.727 | 0.515 | 0.461 | 0.365 | 0.192 | 0.326 | 0.326 |
| *Delphinium aemulans*_LHM1280 | *ndh*F | 43.276 | 0.537 | 0.49 | 0.372 | 0.19 | 0.327 | 0.327 |
| *Delphinium anthriscifolium*_MK253461 | *ndh*F | 42.987 | 0.538 | 0.5 | 0.364 | 0.182 | 0.32 | 0.319 |
| *Delphinium brunonianum*_NC_051554 | *ndh*F | 43.072 | 0.539 | 0.497 | 0.372 | 0.19 | 0.326 | 0.326 |
| *Delphinium candelabrum* var. *monanthum*_MW246165 | *ndh*F | 43.475 | 0.525 | 0.475 | 0.371 | 0.194 | 0.328 | 0.328 |
| *Delphinium ceratophorum*_MK253460 | *ndh*F | 43.471 | 0.529 | 0.479 | 0.371 | 0.194 | 0.328 | 0.328 |
| *Delphinium elatum* var. *sericeum*_LHM1265 | *ndh*F | 43.29 | 0.535 | 0.488 | 0.372 | 0.192 | 0.328 | 0.328 |
| *Delphinium iliense*_LHM1285 | *ndh*F | 43.472 | 0.527 | 0.476 | 0.372 | 0.196 | 0.329 | 0.329 |
| *Delphinium maackianum*_NC_047293 | *ndh*F | 43.231 | 0.546 | 0.501 | 0.371 | 0.185 | 0.324 | 0.324 |
| *Delphinium mollifolium*_LHM1295 | *ndh*F | 43.224 | 0.535 | 0.487 | 0.372 | 0.192 | 0.328 | 0.328 |
| *Delphinium naviculare* var. *lasiocarpum*_LHM1293 | *ndh*F | 43.419 | 0.529 | 0.478 | 0.372 | 0.194 | 0.328 | 0.328 |
| *Delphinium sauricum*_LHM1266 | *ndh*F | 43.234 | 0.535 | 0.487 | 0.372 | 0.192 | 0.327 | 0.327 |
| *Delphinium shawurense*_LHM1271 | *ndh*F | 43.29 | 0.535 | 0.488 | 0.372 | 0.192 | 0.328 | 0.328 |
| *Delphinium winklerianum*_LHM1299 | *ndh*F | 43.213 | 0.535 | 0.488 | 0.372 | 0.193 | 0.328 | 0.328 |
| *Delphinium yunnanense*_MW246156 | *ndh*F | 43.625 | 0.531 | 0.479 | 0.372 | 0.192 | 0.328 | 0.328 |
| *Aconitum brachypodum*_MT584424 | *ndh*G | 47.328 | 0.494 | 0.511 | 0.356 | 0.241 | 0.363 | 0.361 |
| *Aconitum delavayi*_OM289058 | *ndh*G | 47.364 | 0.494 | 0.512 | 0.356 | 0.241 | 0.362 | 0.36 |
| *Delphinium aemulans*_LHM1280 | *ndh*G | 46.305 | 0.479 | 0.518 | 0.35 | 0.235 | 0.358 | 0.356 |
| *Delphinium anthriscifolium*_MK253461 | *ndh*G | 45.798 | 0.492 | 0.513 | 0.35 | 0.229 | 0.354 | 0.352 |
| *Delphinium brunonianum*_NC_051554 | *ndh*G | 47.029 | 0.472 | 0.497 | 0.345 | 0.235 | 0.356 | 0.354 |
| *Delphinium candelabrum* var. *monanthum*_MW246165 | *ndh*G | 48.119 | 0.48 | 0.496 | 0.356 | 0.235 | 0.36 | 0.358 |
| *Delphinium ceratophorum*_MK253460 | *ndh*G | 48.978 | 0.477 | 0.484 | 0.356 | 0.241 | 0.362 | 0.36 |
| *Delphinium elatum* var. *sericeum*_LHM1265 | *ndh*G | 44.911 | 0.486 | 0.549 | 0.35 | 0.229 | 0.356 | 0.354 |
| *Delphinium iliense*_LHM1285 | *ndh*G | 46.305 | 0.479 | 0.518 | 0.35 | 0.235 | 0.358 | 0.356 |
| *Delphinium maackianum*_NC_047293 | *ndh*G | 46.305 | 0.479 | 0.518 | 0.35 | 0.235 | 0.358 | 0.356 |
| *Delphinium mollifolium*_LHM1295 | *ndh*G | 44.911 | 0.486 | 0.549 | 0.35 | 0.229 | 0.356 | 0.354 |
| *Delphinium naviculare* var. *lasiocarpum*_LHM1293 | *ndh*G | 46.305 | 0.479 | 0.518 | 0.35 | 0.235 | 0.358 | 0.356 |
| *Delphinium sauricum*_LHM1266 | *ndh*G | 44.987 | 0.485 | 0.548 | 0.35 | 0.229 | 0.354 | 0.352 |
| *Delphinium shawurense*_LHM1271 | *ndh*G | 44.911 | 0.486 | 0.549 | 0.35 | 0.229 | 0.356 | 0.354 |
| *Delphinium winklerianum*_LHM1299 | *ndh*G | 44.911 | 0.486 | 0.549 | 0.35 | 0.229 | 0.356 | 0.354 |
| *Delphinium yunnanense*_MW246156 | *ndh*G | 45.908 | 0.486 | 0.527 | 0.356 | 0.229 | 0.358 | 0.356 |
| *Aconitum brachypodum*_MT584424 | *ndh*H | 51.754 | 0.42 | 0.322 | 0.369 | 0.253 | 0.394 | 0.394 |
| *Aconitum delavayi*_OM289058 | *ndh*H | 51.926 | 0.422 | 0.322 | 0.372 | 0.253 | 0.395 | 0.395 |
| *Delphinium aemulans*_LHM1280 | *ndh*H | 50.656 | 0.407 | 0.316 | 0.369 | 0.256 | 0.398 | 0.398 |
| *Delphinium anthriscifolium*_MK253461 | *ndh*H | 53.085 | 0.429 | 0.324 | 0.366 | 0.245 | 0.394 | 0.394 |
| *Delphinium brunonianum*_NC_051554 | *ndh*H | 50.885 | 0.404 | 0.312 | 0.369 | 0.259 | 0.399 | 0.398 |
| *Delphinium candelabrum* var. *monanthum*_MW246165 | *ndh*H | 50.612 | 0.408 | 0.317 | 0.369 | 0.259 | 0.399 | 0.398 |
| *Delphinium ceratophorum*_MK253460 | *ndh*H | 50.612 | 0.405 | 0.316 | 0.372 | 0.256 | 0.399 | 0.398 |
| *Delphinium elatum* var. *sericeum*_LHM1265 | *ndh*H | 50.656 | 0.407 | 0.316 | 0.369 | 0.256 | 0.398 | 0.398 |
| *Delphinium iliense*_LHM1285 | *ndh*H | 50.656 | 0.407 | 0.316 | 0.369 | 0.256 | 0.398 | 0.398 |
| *Delphinium maackianum*_NC_047293 | *ndh*H | 50.656 | 0.407 | 0.316 | 0.369 | 0.256 | 0.398 | 0.398 |
| *Delphinium mollifolium*_LHM1295 | *ndh*H | 50.656 | 0.407 | 0.316 | 0.369 | 0.256 | 0.398 | 0.398 |
| *Delphinium naviculare* var. *lasiocarpum*_LHM1293 | *ndh*H | 50.656 | 0.407 | 0.316 | 0.369 | 0.256 | 0.398 | 0.398 |
| *Delphinium sauricum*_LHM1266 | *ndh*H | 50.656 | 0.407 | 0.316 | 0.369 | 0.256 | 0.398 | 0.398 |
| *Delphinium shawurense*_LHM1271 | *ndh*H | 50.656 | 0.407 | 0.316 | 0.369 | 0.256 | 0.398 | 0.398 |
| *Delphinium winklerianum*_LHM1299 | *ndh*H | 50.656 | 0.407 | 0.316 | 0.369 | 0.256 | 0.398 | 0.398 |
| *Delphinium yunnanense*_MW246156 | *ndh*H | 50.612 | 0.408 | 0.317 | 0.369 | 0.259 | 0.399 | 0.398 |
| *Aconitum brachypodum*_MT584424 | *ndh*I | 51.526 | 0.49 | 0.441 | 0.394 | 0.262 | 0.369 | 0.366 |
| *Aconitum delavayi*_OM289058 | *ndh*I | 51.526 | 0.49 | 0.441 | 0.394 | 0.262 | 0.369 | 0.366 |
| *Delphinium aemulans*_LHM1280 | *ndh*I | 50.729 | 0.549 | 0.516 | 0.399 | 0.246 | 0.363 | 0.359 |
| *Delphinium anthriscifolium*_MK253461 | *ndh*I | 50.53 | 0.519 | 0.497 | 0.406 | 0.25 | 0.369 | 0.366 |
| *Delphinium brunonianum*_NC_051554 | *ndh*I | 51.465 | 0.519 | 0.47 | 0.396 | 0.263 | 0.364 | 0.362 |
| *Delphinium candelabrum* var. *monanthum*_MW246165 | *ndh*I | 52.236 | 0.513 | 0.459 | 0.399 | 0.257 | 0.367 | 0.363 |
| *Delphinium ceratophorum*_MK253460 | *ndh*I | 51.504 | 0.519 | 0.469 | 0.399 | 0.263 | 0.369 | 0.365 |
| *Delphinium elatum* var. *sericeum*_LHM1265 | *ndh*I | 50.729 | 0.549 | 0.516 | 0.399 | 0.246 | 0.363 | 0.359 |
| *Delphinium iliense*_LHM1285 | *ndh*I | 51.171 | 0.533 | 0.486 | 0.399 | 0.257 | 0.367 | 0.363 |
| *Delphinium maackianum*_NC_047293 | *ndh*I | 51.504 | 0.519 | 0.469 | 0.399 | 0.263 | 0.369 | 0.365 |
| *Delphinium mollifolium*_LHM1295 | *ndh*I | 51.171 | 0.533 | 0.486 | 0.399 | 0.257 | 0.367 | 0.363 |
| *Delphinium naviculare* var. *lasiocarpum*_LHM1293 | *ndh*I | 51.171 | 0.533 | 0.486 | 0.399 | 0.257 | 0.367 | 0.363 |
| *Delphinium sauricum*_LHM1266 | *ndh*I | 51.171 | 0.533 | 0.486 | 0.399 | 0.257 | 0.367 | 0.363 |
| *Delphinium shawurense*_LHM1271 | *ndh*I | 50.729 | 0.549 | 0.516 | 0.399 | 0.246 | 0.363 | 0.359 |
| *Delphinium winklerianum*_LHM1299 | *ndh*I | 51.171 | 0.533 | 0.486 | 0.399 | 0.257 | 0.367 | 0.363 |
| *Delphinium yunnanense*_MW246156 | *ndh*I | 51.504 | 0.519 | 0.469 | 0.399 | 0.263 | 0.369 | 0.365 |
| *Aconitum brachypodum*_MT584424 | *ndh*J | 58.985 | 0.398 | 0.347 | 0.38 | 0.309 | 0.411 | 0.411 |
| *Aconitum delavayi*_OM289058 | *ndh*J | 58.985 | 0.398 | 0.347 | 0.38 | 0.309 | 0.411 | 0.411 |
| *Delphinium aemulans*_LHM1280 | *ndh*J | 57.198 | 0.424 | 0.372 | 0.386 | 0.295 | 0.407 | 0.407 |
| *Delphinium anthriscifolium*_MK253461 | *ndh*J | 60.558 | 0.408 | 0.332 | 0.38 | 0.309 | 0.411 | 0.411 |
| *Delphinium brunonianum*_NC_051554 | *ndh*J | 57.198 | 0.424 | 0.372 | 0.386 | 0.295 | 0.407 | 0.407 |
| *Delphinium candelabrum* var. *monanthum*_MW246165 | *ndh*J | 57.198 | 0.424 | 0.372 | 0.386 | 0.295 | 0.407 | 0.407 |
| *Delphinium ceratophorum*_MK253460 | *ndh*J | 55.774 | 0.434 | 0.403 | 0.386 | 0.295 | 0.405 | 0.405 |
| *Delphinium elatum* var. *sericeum*_LHM1265 | *ndh*J | 57.198 | 0.424 | 0.372 | 0.386 | 0.295 | 0.407 | 0.407 |
| *Delphinium iliense*_LHM1285 | *ndh*J | 57.198 | 0.424 | 0.372 | 0.386 | 0.295 | 0.407 | 0.407 |
| *Delphinium maackianum*_NC_047293 | *ndh*J | 58.205 | 0.422 | 0.361 | 0.38 | 0.295 | 0.405 | 0.405 |
| *Delphinium mollifolium*_LHM1295 | *ndh*J | 57.198 | 0.424 | 0.372 | 0.386 | 0.295 | 0.407 | 0.407 |
| *Delphinium naviculare* var. *lasiocarpum*_LHM1293 | *ndh*J | 57.198 | 0.424 | 0.372 | 0.386 | 0.295 | 0.407 | 0.407 |
| *Delphinium sauricum*_LHM1266 | *ndh*J | 57.198 | 0.424 | 0.372 | 0.386 | 0.295 | 0.407 | 0.407 |
| *Delphinium shawurense*_LHM1271 | *ndh*J | 57.198 | 0.424 | 0.372 | 0.386 | 0.295 | 0.407 | 0.407 |
| *Delphinium winklerianum*_LHM1299 | *ndh*J | 57.198 | 0.424 | 0.372 | 0.386 | 0.295 | 0.407 | 0.407 |
| *Delphinium yunnanense*_MW246156 | *ndh*J | 57.198 | 0.424 | 0.372 | 0.386 | 0.295 | 0.407 | 0.407 |
| *Aconitum brachypodum*_MT584424 | *ndh*K | 53.198 | 0.422 | 0.366 | 0.454 | 0.283 | 0.404 | 0.404 |
| *Aconitum delavayi*_OM289058 | *ndh*K | 53.198 | 0.422 | 0.366 | 0.454 | 0.283 | 0.404 | 0.404 |
| *Delphinium aemulans*_LHM1280 | *ndh*K | 52.837 | 0.411 | 0.369 | 0.449 | 0.274 | 0.398 | 0.398 |
| *Delphinium anthriscifolium*_MK253461 | *ndh*K | 51.313 | 0.44 | 0.404 | 0.445 | 0.269 | 0.395 | 0.395 |
| *Delphinium brunonianum*_NC_051554 | *ndh*K | 52.837 | 0.411 | 0.369 | 0.449 | 0.274 | 0.398 | 0.398 |
| *Delphinium candelabrum* var. *monanthum*_MW246165 | *ndh*K | 52.837 | 0.411 | 0.369 | 0.449 | 0.274 | 0.398 | 0.398 |
| *Delphinium ceratophorum*_MK253460 | *ndh*K | 52.837 | 0.411 | 0.369 | 0.449 | 0.274 | 0.398 | 0.398 |
| *Delphinium elatum* var. *sericeum*_LHM1265 | *ndh*K | 52.837 | 0.411 | 0.369 | 0.449 | 0.274 | 0.398 | 0.398 |
| *Delphinium iliense*_LHM1285 | *ndh*K | 52.837 | 0.411 | 0.369 | 0.449 | 0.274 | 0.398 | 0.398 |
| *Delphinium maackianum*_NC_047293 | *ndh*K | 52.837 | 0.411 | 0.369 | 0.449 | 0.274 | 0.398 | 0.398 |
| *Delphinium mollifolium*_LHM1295 | *ndh*K | 52.837 | 0.411 | 0.369 | 0.449 | 0.274 | 0.398 | 0.398 |
| *Delphinium naviculare* var. *lasiocarpum*_LHM1293 | *ndh*K | 52.837 | 0.411 | 0.369 | 0.449 | 0.274 | 0.398 | 0.398 |
| *Delphinium sauricum*_LHM1266 | *ndh*K | 52.837 | 0.411 | 0.369 | 0.449 | 0.274 | 0.398 | 0.398 |
| *Delphinium shawurense*_LHM1271 | *ndh*K | 52.837 | 0.411 | 0.369 | 0.449 | 0.274 | 0.398 | 0.398 |
| *Delphinium winklerianum*_LHM1299 | *ndh*K | 52.837 | 0.411 | 0.369 | 0.449 | 0.274 | 0.398 | 0.398 |
| *Delphinium yunnanense*_MW246156 | *ndh*K | 52.837 | 0.411 | 0.369 | 0.449 | 0.274 | 0.398 | 0.398 |
| *Aconitum brachypodum*_MT584424 | *pet*A | 50.056 | 0.387 | 0.327 | 0.363 | 0.289 | 0.39 | 0.39 |
| *Aconitum delavayi*_OM289058 | *pet*A | 50.056 | 0.387 | 0.327 | 0.363 | 0.289 | 0.39 | 0.39 |
| *Delphinium aemulans*_LHM1280 | *pet*A | 48.822 | 0.378 | 0.338 | 0.366 | 0.297 | 0.392 | 0.392 |
| *Delphinium anthriscifolium*_MK253461 | *pet*A | 51.318 | 0.378 | 0.311 | 0.366 | 0.305 | 0.394 | 0.394 |
| *Delphinium brunonianum*_NC_051554 | *pet*A | 48.708 | 0.385 | 0.343 | 0.366 | 0.291 | 0.39 | 0.39 |
| *Delphinium candelabrum* var. *monanthum*_MW246165 | *pet*A | 48.633 | 0.385 | 0.344 | 0.366 | 0.294 | 0.391 | 0.391 |
| *Delphinium ceratophorum*_MK253460 | *pet*A | 48.633 | 0.385 | 0.344 | 0.366 | 0.294 | 0.391 | 0.391 |
| *Delphinium elatum* var. *sericeum*_LHM1265 | *pet*A | 48.822 | 0.378 | 0.338 | 0.366 | 0.297 | 0.392 | 0.392 |
| *Delphinium iliense*_LHM1285 | *pet*A | 48.822 | 0.378 | 0.338 | 0.366 | 0.297 | 0.392 | 0.392 |
| *Delphinium maackianum*_NC_047293 | *pet*A | 48.633 | 0.385 | 0.344 | 0.366 | 0.294 | 0.391 | 0.391 |
| *Delphinium mollifolium*_LHM1295 | *pet*A | 48.822 | 0.378 | 0.338 | 0.366 | 0.297 | 0.392 | 0.392 |
| *Delphinium naviculare* var. *lasiocarpum*_LHM1293 | *pet*A | 48.822 | 0.378 | 0.338 | 0.366 | 0.297 | 0.392 | 0.392 |
| *Delphinium sauricum*_LHM1266 | *pet*A | 48.822 | 0.378 | 0.338 | 0.366 | 0.297 | 0.392 | 0.392 |
| *Delphinium shawurense*_LHM1271 | *pet*A | 48.695 | 0.384 | 0.344 | 0.366 | 0.294 | 0.391 | 0.391 |
| *Delphinium winklerianum*_LHM1299 | *pet*A | 49.601 | 0.373 | 0.324 | 0.366 | 0.301 | 0.393 | 0.393 |
| *Delphinium yunnanense*_MW246156 | *pet*A | 48.597 | 0.386 | 0.345 | 0.366 | 0.291 | 0.39 | 0.39 |
| *Aconitum brachypodum*_MT584424 | *pet*B | 42.558 | 0.498 | 0.548 | 0.419 | 0.281 | 0.412 | 0.412 |
| *Aconitum delavayi*_OM289058 | *pet*B | 42.558 | 0.498 | 0.548 | 0.419 | 0.281 | 0.412 | 0.412 |
| *Delphinium aemulans*_LHM1280 | *pet*B | 44.775 | 0.482 | 0.49 | 0.419 | 0.291 | 0.417 | 0.417 |
| *Delphinium anthriscifolium*_MK253461 | *pet*B | 44.376 | 0.486 | 0.508 | 0.419 | 0.281 | 0.414 | 0.414 |
| *Delphinium brunonianum*_NC_051554 | *pet*B | 44.775 | 0.482 | 0.49 | 0.419 | 0.291 | 0.417 | 0.417 |
| *Delphinium candelabrum* var. *monanthum*_MW246165 | *pet*B | 44.775 | 0.482 | 0.49 | 0.419 | 0.291 | 0.417 | 0.417 |
| *Delphinium ceratophorum*_MK253460 | *pet*B | 44.775 | 0.482 | 0.49 | 0.419 | 0.291 | 0.417 | 0.417 |
| *Delphinium elatum* var. *sericeum*_LHM1265 | *pet*B | 44.775 | 0.482 | 0.49 | 0.419 | 0.291 | 0.417 | 0.417 |
| *Delphinium iliense*_LHM1285 | *pet*B | 44.945 | 0.472 | 0.482 | 0.419 | 0.296 | 0.419 | 0.418 |
| *Delphinium maackianum*_NC_047293 | *pet*B | 44.775 | 0.482 | 0.49 | 0.419 | 0.291 | 0.417 | 0.417 |
| *Delphinium mollifolium*_LHM1295 | *pet*B | 44.775 | 0.482 | 0.49 | 0.419 | 0.291 | 0.417 | 0.417 |
| *Delphinium naviculare* var. *lasiocarpum*_LHM1293 | *pet*B | 44.945 | 0.472 | 0.482 | 0.419 | 0.296 | 0.419 | 0.418 |
| *Delphinium sauricum*_LHM1266 | *pet*B | 44.775 | 0.482 | 0.49 | 0.419 | 0.291 | 0.417 | 0.417 |
| *Delphinium shawurense*_LHM1271 | *pet*B | 44.493 | 0.484 | 0.499 | 0.419 | 0.291 | 0.417 | 0.417 |
| *Delphinium winklerianum*_LHM1299 | *pet*B | 44.775 | 0.482 | 0.49 | 0.419 | 0.291 | 0.417 | 0.417 |
| *Delphinium yunnanense*_MW246156 | *pet*B | 44.775 | 0.482 | 0.49 | 0.419 | 0.291 | 0.417 | 0.417 |
| *Aconitum brachypodum*_MT584424 | *pet*D | 41.616 | 0.577 | 0.681 | 0.389 | 0.219 | 0.381 | 0.379 |
| *Aconitum delavayi*_OM289058 | *pet*D | 40.658 | 0.585 | 0.704 | 0.389 | 0.219 | 0.383 | 0.381 |
| *Delphinium aemulans*_LHM1280 | *pet*D | 42.678 | 0.55 | 0.596 | 0.383 | 0.231 | 0.385 | 0.383 |
| *Delphinium anthriscifolium*_MK253461 | *pet*D | 42.156 | 0.553 | 0.644 | 0.383 | 0.231 | 0.385 | 0.383 |
| *Delphinium brunonianum*_NC_051554 | *pet*D | 42.717 | 0.545 | 0.599 | 0.383 | 0.231 | 0.385 | 0.383 |
| *Delphinium candelabrum* var. *monanthum*_MW246165 | *pet*D | 42.232 | 0.56 | 0.616 | 0.383 | 0.225 | 0.383 | 0.381 |
| *Delphinium ceratophorum*_MK253460 | *pet*D | 42.772 | 0.543 | 0.6 | 0.383 | 0.225 | 0.383 | 0.381 |
| *Delphinium elatum* var. *sericeum*_LHM1265 | *pet*D | 42.678 | 0.55 | 0.596 | 0.383 | 0.231 | 0.385 | 0.383 |
| *Delphinium iliense*_LHM1285 | *pet*D | 42.232 | 0.56 | 0.616 | 0.383 | 0.225 | 0.383 | 0.381 |
| *Delphinium maackianum*_NC_047293 | *pet*D | 42.909 | 0.55 | 0.593 | 0.383 | 0.231 | 0.385 | 0.383 |
| *Delphinium mollifolium*_LHM1295 | *pet*D | 42.049 | 0.569 | 0.625 | 0.383 | 0.231 | 0.385 | 0.383 |
| *Delphinium naviculare* var. *lasiocarpum*_LHM1293 | *pet*D | 42.232 | 0.56 | 0.616 | 0.383 | 0.225 | 0.383 | 0.381 |
| *Delphinium sauricum*_LHM1266 | *pet*D | 42.232 | 0.56 | 0.616 | 0.383 | 0.225 | 0.383 | 0.381 |
| *Delphinium shawurense*_LHM1271 | *pet*D | 42.678 | 0.55 | 0.596 | 0.383 | 0.231 | 0.385 | 0.383 |
| *Delphinium winklerianum*_LHM1299 | *pet*D | 42.232 | 0.56 | 0.616 | 0.383 | 0.225 | 0.383 | 0.381 |
| *Delphinium yunnanense*_MW246156 | *pet*D | 42.232 | 0.56 | 0.616 | 0.383 | 0.225 | 0.383 | 0.381 |
| *Aconitum brachypodum*_MT584424 | *pet*G |  | 0.502 | 0.856 | 0.297 | 0.361 | 0.396 | 0.395 |
| *Aconitum delavayi*_OM289058 | *pet*G |  | 0.502 | 0.856 | 0.297 | 0.361 | 0.396 | 0.395 |
| *Delphinium aemulans*_LHM1280 | *pet*G | 35.929 | 0.558 | 0.912 | 0.297 | 0.333 | 0.387 | 0.386 |
| *Delphinium anthriscifolium*_MK253461 | *pet*G |  | 0.502 | 0.856 | 0.297 | 0.361 | 0.396 | 0.395 |
| *Delphinium brunonianum*_NC_051554 | *pet*G | 35.929 | 0.558 | 0.912 | 0.297 | 0.333 | 0.387 | 0.386 |
| *Delphinium candelabrum* var. *monanthum*_MW246165 | *pet*G | 35.929 | 0.558 | 0.912 | 0.297 | 0.333 | 0.396 | 0.395 |
| *Delphinium ceratophorum*_MK253460 | *pet*G | 35.929 | 0.558 | 0.912 | 0.297 | 0.333 | 0.387 | 0.386 |
| *Delphinium elatum* var. *sericeum*_LHM1265 | *pet*G | 35.929 | 0.558 | 0.912 | 0.297 | 0.333 | 0.387 | 0.386 |
| *Delphinium iliense*_LHM1285 | *pet*G | 35.929 | 0.558 | 0.912 | 0.297 | 0.333 | 0.387 | 0.386 |
| *Delphinium maackianum*_NC_047293 | *pet*G | 35.929 | 0.558 | 0.912 | 0.297 | 0.333 | 0.387 | 0.386 |
| *Delphinium mollifolium*_LHM1295 | *pet*G | 35.929 | 0.558 | 0.912 | 0.297 | 0.333 | 0.387 | 0.386 |
| *Delphinium naviculare* var. *lasiocarpum*_LHM1293 | *pet*G | 35.929 | 0.558 | 0.912 | 0.297 | 0.333 | 0.387 | 0.386 |
| *Delphinium sauricum*_LHM1266 | *pet*G | 35.929 | 0.558 | 0.912 | 0.297 | 0.333 | 0.387 | 0.386 |
| *Delphinium shawurense*_LHM1271 | *pet*G | 35.929 | 0.558 | 0.912 | 0.297 | 0.333 | 0.387 | 0.386 |
| *Delphinium winklerianum*_LHM1299 | *pet*G | 35.929 | 0.558 | 0.912 | 0.297 | 0.333 | 0.387 | 0.386 |
| *Delphinium yunnanense*_MW246156 | *pet*G | 35.929 | 0.558 | 0.912 | 0.297 | 0.333 | 0.387 | 0.386 |
| *Aconitum brachypodum*_MT584424 | *pet*L | 60.429 | 0.686 | 1.393 | 0.452 | 0.2 | 0.355 | 0.354 |
| *Aconitum delavayi*_OM289058 | *pet*L | 60.429 | 0.686 | 1.393 | 0.452 | 0.2 | 0.355 | 0.354 |
| *Delphinium aemulans*_LHM1280 | *pet*L | 60.429 | 0.686 | 1.393 | 0.452 | 0.2 | 0.355 | 0.354 |
| *Delphinium anthriscifolium*_MK253461 | *pet*L | 60.429 | 0.686 | 1.393 | 0.452 | 0.2 | 0.355 | 0.354 |
| *Delphinium brunonianum*_NC_051554 | *pet*L | 60.429 | 0.686 | 1.393 | 0.452 | 0.2 | 0.355 | 0.354 |
| *Delphinium candelabrum* var. *monanthum*_MW246165 | *pet*L | 60.429 | 0.686 | 1.393 | 0.452 | 0.233 | 0.366 | 0.365 |
| *Delphinium ceratophorum*_MK253460 | *pet*L | 60.429 | 0.686 | 1.393 | 0.452 | 0.2 | 0.355 | 0.354 |
| *Delphinium elatum* var. *sericeum*_LHM1265 | *pet*L | 60.429 | 0.686 | 1.393 | 0.452 | 0.167 | 0.344 | 0.344 |
| *Delphinium iliense*_LHM1285 | *pet*L | 60.429 | 0.686 | 1.393 | 0.452 | 0.2 | 0.355 | 0.354 |
| *Delphinium maackianum*_NC_047293 | *pet*L | 60.429 | 0.686 | 1.393 | 0.452 | 0.2 | 0.355 | 0.354 |
| *Delphinium mollifolium*_LHM1295 | *pet*L | 60.429 | 0.686 | 1.393 | 0.452 | 0.2 | 0.355 | 0.354 |
| *Delphinium naviculare* var. *lasiocarpum*_LHM1293 | *pet*L | 60.429 | 0.686 | 1.393 | 0.452 | 0.2 | 0.355 | 0.354 |
| *Delphinium sauricum*_LHM1266 | *pet*L | 60.429 | 0.686 | 1.393 | 0.452 | 0.2 | 0.355 | 0.354 |
| *Delphinium shawurense*_LHM1271 | *pet*L | 60.429 | 0.686 | 1.393 | 0.452 | 0.2 | 0.355 | 0.354 |
| *Delphinium winklerianum*_LHM1299 | *pet*L | 61 | 0.614 | 1.313 | 0.452 | 0.233 | 0.366 | 0.365 |
| *Delphinium yunnanense*_MW246156 | *pet*L | 60.429 | 0.686 | 1.393 | 0.452 | 0.233 | 0.366 | 0.365 |
| *Aconitum brachypodum*_MT584424 | *pet*N | 34.204 | 0.853 | 1.896 | 0.448 | 0.16 | 0.414 | 0.411 |
| *Aconitum delavayi*_OM289058 | *pet*N | 34.204 | 0.853 | 1.896 | 0.448 | 0.16 | 0.414 | 0.411 |
| *Delphinium aemulans*_LHM1280 | *pet*N | 34.204 | 0.853 | 1.896 | 0.448 | 0.16 | 0.414 | 0.411 |
| *Delphinium anthriscifolium*_MK253461 | *pet*N | 34.204 | 0.853 | 1.896 | 0.448 | 0.16 | 0.414 | 0.4 |
| *Delphinium brunonianum*_NC_051554 | *pet*N | 34.204 | 0.853 | 1.896 | 0.448 | 0.16 | 0.414 | 0.411 |
| *Delphinium candelabrum* var. *monanthum*_MW246165 | *pet*N | 34.204 | 0.853 | 1.896 | 0.448 | 0.16 | 0.414 | 0.411 |
| *Delphinium ceratophorum*_MK253460 | *pet*N | 34.204 | 0.853 | 1.896 | 0.448 | 0.16 | 0.414 | 0.411 |
| *Delphinium elatum* var. *sericeum*_LHM1265 | *pet*N | 34.204 | 0.853 | 1.896 | 0.448 | 0.16 | 0.414 | 0.411 |
| *Delphinium iliense*_LHM1285 | *pet*N | 34.204 | 0.853 | 1.896 | 0.448 | 0.16 | 0.414 | 0.411 |
| *Delphinium maackianum*_NC_047293 | *pet*N | 34.204 | 0.853 | 1.896 | 0.448 | 0.16 | 0.414 | 0.411 |
| *Delphinium mollifolium*_LHM1295 | *pet*N | 34.204 | 0.853 | 1.896 | 0.448 | 0.16 | 0.414 | 0.411 |
| *Delphinium naviculare* var. *lasiocarpum*_LHM1293 | *pet*N | 34.204 | 0.853 | 1.896 | 0.448 | 0.16 | 0.414 | 0.411 |
| *Delphinium sauricum*_LHM1266 | *pet*N | 34.204 | 0.853 | 1.896 | 0.448 | 0.16 | 0.414 | 0.411 |
| *Delphinium shawurense*_LHM1271 | *pet*N | 34.204 | 0.853 | 1.896 | 0.448 | 0.16 | 0.414 | 0.411 |
| *Delphinium winklerianum*_LHM1299 | *pet*N | 34.204 | 0.853 | 1.896 | 0.448 | 0.16 | 0.414 | 0.411 |
| *Delphinium yunnanense*_MW246156 | *pet*N | 34.204 | 0.853 | 1.896 | 0.448 | 0.16 | 0.414 | 0.411 |
| *Aconitum brachypodum*_MT584424 | *psa*A | 49.387 | 0.343 | 0.253 | 0.435 | 0.294 | 0.43 | 0.43 |
| *Aconitum delavayi*_OM289058 | *psa*A | 49.387 | 0.343 | 0.253 | 0.435 | 0.294 | 0.43 | 0.43 |
| *Delphinium aemulans*_LHM1280 | *psa*A | 48.61 | 0.358 | 0.272 | 0.435 | 0.287 | 0.429 | 0.429 |
| *Delphinium anthriscifolium*_MK253461 | *psa*A | 49.73 | 0.338 | 0.246 | 0.435 | 0.297 | 0.432 | 0.432 |
| *Delphinium brunonianum*_NC_051554 | *psa*A | 48.61 | 0.358 | 0.272 | 0.435 | 0.287 | 0.429 | 0.429 |
| *Delphinium candelabrum* var. *monanthum*_MW246165 | *psa*A | 48.684 | 0.355 | 0.27 | 0.435 | 0.289 | 0.43 | 0.429 |
| *Delphinium ceratophorum*_MK253460 | *psa*A | 48.499 | 0.361 | 0.277 | 0.435 | 0.286 | 0.429 | 0.428 |
| *Delphinium elatum* var. *sericeum*_LHM1265 | *psa*A | 48.61 | 0.358 | 0.272 | 0.435 | 0.287 | 0.429 | 0.429 |
| *Delphinium iliense*_LHM1285 | *psa*A | 48.625 | 0.358 | 0.272 | 0.435 | 0.289 | 0.43 | 0.429 |
| *Delphinium maackianum*_NC_047293 | *psa*A | 48.61 | 0.358 | 0.272 | 0.435 | 0.287 | 0.429 | 0.429 |
| *Delphinium mollifolium*_LHM1295 | *psa*A | 48.625 | 0.358 | 0.272 | 0.435 | 0.289 | 0.43 | 0.429 |
| *Delphinium naviculare* var. *lasiocarpum*_LHM1293 | *psa*A | 48.625 | 0.358 | 0.272 | 0.435 | 0.289 | 0.43 | 0.429 |
| *Delphinium sauricum*_LHM1266 | *psa*A | 48.625 | 0.358 | 0.272 | 0.435 | 0.289 | 0.43 | 0.429 |
| *Delphinium shawurense*_LHM1271 | *psa*A | 48.534 | 0.362 | 0.275 | 0.435 | 0.286 | 0.429 | 0.428 |
| *Delphinium winklerianum*_LHM1299 | *psa*A | 48.625 | 0.358 | 0.272 | 0.435 | 0.289 | 0.43 | 0.429 |
| *Delphinium yunnanense*_MW246156 | *psa*A | 48.684 | 0.355 | 0.27 | 0.435 | 0.289 | 0.43 | 0.429 |
| *Aconitum brachypodum*_MT584424 | *psa*B | 51.239 | 0.365 | 0.29 | 0.431 | 0.293 | 0.419 | 0.418 |
| *Aconitum delavayi*_OM289058 | *psa*B | 51.074 | 0.366 | 0.291 | 0.432 | 0.293 | 0.419 | 0.419 |
| *Delphinium aemulans*_LHM1280 | *psa*B | 51.08 | 0.358 | 0.287 | 0.432 | 0.296 | 0.42 | 0.42 |
| *Delphinium anthriscifolium*_MK253461 | *psa*B | 51.276 | 0.351 | 0.279 | 0.432 | 0.295 | 0.42 | 0.42 |
| *Delphinium brunonianum*_NC_051554 | *psa*B | 51.306 | 0.35 | 0.281 | 0.432 | 0.302 | 0.422 | 0.421 |
| *Delphinium candelabrum* var. *monanthum*_MW246165 | *psa*B | 51.258 | 0.355 | 0.284 | 0.432 | 0.295 | 0.42 | 0.419 |
| *Delphinium ceratophorum*_MK253460 | *psa*B | 51.561 | 0.352 | 0.28 | 0.432 | 0.299 | 0.421 | 0.42 |
| *Delphinium elatum* var. *sericeum*_LHM1265 | *psa*B | 51.08 | 0.358 | 0.287 | 0.432 | 0.296 | 0.42 | 0.42 |
| *Delphinium iliense*_LHM1285 | *psa*B | 51.08 | 0.358 | 0.287 | 0.432 | 0.296 | 0.42 | 0.42 |
| *Delphinium maackianum*_NC_047293 | *psa*B | 51.267 | 0.355 | 0.283 | 0.432 | 0.298 | 0.421 | 0.42 |
| *Delphinium mollifolium*_LHM1295 | *psa*B | 51.08 | 0.358 | 0.287 | 0.432 | 0.296 | 0.42 | 0.42 |
| *Delphinium naviculare* var. *lasiocarpum*_LHM1293 | *psa*B | 51.08 | 0.358 | 0.287 | 0.432 | 0.296 | 0.42 | 0.42 |
| *Delphinium sauricum*_LHM1266 | *psa*B | 51.007 | 0.358 | 0.288 | 0.432 | 0.298 | 0.421 | 0.42 |
| *Delphinium shawurense*_LHM1271 | *psa*B | 51.08 | 0.358 | 0.287 | 0.432 | 0.296 | 0.42 | 0.42 |
| *Delphinium winklerianum*_LHM1299 | *psa*B | 51.007 | 0.358 | 0.288 | 0.432 | 0.298 | 0.421 | 0.42 |
| *Delphinium yunnanense*_MW246156 | *psa*B | 51.32 | 0.353 | 0.282 | 0.432 | 0.296 | 0.42 | 0.42 |
| *Aconitum brachypodum*_MT584424 | *psa*C | 47.661 | 0.623 | 0.732 | 0.5 | 0.202 | 0.4 | 0.399 |
| *Aconitum delavayi*_OM289058 | *psa*C | 47.661 | 0.623 | 0.732 | 0.5 | 0.202 | 0.4 | 0.399 |
| *Delphinium aemulans*_LHM1280 | *psa*C | 47.445 | 0.562 | 0.615 | 0.489 | 0.25 | 0.411 | 0.41 |
| *Delphinium anthriscifolium*_MK253461 | *psa*C | 48.208 | 0.51 | 0.568 | 0.489 | 0.253 | 0.411 | 0.41 |
| *Delphinium brunonianum*_NC_051554 | *psa*C | 46.758 | 0.594 | 0.663 | 0.489 | 0.238 | 0.411 | 0.41 |
| *Delphinium candelabrum* var. *monanthum*_MW246165 | *psa*C | 45.57 | 0.57 | 0.639 | 0.489 | 0.238 | 0.407 | 0.407 |
| *Delphinium ceratophorum*_MK253460 | *psa*C | 47.445 | 0.562 | 0.615 | 0.489 | 0.25 | 0.411 | 0.41 |
| *Delphinium elatum* var. *sericeum*_LHM1265 | *psa*C | 47.445 | 0.562 | 0.615 | 0.489 | 0.25 | 0.411 | 0.41 |
| *Delphinium iliense*_LHM1285 | *psa*C | 47.445 | 0.562 | 0.615 | 0.489 | 0.238 | 0.407 | 0.407 |
| *Delphinium maackianum*_NC_047293 | *psa*C | 47.445 | 0.562 | 0.615 | 0.489 | 0.25 | 0.411 | 0.41 |
| *Delphinium mollifolium*_LHM1295 | *psa*C | 47.445 | 0.562 | 0.615 | 0.489 | 0.238 | 0.407 | 0.407 |
| *Delphinium naviculare* var. *lasiocarpum*_LHM1293 | *psa*C | 47.445 | 0.562 | 0.615 | 0.489 | 0.238 | 0.407 | 0.407 |
| *Delphinium sauricum*_LHM1266 | *psa*C | 47.445 | 0.562 | 0.615 | 0.489 | 0.238 | 0.407 | 0.407 |
| *Delphinium shawurense*_LHM1271 | *psa*C | 54.767 | 0.54 | 0.604 | 0.531 | 0.263 | 0.432 | 0.431 |
| *Delphinium winklerianum*_LHM1299 | *psa*C | 47.445 | 0.562 | 0.615 | 0.489 | 0.238 | 0.407 | 0.407 |
| *Delphinium yunnanense*_MW246156 | *psa*C | 47.445 | 0.562 | 0.615 | 0.489 | 0.25 | 0.411 | 0.41 |
| *Aconitum brachypodum*_MT584424 | *psa*I | 38.536 | 0.691 | 1.384 | 0.333 | 0.265 | 0.343 | 0.342 |
| *Aconitum delavayi*_OM289058 | *psa*I | 38.536 | 0.691 | 1.384 | 0.333 | 0.265 | 0.343 | 0.342 |
| *Delphinium aemulans*_LHM1280 | *psa*I | 28.87 | 0.64 | 1.139 | 0.278 | 0.314 | 0.333 | 0.333 |
| *Delphinium anthriscifolium*_MK253461 | *psa*I | 38.536 | 0.691 | 1.384 | 0.333 | 0.265 | 0.343 | 0.342 |
| *Delphinium brunonianum*_NC_051554 | *psa*I | 28.87 | 0.64 | 1.139 | 0.278 | 0.314 | 0.333 | 0.333 |
| *Delphinium candelabrum* var. *monanthum*_MW246165 | *psa*I | 28.87 | 0.64 | 1.139 | 0.278 | 0.314 | 0.333 | 0.333 |
| *Delphinium ceratophorum*_MK253460 | *psa*I | 28.87 | 0.64 | 1.139 | 0.278 | 0.314 | 0.333 | 0.333 |
| *Delphinium elatum* var. *sericeum*_LHM1265 | *psa*I | 28.87 | 0.64 | 1.139 | 0.278 | 0.314 | 0.333 | 0.333 |
| *Delphinium iliense*_LHM1285 | *psa*I | 28.87 | 0.64 | 1.139 | 0.278 | 0.314 | 0.333 | 0.333 |
| *Delphinium maackianum*_NC_047293 | *psa*I | 28.87 | 0.64 | 1.139 | 0.278 | 0.314 | 0.333 | 0.333 |
| *Delphinium mollifolium*_LHM1295 | *psa*I | 28.87 | 0.64 | 1.139 | 0.278 | 0.314 | 0.333 | 0.333 |
| *Delphinium naviculare* var. *lasiocarpum*_LHM1293 | *psa*I | 28.87 | 0.64 | 1.139 | 0.278 | 0.314 | 0.333 | 0.333 |
| *Delphinium sauricum*_LHM1266 | *psa*I | 28.87 | 0.64 | 1.139 | 0.278 | 0.314 | 0.333 | 0.333 |
| *Delphinium shawurense*_LHM1271 | *psa*I | 28.87 | 0.64 | 1.139 | 0.278 | 0.314 | 0.333 | 0.333 |
| *Delphinium winklerianum*_LHM1299 | *psa*I | 28.87 | 0.64 | 1.139 | 0.278 | 0.314 | 0.333 | 0.333 |
| *Delphinium yunnanense*_MW246156 | *psa*I | 28.87 | 0.64 | 1.139 | 0.278 | 0.314 | 0.333 | 0.333 |
| *Aconitum brachypodum*_MT584424 | *psa*J | 45.657 | 0.631 | 0.905 | 0.409 | 0.357 | 0.402 | 0.4 |
| *Aconitum delavayi*_OM289058 | *psa*J | 45.657 | 0.631 | 0.905 | 0.409 | 0.357 | 0.402 | 0.4 |
| *Delphinium aemulans*_LHM1280 | *psa*J | 41.257 | 0.631 | 0.996 | 0.409 | 0.31 | 0.386 | 0.385 |
| *Delphinium anthriscifolium*_MK253461 | *psa*J | 40.394 | 0.681 | 1.067 | 0.422 | 0.326 | 0.4 | 0.399 |
| *Delphinium brunonianum*_NC_051554 | *psa*J | 42.257 | 0.661 | 1.06 | 0.409 | 0.31 | 0.386 | 0.385 |
| *Delphinium candelabrum* var. *monanthum*_MW246165 | *psa*J | 42.257 | 0.661 | 1.06 | 0.409 | 0.31 | 0.386 | 0.385 |
| *Delphinium ceratophorum*_MK253460 | *psa*J | 41.257 | 0.631 | 0.996 | 0.409 | 0.286 | 0.379 | 0.378 |
| *Delphinium elatum* var. *sericeum*_LHM1265 | *psa*J | 41.257 | 0.631 | 0.996 | 0.409 | 0.31 | 0.386 | 0.385 |
| *Delphinium iliense*_LHM1285 | *psa*J | 41.257 | 0.631 | 0.996 | 0.409 | 0.31 | 0.386 | 0.385 |
| *Delphinium maackianum*_NC_047293 | *psa*J | 41.257 | 0.631 | 0.996 | 0.409 | 0.286 | 0.379 | 0.378 |
| *Delphinium mollifolium*_LHM1295 | *psa*J | 41.257 | 0.631 | 0.996 | 0.409 | 0.31 | 0.386 | 0.385 |
| *Delphinium naviculare* var. *lasiocarpum*_LHM1293 | *psa*J | 41.257 | 0.631 | 0.996 | 0.409 | 0.31 | 0.386 | 0.385 |
| *Delphinium sauricum*_LHM1266 | *psa*J | 42.257 | 0.661 | 1.06 | 0.409 | 0.333 | 0.394 | 0.393 |
| *Delphinium shawurense*_LHM1271 | *psa*J | 41.257 | 0.631 | 0.996 | 0.409 | 0.31 | 0.386 | 0.385 |
| *Delphinium winklerianum*_LHM1299 | *psa*J | 41.257 | 0.631 | 0.996 | 0.409 | 0.31 | 0.386 | 0.385 |
| *Delphinium yunnanense*_MW246156 | *psa*J | 41.257 | 0.631 | 0.996 | 0.409 | 0.286 | 0.379 | 0.378 |
| *Aconitum brachypodum*_MT584424 | *psb*A | 41.886 | 0.507 | 0.573 | 0.433 | 0.308 | 0.429 | 0.427 |
| *Aconitum delavayi*_OM289058 | *psb*A | 41.98 | 0.503 | 0.568 | 0.433 | 0.311 | 0.431 | 0.429 |
| *Delphinium aemulans*_LHM1280 | *psb*A | 41.897 | 0.51 | 0.593 | 0.433 | 0.311 | 0.43 | 0.428 |
| *Delphinium anthriscifolium*_MK253461 | *psb*A | 41.122 | 0.521 | 0.63 | 0.433 | 0.293 | 0.424 | 0.423 |
| *Delphinium brunonianum*_NC_051554 | *psb*A | 42.32 | 0.503 | 0.568 | 0.433 | 0.317 | 0.432 | 0.43 |
| *Delphinium candelabrum* var. *monanthum*_MW246165 | *psb*A | 41.977 | 0.514 | 0.59 | 0.433 | 0.311 | 0.43 | 0.428 |
| *Delphinium ceratophorum*_MK253460 | *psb*A | 42.138 | 0.507 | 0.583 | 0.433 | 0.314 | 0.431 | 0.429 |
| *Delphinium elatum* var. *sericeum*_LHM1265 | *psb*A | 41.897 | 0.51 | 0.593 | 0.433 | 0.311 | 0.43 | 0.428 |
| *Delphinium iliense*_LHM1285 | *psb*A | 42.099 | 0.507 | 0.584 | 0.433 | 0.311 | 0.43 | 0.428 |
| *Delphinium maackianum*_NC_047293 | *psb*A | 42.546 | 0.499 | 0.569 | 0.433 | 0.314 | 0.431 | 0.429 |
| *Delphinium mollifolium*_LHM1295 | *psb*A | 42.138 | 0.507 | 0.583 | 0.433 | 0.314 | 0.431 | 0.429 |
| *Delphinium naviculare* var. *lasiocarpum*_LHM1293 | *psb*A | 42.251 | 0.5 | 0.577 | 0.433 | 0.311 | 0.43 | 0.428 |
| *Delphinium sauricum*_LHM1266 | *psb*A | 42.138 | 0.507 | 0.583 | 0.433 | 0.314 | 0.431 | 0.429 |
| *Delphinium shawurense*_LHM1271 | *psb*A | 41.897 | 0.51 | 0.593 | 0.433 | 0.311 | 0.43 | 0.428 |
| *Delphinium winklerianum*_LHM1299 | *psb*A | 42.138 | 0.507 | 0.583 | 0.433 | 0.314 | 0.431 | 0.429 |
| *Delphinium yunnanense*_MW246156 | *psb*A | 41.977 | 0.514 | 0.59 | 0.433 | 0.311 | 0.43 | 0.428 |
| *Aconitum brachypodum*_MT584424 | *psb*B | 48.348 | 0.434 | 0.355 | 0.457 | 0.254 | 0.433 | 0.433 |
| *Aconitum delavayi*_OM289058 | *psb*B | 48.123 | 0.436 | 0.36 | 0.455 | 0.252 | 0.432 | 0.432 |
| *Delphinium aemulans*_LHM1280 | *psb*B | 48.456 | 0.442 | 0.362 | 0.457 | 0.254 | 0.432 | 0.432 |
| *Delphinium anthriscifolium*_MK253461 | *psb*B | 48.508 | 0.435 | 0.356 | 0.455 | 0.256 | 0.433 | 0.433 |
| *Delphinium brunonianum*_NC_051554 | *psb*B | 48.574 | 0.437 | 0.356 | 0.455 | 0.256 | 0.431 | 0.431 |
| *Delphinium candelabrum* var. *monanthum*_MW246165 | *psb*B | 48.477 | 0.439 | 0.361 | 0.455 | 0.254 | 0.432 | 0.432 |
| *Delphinium ceratophorum*_MK253460 | *psb*B | 48.472 | 0.438 | 0.36 | 0.455 | 0.254 | 0.431 | 0.431 |
| *Delphinium elatum* var. *sericeum*_LHM1265 | *psb*B | 48.477 | 0.439 | 0.361 | 0.455 | 0.254 | 0.432 | 0.432 |
| *Delphinium iliense*_LHM1285 | *psb*B | 48.477 | 0.439 | 0.361 | 0.455 | 0.252 | 0.431 | 0.431 |
| *Delphinium maackianum*_NC_047293 | *psb*B | 48.674 | 0.441 | 0.358 | 0.457 | 0.254 | 0.432 | 0.432 |
| *Delphinium mollifolium*_LHM1295 | *psb*B | 48.477 | 0.439 | 0.361 | 0.455 | 0.252 | 0.431 | 0.431 |
| *Delphinium naviculare* var. *lasiocarpum*_LHM1293 | *psb*B | 48.477 | 0.439 | 0.361 | 0.455 | 0.252 | 0.431 | 0.431 |
| *Delphinium sauricum*_LHM1266 | *psb*B | 48.477 | 0.439 | 0.361 | 0.455 | 0.252 | 0.431 | 0.431 |
| *Delphinium shawurense*_LHM1271 | *psb*B | 48.475 | 0.439 | 0.36 | 0.455 | 0.254 | 0.431 | 0.431 |
| *Delphinium winklerianum*_LHM1299 | *psb*B | 48.477 | 0.439 | 0.361 | 0.455 | 0.252 | 0.431 | 0.431 |
| *Delphinium yunnanense*_MW246156 | *psb*B | 48.477 | 0.439 | 0.361 | 0.455 | 0.254 | 0.432 | 0.432 |
| *Aconitum brachypodum*_MT584424 | *psb*C | 45.869 | 0.395 | 0.375 | 0.457 | 0.297 | 0.443 | 0.443 |
| *Aconitum delavayi*_OM289058 | *psb*C | 45.869 | 0.395 | 0.375 | 0.457 | 0.297 | 0.443 | 0.443 |
| *Delphinium aemulans*_LHM1280 | *psb*C | 47.276 | 0.37 | 0.34 | 0.457 | 0.313 | 0.449 | 0.449 |
| *Delphinium anthriscifolium*_MK253461 | *psb*C | 46.171 | 0.373 | 0.348 | 0.457 | 0.317 | 0.45 | 0.449 |
| *Delphinium brunonianum*_NC_051554 | *psb*C | 47.276 | 0.37 | 0.34 | 0.457 | 0.313 | 0.449 | 0.449 |
| *Delphinium candelabrum* var. *monanthum*_MW246165 | *psb*C | 47.276 | 0.37 | 0.34 | 0.457 | 0.313 | 0.449 | 0.449 |
| *Delphinium ceratophorum*_MK253460 | *psb*C | 47.276 | 0.37 | 0.34 | 0.457 | 0.313 | 0.449 | 0.449 |
| *Delphinium elatum* var. *sericeum*_LHM1265 | *psb*C | 47.276 | 0.37 | 0.34 | 0.457 | 0.313 | 0.449 | 0.449 |
| *Delphinium iliense*_LHM1285 | *psb*C | 47.276 | 0.37 | 0.34 | 0.457 | 0.313 | 0.449 | 0.449 |
| *Delphinium maackianum*_NC_047293 | *psb*C | 47.336 | 0.366 | 0.337 | 0.457 | 0.313 | 0.45 | 0.449 |
| *Delphinium mollifolium*_LHM1295 | *psb*C | 47.276 | 0.37 | 0.34 | 0.457 | 0.313 | 0.449 | 0.449 |
| *Delphinium naviculare* var. *lasiocarpum*_LHM1293 | *psb*C | 47.276 | 0.37 | 0.34 | 0.457 | 0.313 | 0.449 | 0.449 |
| *Delphinium sauricum*_LHM1266 | *psb*C | 47.276 | 0.37 | 0.34 | 0.457 | 0.313 | 0.449 | 0.449 |
| *Delphinium shawurense*_LHM1271 | *psb*C | 47.276 | 0.37 | 0.34 | 0.457 | 0.313 | 0.449 | 0.449 |
| *Delphinium winklerianum*_LHM1299 | *psb*C | 47.276 | 0.37 | 0.34 | 0.457 | 0.313 | 0.449 | 0.449 |
| *Delphinium yunnanense*_MW246156 | *psb*C | 47.276 | 0.37 | 0.34 | 0.457 | 0.313 | 0.449 | 0.449 |
| *Aconitum brachypodum*_MT584424 | *psb*D | 47.952 | 0.363 | 0.365 | 0.439 | 0.302 | 0.438 | 0.437 |
| *Aconitum delavayi*_OM289058 | *psb*D | 47.616 | 0.368 | 0.374 | 0.439 | 0.299 | 0.437 | 0.436 |
| *Delphinium aemulans*_LHM1280 | *psb*D | 46.901 | 0.378 | 0.383 | 0.439 | 0.296 | 0.436 | 0.435 |
| *Delphinium anthriscifolium*_MK253461 | *psb*D | 48.543 | 0.371 | 0.346 | 0.439 | 0.305 | 0.438 | 0.437 |
| *Delphinium brunonianum*_NC_051554 | *psb*D | 46.901 | 0.378 | 0.383 | 0.439 | 0.296 | 0.436 | 0.435 |
| *Delphinium candelabrum* var. *monanthum*_MW246165 | *psb*D | 46.901 | 0.378 | 0.383 | 0.439 | 0.296 | 0.436 | 0.435 |
| *Delphinium ceratophorum*_MK253460 | *psb*D | 46.962 | 0.377 | 0.379 | 0.439 | 0.299 | 0.437 | 0.436 |
| *Delphinium elatum* var. *sericeum*_LHM1265 | *psb*D | 46.901 | 0.378 | 0.383 | 0.439 | 0.296 | 0.436 | 0.435 |
| *Delphinium iliense*_LHM1285 | *psb*D | 46.826 | 0.384 | 0.387 | 0.439 | 0.296 | 0.435 | 0.434 |
| *Delphinium maackianum*_NC_047293 | *psb*D | 46.901 | 0.378 | 0.383 | 0.439 | 0.296 | 0.436 | 0.435 |
| *Delphinium mollifolium*_LHM1295 | *psb*D | 46.57 | 0.379 | 0.39 | 0.439 | 0.293 | 0.435 | 0.434 |
| *Delphinium naviculare* var. *lasiocarpum*_LHM1293 | *psb*D | 46.826 | 0.384 | 0.387 | 0.439 | 0.296 | 0.435 | 0.434 |
| *Delphinium sauricum*_LHM1266 | *psb*D | 46.548 | 0.383 | 0.391 | 0.439 | 0.29 | 0.434 | 0.433 |
| *Delphinium shawurense*_LHM1271 | *psb*D | 46.901 | 0.378 | 0.383 | 0.439 | 0.296 | 0.436 | 0.435 |
| *Delphinium winklerianum*_LHM1299 | *psb*D | 46.57 | 0.379 | 0.39 | 0.439 | 0.293 | 0.435 | 0.434 |
| *Delphinium yunnanense*_MW246156 | *psb*D | 46.901 | 0.378 | 0.383 | 0.439 | 0.296 | 0.436 | 0.435 |
| *Aconitum brachypodum*_MT584424 | *psb*E | 51.508 | 0.536 | 0.616 | 0.482 | 0.325 | 0.422 | 0.421 |
| *Aconitum delavayi*_OM289058 | *psb*E | 51.508 | 0.536 | 0.616 | 0.482 | 0.325 | 0.422 | 0.421 |
| *Delphinium aemulans*_LHM1280 | *psb*E | 47.777 | 0.523 | 0.666 | 0.482 | 0.3 | 0.414 | 0.413 |
| *Delphinium anthriscifolium*_MK253461 | *psb*E | 51.508 | 0.536 | 0.616 | 0.482 | 0.325 | 0.422 | 0.421 |
| *Delphinium brunonianum*_NC_051554 | *psb*E | 47.777 | 0.523 | 0.666 | 0.482 | 0.3 | 0.414 | 0.413 |
| *Delphinium candelabrum* var. *monanthum*_MW246165 | *psb*E | 47.777 | 0.523 | 0.666 | 0.482 | 0.3 | 0.414 | 0.413 |
| *Delphinium ceratophorum*_MK253460 | *psb*E | 47.777 | 0.523 | 0.666 | 0.482 | 0.288 | 0.41 | 0.409 |
| *Delphinium elatum* var. *sericeum*_LHM1265 | *psb*E | 47.777 | 0.523 | 0.666 | 0.482 | 0.3 | 0.414 | 0.413 |
| *Delphinium iliense*_LHM1285 | *psb*E | 46.622 | 0.548 | 0.691 | 0.482 | 0.313 | 0.418 | 0.417 |
| *Delphinium maackianum*_NC_047293 | *psb*E | 47.777 | 0.523 | 0.666 | 0.482 | 0.3 | 0.414 | 0.413 |
| *Delphinium mollifolium*_LHM1295 | *psb*E | 47.777 | 0.523 | 0.666 | 0.482 | 0.3 | 0.414 | 0.413 |
| *Delphinium naviculare* var. *lasiocarpum*_LHM1293 | *psb*E | 46.622 | 0.548 | 0.691 | 0.482 | 0.313 | 0.418 | 0.417 |
| *Delphinium sauricum*_LHM1266 | *psb*E | 47.777 | 0.523 | 0.666 | 0.482 | 0.3 | 0.414 | 0.413 |
| *Delphinium shawurense*_LHM1271 | *psb*E | 47.777 | 0.523 | 0.666 | 0.482 | 0.3 | 0.414 | 0.413 |
| *Delphinium winklerianum*_LHM1299 | *psb*E | 47.777 | 0.523 | 0.666 | 0.482 | 0.3 | 0.414 | 0.413 |
| *Delphinium yunnanense*_MW246156 | *psb*E | 47.777 | 0.523 | 0.666 | 0.482 | 0.3 | 0.414 | 0.413 |
| *Aconitum brachypodum*_MT584424 | *psb*F | 61 | 0.592 | 1.014 | 0.462 | 0.25 | 0.41 | 0.4 |
| *Aconitum delavayi*_OM289058 | *psb*F | 61 | 0.592 | 1.014 | 0.462 | 0.25 | 0.41 | 0.4 |
| *Delphinium aemulans*_LHM1280 | *psb*F | 61 | 0.571 | 0.903 | 0.462 | 0.222 | 0.402 | 0.392 |
| *Delphinium anthriscifolium*_MK253461 | *psb*F | 61 | 0.592 | 1.014 | 0.462 | 0.278 | 0.419 | 0.408 |
| *Delphinium brunonianum*_NC_051554 | *psb*F | 61 | 0.571 | 0.903 | 0.462 | 0.222 | 0.402 | 0.392 |
| *Delphinium candelabrum* var. *monanthum*_MW246165 | *psb*F | 61 | 0.624 | 0.986 | 0.462 | 0.194 | 0.393 | 0.383 |
| *Delphinium ceratophorum*_MK253460 | *psb*F | 61 | 0.571 | 0.903 | 0.462 | 0.222 | 0.402 | 0.392 |
| *Delphinium elatum* var. *sericeum*_LHM1265 | *psb*F | 61 | 0.571 | 0.903 | 0.462 | 0.222 | 0.402 | 0.392 |
| *Delphinium iliense*_LHM1285 | *psb*F | 61 | 0.571 | 0.903 | 0.462 | 0.222 | 0.402 | 0.392 |
| *Delphinium maackianum*_NC_047293 | *psb*F | 61 | 0.624 | 0.986 | 0.462 | 0.194 | 0.393 | 0.383 |
| *Delphinium mollifolium*_LHM1295 | *psb*F | 61 | 0.571 | 0.903 | 0.462 | 0.222 | 0.402 | 0.392 |
| *Delphinium naviculare* var. *lasiocarpum*_LHM1293 | *psb*F | 61 | 0.571 | 0.903 | 0.462 | 0.222 | 0.402 | 0.392 |
| *Delphinium sauricum*_LHM1266 | *psb*F | 61 | 0.571 | 0.903 | 0.462 | 0.222 | 0.402 | 0.392 |
| *Delphinium shawurense*_LHM1271 | *psb*F | 61 | 0.571 | 0.903 | 0.462 | 0.222 | 0.402 | 0.392 |
| *Delphinium winklerianum*_LHM1299 | *psb*F | 61 | 0.571 | 0.903 | 0.462 | 0.222 | 0.402 | 0.392 |
| *Delphinium yunnanense*_MW246156 | *psb*F | 61 | 0.624 | 0.986 | 0.462 | 0.194 | 0.393 | 0.383 |
| *Aconitum brachypodum*_MT584424 | *psb*H | 47.23 | 0.607 | 0.792 | 0.479 | 0.279 | 0.416 | 0.414 |
| *Aconitum delavayi*_OM289058 | *psb*H | 47.23 | 0.607 | 0.792 | 0.479 | 0.265 | 0.411 | 0.41 |
| *Delphinium aemulans*_LHM1280 | *psb*H | 44.009 | 0.628 | 0.839 | 0.479 | 0.25 | 0.406 | 0.405 |
| *Delphinium anthriscifolium*_MK253461 | *psb*H | 44.009 | 0.628 | 0.839 | 0.479 | 0.25 | 0.406 | 0.405 |
| *Delphinium brunonianum*_NC_051554 | *psb*H | 44.009 | 0.628 | 0.839 | 0.479 | 0.25 | 0.406 | 0.405 |
| *Delphinium candelabrum* var. *monanthum*_MW246165 | *psb*H | 44.009 | 0.628 | 0.839 | 0.479 | 0.25 | 0.406 | 0.405 |
| *Delphinium ceratophorum*_MK253460 | *psb*H | 44.009 | 0.628 | 0.839 | 0.479 | 0.25 | 0.406 | 0.405 |
| *Delphinium elatum* var. *sericeum*_LHM1265 | *psb*H | 44.009 | 0.628 | 0.839 | 0.479 | 0.25 | 0.406 | 0.405 |
| *Delphinium iliense*_LHM1285 | *psb*H | 44.009 | 0.628 | 0.839 | 0.479 | 0.25 | 0.406 | 0.405 |
| *Delphinium maackianum*_NC_047293 | *psb*H | 44.009 | 0.628 | 0.839 | 0.479 | 0.25 | 0.406 | 0.405 |
| *Delphinium mollifolium*_LHM1295 | *psb*H | 44.009 | 0.628 | 0.839 | 0.479 | 0.25 | 0.406 | 0.405 |
| *Delphinium naviculare* var. *lasiocarpum*_LHM1293 | *psb*H | 44.009 | 0.628 | 0.839 | 0.479 | 0.25 | 0.406 | 0.405 |
| *Delphinium sauricum*_LHM1266 | *psb*H | 44.009 | 0.628 | 0.839 | 0.479 | 0.25 | 0.406 | 0.405 |
| *Delphinium shawurense*_LHM1271 | *psb*H | 44.009 | 0.628 | 0.839 | 0.479 | 0.25 | 0.406 | 0.405 |
| *Delphinium winklerianum*_LHM1299 | *psb*H | 44.009 | 0.628 | 0.839 | 0.479 | 0.25 | 0.406 | 0.405 |
| *Delphinium yunnanense*_MW246156 | *psb*H | 45.205 | 0.624 | 0.812 | 0.466 | 0.25 | 0.402 | 0.401 |
| *Aconitum brachypodum*_MT584424 | *psb*I | 25.546 | 0.745 | 1.539 | 0.306 | 0.4 | 0.417 | 0.405 |
| *Aconitum delavayi*_OM289058 | *psb*I | 25.546 | 0.745 | 1.539 | 0.306 | 0.4 | 0.417 | 0.405 |
| *Delphinium aemulans*_LHM1280 | *psb*I | 24.824 | 0.802 | 1.596 | 0.306 | 0.343 | 0.398 | 0.387 |
| *Delphinium anthriscifolium*_MK253461 | *psb*I | 24.824 | 0.802 | 1.596 | 0.306 | 0.343 | 0.398 | 0.387 |
| *Delphinium brunonianum*_NC_051554 | *psb*I | 24.824 | 0.802 | 1.596 | 0.306 | 0.343 | 0.398 | 0.387 |
| *Delphinium candelabrum* var. *monanthum*_MW246165 | *psb*I | 24.824 | 0.802 | 1.596 | 0.306 | 0.343 | 0.398 | 0.387 |
| *Delphinium ceratophorum*_MK253460 | *psb*I | 24.824 | 0.802 | 1.596 | 0.306 | 0.343 | 0.398 | 0.387 |
| *Delphinium elatum* var. *sericeum*_LHM1265 | *psb*I | 24.824 | 0.802 | 1.596 | 0.306 | 0.343 | 0.398 | 0.387 |
| *Delphinium iliense*_LHM1285 | *psb*I | 24.824 | 0.802 | 1.596 | 0.306 | 0.343 | 0.398 | 0.387 |
| *Delphinium maackianum*_NC_047293 | *psb*I | 24.824 | 0.802 | 1.596 | 0.306 | 0.343 | 0.398 | 0.387 |
| *Delphinium mollifolium*_LHM1295 | *psb*I | 24.824 | 0.802 | 1.596 | 0.306 | 0.343 | 0.398 | 0.387 |
| *Delphinium naviculare* var. *lasiocarpum*_LHM1293 | *psb*I | 24.824 | 0.802 | 1.596 | 0.306 | 0.343 | 0.398 | 0.387 |
| *Delphinium sauricum*_LHM1266 | *psb*I | 24.824 | 0.802 | 1.596 | 0.306 | 0.343 | 0.398 | 0.387 |
| *Delphinium shawurense*_LHM1271 | *psb*I | 24.824 | 0.802 | 1.596 | 0.306 | 0.343 | 0.398 | 0.387 |
| *Delphinium winklerianum*_LHM1299 | *psb*I | 24.824 | 0.802 | 1.596 | 0.306 | 0.343 | 0.398 | 0.387 |
| *Delphinium yunnanense*_MW246156 | *psb*I | 24.824 | 0.802 | 1.596 | 0.306 | 0.343 | 0.398 | 0.387 |
| *Aconitum brachypodum*_MT584424 | *psb*J | 43.753 | 0.609 | 1.017 | 0.5 | 0.263 | 0.433 | 0.423 |
| *Aconitum delavayi*_OM289058 | *psb*J | 43.753 | 0.609 | 1.017 | 0.5 | 0.263 | 0.433 | 0.423 |
| *Delphinium aemulans*_LHM1280 | *psb*J | 43.753 | 0.609 | 1.017 | 0.5 | 0.263 | 0.433 | 0.431 |
| *Delphinium anthriscifolium*_MK253461 | *psb*J | 43.753 | 0.609 | 1.017 | 0.5 | 0.263 | 0.433 | 0.431 |
| *Delphinium brunonianum*_NC_051554 | *psb*J | 43.753 | 0.609 | 1.017 | 0.5 | 0.263 | 0.433 | 0.431 |
| *Delphinium candelabrum* var. *monanthum*_MW246165 | *psb*J | 43.753 | 0.609 | 1.017 | 0.5 | 0.263 | 0.433 | 0.431 |
| *Delphinium ceratophorum*_MK253460 | *psb*J | 43.753 | 0.609 | 1.017 | 0.5 | 0.263 | 0.433 | 0.431 |
| *Delphinium elatum* var. *sericeum*_LHM1265 | *psb*J | 43.753 | 0.609 | 1.017 | 0.5 | 0.263 | 0.433 | 0.431 |
| *Delphinium iliense*_LHM1285 | *psb*J | 43.753 | 0.609 | 1.017 | 0.5 | 0.263 | 0.433 | 0.431 |
| *Delphinium maackianum*_NC_047293 | *psb*J | 43.753 | 0.609 | 1.017 | 0.5 | 0.263 | 0.433 | 0.431 |
| *Delphinium mollifolium*_LHM1295 | *psb*J | 43.753 | 0.609 | 1.017 | 0.5 | 0.263 | 0.433 | 0.431 |
| *Delphinium naviculare* var. *lasiocarpum*_LHM1293 | *psb*J | 43.753 | 0.609 | 1.017 | 0.5 | 0.263 | 0.433 | 0.431 |
| *Delphinium sauricum*_LHM1266 | *psb*J | 43.753 | 0.609 | 1.017 | 0.5 | 0.263 | 0.433 | 0.431 |
| *Delphinium shawurense*_LHM1271 | *psb*J | 43.753 | 0.609 | 1.017 | 0.5 | 0.263 | 0.433 | 0.431 |
| *Delphinium winklerianum*_LHM1299 | *psb*J | 43.753 | 0.609 | 1.017 | 0.5 | 0.263 | 0.433 | 0.431 |
| *Delphinium yunnanense*_MW246156 | *psb*J | 43.753 | 0.609 | 1.017 | 0.5 | 0.263 | 0.433 | 0.431 |
| *Aconitum brachypodum*_MT584424 | *psb*K | 43.912 | 0.658 | 0.862 | 0.295 | 0.31 | 0.35 | 0.344 |
| *Aconitum delavayi*_OM289058 | *psb*K | 43.912 | 0.658 | 0.862 | 0.295 | 0.31 | 0.35 | 0.344 |
| *Delphinium aemulans*_LHM1280 | *psb*K | 43.912 | 0.658 | 0.862 | 0.295 | 0.293 | 0.344 | 0.344 |
| *Delphinium anthriscifolium*_MK253461 | *psb*K | 43.912 | 0.658 | 0.862 | 0.295 | 0.293 | 0.344 | 0.344 |
| *Delphinium brunonianum*_NC_051554 | *psb*K | 43.912 | 0.658 | 0.862 | 0.295 | 0.293 | 0.344 | 0.344 |
| *Delphinium candelabrum* var. *monanthum*_MW246165 | *psb*K | 43.912 | 0.658 | 0.862 | 0.295 | 0.293 | 0.344 | 0.344 |
| *Delphinium ceratophorum*_MK253460 | *psb*K | 43.912 | 0.658 | 0.862 | 0.295 | 0.293 | 0.344 | 0.344 |
| *Delphinium elatum* var. *sericeum*_LHM1265 | *psb*K | 43.912 | 0.658 | 0.862 | 0.295 | 0.293 | 0.344 | 0.344 |
| *Delphinium iliense*_LHM1285 | *psb*K | 45.477 | 0.653 | 0.839 | 0.295 | 0.276 | 0.339 | 0.339 |
| *Delphinium maackianum*_NC_047293 | *psb*K | 43.912 | 0.658 | 0.862 | 0.295 | 0.293 | 0.344 | 0.344 |
| *Delphinium mollifolium*_LHM1295 | *psb*K | 45.477 | 0.653 | 0.839 | 0.295 | 0.276 | 0.339 | 0.339 |
| *Delphinium naviculare* var. *lasiocarpum*_LHM1293 | *psb*K | 45.477 | 0.653 | 0.839 | 0.295 | 0.276 | 0.339 | 0.339 |
| *Delphinium sauricum*_LHM1266 | *psb*K | 45.477 | 0.653 | 0.839 | 0.295 | 0.276 | 0.339 | 0.339 |
| *Delphinium shawurense*_LHM1271 | *psb*K | 43.912 | 0.658 | 0.862 | 0.295 | 0.293 | 0.344 | 0.344 |
| *Delphinium winklerianum*_LHM1299 | *psb*K | 45.477 | 0.653 | 0.839 | 0.295 | 0.276 | 0.339 | 0.339 |
| *Delphinium yunnanense*_MW246156 | *psb*K | 43.912 | 0.658 | 0.862 | 0.295 | 0.293 | 0.344 | 0.344 |
| *Aconitum brachypodum*_MT584424 | *psb*L | 48.104 | 0.609 | 1.048 | 0.289 | 0.278 | 0.325 | 0.316 |
| *Aconitum delavayi*_OM289058 | *psb*L | 48.104 | 0.609 | 1.048 | 0.289 | 0.278 | 0.325 | 0.316 |
| *Delphinium aemulans*_LHM1280 | *psb*L | 48.104 | 0.609 | 1.048 | 0.289 | 0.278 | 0.325 | 0.316 |
| *Delphinium anthriscifolium*_MK253461 | *psb*L | 48.104 | 0.609 | 1.048 | 0.289 | 0.25 | 0.316 | 0.308 |
| *Delphinium brunonianum*_NC_051554 | *psb*L | 48.104 | 0.609 | 1.048 | 0.289 | 0.278 | 0.325 | 0.316 |
| *Delphinium candelabrum* var. *monanthum*_MW246165 | *psb*L | 48.104 | 0.609 | 1.048 | 0.289 | 0.278 | 0.325 | 0.316 |
| *Delphinium ceratophorum*_MK253460 | *psb*L | 48.104 | 0.609 | 1.048 | 0.289 | 0.278 | 0.325 | 0.316 |
| *Delphinium elatum* var. *sericeum*_LHM1265 | *psb*L | 48.104 | 0.609 | 1.048 | 0.289 | 0.278 | 0.325 | 0.316 |
| *Delphinium iliense*_LHM1285 | *psb*L | 48.104 | 0.609 | 1.048 | 0.289 | 0.278 | 0.325 | 0.316 |
| *Delphinium maackianum*_NC_047293 | *psb*L | 48.104 | 0.609 | 1.048 | 0.289 | 0.278 | 0.325 | 0.316 |
| *Delphinium mollifolium*_LHM1295 | *psb*L | 48.104 | 0.609 | 1.048 | 0.289 | 0.278 | 0.325 | 0.316 |
| *Delphinium naviculare* var. *lasiocarpum*_LHM1293 | *psb*L | 48.104 | 0.609 | 1.048 | 0.289 | 0.278 | 0.325 | 0.316 |
| *Delphinium sauricum*_LHM1266 | *psb*L | 48.104 | 0.609 | 1.048 | 0.289 | 0.278 | 0.325 | 0.316 |
| *Delphinium shawurense*_LHM1271 | *psb*L | 48.104 | 0.609 | 1.048 | 0.289 | 0.278 | 0.325 | 0.316 |
| *Delphinium winklerianum*_LHM1299 | *psb*L | 48.104 | 0.609 | 1.048 | 0.289 | 0.278 | 0.325 | 0.316 |
| *Delphinium yunnanense*_MW246156 | *psb*L | 48.104 | 0.609 | 1.048 | 0.289 | 0.278 | 0.325 | 0.316 |
| *Aconitum brachypodum*_MT584424 | *psb*M |  | 0.661 | 0.966 | 0.265 | 0.212 | 0.324 | 0.314 |
| *Aconitum delavayi*_OM289058 | *psb*M |  | 0.654 | 0.945 | 0.265 | 0.212 | 0.324 | 0.314 |
| *Delphinium aemulans*_LHM1280 | *psb*M |  | 0.634 | 0.885 | 0.265 | 0.242 | 0.333 | 0.324 |
| *Delphinium anthriscifolium*_MK253461 | *psb*M |  | 0.654 | 0.945 | 0.265 | 0.212 | 0.324 | 0.314 |
| *Delphinium brunonianum*_NC_051554 | *psb*M |  | 0.634 | 0.885 | 0.265 | 0.242 | 0.333 | 0.324 |
| *Delphinium candelabrum* var. *monanthum*_MW246165 | *psb*M |  | 0.634 | 0.885 | 0.265 | 0.273 | 0.343 | 0.333 |
| *Delphinium ceratophorum*_MK253460 | *psb*M |  | 0.634 | 0.885 | 0.265 | 0.242 | 0.333 | 0.324 |
| *Delphinium elatum* var. *sericeum*_LHM1265 | *psb*M |  | 0.634 | 0.885 | 0.265 | 0.242 | 0.333 | 0.324 |
| *Delphinium iliense*_LHM1285 | *psb*M |  | 0.634 | 0.885 | 0.265 | 0.242 | 0.333 | 0.324 |
| *Delphinium maackianum*_NC_047293 | *psb*M |  | 0.634 | 0.885 | 0.265 | 0.242 | 0.333 | 0.324 |
| *Delphinium mollifolium*_LHM1295 | *psb*M |  | 0.634 | 0.885 | 0.265 | 0.242 | 0.333 | 0.324 |
| *Delphinium naviculare* var. *lasiocarpum*_LHM1293 | *psb*M |  | 0.634 | 0.885 | 0.265 | 0.242 | 0.333 | 0.324 |
| *Delphinium sauricum*_LHM1266 | *psb*M |  | 0.573 | 0.824 | 0.265 | 0.273 | 0.343 | 0.333 |
| *Delphinium shawurense*_LHM1271 | *psb*M |  | 0.634 | 0.885 | 0.265 | 0.242 | 0.333 | 0.324 |
| *Delphinium winklerianum*_LHM1299 | *psb*M |  | 0.634 | 0.885 | 0.265 | 0.242 | 0.333 | 0.324 |
| *Delphinium yunnanense*_MW246156 | *psb*M |  | 0.634 | 0.885 | 0.265 | 0.273 | 0.343 | 0.333 |
| *Aconitum brachypodum*_MT584424 | *psb*N | 42.834 | 0.607 | 1.003 | 0.419 | 0.381 | 0.457 | 0.455 |
| *Aconitum delavayi*_OM289058 | *psb*N | 42.834 | 0.607 | 1.003 | 0.419 | 0.381 | 0.457 | 0.455 |
| *Delphinium aemulans*_LHM1280 | *psb*N | 42.834 | 0.607 | 1.003 | 0.419 | 0.357 | 0.45 | 0.447 |
| *Delphinium anthriscifolium*_MK253461 | *psb*N | 46.584 | 0.59 | 0.908 | 0.419 | 0.381 | 0.457 | 0.455 |
| *Delphinium brunonianum*_NC_051554 | *psb*N | 42.834 | 0.607 | 1.003 | 0.419 | 0.357 | 0.45 | 0.447 |
| *Delphinium candelabrum* var. *monanthum*_MW246165 | *psb*N | 42.834 | 0.607 | 1.003 | 0.419 | 0.357 | 0.45 | 0.447 |
| *Delphinium ceratophorum*_MK253460 | *psb*N | 42.834 | 0.607 | 1.003 | 0.419 | 0.357 | 0.45 | 0.447 |
| *Delphinium elatum* var. *sericeum*_LHM1265 | *psb*N | 42.834 | 0.607 | 1.003 | 0.419 | 0.357 | 0.45 | 0.447 |
| *Delphinium iliense*_LHM1285 | *psb*N | 42.834 | 0.607 | 1.003 | 0.419 | 0.357 | 0.45 | 0.447 |
| *Delphinium maackianum*_NC_047293 | *psb*N | 42.834 | 0.607 | 1.003 | 0.419 | 0.357 | 0.45 | 0.447 |
| *Delphinium mollifolium*_LHM1295 | *psb*N | 42.834 | 0.607 | 1.003 | 0.419 | 0.357 | 0.45 | 0.447 |
| *Delphinium naviculare* var. *lasiocarpum*_LHM1293 | *psb*N | 42.834 | 0.607 | 1.003 | 0.419 | 0.357 | 0.45 | 0.447 |
| *Delphinium sauricum*_LHM1266 | *psb*N | 42.834 | 0.607 | 1.003 | 0.419 | 0.357 | 0.45 | 0.447 |
| *Delphinium shawurense*_LHM1271 | *psb*N | 42.834 | 0.607 | 1.003 | 0.419 | 0.357 | 0.45 | 0.447 |
| *Delphinium winklerianum*_LHM1299 | *psb*N | 42.834 | 0.607 | 1.003 | 0.419 | 0.357 | 0.45 | 0.447 |
| *Delphinium yunnanense*_MW246156 | *psb*N | 42.834 | 0.607 | 1.003 | 0.419 | 0.357 | 0.45 | 0.447 |
| *Aconitum brachypodum*_MT584424 | *psb*T |  | 0.409 | 0.829 | 0.343 | 0.412 | 0.4 | 0.398 |
| *Aconitum delavayi*_OM289058 | *psb*T |  | 0.409 | 0.829 | 0.343 | 0.412 | 0.4 | 0.398 |
| *Delphinium aemulans*_LHM1280 | *psb*T |  | 0.501 | 0.967 | 0.343 | 0.324 | 0.371 | 0.37 |
| *Delphinium anthriscifolium*_MK253461 | *psb*T |  | 0.523 | 0.947 | 0.343 | 0.353 | 0.381 | 0.38 |
| *Delphinium brunonianum*_NC_051554 | *psb*T |  | 0.501 | 0.967 | 0.343 | 0.324 | 0.371 | 0.37 |
| *Delphinium candelabrum* var. *monanthum*_MW246165 | *psb*T |  | 0.501 | 0.967 | 0.343 | 0.324 | 0.371 | 0.37 |
| *Delphinium ceratophorum*_MK253460 | *psb*T |  | 0.501 | 0.967 | 0.343 | 0.324 | 0.371 | 0.37 |
| *Delphinium elatum* var. *sericeum*_LHM1265 | *psb*T |  | 0.501 | 0.967 | 0.343 | 0.324 | 0.371 | 0.37 |
| *Delphinium iliense*_LHM1285 | *psb*T |  | 0.446 | 0.908 | 0.343 | 0.353 | 0.381 | 0.38 |
| *Delphinium maackianum*_NC_047293 | *psb*T |  | 0.501 | 0.967 | 0.343 | 0.324 | 0.371 | 0.37 |
| *Delphinium mollifolium*_LHM1295 | *psb*T |  | 0.501 | 0.967 | 0.343 | 0.324 | 0.371 | 0.37 |
| *Delphinium naviculare* var. *lasiocarpum*_LHM1293 | *psb*T |  | 0.446 | 0.908 | 0.343 | 0.353 | 0.381 | 0.38 |
| *Delphinium sauricum*_LHM1266 | *psb*T |  | 0.501 | 0.967 | 0.343 | 0.324 | 0.371 | 0.37 |
| *Delphinium shawurense*_LHM1271 | *psb*T |  | 0.501 | 0.967 | 0.343 | 0.324 | 0.371 | 0.37 |
| *Delphinium winklerianum*_LHM1299 | *psb*T |  | 0.501 | 0.967 | 0.343 | 0.324 | 0.371 | 0.37 |
| *Delphinium yunnanense*_MW246156 | *psb*T |  | 0.501 | 0.967 | 0.343 | 0.324 | 0.371 | 0.361 |
| *Aconitum brachypodum*_MT584424 | *psb*Z | 43.847 | 0.662 | 0.919 | 0.419 | 0.186 | 0.339 | 0.339 |
| *Aconitum delavayi*_OM289058 | *psb*Z | 43.847 | 0.662 | 0.919 | 0.419 | 0.186 | 0.339 | 0.339 |
| *Delphinium aemulans*_LHM1280 | *psb*Z | 43.847 | 0.662 | 0.919 | 0.419 | 0.186 | 0.339 | 0.339 |
| *Delphinium anthriscifolium*_MK253461 | *psb*Z | 44.807 | 0.658 | 0.897 | 0.419 | 0.186 | 0.344 | 0.344 |
| *Delphinium brunonianum*_NC_051554 | *psb*Z | 43.847 | 0.662 | 0.919 | 0.419 | 0.186 | 0.339 | 0.339 |
| *Delphinium candelabrum* var. *monanthum*_MW246165 | *psb*Z | 43.847 | 0.662 | 0.919 | 0.419 | 0.186 | 0.339 | 0.339 |
| *Delphinium ceratophorum*_MK253460 | *psb*Z | 43.847 | 0.662 | 0.919 | 0.419 | 0.186 | 0.339 | 0.339 |
| *Delphinium elatum* var. *sericeum*_LHM1265 | *psb*Z | 43.847 | 0.662 | 0.919 | 0.419 | 0.186 | 0.339 | 0.339 |
| *Delphinium iliense*_LHM1285 | *psb*Z | 43.847 | 0.662 | 0.919 | 0.419 | 0.186 | 0.339 | 0.339 |
| *Delphinium maackianum*_NC_047293 | *psb*Z | 43.847 | 0.662 | 0.919 | 0.419 | 0.186 | 0.339 | 0.339 |
| *Delphinium mollifolium*_LHM1295 | *psb*Z | 43.847 | 0.662 | 0.919 | 0.419 | 0.186 | 0.339 | 0.339 |
| *Delphinium naviculare* var. *lasiocarpum*_LHM1293 | *psb*Z | 43.847 | 0.662 | 0.919 | 0.419 | 0.186 | 0.339 | 0.339 |
| *Delphinium sauricum*_LHM1266 | *psb*Z | 43.847 | 0.662 | 0.919 | 0.419 | 0.186 | 0.339 | 0.339 |
| *Delphinium shawurense*_LHM1271 | *psb*Z | 43.847 | 0.662 | 0.919 | 0.419 | 0.186 | 0.339 | 0.339 |
| *Delphinium winklerianum*_LHM1299 | *psb*Z | 43.847 | 0.662 | 0.919 | 0.419 | 0.186 | 0.339 | 0.339 |
| *Delphinium yunnanense*_MW246156 | *psb*Z | 43.847 | 0.662 | 0.919 | 0.419 | 0.186 | 0.339 | 0.339 |
| *Aconitum brachypodum*_MT584424 | *rbc*L | 50.986 | 0.367 | 0.297 | 0.429 | 0.295 | 0.446 | 0.445 |
| *Aconitum delavayi*_OM289058 | *rbc*L | 51.107 | 0.369 | 0.297 | 0.432 | 0.295 | 0.446 | 0.445 |
| *Delphinium aemulans*_LHM1280 | *rbc*L | 49.608 | 0.381 | 0.325 | 0.432 | 0.293 | 0.445 | 0.444 |
| *Delphinium anthriscifolium*_MK253461 | *rbc*L | 50.106 | 0.379 | 0.308 | 0.432 | 0.293 | 0.446 | 0.445 |
| *Delphinium brunonianum*_NC_051554 | *rbc*L | 49.593 | 0.38 | 0.326 | 0.434 | 0.295 | 0.446 | 0.445 |
| *Delphinium candelabrum* var. *monanthum*_MW246165 | *rbc*L | 49.458 | 0.384 | 0.327 | 0.432 | 0.29 | 0.445 | 0.444 |
| *Delphinium ceratophorum*_MK253460 | *rbc*L | 49.552 | 0.381 | 0.325 | 0.432 | 0.293 | 0.446 | 0.445 |
| *Delphinium elatum* var. *sericeum*_LHM1265 | *rbc*L | 49.552 | 0.381 | 0.325 | 0.432 | 0.293 | 0.446 | 0.445 |
| *Delphinium iliense*_LHM1285 | *rbc*L | 49.552 | 0.381 | 0.325 | 0.432 | 0.293 | 0.446 | 0.445 |
| *Delphinium maackianum*_NC_047293 | *rbc*L | 49.472 | 0.381 | 0.329 | 0.432 | 0.29 | 0.444 | 0.443 |
| *Delphinium mollifolium*_LHM1295 | *rbc*L | 49.552 | 0.381 | 0.325 | 0.432 | 0.293 | 0.446 | 0.445 |
| *Delphinium naviculare* var. *lasiocarpum*_LHM1293 | *rbc*L | 49.552 | 0.381 | 0.325 | 0.432 | 0.293 | 0.446 | 0.445 |
| *Delphinium sauricum*_LHM1266 | *rbc*L | 49.552 | 0.381 | 0.325 | 0.432 | 0.293 | 0.446 | 0.445 |
| *Delphinium shawurense*_LHM1271 | *rbc*L | 49.552 | 0.381 | 0.325 | 0.432 | 0.293 | 0.446 | 0.445 |
| *Delphinium winklerianum*_LHM1299 | *rbc*L | 49.552 | 0.381 | 0.325 | 0.432 | 0.293 | 0.446 | 0.445 |
| *Delphinium yunnanense*_MW246156 | *rbc*L | 49.458 | 0.384 | 0.327 | 0.432 | 0.29 | 0.445 | 0.444 |
| *Aconitum brachypodum*_MT584424 | *rpl*2 | 51.932 | 0.38 | 0.369 | 0.502 | 0.304 | 0.446 | 0.446 |
| *Aconitum delavayi*_OM289058 | *rpl*2 | 51.932 | 0.38 | 0.369 | 0.502 | 0.304 | 0.446 | 0.446 |
| *Delphinium aemulans*_LHM1280 | *rpl*2 | 53.239 | 0.37 | 0.349 | 0.498 | 0.308 | 0.446 | 0.446 |
| *Delphinium anthriscifolium*_MK253461 | *rpl*2 | 52.729 | 0.37 | 0.357 | 0.502 | 0.304 | 0.446 | 0.446 |
| *Delphinium brunonianum*_NC_051554 | *rpl*2 | 53.239 | 0.37 | 0.349 | 0.498 | 0.308 | 0.446 | 0.446 |
| *Delphinium candelabrum* var. *monanthum*_MW246165 | *rpl*2 | 53.239 | 0.37 | 0.349 | 0.498 | 0.308 | 0.446 | 0.446 |
| *Delphinium ceratophorum*_MK253460 | *rpl*2 | 53.239 | 0.37 | 0.349 | 0.498 | 0.308 | 0.446 | 0.446 |
| *Delphinium elatum* var. *sericeum*_LHM1265 | *rpl*2 | 53.239 | 0.37 | 0.349 | 0.498 | 0.308 | 0.446 | 0.446 |
| *Delphinium iliense*_LHM1285 | *rpl*2 | 53.239 | 0.37 | 0.349 | 0.498 | 0.308 | 0.446 | 0.446 |
| *Delphinium maackianum*_NC_047293 | *rpl*2 | 53.239 | 0.37 | 0.349 | 0.498 | 0.308 | 0.446 | 0.446 |
| *Delphinium mollifolium*_LHM1295 | *rpl*2 | 53.239 | 0.37 | 0.349 | 0.498 | 0.308 | 0.446 | 0.446 |
| *Delphinium naviculare* var. *lasiocarpum*_LHM1293 | *rpl*2 | 53.239 | 0.37 | 0.349 | 0.498 | 0.308 | 0.446 | 0.446 |
| *Delphinium sauricum*_LHM1266 | *rpl*2 | 53.291 | 0.367 | 0.348 | 0.498 | 0.313 | 0.448 | 0.447 |
| *Delphinium shawurense*_LHM1271 | *rpl*2 | 53.239 | 0.37 | 0.349 | 0.498 | 0.308 | 0.446 | 0.446 |
| *Delphinium winklerianum*_LHM1299 | *rpl*2 | 53.291 | 0.367 | 0.348 | 0.498 | 0.313 | 0.448 | 0.447 |
| *Delphinium yunnanense*_MW246156 | *rpl*2 | 53.239 | 0.37 | 0.349 | 0.498 | 0.308 | 0.446 | 0.446 |
| *Aconitum brachypodum*_MT584424 | *rpl*14 | 51.005 | 0.507 | 0.515 | 0.369 | 0.229 | 0.385 | 0.382 |
| *Aconitum delavayi*_OM289058 | *rpl*14 | 51.005 | 0.507 | 0.515 | 0.369 | 0.229 | 0.385 | 0.382 |
| *Delphinium aemulans*_LHM1280 | *rpl*14 | 50.143 | 0.512 | 0.534 | 0.369 | 0.22 | 0.383 | 0.379 |
| *Delphinium anthriscifolium*_MK253461 | *rpl*14 | 50.467 | 0.514 | 0.531 | 0.369 | 0.22 | 0.383 | 0.379 |
| *Delphinium brunonianum*_NC_051554 | *rpl*14 | 50.143 | 0.512 | 0.534 | 0.369 | 0.22 | 0.383 | 0.379 |
| *Delphinium candelabrum* var. *monanthum*_MW246165 | *rpl*14 | 50.143 | 0.512 | 0.534 | 0.369 | 0.22 | 0.383 | 0.379 |
| *Delphinium ceratophorum*_MK253460 | *rpl*14 | 50.143 | 0.512 | 0.534 | 0.369 | 0.22 | 0.383 | 0.379 |
| *Delphinium elatum* var. *sericeum*_LHM1265 | *rpl*14 | 50.143 | 0.512 | 0.534 | 0.369 | 0.22 | 0.383 | 0.379 |
| *Delphinium iliense*_LHM1285 | *rpl*14 | 50.143 | 0.512 | 0.534 | 0.369 | 0.22 | 0.383 | 0.379 |
| *Delphinium maackianum*_NC_047293 | *rpl*14 | 50.143 | 0.512 | 0.534 | 0.369 | 0.22 | 0.383 | 0.379 |
| *Delphinium mollifolium*_LHM1295 | *rpl*14 | 50.143 | 0.512 | 0.534 | 0.369 | 0.22 | 0.383 | 0.379 |
| *Delphinium naviculare* var. *lasiocarpum*_LHM1293 | *rpl*14 | 50.143 | 0.512 | 0.534 | 0.369 | 0.22 | 0.383 | 0.379 |
| *Delphinium sauricum*_LHM1266 | *rpl*14 | 50.143 | 0.512 | 0.534 | 0.369 | 0.22 | 0.383 | 0.379 |
| *Delphinium shawurense*_LHM1271 | *rpl*14 | 50.143 | 0.512 | 0.534 | 0.369 | 0.22 | 0.383 | 0.379 |
| *Delphinium winklerianum*_LHM1299 | *rpl*14 | 50.143 | 0.512 | 0.534 | 0.369 | 0.22 | 0.383 | 0.379 |
| *Delphinium yunnanense*_MW246156 | *rpl*14 | 50.143 | 0.512 | 0.534 | 0.369 | 0.22 | 0.383 | 0.379 |
| *Aconitum brachypodum*_MT584424 | *rpl*16 | 44.121 | 0.521 | 0.65 | 0.526 | 0.23 | 0.444 | 0.444 |
| *Aconitum delavayi*_OM289058 | *rpl*16 | 44.121 | 0.521 | 0.65 | 0.526 | 0.23 | 0.444 | 0.444 |
| *Delphinium aemulans*_LHM1280 | *rpl*16 | 42.068 | 0.524 | 0.644 | 0.533 | 0.248 | 0.452 | 0.451 |
| *Delphinium anthriscifolium*_MK253461 | *rpl*16 | 44.649 | 0.509 | 0.62 | 0.526 | 0.246 | 0.452 | 0.451 |
| *Delphinium brunonianum*_NC_051554 | *rpl*16 | 42.068 | 0.524 | 0.644 | 0.533 | 0.248 | 0.452 | 0.451 |
| *Delphinium candelabrum* var. *monanthum*_MW246165 | *rpl*16 | 42.068 | 0.524 | 0.644 | 0.533 | 0.248 | 0.452 | 0.451 |
| *Delphinium ceratophorum*_MK253460 | *rpl*16 | 42.068 | 0.524 | 0.644 | 0.533 | 0.248 | 0.452 | 0.451 |
| *Delphinium elatum* var. *sericeum*_LHM1265 | *rpl*16 | 42.068 | 0.524 | 0.644 | 0.533 | 0.248 | 0.452 | 0.451 |
| *Delphinium iliense*_LHM1285 | *rpl*16 | 42.068 | 0.524 | 0.644 | 0.533 | 0.248 | 0.452 | 0.451 |
| *Delphinium maackianum*_NC_047293 | *rpl*16 | 42.068 | 0.524 | 0.644 | 0.533 | 0.248 | 0.452 | 0.451 |
| *Delphinium mollifolium*_LHM1295 | *rpl*16 | 42.068 | 0.524 | 0.644 | 0.533 | 0.248 | 0.452 | 0.451 |
| *Delphinium naviculare* var. *lasiocarpum*_LHM1293 | *rpl*16 | 42.068 | 0.524 | 0.644 | 0.533 | 0.248 | 0.452 | 0.451 |
| *Delphinium sauricum*_LHM1266 | *rpl*16 | 42.068 | 0.524 | 0.644 | 0.533 | 0.248 | 0.452 | 0.451 |
| *Delphinium shawurense*_LHM1271 | *rpl*16 | 42.068 | 0.524 | 0.644 | 0.533 | 0.248 | 0.452 | 0.451 |
| *Delphinium winklerianum*_LHM1299 | *rpl*16 | 42.068 | 0.524 | 0.644 | 0.533 | 0.248 | 0.452 | 0.451 |
| *Delphinium yunnanense*_MW246156 | *rpl*16 | 41.353 | 0.542 | 0.672 | 0.533 | 0.24 | 0.447 | 0.446 |
| *Aconitum brachypodum*_MT584424 | *rpl*20 | 47.146 | 0.482 | 0.494 | 0.441 | 0.313 | 0.396 | 0.394 |
| *Aconitum delavayi*_OM289058 | *rpl*20 | 46.99 | 0.475 | 0.503 | 0.438 | 0.316 | 0.395 | 0.393 |
| *Delphinium aemulans*_LHM1280 | *rpl*20 | 44.75 | 0.488 | 0.535 | 0.479 | 0.27 | 0.379 | 0.376 |
| *Delphinium anthriscifolium*_MK253461 | *rpl*20 | 43.124 | 0.515 | 0.599 | 0.479 | 0.252 | 0.37 | 0.367 |
| *Delphinium brunonianum*_NC_051554 | *rpl*20 | 44.304 | 0.501 | 0.546 | 0.479 | 0.27 | 0.382 | 0.379 |
| *Delphinium candelabrum* var. *monanthum*_MW246165 | *rpl*20 | 46.418 | 0.484 | 0.508 | 0.479 | 0.277 | 0.382 | 0.379 |
| *Delphinium ceratophorum*_MK253460 | *rpl*20 | 44.406 | 0.507 | 0.55 | 0.479 | 0.261 | 0.376 | 0.373 |
| *Delphinium elatum* var. *sericeum*_LHM1265 | *rpl*20 | 44.75 | 0.488 | 0.535 | 0.479 | 0.27 | 0.379 | 0.376 |
| *Delphinium iliense*_LHM1285 | *rpl*20 | 44.75 | 0.488 | 0.535 | 0.479 | 0.27 | 0.379 | 0.376 |
| *Delphinium maackianum*_NC_047293 | *rpl*20 | 44.75 | 0.488 | 0.535 | 0.479 | 0.27 | 0.379 | 0.376 |
| *Delphinium mollifolium*_LHM1295 | *rpl*20 | 44.75 | 0.488 | 0.535 | 0.479 | 0.27 | 0.379 | 0.376 |
| *Delphinium naviculare* var. *lasiocarpum*_LHM1293 | *rpl*20 | 44.75 | 0.488 | 0.535 | 0.479 | 0.27 | 0.379 | 0.376 |
| *Delphinium sauricum*_LHM1266 | *rpl*20 | 44.75 | 0.488 | 0.535 | 0.479 | 0.27 | 0.379 | 0.376 |
| *Delphinium shawurense*_LHM1271 | *rpl*20 | 44.75 | 0.488 | 0.535 | 0.479 | 0.27 | 0.379 | 0.376 |
| *Delphinium winklerianum*_LHM1299 | *rpl*20 | 44.75 | 0.488 | 0.535 | 0.479 | 0.27 | 0.379 | 0.376 |
| *Delphinium yunnanense*_MW246156 | *rpl*20 | 46.418 | 0.484 | 0.508 | 0.479 | 0.277 | 0.382 | 0.379 |
| *Aconitum brachypodum*_MT584424 | *rpl*22 | 48.847 | 0.445 | 0.452 | 0.363 | 0.242 | 0.363 | 0.361 |
| *Aconitum delavayi*_OM289058 | *rpl*22 | 49.483 | 0.444 | 0.448 | 0.358 | 0.242 | 0.361 | 0.359 |
| *Delphinium aemulans*_LHM1280 | *rpl*22 | 45.741 | 0.466 | 0.49 | 0.374 | 0.236 | 0.361 | 0.359 |
| *Delphinium anthriscifolium*_MK253461 | *rpl*22 | 48.565 | 0.457 | 0.477 | 0.371 | 0.247 | 0.371 | 0.373 |
| *Delphinium brunonianum*_NC_051554 | *rpl*22 | 45.741 | 0.466 | 0.49 | 0.374 | 0.236 | 0.361 | 0.359 |
| *Delphinium candelabrum* var. *monanthum*_MW246165 | *rpl*22 | 45.741 | 0.466 | 0.49 | 0.374 | 0.236 | 0.361 | 0.359 |
| *Delphinium ceratophorum*_MK253460 | *rpl*22 | 45.741 | 0.466 | 0.49 | 0.374 | 0.236 | 0.361 | 0.359 |
| *Delphinium elatum* var. *sericeum*_LHM1265 | *rpl*22 | 45.741 | 0.466 | 0.49 | 0.374 | 0.236 | 0.361 | 0.359 |
| *Delphinium iliense*_LHM1285 | *rpl*22 | 45.741 | 0.466 | 0.49 | 0.374 | 0.236 | 0.361 | 0.359 |
| *Delphinium maackianum*_NC_047293 | *rpl*22 | 45.212 | 0.476 | 0.501 | 0.37 | 0.24 | 0.359 | 0.357 |
| *Delphinium mollifolium*_LHM1295 | *rpl*22 | 45.741 | 0.466 | 0.49 | 0.374 | 0.236 | 0.361 | 0.359 |
| *Delphinium naviculare* var. *lasiocarpum*_LHM1293 | *rpl*22 | 45.741 | 0.466 | 0.49 | 0.374 | 0.236 | 0.361 | 0.359 |
| *Delphinium sauricum*_LHM1266 | *rpl*22 | 45.741 | 0.466 | 0.49 | 0.374 | 0.236 | 0.361 | 0.359 |
| *Delphinium shawurense*_LHM1271 | *rpl*22 | 45.741 | 0.466 | 0.49 | 0.374 | 0.236 | 0.361 | 0.359 |
| *Delphinium winklerianum*_LHM1299 | *rpl*22 | 45.741 | 0.466 | 0.49 | 0.374 | 0.236 | 0.361 | 0.359 |
| *Delphinium yunnanense*_MW246156 | *rpl*22 | 44.961 | 0.472 | 0.498 | 0.363 | 0.236 | 0.358 | 0.356 |
| *Aconitum brachypodum*_MT584424 | *rpl*23 | 50.897 | 0.507 | 0.596 | 0.419 | 0.267 | 0.387 | 0.383 |
| *Aconitum delavayi*_OM289058 | *rpl*23 | 50.897 | 0.507 | 0.596 | 0.419 | 0.267 | 0.387 | 0.383 |
| *Delphinium aemulans*_LHM1280 | *rpl*23 | 50.897 | 0.507 | 0.596 | 0.419 | 0.267 | 0.387 | 0.383 |
| *Delphinium anthriscifolium*_MK253461 | *rpl*23 | 50.897 | 0.507 | 0.596 | 0.419 | 0.267 | 0.387 | 0.383 |
| *Delphinium brunonianum*_NC_051554 | *rpl*23 | 50.897 | 0.507 | 0.596 | 0.419 | 0.267 | 0.387 | 0.383 |
| *Delphinium candelabrum* var. *monanthum*_MW246165 | *rpl*23 | 50.897 | 0.507 | 0.596 | 0.419 | 0.267 | 0.387 | 0.383 |
| *Delphinium ceratophorum*_MK253460 | *rpl*23 | 50.897 | 0.507 | 0.596 | 0.419 | 0.267 | 0.387 | 0.383 |
| *Delphinium elatum* var. *sericeum*_LHM1265 | *rpl*23 | 50.897 | 0.507 | 0.596 | 0.419 | 0.267 | 0.387 | 0.383 |
| *Delphinium iliense*_LHM1285 | *rpl*23 | 50.897 | 0.507 | 0.596 | 0.419 | 0.267 | 0.387 | 0.383 |
| *Delphinium maackianum*_NC_047293 | *rpl*23 | 50.897 | 0.507 | 0.596 | 0.419 | 0.267 | 0.387 | 0.383 |
| *Delphinium mollifolium*_LHM1295 | *rpl*23 | 50.897 | 0.507 | 0.596 | 0.419 | 0.267 | 0.387 | 0.383 |
| *Delphinium naviculare* var. *lasiocarpum*_LHM1293 | *rpl*23 | 50.897 | 0.507 | 0.596 | 0.419 | 0.267 | 0.387 | 0.383 |
| *Delphinium sauricum*_LHM1266 | *rpl*23 | 50.897 | 0.507 | 0.596 | 0.419 | 0.267 | 0.387 | 0.383 |
| *Delphinium shawurense*_LHM1271 | *rpl*23 | 50.897 | 0.507 | 0.596 | 0.419 | 0.267 | 0.387 | 0.383 |
| *Delphinium winklerianum*_LHM1299 | *rpl*23 | 50.897 | 0.507 | 0.596 | 0.419 | 0.267 | 0.387 | 0.383 |
| *Delphinium yunnanense*_MW246156 | *rpl*23 | 50.897 | 0.507 | 0.596 | 0.419 | 0.267 | 0.387 | 0.383 |
| *Aconitum brachypodum*_MT584424 | *rpl*33 | 40.935 | 0.512 | 0.884 | 0.379 | 0.323 | 0.354 | 0.353 |
| *Aconitum delavayi*_OM289058 | *rpl*33 | 40.935 | 0.512 | 0.884 | 0.379 | 0.323 | 0.354 | 0.353 |
| *Delphinium aemulans*_LHM1280 | *rpl*33 | 40.935 | 0.531 | 0.925 | 0.379 | 0.338 | 0.359 | 0.358 |
| *Delphinium anthriscifolium*_MK253461 | *rpl*33 | 40.125 | 0.56 | 0.939 | 0.364 | 0.344 | 0.359 | 0.358 |
| *Delphinium brunonianum*_NC_051554 | *rpl*33 | 41.102 | 0.524 | 0.912 | 0.379 | 0.344 | 0.364 | 0.363 |
| *Delphinium candelabrum* var. *monanthum*_MW246165 | *rpl*33 | 40.935 | 0.531 | 0.925 | 0.379 | 0.338 | 0.359 | 0.358 |
| *Delphinium ceratophorum*_MK253460 | *rpl*33 | 40.935 | 0.531 | 0.925 | 0.379 | 0.338 | 0.354 | 0.353 |
| *Delphinium elatum* var. *sericeum*_LHM1265 | *rpl*33 | 40.935 | 0.531 | 0.925 | 0.379 | 0.338 | 0.359 | 0.358 |
| *Delphinium iliense*_LHM1285 | *rpl*33 | 40.935 | 0.531 | 0.925 | 0.379 | 0.338 | 0.359 | 0.358 |
| *Delphinium maackianum*_NC_047293 | *rpl*33 | 40.935 | 0.531 | 0.925 | 0.379 | 0.338 | 0.359 | 0.358 |
| *Delphinium mollifolium*_LHM1295 | *rpl*33 | 40.935 | 0.531 | 0.925 | 0.379 | 0.338 | 0.359 | 0.358 |
| *Delphinium naviculare* var. *lasiocarpum*_LHM1293 | *rpl*33 | 40.935 | 0.531 | 0.925 | 0.379 | 0.338 | 0.359 | 0.358 |
| *Delphinium sauricum*_LHM1266 | *rpl*33 | 40.935 | 0.531 | 0.925 | 0.379 | 0.338 | 0.359 | 0.358 |
| *Delphinium shawurense*_LHM1271 | *rpl*33 | 40.935 | 0.531 | 0.925 | 0.379 | 0.338 | 0.359 | 0.358 |
| *Delphinium winklerianum*_LHM1299 | *rpl*33 | 40.935 | 0.531 | 0.925 | 0.379 | 0.338 | 0.359 | 0.358 |
| *Delphinium yunnanense*_MW246156 | *rpl*33 | 40.935 | 0.531 | 0.925 | 0.379 | 0.338 | 0.359 | 0.358 |
| *Aconitum brachypodum*_MT584424 | *rpl*36 | 25.15 | 0.706 | 1.231 | 0.486 | 0.361 | 0.432 | 0.421 |
| *Aconitum delavayi*_OM289058 | *rpl*36 | 25.15 | 0.706 | 1.231 | 0.486 | 0.361 | 0.432 | 0.421 |
| *Delphinium aemulans*_LHM1280 | *rpl*36 |  | 0.687 | 1.12 | 0.486 | 0.389 | 0.441 | 0.43 |
| *Delphinium anthriscifolium*_MK253461 | *rpl*36 | 25.288 | 0.649 | 1.194 | 0.486 | 0.333 | 0.423 | 0.412 |
| *Delphinium brunonianum*_NC_051554 | *rpl*36 |  | 0.687 | 1.12 | 0.486 | 0.389 | 0.441 | 0.43 |
| *Delphinium candelabrum* var. *monanthum*_MW246165 | *rpl*36 |  | 0.687 | 1.12 | 0.486 | 0.389 | 0.441 | 0.43 |
| *Delphinium ceratophorum*_MK253460 | *rpl*36 |  | 0.687 | 1.12 | 0.486 | 0.389 | 0.441 | 0.43 |
| *Delphinium elatum* var. *sericeum*_LHM1265 | *rpl*36 |  | 0.687 | 1.12 | 0.486 | 0.389 | 0.441 | 0.43 |
| *Delphinium iliense*_LHM1285 | *rpl*36 |  | 0.687 | 1.12 | 0.486 | 0.389 | 0.441 | 0.43 |
| *Delphinium maackianum*_NC_047293 | *rpl*36 |  | 0.651 | 1.083 | 0.514 | 0.389 | 0.45 | 0.439 |
| *Delphinium mollifolium*_LHM1295 | *rpl*36 |  | 0.687 | 1.12 | 0.486 | 0.389 | 0.441 | 0.43 |
| *Delphinium naviculare* var. *lasiocarpum*_LHM1293 | *rpl*36 |  | 0.687 | 1.12 | 0.486 | 0.389 | 0.441 | 0.43 |
| *Delphinium sauricum*_LHM1266 | *rpl*36 |  | 0.687 | 1.12 | 0.486 | 0.389 | 0.441 | 0.43 |
| *Delphinium shawurense*_LHM1271 | *rpl*36 |  | 0.687 | 1.12 | 0.486 | 0.389 | 0.441 | 0.43 |
| *Delphinium winklerianum*_LHM1299 | *rpl*36 |  | 0.687 | 1.12 | 0.486 | 0.389 | 0.441 | 0.43 |
| *Delphinium yunnanense*_MW246156 | *rpl*36 |  | 0.687 | 1.12 | 0.486 | 0.389 | 0.441 | 0.43 |
| *Aconitum brachypodum*_MT584424 | *rpo*A | 48.521 | 0.439 | 0.374 | 0.329 | 0.247 | 0.353 | 0.353 |
| *Aconitum delavayi*_OM289058 | *rpo*A | 48.494 | 0.447 | 0.381 | 0.323 | 0.244 | 0.35 | 0.35 |
| *Delphinium aemulans*_LHM1280 | *rpo*A | 48.522 | 0.433 | 0.365 | 0.344 | 0.251 | 0.361 | 0.361 |
| *Delphinium anthriscifolium*_MK253461 | *rpo*A | 48.903 | 0.466 | 0.392 | 0.338 | 0.242 | 0.353 | 0.353 |
| *Delphinium brunonianum*_NC_051554 | *rpo*A | 48.553 | 0.432 | 0.364 | 0.344 | 0.253 | 0.362 | 0.362 |
| *Delphinium candelabrum* var. *monanthum*_MW246165 | *rpo*A | 48.949 | 0.425 | 0.355 | 0.347 | 0.259 | 0.366 | 0.366 |
| *Delphinium ceratophorum*_MK253460 | *rpo*A | 48.727 | 0.427 | 0.36 | 0.344 | 0.258 | 0.365 | 0.365 |
| *Delphinium elatum* var. *sericeum*_LHM1265 | *rpo*A | 48.522 | 0.433 | 0.365 | 0.344 | 0.251 | 0.361 | 0.361 |
| *Delphinium iliense*_LHM1285 | *rpo*A | 48.616 | 0.431 | 0.362 | 0.344 | 0.254 | 0.362 | 0.362 |
| *Delphinium maackianum*_NC_047293 | *rpo*A | 48.668 | 0.428 | 0.365 | 0.344 | 0.252 | 0.362 | 0.362 |
| *Delphinium mollifolium*_LHM1295 | *rpo*A | 48.616 | 0.431 | 0.362 | 0.344 | 0.254 | 0.362 | 0.362 |
| *Delphinium naviculare* var. *lasiocarpum*_LHM1293 | *rpo*A | 48.616 | 0.431 | 0.362 | 0.344 | 0.254 | 0.362 | 0.362 |
| *Delphinium sauricum*_LHM1266 | *rpo*A | 48.616 | 0.431 | 0.362 | 0.344 | 0.254 | 0.362 | 0.362 |
| *Delphinium shawurense*_LHM1271 | *rpo*A | 48.582 | 0.433 | 0.364 | 0.344 | 0.251 | 0.362 | 0.362 |
| *Delphinium winklerianum*_LHM1299 | *rpo*A | 48.729 | 0.427 | 0.357 | 0.344 | 0.257 | 0.363 | 0.363 |
| *Delphinium yunnanense*_MW246156 | *rpo*A | 48.949 | 0.425 | 0.355 | 0.347 | 0.259 | 0.366 | 0.366 |
| *Aconitum brachypodum*_MT584424 | *rpo*B | 49.633 | 0.398 | 0.291 | 0.386 | 0.27 | 0.396 | 0.396 |
| *Aconitum delavayi*_OM289058 | *rpo*B | 49.494 | 0.398 | 0.292 | 0.387 | 0.271 | 0.396 | 0.396 |
| *Delphinium aemulans*_LHM1280 | *rpo*B | 49.789 | 0.398 | 0.287 | 0.386 | 0.27 | 0.394 | 0.394 |
| *Delphinium anthriscifolium*_MK253461 | *rpo*B | 49.952 | 0.398 | 0.286 | 0.388 | 0.271 | 0.396 | 0.396 |
| *Delphinium brunonianum*_NC_051554 | *rpo*B | 49.528 | 0.401 | 0.291 | 0.385 | 0.267 | 0.393 | 0.393 |
| *Delphinium candelabrum* var. *monanthum*_MW246165 | *rpo*B | 49.778 | 0.399 | 0.287 | 0.385 | 0.269 | 0.394 | 0.394 |
| *Delphinium ceratophorum*_MK253460 | *rpo*B | 49.907 | 0.394 | 0.284 | 0.385 | 0.27 | 0.394 | 0.394 |
| *Delphinium elatum* var. *sericeum*_LHM1265 | *rpo*B | 49.735 | 0.398 | 0.288 | 0.386 | 0.269 | 0.394 | 0.394 |
| *Delphinium iliense*_LHM1285 | *rpo*B | 49.718 | 0.401 | 0.29 | 0.386 | 0.268 | 0.394 | 0.394 |
| *Delphinium maackianum*_NC_047293 | *rpo*B | 49.851 | 0.395 | 0.284 | 0.386 | 0.272 | 0.395 | 0.395 |
| *Delphinium mollifolium*_LHM1295 | *rpo*B | 49.667 | 0.403 | 0.292 | 0.386 | 0.267 | 0.394 | 0.394 |
| *Delphinium naviculare* var. *lasiocarpum*_LHM1293 | *rpo*B | 49.718 | 0.401 | 0.29 | 0.386 | 0.268 | 0.394 | 0.394 |
| *Delphinium sauricum*_LHM1266 | *rpo*B | 49.718 | 0.401 | 0.29 | 0.386 | 0.268 | 0.394 | 0.394 |
| *Delphinium shawurense*_LHM1271 | *rpo*B | 49.802 | 0.398 | 0.288 | 0.385 | 0.269 | 0.394 | 0.394 |
| *Delphinium winklerianum*_LHM1299 | *rpo*B | 49.718 | 0.401 | 0.29 | 0.386 | 0.268 | 0.394 | 0.394 |
| *Delphinium yunnanense*_MW246156 | *rpo*B | 49.634 | 0.403 | 0.292 | 0.386 | 0.267 | 0.393 | 0.393 |
| *Aconitum brachypodum*_MT584424 | *rpo*C1 | 49.1 | 0.42 | 0.32 | 0.38 | 0.249 | 0.389 | 0.388 |
| *Aconitum delavayi*_OM289058 | *rpo*C1 | 48.958 | 0.425 | 0.325 | 0.378 | 0.246 | 0.386 | 0.387 |
| *Delphinium aemulans*_LHM1280 | *rpo*C1 | 49.64 | 0.414 | 0.309 | 0.375 | 0.252 | 0.388 | 0.389 |
| *Delphinium anthriscifolium*_MK253461 | *rpo*C1 | 49.809 | 0.414 | 0.311 | 0.382 | 0.255 | 0.392 | 0.393 |
| *Delphinium brunonianum*_NC_051554 | *rpo*C1 | 49.647 | 0.417 | 0.315 | 0.375 | 0.252 | 0.388 | 0.389 |
| *Delphinium candelabrum* var. *monanthum*_MW246165 | *rpo*C1 | 49.483 | 0.419 | 0.319 | 0.375 | 0.25 | 0.388 | 0.389 |
| *Delphinium ceratophorum*_MK253460 | *rpo*C1 | 49.444 | 0.419 | 0.317 | 0.37 | 0.252 | 0.388 | 0.388 |
| *Delphinium elatum* var. *sericeum*_LHM1265 | *rpo*C1 | 49.752 | 0.411 | 0.307 | 0.375 | 0.252 | 0.388 | 0.389 |
| *Delphinium iliense*_LHM1285 | *rpo*C1 | 49.908 | 0.41 | 0.306 | 0.374 | 0.253 | 0.388 | 0.389 |
| *Delphinium maackianum*_NC_047293 | *rpo*C1 | 49.632 | 0.416 | 0.312 | 0.376 | 0.253 | 0.39 | 0.391 |
| *Delphinium mollifolium*_LHM1295 | *rpo*C1 | 49.908 | 0.41 | 0.306 | 0.374 | 0.253 | 0.388 | 0.389 |
| *Delphinium naviculare* var. *lasiocarpum*_LHM1293 | *rpo*C1 | 49.908 | 0.41 | 0.306 | 0.374 | 0.253 | 0.388 | 0.389 |
| *Delphinium sauricum*_LHM1266 | *rpo*C1 | 49.889 | 0.409 | 0.307 | 0.374 | 0.253 | 0.388 | 0.389 |
| *Delphinium shawurense*_LHM1271 | *rpo*C1 | 49.752 | 0.411 | 0.307 | 0.375 | 0.252 | 0.388 | 0.389 |
| *Delphinium winklerianum*_LHM1299 | *rpo*C1 | 49.889 | 0.409 | 0.307 | 0.374 | 0.253 | 0.388 | 0.389 |
| *Delphinium yunnanense*_MW246156 | *rpo*C1 | 49.406 | 0.419 | 0.32 | 0.376 | 0.25 | 0.388 | 0.389 |
| *Aconitum brachypodum*_MT584424 | *rpo*C2 | 50.214 | 0.382 | 0.266 | 0.381 | 0.273 | 0.379 | 0.378 |
| *Aconitum delavayi*_OM289058 | *rpo*C2 | 50.35 | 0.378 | 0.262 | 0.381 | 0.274 | 0.379 | 0.378 |
| *Delphinium aemulans*_LHM1280 | *rpo*C2 | 50.548 | 0.379 | 0.266 | 0.379 | 0.272 | 0.378 | 0.378 |
| *Delphinium anthriscifolium*_MK253461 | *rpo*C2 | 51.006 | 0.356 | 0.247 | 0.384 | 0.281 | 0.38 | 0.379 |
| *Delphinium brunonianum*_NC_051554 | *rpo*C2 | 50.53 | 0.383 | 0.268 | 0.382 | 0.273 | 0.379 | 0.379 |
| *Delphinium candelabrum* var. *monanthum*_MW246165 | *rpo*C2 | 50.594 | 0.379 | 0.266 | 0.38 | 0.273 | 0.379 | 0.378 |
| *Delphinium ceratophorum*_MK253460 | *rpo*C2 | 50.534 | 0.381 | 0.266 | 0.38 | 0.274 | 0.379 | 0.379 |
| *Delphinium elatum* var. *sericeum*_LHM1265 | *rpo*C2 | 50.556 | 0.38 | 0.266 | 0.38 | 0.273 | 0.379 | 0.378 |
| *Delphinium iliense*_LHM1285 | *rpo*C2 | 50.549 | 0.382 | 0.268 | 0.38 | 0.273 | 0.379 | 0.378 |
| *Delphinium maackianum*_NC_047293 | *rpo*C2 | 50.626 | 0.38 | 0.266 | 0.379 | 0.273 | 0.378 | 0.378 |
| *Delphinium mollifolium*_LHM1295 | *rpo*C2 | 50.557 | 0.381 | 0.267 | 0.381 | 0.273 | 0.379 | 0.378 |
| *Delphinium naviculare* var. *lasiocarpum*_LHM1293 | *rpo*C2 | 50.549 | 0.382 | 0.268 | 0.38 | 0.273 | 0.379 | 0.378 |
| *Delphinium sauricum*_LHM1266 | *rpo*C2 | 50.605 | 0.381 | 0.266 | 0.382 | 0.273 | 0.379 | 0.378 |
| *Delphinium shawurense*_LHM1271 | *rpo*C2 | 50.558 | 0.379 | 0.266 | 0.38 | 0.273 | 0.379 | 0.378 |
| *Delphinium winklerianum*_LHM1299 | *rpo*C2 | 50.566 | 0.382 | 0.267 | 0.381 | 0.273 | 0.379 | 0.378 |
| *Delphinium yunnanense*_MW246156 | *rpo*C2 | 50.621 | 0.379 | 0.264 | 0.38 | 0.274 | 0.379 | 0.378 |
| *Aconitum brachypodum*_MT584424 | *rps*2 | 45.463 | 0.504 | 0.508 | 0.438 | 0.219 | 0.379 | 0.38 |
| *Aconitum delavayi*_OM289058 | *rps*2 | 45.463 | 0.504 | 0.508 | 0.438 | 0.219 | 0.379 | 0.38 |
| *Delphinium aemulans*_LHM1280 | *rps*2 | 48.288 | 0.467 | 0.446 | 0.437 | 0.238 | 0.382 | 0.382 |
| *Delphinium anthriscifolium*_MK253461 | *rps*2 | 47.577 | 0.492 | 0.464 | 0.438 | 0.238 | 0.383 | 0.384 |
| *Delphinium brunonianum*_NC_051554 | *rps*2 | 47.842 | 0.47 | 0.456 | 0.437 | 0.233 | 0.381 | 0.381 |
| *Delphinium candelabrum* var. *monanthum*_MW246165 | *rps*2 | 47.199 | 0.475 | 0.466 | 0.434 | 0.232 | 0.38 | 0.381 |
| *Delphinium ceratophorum*_MK253460 | *rps*2 | 47.838 | 0.47 | 0.453 | 0.437 | 0.233 | 0.38 | 0.379 |
| *Delphinium elatum* var. *sericeum*_LHM1265 | *rps*2 | 48.288 | 0.467 | 0.446 | 0.437 | 0.238 | 0.382 | 0.382 |
| *Delphinium iliense*_LHM1285 | *rps*2 | 48.288 | 0.467 | 0.446 | 0.437 | 0.238 | 0.382 | 0.382 |
| *Delphinium maackianum*_NC_047293 | *rps*2 | 47.842 | 0.47 | 0.456 | 0.437 | 0.233 | 0.381 | 0.381 |
| *Delphinium mollifolium*_LHM1295 | *rps*2 | 48.288 | 0.467 | 0.446 | 0.437 | 0.238 | 0.382 | 0.382 |
| *Delphinium naviculare* var. *lasiocarpum*_LHM1293 | *rps*2 | 48.288 | 0.467 | 0.446 | 0.437 | 0.238 | 0.382 | 0.382 |
| *Delphinium sauricum*_LHM1266 | *rps*2 | 48.18 | 0.473 | 0.451 | 0.441 | 0.238 | 0.384 | 0.384 |
| *Delphinium shawurense*_LHM1271 | *rps*2 | 48.288 | 0.467 | 0.446 | 0.437 | 0.238 | 0.382 | 0.382 |
| *Delphinium winklerianum*_LHM1299 | *rps*2 | 48.288 | 0.467 | 0.446 | 0.437 | 0.238 | 0.382 | 0.382 |
| *Delphinium yunnanense*_MW246156 | *rps*2 | 47.199 | 0.475 | 0.466 | 0.434 | 0.232 | 0.38 | 0.381 |
| *Aconitum brachypodum*_MT584424 | *rps*3 | 47.917 | 0.491 | 0.461 | 0.355 | 0.217 | 0.352 | 0.35 |
| *Aconitum delavayi*_OM289058 | *rps*3 | 48.988 | 0.479 | 0.452 | 0.35 | 0.222 | 0.352 | 0.35 |
| *Delphinium aemulans*_LHM1280 | *rps*3 | 47.401 | 0.496 | 0.461 | 0.35 | 0.224 | 0.356 | 0.355 |
| *Delphinium anthriscifolium*_MK253461 | *rps*3 | 46.381 | 0.505 | 0.486 | 0.35 | 0.214 | 0.353 | 0.352 |
| *Delphinium brunonianum*_NC_051554 | *rps*3 | 49.043 | 0.49 | 0.433 | 0.35 | 0.229 | 0.358 | 0.356 |
| *Delphinium candelabrum* var. *monanthum*_MW246165 | *rps*3 | 48.107 | 0.496 | 0.449 | 0.35 | 0.229 | 0.358 | 0.356 |
| *Delphinium ceratophorum*_MK253460 | *rps*3 | 48.499 | 0.485 | 0.437 | 0.35 | 0.233 | 0.361 | 0.359 |
| *Delphinium elatum* var. *sericeum*_LHM1265 | *rps*3 | 47.545 | 0.495 | 0.458 | 0.35 | 0.229 | 0.358 | 0.356 |
| *Delphinium iliense*_LHM1285 | *rps*3 | 47.401 | 0.496 | 0.461 | 0.35 | 0.224 | 0.356 | 0.355 |
| *Delphinium maackianum*_NC_047293 | *rps*3 | 47.949 | 0.496 | 0.447 | 0.35 | 0.231 | 0.356 | 0.354 |
| *Delphinium mollifolium*_LHM1295 | *rps*3 | 47.401 | 0.496 | 0.461 | 0.35 | 0.224 | 0.356 | 0.355 |
| *Delphinium naviculare* var. *lasiocarpum*_LHM1293 | *rps*3 | 47.401 | 0.496 | 0.461 | 0.35 | 0.224 | 0.356 | 0.355 |
| *Delphinium sauricum*_LHM1266 | *rps*3 | 47.401 | 0.496 | 0.461 | 0.35 | 0.224 | 0.356 | 0.355 |
| *Delphinium shawurense*_LHM1271 | *rps*3 | 47.401 | 0.496 | 0.461 | 0.35 | 0.224 | 0.356 | 0.355 |
| *Delphinium winklerianum*_LHM1299 | *rps*3 | 47.401 | 0.496 | 0.461 | 0.35 | 0.224 | 0.356 | 0.355 |
| *Delphinium yunnanense*_MW246156 | *rps*3 | 48.577 | 0.487 | 0.435 | 0.35 | 0.233 | 0.359 | 0.358 |
| *Aconitum brachypodum*_MT584424 | *rps*4 | 51.863 | 0.44 | 0.396 | 0.388 | 0.274 | 0.395 | 0.394 |
| *Aconitum delavayi*_OM289058 | *rps*4 | 52.598 | 0.438 | 0.39 | 0.388 | 0.269 | 0.393 | 0.393 |
| *Delphinium aemulans*_LHM1280 | *rps*4 | 52.64 | 0.449 | 0.393 | 0.383 | 0.269 | 0.391 | 0.391 |
| *Delphinium anthriscifolium*_MK253461 | *rps*4 | 49.769 | 0.489 | 0.448 | 0.383 | 0.249 | 0.385 | 0.384 |
| *Delphinium brunonianum*_NC_051554 | *rps*4 | 52.353 | 0.451 | 0.397 | 0.383 | 0.274 | 0.393 | 0.393 |
| *Delphinium candelabrum* var. *monanthum*_MW246165 | *rps*4 | 52.564 | 0.444 | 0.392 | 0.388 | 0.274 | 0.395 | 0.394 |
| *Delphinium ceratophorum*_MK253460 | *rps*4 | 52.353 | 0.451 | 0.397 | 0.383 | 0.274 | 0.393 | 0.393 |
| *Delphinium elatum* var. *sericeum*_LHM1265 | *rps*4 | 52.64 | 0.449 | 0.393 | 0.383 | 0.269 | 0.391 | 0.391 |
| *Delphinium iliense*_LHM1285 | *rps*4 | 52.667 | 0.445 | 0.391 | 0.383 | 0.279 | 0.395 | 0.394 |
| *Delphinium maackianum*_NC_047293 | *rps*4 | 52.664 | 0.449 | 0.393 | 0.383 | 0.274 | 0.395 | 0.394 |
| *Delphinium mollifolium*_LHM1295 | *rps*4 | 52.353 | 0.451 | 0.397 | 0.383 | 0.274 | 0.393 | 0.393 |
| *Delphinium naviculare* var. *lasiocarpum*_LHM1293 | *rps*4 | 52.667 | 0.445 | 0.391 | 0.383 | 0.279 | 0.395 | 0.394 |
| *Delphinium sauricum*_LHM1266 | *rps*4 | 52.353 | 0.451 | 0.397 | 0.383 | 0.274 | 0.393 | 0.393 |
| *Delphinium shawurense*_LHM1271 | *rps*4 | 52.64 | 0.449 | 0.393 | 0.383 | 0.269 | 0.391 | 0.391 |
| *Delphinium winklerianum*_LHM1299 | *rps*4 | 52.353 | 0.451 | 0.397 | 0.383 | 0.274 | 0.393 | 0.393 |
| *Delphinium yunnanense*_MW246156 | *rps*4 | 52.353 | 0.451 | 0.397 | 0.383 | 0.274 | 0.393 | 0.393 |
| *Aconitum brachypodum*_MT584424 | *rps*7 | 45.852 | 0.552 | 0.6 | 0.452 | 0.213 | 0.406 | 0.404 |
| *Aconitum delavayi*_OM289058 | *rps*7 | 45.852 | 0.552 | 0.6 | 0.452 | 0.213 | 0.406 | 0.404 |
| *Delphinium aemulans*_LHM1280 | *rps*7 | 46.03 | 0.537 | 0.585 | 0.452 | 0.22 | 0.409 | 0.406 |
| *Delphinium anthriscifolium*_MK253461 | *rps*7 | 45.636 | 0.552 | 0.605 | 0.452 | 0.213 | 0.409 | 0.406 |
| *Delphinium brunonianum*_NC_051554 | *rps*7 | 46.03 | 0.537 | 0.585 | 0.452 | 0.22 | 0.409 | 0.406 |
| *Delphinium candelabrum* var. *monanthum*_MW246165 | *rps*7 | 46.03 | 0.537 | 0.585 | 0.452 | 0.22 | 0.409 | 0.406 |
| *Delphinium ceratophorum*_MK253460 | *rps*7 | 46.03 | 0.537 | 0.585 | 0.452 | 0.22 | 0.409 | 0.406 |
| *Delphinium elatum* var. *sericeum*_LHM1265 | *rps*7 | 46.03 | 0.537 | 0.585 | 0.452 | 0.22 | 0.409 | 0.406 |
| *Delphinium iliense*_LHM1285 | *rps*7 | 46.03 | 0.537 | 0.585 | 0.452 | 0.22 | 0.409 | 0.406 |
| *Delphinium maackianum*_NC_047293 | *rps*7 | 46.03 | 0.537 | 0.585 | 0.452 | 0.22 | 0.409 | 0.406 |
| *Delphinium mollifolium*_LHM1295 | *rps*7 | 46.03 | 0.537 | 0.585 | 0.452 | 0.22 | 0.409 | 0.406 |
| *Delphinium naviculare* var. *lasiocarpum*_LHM1293 | *rps*7 | 46.03 | 0.537 | 0.585 | 0.452 | 0.22 | 0.409 | 0.406 |
| *Delphinium sauricum*_LHM1266 | *rps*7 | 46.03 | 0.537 | 0.585 | 0.452 | 0.22 | 0.409 | 0.406 |
| *Delphinium shawurense*_LHM1271 | *rps*7 | 46.03 | 0.537 | 0.585 | 0.452 | 0.22 | 0.409 | 0.406 |
| *Delphinium winklerianum*_LHM1299 | *rps*7 | 46.03 | 0.537 | 0.585 | 0.452 | 0.22 | 0.409 | 0.406 |
| *Delphinium yunnanense*_MW246156 | *rps*7 | 46.03 | 0.537 | 0.585 | 0.452 | 0.22 | 0.409 | 0.406 |
| *Aconitum brachypodum*_MT584424 | *rps*8 | 43.245 | 0.531 | 0.634 | 0.424 | 0.228 | 0.379 | 0.378 |
| *Aconitum delavayi*_OM289058 | *rps*8 | 43.444 | 0.532 | 0.653 | 0.432 | 0.228 | 0.381 | 0.381 |
| *Delphinium aemulans*_LHM1280 | *rps*8 | 45.27 | 0.51 | 0.625 | 0.439 | 0.213 | 0.376 | 0.376 |
| *Delphinium anthriscifolium*_MK253461 | *rps*8 | 44.51 | 0.513 | 0.634 | 0.432 | 0.252 | 0.384 | 0.383 |
| *Delphinium brunonianum*_NC_051554 | *rps*8 | 44.849 | 0.531 | 0.636 | 0.447 | 0.228 | 0.384 | 0.383 |
| *Delphinium candelabrum* var. *monanthum*_MW246165 | *rps*8 | 44.95 | 0.521 | 0.627 | 0.439 | 0.228 | 0.381 | 0.381 |
| *Delphinium ceratophorum*_MK253460 | *rps*8 | 44.95 | 0.521 | 0.627 | 0.439 | 0.228 | 0.381 | 0.381 |
| *Delphinium elatum* var. *sericeum*_LHM1265 | *rps*8 | 45.27 | 0.51 | 0.625 | 0.439 | 0.213 | 0.376 | 0.376 |
| *Delphinium iliense*_LHM1285 | *rps*8 | 45.27 | 0.51 | 0.625 | 0.439 | 0.213 | 0.376 | 0.376 |
| *Delphinium maackianum*_NC_047293 | *rps*8 | 44.598 | 0.548 | 0.624 | 0.439 | 0.213 | 0.376 | 0.376 |
| *Delphinium mollifolium*_LHM1295 | *rps*8 | 45.27 | 0.51 | 0.625 | 0.439 | 0.213 | 0.376 | 0.376 |
| *Delphinium naviculare* var. *lasiocarpum*_LHM1293 | *rps*8 | 45.27 | 0.51 | 0.625 | 0.439 | 0.213 | 0.376 | 0.376 |
| *Delphinium sauricum*_LHM1266 | *rps*8 | 45.27 | 0.51 | 0.625 | 0.439 | 0.213 | 0.376 | 0.376 |
| *Delphinium shawurense*_LHM1271 | *rps*8 | 45.27 | 0.51 | 0.625 | 0.439 | 0.213 | 0.376 | 0.376 |
| *Delphinium winklerianum*_LHM1299 | *rps*8 | 45.27 | 0.51 | 0.625 | 0.439 | 0.213 | 0.376 | 0.376 |
| *Delphinium yunnanense*_MW246156 | *rps*8 | 44.95 | 0.521 | 0.627 | 0.439 | 0.228 | 0.381 | 0.381 |
| *Aconitum brachypodum*_MT584424 | *rps*11 | 52.319 | 0.465 | 0.451 | 0.58 | 0.226 | 0.454 | 0.453 |
| *Aconitum delavayi*_OM289058 | *rps*11 | 52.319 | 0.465 | 0.451 | 0.58 | 0.226 | 0.454 | 0.453 |
| *Delphinium aemulans*_LHM1280 | *rps*11 | 51.146 | 0.455 | 0.459 | 0.58 | 0.226 | 0.454 | 0.453 |
| *Delphinium anthriscifolium*_MK253461 | *rps*11 | 51.927 | 0.477 | 0.462 | 0.58 | 0.233 | 0.457 | 0.456 |
| *Delphinium brunonianum*_NC_051554 | *rps*11 | 51.146 | 0.455 | 0.459 | 0.58 | 0.226 | 0.454 | 0.453 |
| *Delphinium candelabrum* var. *monanthum*_MW246165 | *rps*11 | 49.821 | 0.472 | 0.481 | 0.58 | 0.218 | 0.452 | 0.451 |
| *Delphinium ceratophorum*_MK253460 | *rps*11 | 51.146 | 0.455 | 0.459 | 0.58 | 0.226 | 0.454 | 0.453 |
| *Delphinium elatum* var. *sericeum*_LHM1265 | *rps*11 | 51.146 | 0.455 | 0.459 | 0.58 | 0.226 | 0.454 | 0.453 |
| *Delphinium iliense*_LHM1285 | *rps*11 | 51.146 | 0.455 | 0.459 | 0.58 | 0.226 | 0.454 | 0.453 |
| *Delphinium maackianum*_NC_047293 | *rps*11 | 51.146 | 0.455 | 0.459 | 0.58 | 0.226 | 0.454 | 0.453 |
| *Delphinium mollifolium*_LHM1295 | *rps*11 | 51.146 | 0.455 | 0.459 | 0.58 | 0.226 | 0.454 | 0.453 |
| *Delphinium naviculare* var. *lasiocarpum*_LHM1293 | *rps*11 | 51.146 | 0.455 | 0.459 | 0.58 | 0.226 | 0.454 | 0.453 |
| *Delphinium sauricum*_LHM1266 | *rps*11 | 51.146 | 0.455 | 0.459 | 0.58 | 0.226 | 0.454 | 0.453 |
| *Delphinium shawurense*_LHM1271 | *rps*11 | 51.146 | 0.455 | 0.459 | 0.58 | 0.226 | 0.454 | 0.453 |
| *Delphinium winklerianum*_LHM1299 | *rps*11 | 51.146 | 0.455 | 0.459 | 0.58 | 0.226 | 0.454 | 0.453 |
| *Delphinium yunnanense*_MW246156 | *rps*11 | 51.146 | 0.455 | 0.459 | 0.58 | 0.226 | 0.454 | 0.453 |
| *Aconitum brachypodum*_MT584424 | *rps*12 | 43.795 | 0.523 | 0.59 | 0.48 | 0.238 | 0.415 | 0.411 |
| *Aconitum delavayi*_OM289058 | *rps*12 | 43.795 | 0.523 | 0.59 | 0.48 | 0.238 | 0.415 | 0.411 |
| *Delphinium aemulans*_LHM1280 | *rps*12 | 44.138 | 0.508 | 0.582 | 0.48 | 0.246 | 0.417 | 0.414 |
| *Delphinium anthriscifolium*_MK253461 | *rps*12 | 44.138 | 0.508 | 0.582 | 0.48 | 0.246 | 0.417 | 0.414 |
| *Delphinium brunonianum*_NC_051554 | *rps*12 | 44.138 | 0.508 | 0.582 | 0.48 | 0.246 | 0.417 | 0.414 |
| *Delphinium candelabrum* var. *monanthum*_MW246165 | *rps*12 | 44.138 | 0.508 | 0.582 | 0.48 | 0.246 | 0.417 | 0.414 |
| *Delphinium ceratophorum*_MK253460 | *rps*12 | 44.138 | 0.508 | 0.582 | 0.48 | 0.246 | 0.417 | 0.414 |
| *Delphinium elatum* var. *sericeum*_LHM1265 | *rps*12 | 44.138 | 0.508 | 0.582 | 0.48 | 0.246 | 0.417 | 0.414 |
| *Delphinium iliense*_LHM1285 | *rps*12 | 44.138 | 0.508 | 0.582 | 0.48 | 0.246 | 0.417 | 0.414 |
| *Delphinium maackianum*_NC_047293 | *rps*12 | 44.138 | 0.508 | 0.582 | 0.48 | 0.246 | 0.417 | 0.414 |
| *Delphinium mollifolium*_LHM1295 | *rps*12 | 44.138 | 0.508 | 0.582 | 0.48 | 0.246 | 0.417 | 0.414 |
| *Delphinium naviculare* var. *lasiocarpum*_LHM1293 | *rps*12 | 44.138 | 0.508 | 0.582 | 0.48 | 0.246 | 0.417 | 0.414 |
| *Delphinium sauricum*_LHM1266 | *rps*12 | 44.138 | 0.508 | 0.582 | 0.48 | 0.246 | 0.417 | 0.414 |
| *Delphinium shawurense*_LHM1271 | *rps*12 | 44.138 | 0.508 | 0.582 | 0.48 | 0.246 | 0.417 | 0.414 |
| *Delphinium winklerianum*_LHM1299 | *rps*12 | 44.138 | 0.508 | 0.582 | 0.48 | 0.246 | 0.417 | 0.414 |
| *Delphinium yunnanense*_MW246156 | *rps*12 | 44.138 | 0.508 | 0.582 | 0.48 | 0.246 | 0.417 | 0.414 |
| *Aconitum brachypodum*_MT584424 | *rps*14 | 40.285 | 0.567 | 0.697 | 0.49 | 0.281 | 0.41 | 0.406 |
| *Aconitum delavayi*_OM289058 | *rps*14 | 40.285 | 0.567 | 0.697 | 0.49 | 0.281 | 0.41 | 0.406 |
| *Delphinium aemulans*_LHM1280 | *rps*14 | 41.818 | 0.539 | 0.653 | 0.49 | 0.313 | 0.42 | 0.416 |
| *Delphinium anthriscifolium*_MK253461 | *rps*14 | 38.746 | 0.561 | 0.685 | 0.49 | 0.302 | 0.417 | 0.413 |
| *Delphinium brunonianum*_NC_051554 | *rps*14 | 41.818 | 0.539 | 0.653 | 0.49 | 0.313 | 0.42 | 0.416 |
| *Delphinium candelabrum* var. *monanthum*_MW246165 | *rps*14 | 41.818 | 0.539 | 0.653 | 0.49 | 0.313 | 0.42 | 0.416 |
| *Delphinium ceratophorum*_MK253460 | *rps*14 | 41.818 | 0.539 | 0.653 | 0.49 | 0.313 | 0.42 | 0.416 |
| *Delphinium elatum* var. *sericeum*_LHM1265 | *rps*14 | 41.818 | 0.539 | 0.653 | 0.49 | 0.313 | 0.42 | 0.416 |
| *Delphinium iliense*_LHM1285 | *rps*14 | 41.818 | 0.539 | 0.653 | 0.49 | 0.313 | 0.42 | 0.416 |
| *Delphinium maackianum*_NC_047293 | *rps*14 | 41.818 | 0.539 | 0.653 | 0.49 | 0.313 | 0.42 | 0.416 |
| *Delphinium mollifolium*_LHM1295 | *rps*14 | 41.818 | 0.539 | 0.653 | 0.49 | 0.313 | 0.42 | 0.416 |
| *Delphinium naviculare* var. *lasiocarpum*_LHM1293 | *rps*14 | 41.818 | 0.539 | 0.653 | 0.49 | 0.313 | 0.42 | 0.416 |
| *Delphinium sauricum*_LHM1266 | *rps*14 | 41.818 | 0.539 | 0.653 | 0.49 | 0.313 | 0.42 | 0.416 |
| *Delphinium shawurense*_LHM1271 | *rps*14 | 41.818 | 0.539 | 0.653 | 0.49 | 0.313 | 0.42 | 0.416 |
| *Delphinium winklerianum*_LHM1299 | *rps*14 | 41.818 | 0.539 | 0.653 | 0.49 | 0.313 | 0.42 | 0.416 |
| *Delphinium yunnanense*_MW246156 | *rps*14 | 41.818 | 0.539 | 0.653 | 0.49 | 0.313 | 0.42 | 0.416 |
| *Aconitum brachypodum*_MT584424 | *rps*15 | 46.117 | 0.589 | 0.671 | 0.278 | 0.236 | 0.319 | 0.315 |
| *Aconitum delavayi*_OM289058 | *rps*15 | 46.117 | 0.589 | 0.671 | 0.278 | 0.236 | 0.319 | 0.315 |
| *Delphinium aemulans*_LHM1280 | *rps*15 | 44.932 | 0.571 | 0.714 | 0.279 | 0.259 | 0.318 | 0.314 |
| *Delphinium anthriscifolium*_MK253461 | *rps*15 | 45.712 | 0.614 | 0.704 | 0.278 | 0.225 | 0.315 | 0.311 |
| *Delphinium brunonianum*_NC_051554 | *rps*15 | 45.56 | 0.559 | 0.693 | 0.291 | 0.259 | 0.322 | 0.318 |
| *Delphinium candelabrum* var. *monanthum*_MW246165 | *rps*15 | 44.526 | 0.57 | 0.736 | 0.291 | 0.247 | 0.318 | 0.314 |
| *Delphinium ceratophorum*_MK253460 | *rps*15 | 45.56 | 0.559 | 0.693 | 0.291 | 0.259 | 0.322 | 0.318 |
| *Delphinium elatum* var. *sericeum*_LHM1265 | *rps*15 | 44.932 | 0.571 | 0.714 | 0.279 | 0.259 | 0.318 | 0.314 |
| *Delphinium iliense*_LHM1285 | *rps*15 | 45.56 | 0.559 | 0.693 | 0.291 | 0.259 | 0.322 | 0.318 |
| *Delphinium maackianum*_NC_047293 | *rps*15 | 43.947 | 0.564 | 0.721 | 0.291 | 0.247 | 0.318 | 0.314 |
| *Delphinium mollifolium*_LHM1295 | *rps*15 | 45.56 | 0.559 | 0.693 | 0.291 | 0.259 | 0.322 | 0.318 |
| *Delphinium naviculare* var. *lasiocarpum*_LHM1293 | *rps*15 | 45.56 | 0.559 | 0.693 | 0.291 | 0.259 | 0.322 | 0.318 |
| *Delphinium sauricum*_LHM1266 | *rps*15 | 45.56 | 0.559 | 0.693 | 0.291 | 0.259 | 0.322 | 0.318 |
| *Delphinium shawurense*_LHM1271 | *rps*15 | 44.932 | 0.571 | 0.714 | 0.279 | 0.259 | 0.318 | 0.314 |
| *Delphinium winklerianum*_LHM1299 | *rps*15 | 45.56 | 0.559 | 0.693 | 0.291 | 0.259 | 0.322 | 0.318 |
| *Delphinium yunnanense*_MW246156 | *rps*15 | 43.931 | 0.557 | 0.725 | 0.291 | 0.247 | 0.318 | 0.314 |
| *Aconitum brachypodum*_MT584424 | *rps*18 | 36.768 | 0.681 | 0.902 | 0.436 | 0.202 | 0.34 | 0.34 |
| *Aconitum delavayi*_OM289058 | *rps*18 | 36.768 | 0.681 | 0.902 | 0.436 | 0.202 | 0.34 | 0.34 |
| *Delphinium aemulans*_LHM1280 | *rps*18 | 37.61 | 0.665 | 0.866 | 0.446 | 0.222 | 0.35 | 0.35 |
| *Delphinium anthriscifolium*_MK253461 | *rps*18 | 37.108 | 0.669 | 0.886 | 0.446 | 0.212 | 0.347 | 0.346 |
| *Delphinium brunonianum*_NC_051554 | *rps*18 | 37.61 | 0.665 | 0.866 | 0.446 | 0.222 | 0.35 | 0.35 |
| *Delphinium candelabrum* var. *monanthum*_MW246165 | *rps*18 | 37.61 | 0.665 | 0.866 | 0.446 | 0.222 | 0.35 | 0.35 |
| *Delphinium ceratophorum*_MK253460 | *rps*18 | 38.673 | 0.652 | 0.82 | 0.446 | 0.222 | 0.35 | 0.35 |
| *Delphinium elatum* var. *sericeum*_LHM1265 | *rps*18 | 37.61 | 0.665 | 0.866 | 0.446 | 0.222 | 0.35 | 0.35 |
| *Delphinium iliense*_LHM1285 | *rps*18 | 37.61 | 0.665 | 0.866 | 0.446 | 0.222 | 0.35 | 0.35 |
| *Delphinium maackianum*_NC_047293 | *rps*18 | 37.61 | 0.665 | 0.866 | 0.446 | 0.222 | 0.35 | 0.35 |
| *Delphinium mollifolium*_LHM1295 | *rps*18 | 37.61 | 0.665 | 0.866 | 0.446 | 0.222 | 0.35 | 0.35 |
| *Delphinium naviculare* var. *lasiocarpum*_LHM1293 | *rps*18 | 37.61 | 0.665 | 0.866 | 0.446 | 0.222 | 0.35 | 0.35 |
| *Delphinium sauricum*_LHM1266 | *rps*18 | 36.78 | 0.674 | 0.901 | 0.446 | 0.214 | 0.347 | 0.346 |
| *Delphinium shawurense*_LHM1271 | *rps*18 | 37.61 | 0.665 | 0.866 | 0.446 | 0.222 | 0.35 | 0.35 |
| *Delphinium winklerianum*_LHM1299 | *rps*18 | 37.61 | 0.665 | 0.866 | 0.446 | 0.222 | 0.35 | 0.35 |
| *Delphinium yunnanense*_MW246156 | *rps*18 | 37.61 | 0.665 | 0.866 | 0.446 | 0.222 | 0.35 | 0.35 |
| *Aconitum brachypodum*_MT584424 | *rps*19 | 49.717 | 0.556 | 0.651 | 0.402 | 0.264 | 0.391 | 0.387 |
| *Aconitum delavayi*_OM289058 | *rps*19 | 49.717 | 0.556 | 0.651 | 0.402 | 0.264 | 0.391 | 0.387 |
| *Delphinium aemulans*_LHM1280 | *rps*19 | 49.353 | 0.525 | 0.628 | 0.402 | 0.27 | 0.391 | 0.387 |
| *Delphinium anthriscifolium*_MK253461 | *rps*19 | 50.743 | 0.551 | 0.617 | 0.413 | 0.25 | 0.391 | 0.387 |
| *Delphinium brunonianum*_NC_051554 | *rps*19 | 50.301 | 0.51 | 0.609 | 0.402 | 0.27 | 0.388 | 0.384 |
| *Delphinium candelabrum* var. *monanthum*_MW246165 | *rps*19 | 49.246 | 0.527 | 0.634 | 0.391 | 0.27 | 0.388 | 0.384 |
| *Delphinium ceratophorum*_MK253460 | *rps*19 | 49.501 | 0.523 | 0.622 | 0.402 | 0.27 | 0.388 | 0.384 |
| *Delphinium elatum* var. *sericeum*_LHM1265 | *rps*19 | 49.353 | 0.525 | 0.628 | 0.402 | 0.27 | 0.391 | 0.387 |
| *Delphinium iliense*_LHM1285 | *rps*19 | 49.353 | 0.525 | 0.628 | 0.402 | 0.27 | 0.391 | 0.387 |
| *Delphinium maackianum*_NC_047293 | *rps*19 | 50.152 | 0.513 | 0.61 | 0.402 | 0.281 | 0.395 | 0.391 |
| *Delphinium mollifolium*_LHM1295 | *rps*19 | 49.353 | 0.525 | 0.628 | 0.402 | 0.27 | 0.391 | 0.387 |
| *Delphinium naviculare* var. *lasiocarpum*_LHM1293 | *rps*19 | 49.353 | 0.525 | 0.628 | 0.402 | 0.27 | 0.391 | 0.387 |
| *Delphinium sauricum*_LHM1266 | *rps*19 | 49.353 | 0.525 | 0.628 | 0.402 | 0.27 | 0.391 | 0.387 |
| *Delphinium shawurense*_LHM1271 | *rps*19 | 49.353 | 0.525 | 0.628 | 0.402 | 0.27 | 0.391 | 0.387 |
| *Delphinium winklerianum*_LHM1299 | *rps*19 | 49.353 | 0.525 | 0.628 | 0.402 | 0.27 | 0.391 | 0.387 |
| *Delphinium yunnanense*_MW246156 | *rps*19 | 49.353 | 0.525 | 0.628 | 0.402 | 0.258 | 0.388 | 0.384 |
| *Aconitum brachypodum*_MT584424 | *ycf*1 | 48.761 | 0.401 | 0.296 | 0.309 | 0.251 | 0.322 | 0.321 |
| *Aconitum delavayi*_OM289058 | *ycf*1 | 48.744 | 0.403 | 0.297 | 0.308 | 0.25 | 0.32 | 0.32 |
| *Delphinium aemulans*_LHM1280 | *ycf*1 | 50.285 | 0.383 | 0.262 | 0.305 | 0.262 | 0.322 | 0.322 |
| *Delphinium anthriscifolium*_MK253461 | *ycf*1 | 49.899 | 0.383 | 0.262 | 0.306 | 0.26 | 0.324 | 0.323 |
| *Delphinium brunonianum*_NC_051554 | *ycf*1 | 50.432 | 0.379 | 0.261 | 0.305 | 0.264 | 0.323 | 0.322 |
| *Delphinium candelabrum* var. *monanthum*_MW246165 | *ycf*1 | 49.973 | 0.382 | 0.269 | 0.306 | 0.26 | 0.32 | 0.32 |
| *Delphinium ceratophorum*_MK253460 | *ycf*1 | 50.58 | 0.372 | 0.256 | 0.307 | 0.266 | 0.323 | 0.323 |
| *Delphinium elatum* var. *sericeum*_LHM1265 | *ycf*1 | 50.222 | 0.385 | 0.263 | 0.305 | 0.26 | 0.322 | 0.321 |
| *Delphinium iliense*_LHM1285 | *ycf*1 | 50.173 | 0.385 | 0.266 | 0.304 | 0.261 | 0.322 | 0.321 |
| *Delphinium maackianum*_NC_047293 | *ycf*1 | 50.211 | 0.386 | 0.267 | 0.305 | 0.259 | 0.32 | 0.319 |
| *Delphinium mollifolium*_LHM1295 | *ycf*1 | 50.289 | 0.382 | 0.262 | 0.305 | 0.262 | 0.322 | 0.322 |
| *Delphinium naviculare* var. *lasiocarpum*_LHM1293 | *ycf*1 | 50.173 | 0.385 | 0.266 | 0.304 | 0.261 | 0.322 | 0.321 |
| *Delphinium sauricum*_LHM1266 | *ycf*1 | 50.362 | 0.381 | 0.261 | 0.305 | 0.262 | 0.323 | 0.322 |
| *Delphinium shawurense*_LHM1271 | *ycf*1 | 50.299 | 0.385 | 0.262 | 0.305 | 0.261 | 0.322 | 0.321 |
| *Delphinium winklerianum*_LHM1299 | *ycf*1 | 50.262 | 0.381 | 0.262 | 0.305 | 0.262 | 0.323 | 0.322 |
| *Delphinium yunnanense*_MW246156 | *ycf*1 | 50.049 | 0.384 | 0.269 | 0.304 | 0.26 | 0.32 | 0.319 |
| *Aconitum brachypodum*_MT584424 | *ycf*2 | 53.357 | 0.271 | 0.173 | 0.352 | 0.341 | 0.38 | 0.38 |
| *Aconitum delavayi*_OM289058 | *ycf*2 | 53.361 | 0.272 | 0.173 | 0.353 | 0.34 | 0.38 | 0.38 |
| *Delphinium aemulans*_LHM1280 | *ycf*2 | 53.77 | 0.272 | 0.17 | 0.355 | 0.341 | 0.383 | 0.382 |
| *Delphinium anthriscifolium*_MK253461 | *ycf*2 | 53.658 | 0.268 | 0.168 | 0.351 | 0.342 | 0.381 | 0.381 |
| *Delphinium brunonianum*_NC_051554 | *ycf*2 | 53.732 | 0.272 | 0.171 | 0.355 | 0.34 | 0.382 | 0.382 |
| *Delphinium candelabrum* var. *monanthum*_MW246165 | *ycf*2 | 53.733 | 0.272 | 0.171 | 0.355 | 0.34 | 0.382 | 0.382 |
| *Delphinium ceratophorum*_MK253460 | *ycf*2 | 53.763 | 0.271 | 0.17 | 0.355 | 0.34 | 0.383 | 0.383 |
| *Delphinium elatum* var. *sericeum*_LHM1265 | *ycf*2 | 53.769 | 0.272 | 0.171 | 0.355 | 0.34 | 0.383 | 0.382 |
| *Delphinium iliense*_LHM1285 | *ycf*2 | 53.743 | 0.272 | 0.171 | 0.355 | 0.34 | 0.383 | 0.382 |
| *Delphinium maackianum*_NC_047293 | *ycf*2 | 53.708 | 0.272 | 0.171 | 0.355 | 0.34 | 0.382 | 0.382 |
| *Delphinium mollifolium*_LHM1295 | *ycf*2 | 53.743 | 0.272 | 0.171 | 0.355 | 0.34 | 0.383 | 0.382 |
| *Delphinium naviculare* var. *lasiocarpum*_LHM1293 | *ycf*2 | 53.743 | 0.272 | 0.171 | 0.355 | 0.34 | 0.383 | 0.382 |
| *Delphinium sauricum*_LHM1266 | *ycf*2 | 53.743 | 0.272 | 0.171 | 0.355 | 0.34 | 0.383 | 0.382 |
| *Delphinium shawurense*_LHM1271 | *ycf*2 | 53.747 | 0.272 | 0.171 | 0.355 | 0.34 | 0.382 | 0.382 |
| *Delphinium winklerianum*_LHM1299 | *ycf*2 | 53.743 | 0.272 | 0.171 | 0.355 | 0.34 | 0.383 | 0.382 |
| *Delphinium yunnanense*_MW246156 | *ycf*2 | 53.768 | 0.271 | 0.17 | 0.355 | 0.341 | 0.382 | 0.382 |
| *Aconitum brachypodum*_MT584424 | *ycf*3 | 53.705 | 0.444 | 0.4 | 0.395 | 0.295 | 0.401 | 0.399 |
| *Aconitum delavayi*_OM289058 | *ycf*3 | 55.065 | 0.451 | 0.395 | 0.399 | 0.284 | 0.397 | 0.394 |
| *Delphinium aemulans*_LHM1280 | *ycf*3 | 56.872 | 0.451 | 0.384 | 0.399 | 0.296 | 0.401 | 0.398 |
| *Delphinium anthriscifolium*_MK253461 | *ycf*3 | 56.503 | 0.445 | 0.38 | 0.399 | 0.296 | 0.401 | 0.398 |
| *Delphinium brunonianum*_NC_051554 | *ycf*3 | 56.872 | 0.451 | 0.384 | 0.399 | 0.296 | 0.401 | 0.398 |
| *Delphinium candelabrum* var. *monanthum*_MW246165 | *ycf*3 | 56.342 | 0.452 | 0.389 | 0.399 | 0.29 | 0.399 | 0.396 |
| *Delphinium ceratophorum*_MK253460 | *ycf*3 | 56.872 | 0.451 | 0.384 | 0.399 | 0.296 | 0.401 | 0.398 |
| *Delphinium elatum* var. *sericeum*_LHM1265 | *ycf*3 | 57.188 | 0.438 | 0.375 | 0.399 | 0.302 | 0.403 | 0.4 |
| *Delphinium iliense*_LHM1285 | *ycf*3 | 56.872 | 0.451 | 0.384 | 0.399 | 0.296 | 0.401 | 0.398 |
| *Delphinium maackianum*_NC_047293 | *ycf*3 | 54.907 | 0.478 | 0.419 | 0.399 | 0.284 | 0.397 | 0.394 |
| *Delphinium mollifolium*_LHM1295 | *ycf*3 | 56.872 | 0.451 | 0.384 | 0.399 | 0.296 | 0.401 | 0.398 |
| *Delphinium naviculare* var. *lasiocarpum*_LHM1293 | *ycf*3 | 56.872 | 0.451 | 0.384 | 0.399 | 0.296 | 0.401 | 0.398 |
| *Delphinium sauricum*_LHM1266 | *ycf*3 | 56.872 | 0.451 | 0.384 | 0.399 | 0.296 | 0.401 | 0.398 |
| *Delphinium shawurense*_LHM1271 | *ycf*3 | 57.188 | 0.438 | 0.375 | 0.399 | 0.302 | 0.403 | 0.4 |
| *Delphinium winklerianum*_LHM1299 | *ycf*3 | 56.872 | 0.451 | 0.384 | 0.399 | 0.296 | 0.401 | 0.398 |
| *Delphinium yunnanense*_MW246156 | *ycf*3 | 56.342 | 0.452 | 0.389 | 0.399 | 0.29 | 0.399 | 0.396 |
| *Aconitum brachypodum*_MT584424 | *ycf*4 | 56.244 | 0.345 | 0.314 | 0.424 | 0.328 | 0.408 | 0.407 |
| *Aconitum delavayi*_OM289058 | *ycf*4 | 56.449 | 0.345 | 0.317 | 0.424 | 0.328 | 0.409 | 0.409 |
| *Delphinium aemulans*_LHM1280 | *ycf*4 | 53.707 | 0.363 | 0.342 | 0.424 | 0.305 | 0.4 | 0.4 |
| *Delphinium anthriscifolium*_MK253461 | *ycf*4 | 53.459 | 0.372 | 0.352 | 0.424 | 0.303 | 0.4 | 0.4 |
| *Delphinium brunonianum*_NC_051554 | *ycf*4 | 52.811 | 0.375 | 0.361 | 0.424 | 0.299 | 0.399 | 0.398 |
| *Delphinium candelabrum* var. *monanthum*_MW246165 | *ycf*4 | 52.315 | 0.377 | 0.368 | 0.424 | 0.299 | 0.399 | 0.398 |
| *Delphinium ceratophorum*_MK253460 | *ycf*4 | 52.79 | 0.38 | 0.362 | 0.424 | 0.293 | 0.397 | 0.396 |
| *Delphinium elatum* var. *sericeum*_LHM1265 | *ycf*4 | 53.707 | 0.363 | 0.342 | 0.424 | 0.305 | 0.4 | 0.4 |
| *Delphinium iliense*_LHM1285 | *ycf*4 | 53.707 | 0.363 | 0.342 | 0.424 | 0.305 | 0.4 | 0.4 |
| *Delphinium maackianum*_NC_047293 | *ycf*4 | 52.511 | 0.381 | 0.365 | 0.424 | 0.299 | 0.399 | 0.398 |
| *Delphinium mollifolium*_LHM1295 | *ycf*4 | 53.707 | 0.363 | 0.342 | 0.424 | 0.305 | 0.4 | 0.4 |
| *Delphinium naviculare* var. *lasiocarpum*_LHM1293 | *ycf*4 | 53.707 | 0.363 | 0.342 | 0.424 | 0.305 | 0.4 | 0.4 |
| *Delphinium sauricum*_LHM1266 | *ycf*4 | 53.707 | 0.363 | 0.342 | 0.424 | 0.305 | 0.4 | 0.4 |
| *Delphinium shawurense*_LHM1271 | *ycf*4 | 53.707 | 0.363 | 0.342 | 0.424 | 0.305 | 0.4 | 0.4 |
| *Delphinium winklerianum*_LHM1299 | *ycf*4 | 53.707 | 0.363 | 0.342 | 0.424 | 0.305 | 0.4 | 0.4 |
| *Delphinium yunnanense*_MW246156 | *ycf*4 | 52.811 | 0.375 | 0.361 | 0.424 | 0.299 | 0.399 | 0.398 |
