# Supplementary material for: Complete chloroplast genomes of eight Delphinium taxa (Ranunculaceae) endemic to Xinjiang, China: insights into genome structure, comparative analysis, and phylogenetic relationships
Source: BMC Plant Biol. 2024 Jun 26;24:600. doi: 10.1186/s12870-024-05279-y (PMC11201361; doi:10.1186/s12870-024-05279-y)
Supplement: Supplementary file 7 — Supplementary Material 7 [file 12870_2024_5279_MOESM7_ESM.docx]

**TABLE S7** The codon count and RSCU information in 14 *Delphinium* taxa.

|  |  | *Delphinium aemulans* | | *Delphinium anthriscifolium* | | *Delphinium brunonianum* | | *Delphinium candelabrum* var. *monanthum* | | *Delphinium ceratophorum* | | *Delphinium elatum* var. *sericeum* | | *Delphinium iliense* | | *Delphinium maackianum* | | *Delphinium mollifolium* | | *Delphinium naviculare* var. *lasiocarpum* | | *Delphinium sauricum* | | *Delphinium shawurense* | | *Delphinium winklerianum* | | *Delphinium yunnanense* | |
| --- | --- | --- | --- | --- | --- | --- | --- | --- | --- | --- | --- | --- | --- | --- | --- | --- | --- | --- | --- | --- | --- | --- | --- | --- | --- | --- | --- | --- | --- |
| Amino acid | codon | count | RSCU | Count | RSCU | Count | RSCU | Count | RSCU | Count | RSCU | Count | RSCU | Count | RSCU | Count | RSCU | Count | RSCU | Count | RSCU | Count | RSCU | Count | RSCU | Count | RSCU | Count | RSCU |
| Phe | UUU | 832 | 1.32 | 834 | 1.32 | 830 | 1.32 | 838 | 1.32 | 834 | 1.32 | 833 | 1.32 | 836 | 1.32 | 839 | 1.33 | 834 | 1.32 | 837 | 1.32 | 836 | 1.32 | 833 | 1.32 | 834 | 1.32 | 840 | 1.32 |
|  | UUC | 430 | 0.68 | 425 | 0.68 | 432 | 0.68 | 430 | 0.68 | 433 | 0.68 | 429 | 0.68 | 429 | 0.68 | 423 | 0.67 | 428 | 0.68 | 429 | 0.68 | 427 | 0.68 | 429 | 0.68 | 429 | 0.68 | 429 | 0.68 |
| Leu | UUA | 735 | 1.89 | 737 | 1.9 | 735 | 1.89 | 732 | 1.88 | 729 | 1.87 | 735 | 1.89 | 736 | 1.89 | 739 | 1.9 | 735 | 1.89 | 735 | 1.89 | 734 | 1.89 | 735 | 1.89 | 734 | 1.89 | 735 | 1.89 |
|  | UUG | 494 | 1.27 | 485 | 1.25 | 498 | 1.28 | 496 | 1.28 | 500 | 1.28 | 494 | 1.27 | 496 | 1.27 | 495 | 1.27 | 497 | 1.28 | 497 | 1.28 | 495 | 1.27 | 494 | 1.27 | 495 | 1.27 | 492 | 1.27 |
|  | CUU | 475 | 1.22 | 482 | 1.24 | 476 | 1.23 | 477 | 1.23 | 484 | 1.24 | 476 | 1.22 | 476 | 1.22 | 478 | 1.23 | 477 | 1.23 | 476 | 1.22 | 476 | 1.23 | 476 | 1.22 | 477 | 1.23 | 477 | 1.23 |
|  | CUC | 160 | 0.41 | 155 | 0.4 | 161 | 0.41 | 161 | 0.41 | 160 | 0.41 | 162 | 0.42 | 160 | 0.41 | 161 | 0.41 | 160 | 0.41 | 159 | 0.41 | 160 | 0.41 | 161 | 0.41 | 160 | 0.41 | 161 | 0.41 |
|  | CUA | 325 | 0.84 | 316 | 0.81 | 317 | 0.82 | 320 | 0.82 | 321 | 0.82 | 322 | 0.83 | 323 | 0.83 | 317 | 0.81 | 322 | 0.83 | 323 | 0.83 | 322 | 0.83 | 322 | 0.83 | 323 | 0.83 | 322 | 0.83 |
|  | CUG | 145 | 0.37 | 153 | 0.39 | 143 | 0.37 | 146 | 0.38 | 145 | 0.37 | 144 | 0.37 | 144 | 0.37 | 146 | 0.38 | 144 | 0.37 | 144 | 0.37 | 143 | 0.37 | 144 | 0.37 | 144 | 0.37 | 145 | 0.37 |
| Ile | AUU | 944 | 1.47 | 945 | 1.47 | 943 | 1.46 | 939 | 1.45 | 939 | 1.46 | 941 | 1.46 | 942 | 1.46 | 945 | 1.46 | 942 | 1.46 | 942 | 1.46 | 941 | 1.46 | 943 | 1.47 | 939 | 1.46 | 938 | 1.45 |
|  | AUC | 376 | 0.58 | 374 | 0.58 | 378 | 0.59 | 384 | 0.59 | 379 | 0.59 | 376 | 0.58 | 381 | 0.59 | 376 | 0.58 | 378 | 0.59 | 381 | 0.59 | 380 | 0.59 | 376 | 0.58 | 381 | 0.59 | 384 | 0.6 |
|  | AUA | 612 | 0.95 | 604 | 0.94 | 616 | 0.95 | 615 | 0.95 | 614 | 0.95 | 614 | 0.95 | 609 | 0.95 | 615 | 0.95 | 613 | 0.95 | 609 | 0.95 | 613 | 0.95 | 612 | 0.95 | 611 | 0.95 | 614 | 0.95 |
| Met | AUG | 536 | 1 | 557 | 1 | 538 | 1 | 532 | 1 | 535 | 1 | 538 | 1 | 539 | 1 | 539 | 1 | 538 | 1 | 539 | 1 | 540 | 1 | 536 | 1 | 539 | 1 | 534 | 1 |
| Val | GUU | 472 | 1.48 | 477 | 1.5 | 472 | 1.48 | 469 | 1.48 | 470 | 1.48 | 473 | 1.49 | 472 | 1.48 | 473 | 1.49 | 471 | 1.48 | 472 | 1.48 | 472 | 1.48 | 473 | 1.49 | 472 | 1.48 | 468 | 1.48 |
|  | GUC | 142 | 0.45 | 135 | 0.42 | 142 | 0.45 | 141 | 0.44 | 141 | 0.44 | 142 | 0.45 | 143 | 0.45 | 139 | 0.44 | 142 | 0.45 | 143 | 0.45 | 142 | 0.45 | 142 | 0.45 | 141 | 0.44 | 141 | 0.45 |
|  | GUA | 488 | 1.53 | 493 | 1.55 | 488 | 1.53 | 488 | 1.54 | 489 | 1.54 | 488 | 1.53 | 488 | 1.53 | 489 | 1.54 | 489 | 1.54 | 488 | 1.53 | 489 | 1.54 | 489 | 1.54 | 491 | 1.54 | 487 | 1.54 |
|  | GUG | 170 | 0.53 | 166 | 0.52 | 170 | 0.53 | 172 | 0.54 | 169 | 0.53 | 171 | 0.54 | 170 | 0.53 | 171 | 0.54 | 170 | 0.53 | 170 | 0.53 | 170 | 0.53 | 169 | 0.53 | 169 | 0.53 | 170 | 0.54 |
| Ser | UCU | 455 | 1.64 | 458 | 1.65 | 456 | 1.64 | 459 | 1.65 | 455 | 1.64 | 454 | 1.63 | 455 | 1.63 | 455 | 1.64 | 458 | 1.65 | 455 | 1.63 | 457 | 1.64 | 455 | 1.64 | 457 | 1.64 | 458 | 1.65 |
|  | UCC | 281 | 1.01 | 272 | 0.98 | 283 | 1.02 | 280 | 1.01 | 286 | 1.03 | 282 | 1.01 | 282 | 1.01 | 281 | 1.01 | 281 | 1.01 | 282 | 1.01 | 283 | 1.01 | 281 | 1.01 | 284 | 1.02 | 280 | 1.01 |
|  | UCA | 341 | 1.23 | 340 | 1.23 | 338 | 1.21 | 340 | 1.22 | 336 | 1.21 | 342 | 1.23 | 340 | 1.22 | 339 | 1.22 | 339 | 1.22 | 340 | 1.22 | 339 | 1.22 | 342 | 1.23 | 338 | 1.21 | 340 | 1.22 |
|  | UCG | 155 | 0.56 | 159 | 0.57 | 158 | 0.57 | 157 | 0.56 | 157 | 0.56 | 155 | 0.56 | 157 | 0.56 | 155 | 0.56 | 155 | 0.56 | 157 | 0.56 | 156 | 0.56 | 153 | 0.55 | 155 | 0.56 | 156 | 0.56 |
| Pro | CCU | 376 | 1.55 | 370 | 1.54 | 375 | 1.55 | 374 | 1.54 | 374 | 1.55 | 375 | 1.55 | 375 | 1.55 | 375 | 1.55 | 375 | 1.55 | 375 | 1.55 | 374 | 1.54 | 376 | 1.55 | 375 | 1.55 | 375 | 1.55 |
|  | CCC | 194 | 0.8 | 203 | 0.84 | 195 | 0.81 | 196 | 0.81 | 195 | 0.81 | 195 | 0.8 | 195 | 0.8 | 195 | 0.8 | 196 | 0.81 | 195 | 0.8 | 196 | 0.81 | 194 | 0.8 | 195 | 0.8 | 195 | 0.8 |
|  | CCA | 275 | 1.14 | 271 | 1.12 | 276 | 1.14 | 277 | 1.14 | 277 | 1.14 | 276 | 1.14 | 275 | 1.14 | 276 | 1.14 | 276 | 1.14 | 275 | 1.14 | 275 | 1.13 | 275 | 1.14 | 276 | 1.14 | 279 | 1.15 |
|  | CCG | 124 | 0.51 | 120 | 0.5 | 121 | 0.5 | 123 | 0.51 | 122 | 0.5 | 124 | 0.51 | 124 | 0.51 | 123 | 0.51 | 123 | 0.51 | 124 | 0.51 | 125 | 0.52 | 124 | 0.51 | 124 | 0.51 | 121 | 0.5 |
| Thr | ACU | 459 | 1.57 | 466 | 1.59 | 463 | 1.58 | 458 | 1.57 | 457 | 1.57 | 460 | 1.58 | 460 | 1.58 | 457 | 1.56 | 460 | 1.58 | 460 | 1.58 | 460 | 1.58 | 460 | 1.58 | 460 | 1.58 | 459 | 1.57 |
|  | ACC | 220 | 0.75 | 223 | 0.76 | 223 | 0.76 | 222 | 0.76 | 219 | 0.75 | 220 | 0.75 | 219 | 0.75 | 225 | 0.77 | 219 | 0.75 | 219 | 0.75 | 219 | 0.75 | 220 | 0.75 | 219 | 0.75 | 222 | 0.76 |
|  | ACA | 358 | 1.23 | 354 | 1.21 | 358 | 1.22 | 359 | 1.23 | 361 | 1.24 | 358 | 1.23 | 358 | 1.23 | 363 | 1.24 | 358 | 1.23 | 358 | 1.23 | 358 | 1.23 | 358 | 1.23 | 358 | 1.23 | 359 | 1.23 |
|  | ACG | 130 | 0.45 | 128 | 0.44 | 130 | 0.44 | 129 | 0.44 | 130 | 0.45 | 130 | 0.45 | 129 | 0.44 | 125 | 0.43 | 129 | 0.44 | 129 | 0.44 | 129 | 0.44 | 130 | 0.45 | 129 | 0.44 | 129 | 0.44 |
| Ala | GCU | 541 | 1.73 | 537 | 1.71 | 537 | 1.72 | 538 | 1.72 | 539 | 1.73 | 540 | 1.73 | 540 | 1.73 | 540 | 1.73 | 540 | 1.73 | 540 | 1.73 | 540 | 1.73 | 540 | 1.73 | 540 | 1.73 | 538 | 1.72 |
|  | GCC | 202 | 0.64 | 204 | 0.65 | 204 | 0.65 | 203 | 0.65 | 203 | 0.65 | 202 | 0.65 | 201 | 0.64 | 200 | 0.64 | 201 | 0.64 | 201 | 0.64 | 201 | 0.64 | 203 | 0.65 | 201 | 0.64 | 203 | 0.65 |
|  | GCA | 351 | 1.12 | 353 | 1.12 | 349 | 1.12 | 348 | 1.11 | 345 | 1.1 | 351 | 1.12 | 352 | 1.13 | 351 | 1.12 | 353 | 1.13 | 352 | 1.13 | 352 | 1.13 | 349 | 1.12 | 351 | 1.12 | 348 | 1.11 |
|  | GCG | 159 | 0.51 | 165 | 0.52 | 161 | 0.51 | 160 | 0.51 | 162 | 0.52 | 157 | 0.5 | 158 | 0.51 | 159 | 0.51 | 158 | 0.5 | 158 | 0.51 | 158 | 0.51 | 159 | 0.51 | 159 | 0.51 | 161 | 0.52 |
| Tyr | UAU | 662 | 1.58 | 664 | 1.6 | 660 | 1.59 | 657 | 1.59 | 657 | 1.59 | 662 | 1.59 | 663 | 1.59 | 666 | 1.59 | 663 | 1.59 | 663 | 1.59 | 663 | 1.59 | 663 | 1.59 | 663 | 1.59 | 658 | 1.58 |
|  | UAC | 174 | 0.42 | 168 | 0.4 | 171 | 0.41 | 172 | 0.41 | 172 | 0.41 | 172 | 0.41 | 169 | 0.41 | 170 | 0.41 | 170 | 0.41 | 169 | 0.41 | 170 | 0.41 | 172 | 0.41 | 170 | 0.41 | 173 | 0.42 |
| TER(*) | UAA | 31 | 1.21 | 36 | 1.4 | 32 | 1.25 | 33 | 1.29 | 32 | 1.25 | 31 | 1.21 | 31 | 1.21 | 33 | 1.29 | 31 | 1.21 | 31 | 1.21 | 31 | 1.21 | 31 | 1.21 | 31 | 1.21 | 34 | 1.32 |
|  | UAG | 22 | 0.86 | 21 | 0.82 | 23 | 0.9 | 22 | 0.86 | 23 | 0.9 | 22 | 0.86 | 22 | 0.86 | 22 | 0.86 | 22 | 0.86 | 22 | 0.86 | 22 | 0.86 | 22 | 0.86 | 22 | 0.86 | 22 | 0.86 |
|  | UGA | 24 | 0.94 | 20 | 0.78 | 22 | 0.86 | 22 | 0.86 | 22 | 0.86 | 24 | 0.94 | 24 | 0.94 | 22 | 0.86 | 24 | 0.94 | 24 | 0.94 | 24 | 0.94 | 24 | 0.94 | 24 | 0.94 | 21 | 0.82 |
| His | CAU | 425 | 1.5 | 421 | 1.48 | 422 | 1.5 | 426 | 1.5 | 422 | 1.5 | 425 | 1.5 | 424 | 1.5 | 426 | 1.51 | 424 | 1.5 | 424 | 1.5 | 425 | 1.5 | 425 | 1.5 | 425 | 1.5 | 424 | 1.5 |
|  | CAC | 140 | 0.5 | 149 | 0.52 | 141 | 0.5 | 141 | 0.5 | 140 | 0.5 | 140 | 0.5 | 140 | 0.5 | 139 | 0.49 | 140 | 0.5 | 140 | 0.5 | 140 | 0.5 | 140 | 0.5 | 140 | 0.5 | 141 | 0.5 |
| Gln | CAA | 595 | 1.52 | 589 | 1.53 | 595 | 1.52 | 598 | 1.53 | 597 | 1.52 | 595 | 1.52 | 600 | 1.53 | 595 | 1.53 | 599 | 1.53 | 600 | 1.53 | 598 | 1.53 | 596 | 1.52 | 598 | 1.53 | 600 | 1.53 |
|  | CAG | 187 | 0.48 | 182 | 0.47 | 187 | 0.48 | 183 | 0.47 | 186 | 0.48 | 187 | 0.48 | 186 | 0.47 | 184 | 0.47 | 186 | 0.47 | 186 | 0.47 | 186 | 0.47 | 186 | 0.48 | 186 | 0.47 | 185 | 0.47 |
| Asn | AAU | 816 | 1.56 | 814 | 1.55 | 818 | 1.56 | 813 | 1.56 | 815 | 1.56 | 815 | 1.56 | 814 | 1.55 | 817 | 1.55 | 815 | 1.56 | 814 | 1.55 | 813 | 1.55 | 814 | 1.56 | 813 | 1.56 | 814 | 1.56 |
|  | AAC | 231 | 0.44 | 236 | 0.45 | 231 | 0.44 | 231 | 0.44 | 233 | 0.44 | 231 | 0.44 | 233 | 0.45 | 234 | 0.45 | 232 | 0.44 | 233 | 0.45 | 233 | 0.45 | 231 | 0.44 | 232 | 0.44 | 232 | 0.44 |
| Lys | AAA | 851 | 1.49 | 845 | 1.48 | 850 | 1.5 | 846 | 1.49 | 844 | 1.48 | 851 | 1.49 | 848 | 1.49 | 849 | 1.5 | 849 | 1.49 | 848 | 1.49 | 850 | 1.49 | 849 | 1.49 | 850 | 1.49 | 847 | 1.5 |
|  | AAG | 290 | 0.51 | 296 | 0.52 | 285 | 0.5 | 286 | 0.51 | 293 | 0.52 | 290 | 0.51 | 290 | 0.51 | 283 | 0.5 | 288 | 0.51 | 290 | 0.51 | 288 | 0.51 | 292 | 0.51 | 289 | 0.51 | 286 | 0.5 |
| Asp | GAU | 724 | 1.58 | 717 | 1.56 | 721 | 1.58 | 718 | 1.58 | 721 | 1.58 | 725 | 1.58 | 725 | 1.58 | 719 | 1.58 | 721 | 1.58 | 725 | 1.58 | 721 | 1.58 | 722 | 1.58 | 721 | 1.58 | 719 | 1.58 |
|  | GAC | 190 | 0.42 | 200 | 0.44 | 191 | 0.42 | 190 | 0.42 | 192 | 0.42 | 190 | 0.42 | 190 | 0.42 | 191 | 0.42 | 190 | 0.42 | 190 | 0.42 | 190 | 0.42 | 190 | 0.42 | 190 | 0.42 | 190 | 0.42 |
| Glu | GAA | 860 | 1.48 | 873 | 1.49 | 865 | 1.48 | 860 | 1.48 | 862 | 1.48 | 860 | 1.48 | 861 | 1.48 | 861 | 1.47 | 860 | 1.48 | 861 | 1.48 | 859 | 1.47 | 860 | 1.48 | 859 | 1.47 | 861 | 1.48 |
|  | GAG | 305 | 0.52 | 297 | 0.51 | 306 | 0.52 | 305 | 0.52 | 302 | 0.52 | 305 | 0.52 | 305 | 0.52 | 307 | 0.53 | 306 | 0.52 | 305 | 0.52 | 306 | 0.53 | 305 | 0.52 | 306 | 0.53 | 304 | 0.52 |
| Cys | UGU | 191 | 1.49 | 196 | 1.49 | 190 | 1.5 | 190 | 1.5 | 189 | 1.49 | 189 | 1.49 | 189 | 1.49 | 189 | 1.49 | 189 | 1.49 | 189 | 1.49 | 188 | 1.49 | 191 | 1.5 | 188 | 1.49 | 190 | 1.49 |
|  | UGC | 65 | 0.51 | 67 | 0.51 | 64 | 0.5 | 64 | 0.5 | 65 | 0.51 | 64 | 0.51 | 64 | 0.51 | 65 | 0.51 | 64 | 0.51 | 64 | 0.51 | 64 | 0.51 | 64 | 0.5 | 64 | 0.51 | 65 | 0.51 |
| Trp | UGG | 409 | 1 | 405 | 1 | 408 | 1 | 410 | 1 | 408 | 1 | 411 | 1 | 409 | 1 | 409 | 1 | 409 | 1 | 409 | 1 | 409 | 1 | 410 | 1 | 409 | 1 | 409 | 1 |
| Arg | CGU | 316 | 1.39 | 313 | 1.37 | 318 | 1.4 | 317 | 1.4 | 317 | 1.4 | 316 | 1.39 | 316 | 1.39 | 317 | 1.4 | 316 | 1.39 | 316 | 1.39 | 316 | 1.39 | 316 | 1.39 | 316 | 1.39 | 314 | 1.39 |
|  | CGC | 80 | 0.35 | 85 | 0.37 | 79 | 0.35 | 78 | 0.34 | 78 | 0.34 | 80 | 0.35 | 80 | 0.35 | 78 | 0.34 | 80 | 0.35 | 80 | 0.35 | 80 | 0.35 | 80 | 0.35 | 80 | 0.35 | 78 | 0.35 |
|  | CGA | 301 | 1.33 | 302 | 1.32 | 302 | 1.33 | 297 | 1.31 | 302 | 1.33 | 301 | 1.33 | 300 | 1.32 | 300 | 1.32 | 301 | 1.32 | 300 | 1.32 | 302 | 1.33 | 301 | 1.33 | 302 | 1.33 | 296 | 1.31 |
|  | CGG | 106 | 0.47 | 104 | 0.46 | 105 | 0.46 | 105 | 0.46 | 105 | 0.46 | 106 | 0.47 | 106 | 0.47 | 107 | 0.47 | 106 | 0.47 | 106 | 0.47 | 105 | 0.46 | 106 | 0.47 | 105 | 0.46 | 105 | 0.46 |
|  | AGA | 400 | 1.76 | 408 | 1.79 | 402 | 1.77 | 403 | 1.78 | 398 | 1.75 | 400 | 1.76 | 401 | 1.77 | 400 | 1.76 | 400 | 1.76 | 401 | 1.77 | 400 | 1.76 | 400 | 1.76 | 400 | 1.76 | 404 | 1.79 |
|  | AGG | 158 | 0.7 | 156 | 0.68 | 159 | 0.7 | 157 | 0.69 | 161 | 0.71 | 158 | 0.7 | 160 | 0.7 | 161 | 0.71 | 161 | 0.71 | 160 | 0.7 | 161 | 0.71 | 158 | 0.7 | 160 | 0.7 | 158 | 0.7 |
| Ser | AGU | 338 | 1.22 | 332 | 1.2 | 335 | 1.2 | 338 | 1.21 | 335 | 1.21 | 336 | 1.21 | 337 | 1.21 | 338 | 1.22 | 336 | 1.21 | 337 | 1.21 | 338 | 1.21 | 336 | 1.21 | 337 | 1.21 | 339 | 1.22 |
|  | AGC | 99 | 0.36 | 100 | 0.36 | 100 | 0.36 | 97 | 0.35 | 99 | 0.36 | 100 | 0.36 | 99 | 0.36 | 98 | 0.35 | 100 | 0.36 | 99 | 0.36 | 100 | 0.36 | 100 | 0.36 | 100 | 0.36 | 95 | 0.34 |
| Gly | GGU | 545 | 1.36 | 541 | 1.36 | 543 | 1.35 | 543 | 1.35 | 546 | 1.36 | 544 | 1.35 | 545 | 1.36 | 541 | 1.35 | 545 | 1.36 | 545 | 1.36 | 544 | 1.36 | 543 | 1.35 | 545 | 1.36 | 544 | 1.35 |
|  | GGC | 173 | 0.43 | 174 | 0.44 | 173 | 0.43 | 171 | 0.43 | 172 | 0.43 | 173 | 0.43 | 172 | 0.43 | 175 | 0.44 | 172 | 0.43 | 172 | 0.43 | 172 | 0.43 | 173 | 0.43 | 172 | 0.43 | 174 | 0.43 |
|  | GGA | 614 | 1.53 | 612 | 1.53 | 613 | 1.53 | 616 | 1.54 | 616 | 1.53 | 615 | 1.53 | 613 | 1.53 | 614 | 1.53 | 613 | 1.53 | 613 | 1.53 | 613 | 1.53 | 615 | 1.53 | 613 | 1.53 | 616 | 1.53 |
|  | GGG | 275 | 0.68 | 270 | 0.68 | 276 | 0.69 | 273 | 0.68 | 276 | 0.69 | 275 | 0.68 | 276 | 0.69 | 275 | 0.69 | 276 | 0.69 | 276 | 0.69 | 276 | 0.69 | 275 | 0.68 | 275 | 0.69 | 272 | 0.68 |
