# Supplementary material for: Complete chloroplast genomes of eight Delphinium taxa (Ranunculaceae) endemic to Xinjiang, China: insights into genome structure, comparative analysis, and phylogenetic relationships
Source: BMC Plant Biol. 2024 Jun 26;24:600. doi: 10.1186/s12870-024-05279-y (PMC11201361; doi:10.1186/s12870-024-05279-y)
Supplement: Supplementary file 8 — Supplementary Material 8 [file 12870_2024_5279_MOESM8_ESM.docx]

**TABLE S8** The *d*_N_, *d*_S_, and *ω* ratio under Model 0 and Model 2 in the study. Model 0 assumed the same *d*_N_ / *d*_S_ ratio (*ω* ratio) for all branches in the phylogeny; Model 2 assumed that the outgroup branch had *ω* ratio that differed from that throughout the rest of the tree.

| **Gene** | **Model 0** | | | **Model 2** | | | | | **chi2 (2*ΙLn L2-Ln L0Ι)** | **LRT P-value** |
| --- | --- | --- | --- | --- | --- | --- | --- | --- | --- | --- |
|  | **np** | **Ln L0** | **Estimates of parameters （*d*_N_/*d*s = *ω*）** | **np** | **Ln L2** | **Estimates of parameters （*d*_N_/*d*s = *ω*）** | | |  |  |
|  |  |  |  |  |  | ***ω0*** | ***ω1*** | **Median *ω*** |  |  |
| *acc*D | 32 | -2515.135033 | 0.34532 | 33 | -2514.938237 | 0.37437 | 0.2708 | 0.322585 | 0.393592 | 0.530417311 |
| *atp*A | 32 | -2268.625285 | 0.03476 | 33 | -2268.55052 | 0.04193 | 0.0001 | 0.021015 | 0.14953 | 0.698984912 |
| *atp*B | 32 | -2249.858367 | 0.09468 | 33 | -2249.858168 | 0.0953 | 0.0001 | 0.0477 | 0.000398 | 0.984083309 |
| *atp*E | 32 | -552.917591 | 0.06032 | 33 | -552.754439 | 0.0001 | 0.07219 | 0.036145 | 0.326304 | 0.567843575 |
| *atp*F | 32 | -856.577375 | 0.18535 | 33 | -856.564279 | 0.1779 | 60.19247 | 30.185185 | 0.026192 | 0.871432304 |
| *atp*H | 32 | -330.909944 | 0.08261 | 33 | -328.624231 | 0.0001 | 999 | 499.50005 | 4.571426 | 0.032509494 |
| *atp*I | 32 | -1111.300862 | 0.06104 | 33 | -1110.153053 | 0.09294 | 0.0001 | 0.04652 | 2.295618 | 0.129739565 |
| *ccs*A | 32 | -1763.717266 | 0.40278 | 33 | -1763.201572 | 0.35014 | 1.65232 | 1.00123 | 1.031388 | 0.309832889 |
| *cem*A | 32 | -1093.738346 | 0.51293 | 33 | -1093.738366 | 0.51291 | 1.98306 | 1.247985 | 4E-05 | 0.994953769 |
| *clp*P | 32 | -1011.57991 | 1.3794 | 33 | -1011.444774 | 1.19253 | 999 | 500.096265 | 0.270272 | 0.603149371 |
| *inf*A | 32 | -337.496647 | 0.06655 | 33 | -337.496681 | 0.06655 | 2.26717 | 1.16686 | 6.8E-05 | 0.99342055 |
| *mat*K | 32 | -2808.735477 | 0.4458 | 33 | -2808.735499 | 0.44579 | 1.3244 | 0.885095 | 4.4E-05 | 0.994707471 |
| *ndh*A | 32 | -1686.818764 | 0.18996 | 33 | -1686.411694 | 0.15183 | 8.92043 | 4.53613 | 0.81414 | 0.366899082 |
| *ndh*B | 32 | -2108.103076 | 0.263 | 33 | -2107.588767 | 0.32846 | 0.0001 | 0.16428 | 1.028618 | 0.310483481 |
| *ndh*C | 32 | -555.634987 | 0.06556 | 33 | -554.67944 | 0.11141 | 0.0001 | 0.055755 | 1.911094 | 0.166841786 |
| *ndh*D | 32 | -2601.44428 | 0.30587 | 33 | -2601.436766 | 0.30146 | 2.10594 | 1.2037 | 0.015028 | 0.902432769 |
| *ndh*E | 32 | -451.982039 | 0.03957 | 33 | -450.782176 | 0.0001 | 999 | 499.50005 | 2.399726 | 0.121356507 |
| *ndh*F | 32 | -3888.897689 | 0.24279 | 33 | -3887.234101 | 0.20417 | 0.41468 | 0.309425 | 3.327176 | 0.068143786 |
| *ndh*G | 32 | -839.596998 | 0.22683 | 33 | -839.597067 | 0.22681 | 2.1603 | 1.193555 | 0.000138 | 0.990627194 |
| *ndh*H | 32 | -1847.24391 | 0.08903 | 33 | -1845.812054 | 0.05512 | 8.74966 | 4.40239 | 2.863712 | 0.09059821 |
| *ndh*I | 32 | -833.192039 | 0.11238 | 33 | -832.466387 | 0.07715 | 0.34116 | 0.209155 | 1.451304 | 0.22831883 |
| *ndh*J | 32 | -833.192424 | 0.11238 | 33 | -832.466676 | 0.07712 | 0.35261 | 0.214865 | 1.451496 | 0.228288058 |
| *ndh*K | 32 | -1003.014336 | 0.10323 | 33 | -1002.805144 | 0.06341 | 12.31843 | 6.19092 | 0.418384 | 0.5177445 |
| *pet*A | 32 | -1440.256399 | 0.07518 | 33 | -1440.256666 | 0.07517 | 2.17701 | 1.12609 | 0.000534 | 0.981563773 |
| *pet*B | 32 | -972.717653 | 0.02313 | 33 | -971.838996 | 0.0001 | 0.05834 | 0.02922 | 1.757314 | 0.184959896 |
| *pet*D | 32 | -791.60969 | 0.06743 | 33 | -791.361539 | 0.05658 | 573.82814 | 286.94236 | 0.496302 | 0.481129514 |
| *pet*G | 32 | -156.791514 | 0.53031 | 33 | -156.791514 | 0.53032 | 1.34883 | 0.939575 | 0 | 1 |
| *pet*L | 32 | -131.928959 | 0.0001 | 33 | -131.928957 | 0.0001 | 1.86695 | 0.933525 | 4E-06 | 0.998404232 |
| *pet*N | 32 | -108.307713 | 1.40253 | 33 | -108.307728 | 2.08595 | 2.24145 | 2.1637 | 3E-05 | 0.995629828 |
| *psa*A | 32 | -3288.819302 | 0.05023 | 33 | -3283.900701 | 0.0001 | 2.22502 | 1.11256 | 9.837202 | 0.001710174 |
| *psa*B | 32 | -3242.279495 | 0.03435 | 33 | -3242.241434 | 0.03097 | 0.4179 | 0.224435 | 0.076122 | 0.782623217 |
| *psa*C | 32 | -397.230805 | 0.06364 | 33 | -397.085224 | 0.04566 | 999 | 499.52283 | 0.291162 | 0.589476854 |
| *psa*I | 32 | -162.917873 | 0.41742 | 33 | -162.917875 | 0.41742 | 1.86111 | 1.139265 | 4E-06 | 0.998404232 |
| *psa*J | 32 | -240.282745 | 0.15638 | 33 | -239.589541 | 0.2058 | 0.0001 | 0.10295 | 1.386408 | 0.23901264 |
| *psb*A | 32 | -1600.587374 | 0.04677 | 33 | -1599.717135 | 0.06385 | 0.0001 | 0.031975 | 1.740478 | 0.187078272 |
| *psb*B | 32 | -2282.851285 | 0.13158 | 33 | -2282.787464 | 0.13909 | 0.09156 | 0.115325 | 0.127642 | 0.720889532 |
| *psb*C | 32 | -2073.549918 | 0.06143 | 33 | -2071.751529 | 0.11865 | 0.0001 | 0.059375 | 3.596778 | 0.057891671 |
| *psb*D | 32 | -1588.982598 | 0.03326 | 33 | -1588.225687 | 0.0495 | 0.0001 | 0.0248 | 1.513822 | 0.218556808 |
| *psb*E | 32 | -355.411002 | 0.0001 | 33 | -355.410962 | 0.0001 | 226.23137 | 113.115735 | 8E-05 | 0.992863599 |
| *psb*F | 32 | -169.056403 | 0.0001 | 33 | -169.056399 | 0.0001 | 2.26862 | 1.13436 | 8E-06 | 0.997743245 |
| *psb*H | 32 | -325.596588 | 0.07422 | 33 | -325.143793 | 0.12374 | 0.0001 | 0.06192 | 0.90559 | 0.341287236 |
| *psb*I | 32 | -151.775077 | 0.0001 | 33 | -151.77478 | 176.88187 | 0.0001 | 88.440985 | 0.000594 | 0.980555791 |
| *psb*J | 32 | -152.08401 | 1.84302 | 33 | -152.084009 | 2.37284 | 2.2628 | 2.31782 | 2E-06 | 0.998871621 |
| *psb*K | 32 | -247.844716 | 0.0001 | 33 | -247.844794 | 0.0001 | 0.0001 | 0.0001 | 0.000156 | 0.990034684 |
| *psb*L | 32 | -161.701977 | 0.0001 | 33 | -161.701977 | 0.0001 | 0.0001 | 0.0001 | 0 | 1 |
| *psb*M | 32 | -147.431108 | 0.0001 | 33 | -147.431112 | 0.0001 | 1.87011 | 0.935105 | 8E-06 | 0.997743245 |
| *psb*N | 32 | -182.526947 | 0.0001 | 33 | -182.526947 | 0.0001 | 0.0001 | 0.0001 | 0 | 1 |
| *psb*T | 32 | -162.934486 | 0.0001 | 33 | -162.934486 | 0.0001 | 0.0001 | 0.0001 | 0 | 1 |
| *psb*Z | 32 | -235.225157 | 999 | 33 | -235.225158 | 999 | 0.0001 | 499.50005 | 2E-06 | 0.998871621 |
| *rbc*L | 32 | -2112.126119 | 0.08318 | 33 | -2110.289605 | 0.12573 | 0.0001 | 0.062915 | 3.673028 | 0.055299634 |
| *rpl*14 | 32 | -487.122136 | 0.0001 | 33 | -487.122118 | 0.0001 | 695.27798 | 347.63904 | 3.6E-05 | 0.995212721 |
| *rpl*16 | 32 | -628.675452 | 0.27484 | 33 | -628.597901 | 0.30748 | 0.17897 | 0.243225 | 0.155102 | 0.693706619 |
| *rpl*2 | 32 | -1039.540533 | 0.06046 | 33 | -1038.76939 | 0.14212 | 0.0001 | 0.07111 | 1.542286 | 0.214277859 |
| *rpl*20 | 32 | -659.423701 | 0.64524 | 33 | -655.743815 | 0.1619 | 999 | 499.58095 | 7.359772 | 0.006669925 |
| *rpl*22 | 32 | -854.666676 | 0.50836 | 33 | -854.540557 | 0.58083 | 0.35983 | 0.47033 | 0.252238 | 0.615503623 |
| *rpl*23 | 32 | -370.966063 | 893.90477 | 33 | -370.966063 | 738.73702 | 20.52606 | 379.63154 | 0 | 1 |
| *rpl*33 | 32 | -309.479271 | 0.12845 | 33 | -309.219504 | 0.14997 | 0.0001 | 0.075035 | 0.519534 | 0.471040532 |
| *rpl*36 | 32 | -171.681139 | 0.13863 | 33 | -171.039247 | 0.29193 | 0.0001 | 0.146015 | 1.283784 | 0.257196648 |
| *rpo*A | 32 | -1754.280816 | 0.31457 | 33 | -1753.984786 | 0.28221 | 0.45017 | 0.36619 | 0.59206 | 0.441623633 |
| *rpo*B | 32 | -5009.328332 | 0.17388 | 33 | -5009.238907 | 0.17296 | 0.0001 | 0.08653 | 0.17885 | 0.672363401 |
| *rpo*C1 | 32 | -3115.759439 | 0.20023 | 33 | -3113.841727 | 0.26965 | 0.0001 | 0.134875 | 3.835424 | 0.050180298 |
| *rpo*C2 | 32 | -6700.26944 | 0.3029 | 33 | -6700.264093 | 0.29991 | 1.59469 | 0.9473 | 0.010694 | 0.917636149 |
| *rps*11 | 32 | -605.939949 | 0.0001 | 33 | -605.939949 | 0.0001 | 0.0001 | 0.0001 | 0 | 1 |
| *rps*12 | 32 | -493.211756 | 0.0001 | 33 | -493.211756 | 0.0001 | 0.0001 | 0.0001 | 0 | 1 |
| *rps*14 | 32 | -438.063026 | 0.0001 | 33 | -438.063026 | 0.0001 | 0.0001 | 0.0001 | 0 | 1 |
| *rps*15 | 32 | -383.391272 | 0.12744 | 33 | -383.391289 | 0.12744 | 2.00189 | 1.064665 | 3.4E-05 | 0.9953476 |
| *rps*18 | 32 | -430.948875 | 0.39892 | 33 | -430.782147 | 0.48998 | 0.0001 | 0.24504 | 0.333456 | 0.563631122 |
| *rps*19 | 32 | -449.663243 | 0.34844 | 33 | -447.926308 | 0.2326 | 999 | 499.6163 | 3.47387 | 0.062345291 |
| *rps*2 | 32 | -1051.272324 | 0.2484 | 33 | -1049.323179 | 0.53683 | 0.0581 | 0.297465 | 3.89829 | 0.048335282 |
| *rps*3 | 32 | -1058.023848 | 0.07745 | 33 | -1057.897781 | 0.06727 | 423.87517 | 211.97122 | 0.252134 | 0.615576455 |
| *rps*4 | 32 | -904.026773 | 0.0997 | 33 | -903.882504 | 0.08141 | 84.98809 | 42.53475 | 0.288538 | 0.591158941 |
| *rps*7 | 32 | -627.331565 | 0.29268 | 33 | -627.331575 | 0.29268 | 2.28042 | 1.28655 | 2E-05 | 0.996431764 |
| *rps*8 | 32 | -640.911585 | 0.12464 | 33 | -640.851699 | 0.11505 | 651.40595 | 325.7605 | 0.119772 | 0.729281906 |
| *ycf*1 | 32 | -10455.63321 | 1.02997 | 33 | -10455.56493 | 1.00966 | 1.09423 | 1.051945 | 0.136558 | 0.711727091 |
| *ycf*2 | 32 | -9318.824231 | 0.9829 | 33 | -9318.511206 | 0.87471 | 1.34568 | 1.110195 | 0.62605 | 0.428807913 |
| *ycf*3 | 32 | -736.493101 | 0.0001 | 33 | -736.492871 | 0.0001 | 0.0001 | 0.0001 | 0.00046 | 0.982888595 |
| *ycf*4 | 32 | -877.19989 | 0.24404 | 33 | -876.949497 | 0.20496 | 183.7418 | 91.97338 | 0.500786 | 0.479154964 |
