# Supplementary material for: Complete chloroplast genomes of eight Delphinium taxa (Ranunculaceae) endemic to Xinjiang, China: insights into genome structure, comparative analysis, and phylogenetic relationships
Source: BMC Plant Biol. 2024 Jun 26;24:600. doi: 10.1186/s12870-024-05279-y (PMC11201361; doi:10.1186/s12870-024-05279-y)
Supplement: Supplementary file 9 — Supplementary Material 9 [file 12870_2024_5279_MOESM9_ESM.docx]

**Table S9** The number of parsimony informative characters in 15 genes.

| **Gene** | **Number of Parsimony informative Characters** |
| --- | --- |
| *acc*D-*rbc*L | 163 |
| *ccs*A-*ndh*D | 160 |
| *trn*K-*trn*Q | 74 |
| *ndh*F-*trn*L | 526 |
| *psa*J-*trn*P | 459 |
| *psb*D-*trn*T | 490 |
| *rpo*B-*trn*C | 434 |
| *rpl*16 | 14 |
| *rpl*33 | 9 |
| *rps*15 | 16 |
| *rps*18 | 6 |
| *ycf*1 | 700 |
| *trn*S-*trn*G | 158 |
| *trn*L | 0 |
| *rbc*L | 29 |
