# Supplementary material for: Complete chloroplast genomes of eight Delphinium taxa (Ranunculaceae) endemic to Xinjiang, China: insights into genome structure, comparative analysis, and phylogenetic relationships
Source: BMC Plant Biol. 2024 Jun 26;24:600. doi: 10.1186/s12870-024-05279-y (PMC11201361; doi:10.1186/s12870-024-05279-y)
Supplement: Supplementary file 10 — Supplementary Material 10 [file 12870_2024_5279_MOESM10_ESM.docx]

**TABLE S10** The chromosome information for *Delphinium* taxa in this study.

| **Taxon** | **Chromosome Number (2n)** | **Reference** |
| --- | --- | --- |
| *Delphinium aemulans* Nevski (*) | ? |  |
| *D. anthriscifolium* Hance | 16 | Yuan and Yang (2008) |
| *D. brunonianum* Royle | 16 | Al-Kelidar and Richards (1981) |
| *D. candelabrum* var. *monanthum* (Hand.-Mazz.) W. T. Wang | 16 | Liu and He (1999) |
| *D. ceratophorum* Franch. | 16 | Yuan & Yang (2008) |
| *D. elatum* var. *sericeum* W. T. Wang (*) | ? |  |
| *D. iliense* Huth (*) | 16 | Li (2019) |
| *D. maackianum* Regel | 16, 32 | Volkova and Ulanova (1986); Kurita (1958) |
| *D. mollifolium* W. T. Wang (*) | 32 | Li (2022; unpublished, showing in Fig. S1) |
| *D. naviculare* var. *lasiocarpum* W. T. Wang (*) | 16 | Li (2019) |
| *D. sauricum* Schischk. (*) | 16 | Li (2019) |
| *D. shawurense* W. T. Wang (*) | 32 | Li (2019) |
| *D. winklerianum* Huth (*) | 16 | Li (2019) |
| *D. yunnanense* Franch. | 16 | Yuan & Yang (2008) |
