# Supplementary material for: Complete chloroplast genomes of eight Delphinium taxa (Ranunculaceae) endemic to Xinjiang, China: insights into genome structure, comparative analysis, and phylogenetic relationships
Source: BMC Plant Biol. 2024 Jun 26;24:600. doi: 10.1186/s12870-024-05279-y (PMC11201361; doi:10.1186/s12870-024-05279-y)
Supplement: Supplementary file 11 — Supplementary Material 11 [file 12870_2024_5279_MOESM11_ESM.docx]

**Table S11**. Collecting information, voucher specimen and identification for the eight taxa of *Delphinium* endemic to Xinjiang, China in the study.

| **Genus** | **Species** | **Locality** | **Voucher and Identifier** | **Longitude/Latitude** |
| --- | --- | --- | --- | --- |
| *Delphinium* L. | *D. aemulans* Nevski | Qarabura Town, Yumin County, Tacheng Prefecture | Huimin Li 1280 (NAS); Huimin Li | 82°41′33″/ 46°03′50.12″ |
|  | *D. elatum* var. *sericeum* W. T. Wang | Kanas Lake, Burqin County, Altay Prefecture | H.M. Li 1265 (NAS); Huimin Li | 87°01′58″/48°42′43″ |
|  | *D. iliense* Huth | Qapqal Xibe Autonomous County, Ili Kazakh Autonomous Prefecture | Huimin Li 1285 (NAS); Huimin Li | 81°08′07.12″/43°14′14″ |
|  | *D. mollifolium* W. T. Wang | Taldi Town, Xinyuan County, Ili Kazakh Autonomous Prefecture | Huimin Li 1295 (NAS); Huimin Li | 83°15′45.87″/43°16′54″ |
|  | *D. naviculare* var. *lasiocarpum* W. T. Wang | Kongdeneng Town, Gongliu County, Ili Kazakh Autonomous Prefecture | Huimin Li 1293 (NAS); Huimin Li | 82°37′21″/43°05′53″ |
|  | *D. sauricum* Schischk. | Ulastay Town, Jeminay County, Altay Prefecture | Huimin Li 1266 (NAS); Huimin Li | 86°05′04″/47°05′41″ |
|  | *D. shawurense* W. T. Wang | Hoboksar, Tacheng Prefecture | Huimin Li 1271 (NAS); Huimin Li | 85°07′00.14″/46°56′25″ |
|  | *D. winklerianum* Huth | Taldi Town, Xinyuan County, Ili Kazakh Autonomous Prefecture | Huimin Li 1299 (NAS); Huimin Li | 83°16′33″/43°15′39″ |
