# Supplementary material for: Complete chloroplast genomes of eight Delphinium taxa (Ranunculaceae) endemic to Xinjiang, China: insights into genome structure, comparative analysis, and phylogenetic relationships
Source: BMC Plant Biol. 2024 Jun 26;24:600. doi: 10.1186/s12870-024-05279-y (PMC11201361; doi:10.1186/s12870-024-05279-y)
Supplement: Supplementary file 12 — Supplementary Material 12 [file 12870_2024_5279_MOESM12_ESM.docx]

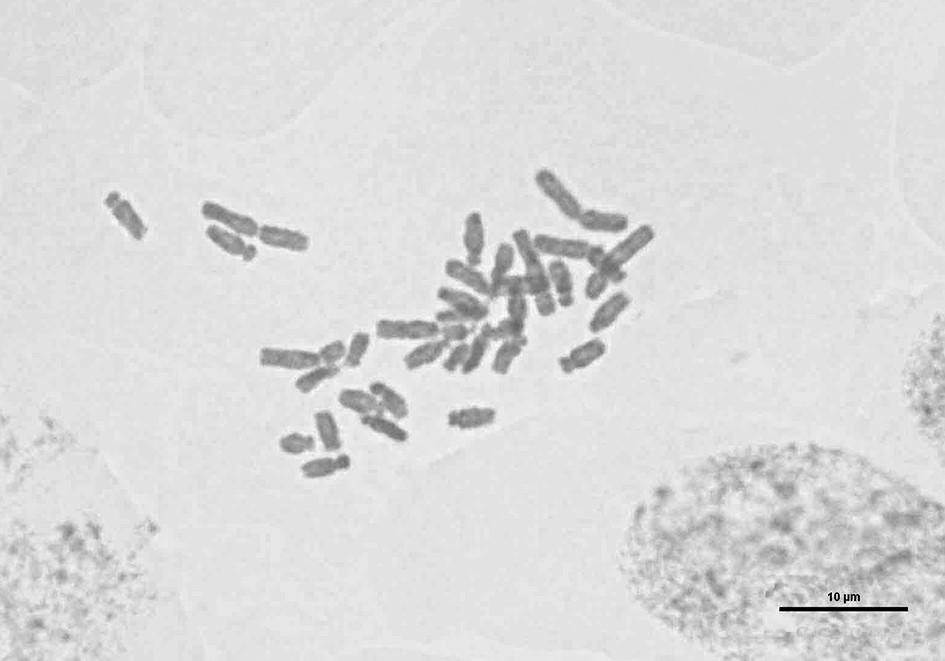


**Fig. S1** Photomicrographs of mitotic metaphase chromosomes (2*n* = 32) in *Delphinium mollifolium* from Xinjiang, China.
